# Exploring the Scope of Tandem Palladium and Isothiourea Relay Catalysis for the Synthesis of α-Amino Acid Derivatives

**DOI:** 10.3390/molecules25102463

**Published:** 2020-05-25

**Authors:** Jacqueline Bitai, Alexandra M. Z. Slawin, David B. Cordes, Andrew D. Smith

**Affiliations:** EaStCHEM, School of Chemistry, University of St Andrews, North Haugh, St Andrews, Fife KY16 9ST, UK; jb344@st-andrews.ac.uk (J.B.); amzs@st-andrews.ac.uk (A.M.Z.S.); dbc21@st-andrews.ac.uk (D.B.C.)

**Keywords:** isothiourea, palladium, enantioselective catalysis, amino acids, [2,3]-rearrangement, *N*-allylation

## Abstract

The scope and limitations of a tandem N-allylation/[2,3]-rearrangement protocol are investigated through the incorporation of a variety of functional groups within an allylic phosphate precursor. This method uses readily accessible N,N-dimethylglycine aryl esters and functionalized allylic phosphates, forming quaternary ammonium salts in situ in the presence of a palladium catalyst. Subsequent enantioselective [2,3]-sigmatropic rearrangement, promoted by the chiral isothiourea tetramisole, generates α-amino acid derivatives with two contiguous stereocenters. The incorporation of electron-withdrawing ester and amide groups gave the best results, furnishing the desired products in moderate to good yields (29–70%), with low diastereocontrol (typically 60:40 dr) but high enantioselectivity (up to 90:10 er). These results indicate that substrate–catalyst interactions in the proposed transition state are sensitive to the substitution pattern of the substrates.

## 1. Introduction

**Figure 1:**
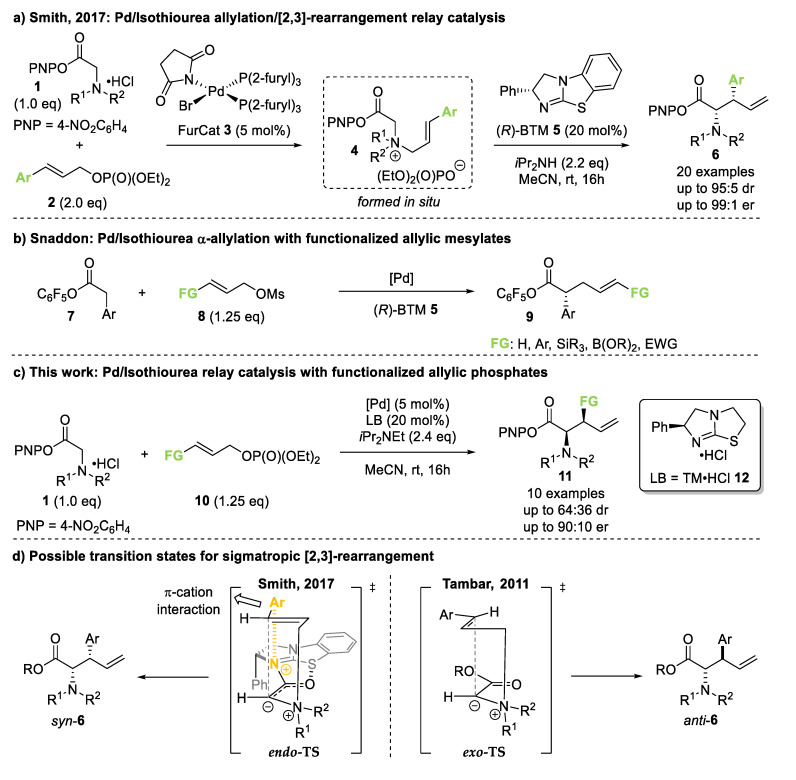
Dual catalytic processes employing isothiourea and palladium catalysts: (**a**) tandem *N*-allylation/[2,3]-rearrangement relay catalysis; (**b**) ammonium enolate and allylation cooperative catalysis; (**c**) this work; (**d**) possible transition states in [2,3]-rearrangement determining product diastereoselectivity.

Enantioenriched α-amino acids are an important class of compounds, used in pharmaceuticals [1], as chiral building blocks in peptide chemistry [2] and total synthesis [3], and as chiral ligands and organocatalysts [4]. The constant development in these areas requires facile and reliable access to modified α-amino acids beyond the naturally occurring selection, meaning that the development of new methods for the enantioselective synthesis of unnatural α-amino acids is of great interest [5]. One synthetic strategy involves the direct stereoselective α-alkylation of amino acid ester derivatives [6]. Catalytic methods for this transformation include enantioselective transition metal catalysis or phase transfer organocatalysis, which have been used to incorporate α-aryl, α-allyl or α-alkyl substituents [7]. A conceptually different approach to the preparation of α-functionalized amino acids is through a [2,3]-sigmatropic rearrangement of allylic ammonium ylides. In 2014, we reported the first catalytic, enantioselective [2,3]-sigmatropic rearrangement of these species, using the isothiourea catalyst benzotetramisole (BTM) to generate *syn*-α-amino acid derivatives with two contiguous stereocenters with excellent stereoselectivity [8]. This methodology could be expanded by merging the organocatalytic rearrangement with a palladium-catalyzed *N*-allylation in a dual catalytic process. Palladium-catalyzed *N*-allylation between allylic phosphates **2** and glycine aryl esters **1** formed quaternary ammonium salts **4** in situ, which underwent isothiourea-catalyzed [2,3]-rearrangement, giving the desired *syn*-α-amino acid derivatives **6** in improved yield and greater scope compared to the previous protocol, whilst maintaining excellent stereocontrol (Figure 1a) [9]. Although a variety of *N*-substituents were tolerated, this methodology is currently limited to the use of allylic phosphates derived from cinnamyl alcohols.

In recent work, Snaddon and co-workers demonstrated broad functional group tolerance in a related dual catalytic system, employing chiral isothiourea and palladium catalysis in the direct α-allylation of aryl acetic acid esters (Figure 1b) [10]. This protocol tolerates the incorporation of various functional groups (FG in Figure 1b) within the allylic fragment, including aryl, boron [11], silicon [12], and electron-withdrawing carbonyl [13] substituents, allowing for the construction of functionalized products with excellent stereoselectivity. Inspired by this work, we questioned if more diverse functional groups could also be tolerated in the previously developed *N*-allylation/[2,3]-rearrangement protocol (Figure 1c). We specifically questioned (i) if the *N*-allylation and subsequent rearrangement would still proceed when the aryl-substituent within the cinnamyl unit was substituted for another functional group and (ii) if the desired products would still be obtained with high diastereo- and enantioselectivity. The latter point was especially intriguing, as the aryl substituent has been shown to be a key control element in the rearrangement process by computational analysis [14]. In the proposed *endo* transition state, this aryl unit provides a stabilizing π-cation interaction, contributing to the observed *syn*-selectivity (Figure 1d, left). In recent work both by ourselves and Shiina and co-workers concerning the isothiourea-catalyzed kinetic resolution of secondary and tertiary alcohols, a carbonyl group had also been identified as a viable partner for a stabilizing C=O•••isothiouronium interaction, which is primarily electrostatic in nature [15,16,17], leading to the postulate that this unit could be incorporated within the allylic fragment in this *N*-allylation/[2,3]-rearrangement protocol. Without such stabilizing interactions, [2,3]-rearrangements are proposed to proceed preferentially through an *exo* transition state, yielding the corresponding *anti*-products (Figure 1d, right). This has been described by Tambar and co-workers in a related, Brønsted base-catalyzed *N*-allylation/[2,3]-rearrangement protocol, including examples on α-amino ester derivatives [18].

In this manuscript, we demonstrate the feasibility of incorporating varying substituents within the allylic phosphate, leading to the desired α-amino acid derivatives following *N*-allylation and [2,3]-rearrangement. The stereochemical outcome of the rearrangement shows great sensitivity regarding the nature of the substituent within the allylic fragment.

## 2. Results and Discussion

### 2.1. Initial Functional Group Assessment

Initial investigations focused on identifying functional groups that were tolerated in the tandem *N*-allylation/[2,3]-rearrangement protocol by probing the electronic and steric limitations within the allylic fragment. Readily accessible allylic alcohols containing an electron-withdrawing C(3)-ester and an electron-donating C(3)-silyl substituent, a silyl-protected homoallylic alcohol functional group, and a C(2)-phenyl branched allylic substrate were chosen. The corresponding allylic phosphates **14**–**17** and *N*,*N*-dimethyl 4-nitrophenyl ester hydrochloride salt **13** were subjected to relay catalysis conditions established in the previous work, using FurCat **3** (5 mol%) as a stable palladium catalyst precursor, (±)-BTM **5** as Lewis base catalyst and *i*Pr_2_NH as base in MeCN at room temperature (Table 1). While ester-containing phosphate **14** gave the product in a promising 70% yield by ^1^H-NMR analysis, trimethylsilyl (SiMe_3_)-containing phosphate **15** did not show any reactivity. Considering the expected electronic requirements in the [2,3]-rearrangement step, these results are consistent with an electron-withdrawing group enhancing the electrophilicity at C(3) and facilitating rearrangement, with an electron-donating group having the opposite effect. Gratifyingly, phosphate **16** and sterically demanding phosphate **17** also furnished the desired products, albeit in lower yields (33% and 32%, respectively). Importantly, 4-nitrophenyl (PNP) ester product **21** obtained from a branched allylic substrate could only be observed when a mesylate was used instead of a phosphate as the leaving group, as has been reported by Snaddon and co-workers [19]. Unfortunately, the product could not be isolated as it proved unstable to column chromatography. Ethyl ester (**18**) could be isolated successfully at 61% yield as a mixture of diastereoisomers (60:40 dr), while rearrangement using *O*-TBDPS-protected phosphate gave alcohol (**20**) in 50:50 dr in combined 30% yield as fully separable diastereoisomers. As ethyl ester **18** gave better results in this initial assessment, it was taken for further optimization.

### 2.2. Reaction Optimization for Ethyl Ester Containing Allylic Phosphate

**Figure 2:**
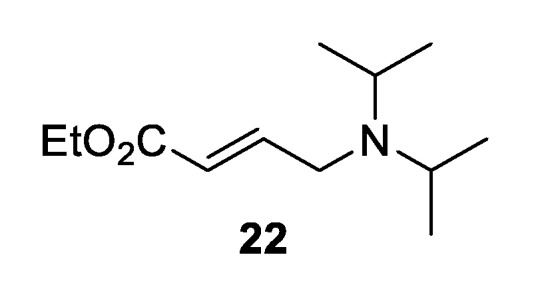
Identified side product during the optimization process.

During the purification of the crude material, several issues were encountered. The desired product **18** contained varying amounts of unreacted phosphate **14**, an unknown side product and isothiourea catalyst BTM. The chromatographic separation of these compounds proved difficult, which resulted in reduced isolated yields of ester **18**. The efforts to mitigate these problems are summarized in Table 2. The side product was identified as **22** (Figure 2), presumably resulting from addition of *i*Pr_2_NH to an intermediate π-allyl-Pd complex. The formation of **22** could be avoided by changing the base to *i*Pr_2_NEt, without effecting the catalytic transformation (Table 2, entry 2). Furthermore, changing the catalyst from BTM to tetramisole (TM) simplified purification without altering the observed yield or stereoselectivity (Table 2, entry 3). Subsequently, the amount of phosphate **14** could be successfully reduced to 1.25 equivalents without effecting the obtained yield (Table 2, entry 4). With these improved conditions in hand, PNP ester **18** could be isolated in 70% yield as an inseparable mixture of diastereoisomers in 60:40 dr, with high enantioselectivity (90:10 er_maj_, 70:30 er_min_) (Table 2, entry 5). The relative and absolute configurations within the major diastereoisomers were assigned by analogy to that previously reported for related isothiourea-catalyzed [2,3]-rearrangement reactions [9].

**Figure 3:**
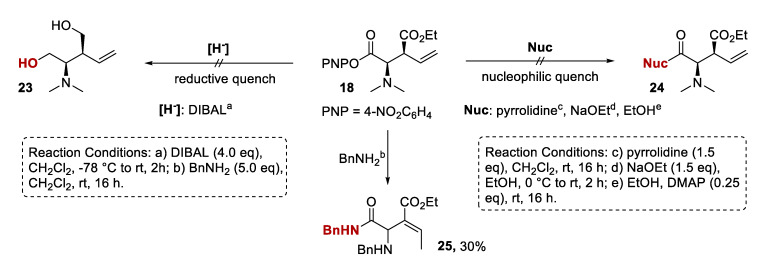
Attempted derivatization of PNP ester **18** with different reagents.

Another concern was the variation in isolated yields in Table 2 compared to the yield expected by ^1^H-NMR analysis of the crude reaction product, indicative of the PNP ester products being unstable to purification by column chromatography. This is a recognized problem in the literature, with the initial PNP ester products commonly converted into stable derivatives [8,9]. Based on established methods within our group, a number of different nucleophiles were trialed for these transformations (Figure 3). Unfortunately, none of the reductive or nucleophilic quenches yielded the desired product but resulted in decomposition. As a representative example, when BnNH_2_ was employed as a nucleophile, side product **25** could be isolated in 30% yield (Figure 3). Although the desired benzylamide formation had proceeded, substitution of the NMe_2_ group and migration of the double bond into conjugation with the ester functionality were also observed (see Supplementary Materials Figure S1 and Appendix in Supplementary Materials Figure A1). A similar migration was observed by ^1^H-NMR analysis of the crude material upon addition of NaOEt as a nucleophile, indicating that the position α to the ester group is readily deprotonated under these reaction conditions. It was hypothesized that changing the ester functionality to a less electron-withdrawing amide substituent could minimize this unwanted alkene migration as the corresponding α C-H should be less acidic.

### 2.3. Amide Containing Allylic Phosphates

#### 2.3.1. Initial Assessment

**Figure 4:**
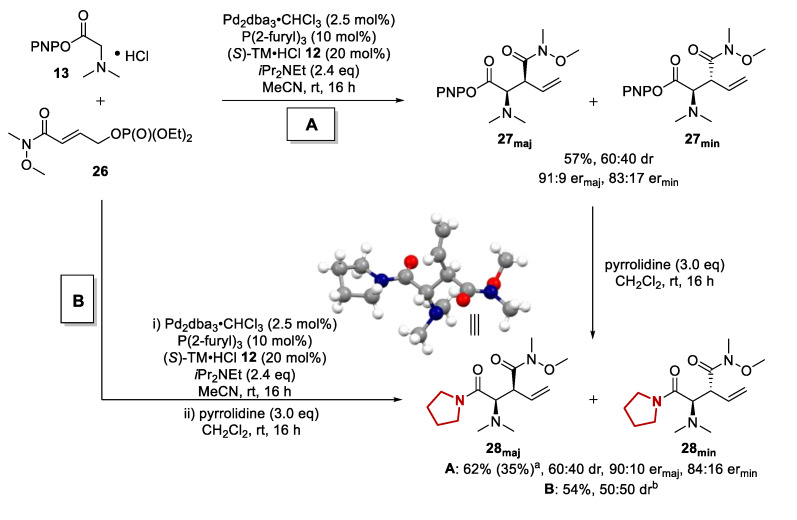
Initial assessment of Weinreb amide containing phosphate in a tandem allylation/[2,3]-rearrangement process and confirmation of relative configuration by single-crystal X-ray diffraction analysis for *syn* diastereoisomer. ^a^ Value in parentheses is isolated yield over two steps. ^b^ Diastereoisomers not fully separable. er not determined.

To test the validity of our hypothesis, Weinreb amide containing phosphate **26** was subjected to the previously developed catalysis conditions. Gratifyingly, good reactivity could be observed, yielding the desired PNP ester product **27** in 57% isolated yield in 60:40 dr and promising enantioselectivity (91:9 er_maj_, 83:17 er_min_) (Figure 4). The diastereoisomers of PNP ester **27** could be readily separated to give **27_maj_** and **27_min_** with >95:5 dr in each case. Importantly, subsequent derivatization of the isolated, single diastereoisomers with pyrrolidine furnished the corresponding amide products **28_maj_** and **28_min_** without loss of enantiointegrity or the formation of unwanted side products. In addition, a crystal structure could be obtained for the major diastereoisomer, revealing its relative configuration to be *syn*. However, the overall yield for this approach was low, resulting in only 35% amide product isolated after two steps. Alternatively, a one-pot derivatization procedure was investigated. Direct addition of the pyrrolidine nucleophile to the catalysis reaction mixture after 16 h resulted in an improved 54% isolated yield of the derivatized product without affecting the diastereomeric ratio. However, the diastereoisomers of the final amide product could no longer be fully separated.

**Figure 5:**
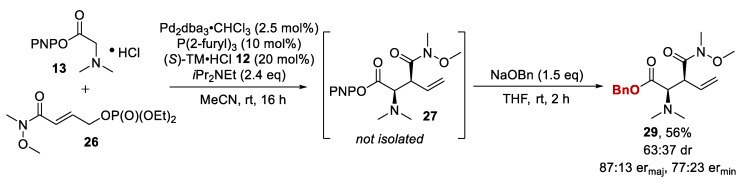
Optimized one-pot derivatization process with sodium benzylate.

An alternative derivatization using sodium benzylate (NaOBn) was also investigated. However, subjecting the crude mixture of PNP ester **27** to NaOBn in THF after the removal of MeCN did not yield the desired benzyl ester. Importantly, for the desired derivatization to occur, the palladium catalyst had to be removed from the crude mixture by filtration through silica prior to addition of the nucleophile. Following these reaction conditions, the benzyl ester **29** was generated in 63:37 dr, with purification allowing separation of the diastereoisomers to >95:5 dr purity in 56% overall isolated yield and without loss of enantiopurity (87:13 er_maj_, 77:23 er_min_) (Figure 5).

#### 2.3.2. Scope of Amide Containing Allylic Phosphates

**Figure 6:**
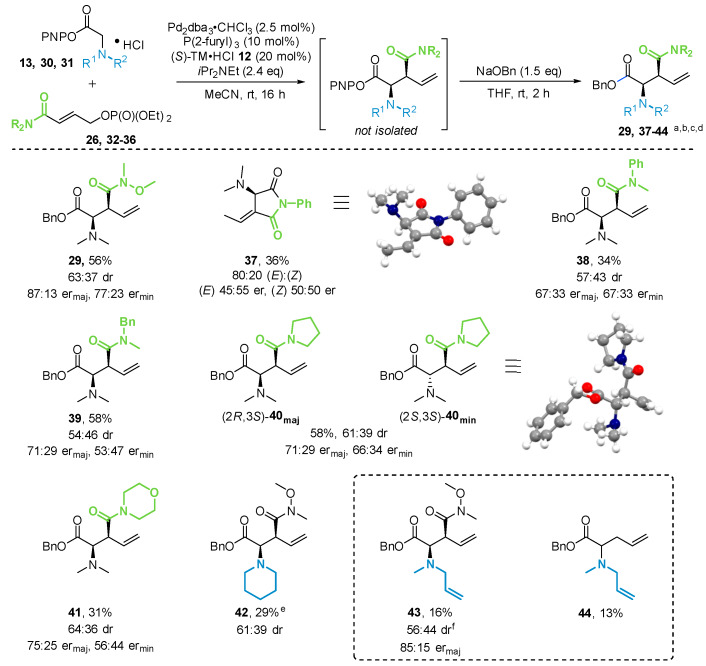
Scope of amide-containing allylic phosphates and glycine esters. ^a^ Reactions performed on a 0.3-mmol scale. ^b^ Combined yields of isolated diastereoisomers. ^c^ dr determined by ^1^H-NMR analysis of the crude material. ^d^ er determined by chiral stationary phase HPLC analysis. ^e^ Reaction run for 84 h. Diastereoisomers could not be separated. er could not be determined. ^f^ er for minor diastereoisomer could not be determined.

Having established the optimal conditions for the relay catalysis and derivatization process, the scope of allylic phosphates containing differently substituted amides was explored (Figure 6) (see Supplementary Materials for experimental details). Weinreb amide containing product **29** could be isolated in 56% yield with a 63:37 dr in favor of the *syn*-diastereoisomer with high enantioselectivity (er_maj_ 87:13, er_min_ 77:23). Interestingly, use of a secondary amide (CONHPh) containing phosphate **32** did not yield the anticipated product, but instead resulted in the formation of cyclic imide **37** in a 80:20 ratio of fully separable (*E*)- and (*Z*)-isomers in 36% combined isolated yield as racemic mixtures (see Appendix in Supplementary Materials Figure A2). The structure of imide (*E*)-**37** was unambiguously identified by single-crystal X-ray diffraction analysis. Subsequently, different acyclic (**38**, **39**) and cyclic (**40**, **41**) tertiary amides were investigated. The nature of the substituents on the tertiary amides did not dramatically influence the observed stereoselectivities, with diastereomeric ratios between 54:46 and 67:33, but generally lower enantioselectivities than the model substrate. Amides containing a *N*-Ph (**37**, **38**) or *N*-morpholinyl (**41**) substituent also resulted in lower yields. All products could be isolated as separable diastereoisomers. The relative and absolute configuration for the minor diastereoisomer of **40** could be unambiguously assigned by single-crystal X-ray diffraction analysis. Next, variation of the *N*-substituents within the glycine ester was investigated. Consistent with previous work [9], the cyclic *N*-piperidinyl substituted glycine ester **30** showed reduced reactivity, giving the product **42** in only 29% isolated yield, even after a prolonged reaction time of 84 h, but with similar diastereoselectivity (61:39 dr). Unfortunately, the diastereoisomers could not be separated, and the enantiomeric ratios could not be determined. When an unsymmetrical *N*-allyl-*N*-methylglycine ester **31** was used, the desired product **43** could only be isolated in 16% yield, but with high enantioselectivity consistent with a Weinreb amide substituent (85:15 er_maj_). Surprisingly, compound **44** was also isolated from the reaction in 13% yield. The formation of **44** is presumably the result of a [2,3]-rearrangement occurring through an *N*-allyl substituent and is consistent with prior exchange of the *N*-allyl amide unit for another unsubstituted allyl from a second glycine ester substrate (see Appendix in Supplementary Materials Figure A3). Even though rearrangement through different *N*-allyl substituents has been observed previously by Tambar and co-workers [18], this exchange of *N*-allyl units has not yet been reported for an *N*-allylation/rearrangement protocol. These examples indicate that the nature of the *N*-substituents on the glycine ester greatly influence the reactivity of the substrate, whereas the observed stereoselectivity is more susceptible to the amide substituents within the allylic phosphate.

#### 2.3.3. Mechanistic Control Experiments

**Figure 7:**
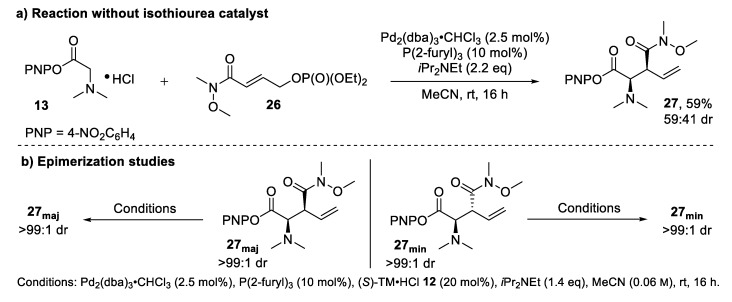
Mechanistic control experiments: (**a**) without isothiourea catalyst; (**b**) epimerization studies under standard catalysis conditions.

To gain further mechanistic insight, a series of control experiments was performed. In the absence of a palladium catalyst, no reaction occurred, with only starting materials returned. In contrast, the reaction still proceeded without the isothiourea catalyst, but only gave racemic product (Figure 7a), indicative of a Brønsted base-catalyzed reaction pathway. As a racemic background reaction was not observed for cinnamate-derived phosphates [9], this competitive reaction pathway may potentially account for the comparatively lower stereoselectivities observed in this work. Interestingly, even without the isothiourea catalyst, there is still a preference for the formation of the *syn*-diastereoisomer. This is in contrast to our previous observations, as well as by Tambar and co-workers, who reported a bias to generate *anti*-α-amino acid derivatives under Brønsted basic conditions for similar substrates. However, an unexpected preference to generate *syn*-amino acid derivates has been observed for an *N*-allylation/[2,3]-rearrangement sequence between proline derivatives and cinnamyl alcohol derivates bearing ortho-substituted aryl units [20]. This finding highlights the sensitivity of the [2,3]-rearrangement to substituent effects, which could also be a reason for the low diastereocontrol observed in this work. Further studies looked at potential epimerization under the conditions of the relay catalysis (Figure 7b). PNP ester **27** was isolated as separated diastereoisomers and each re-subjected to the catalysis conditions. After 16 h, the PNP esters **27_maj_** and **27_min_** were still present as single diastereoisomers in both cases, indicating that epimerization of the PNP ester product does not occur under the reaction conditions used for catalysis. However, epimerization of the acyl ammonium ion generated post-rearrangement cannot be ruled out as catalyst turnover is assumed to be irreversible based upon our previous work [14].

**Figure 8:**
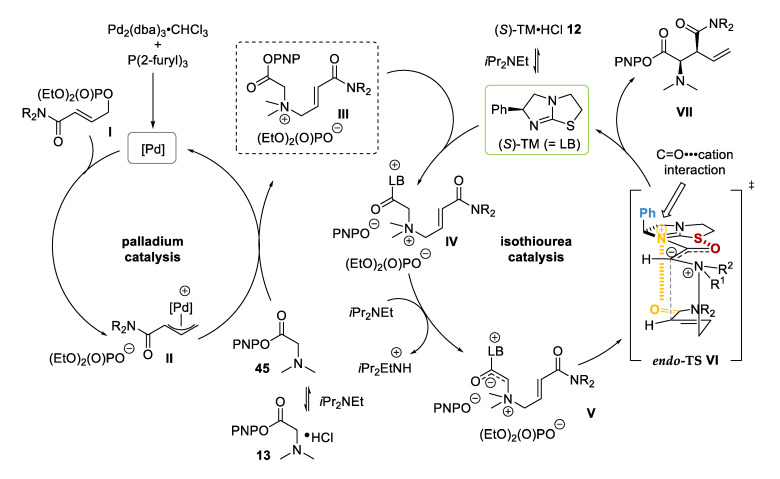
Proposed relay catalytic cycle.

The mechanism for the palladium and isothiourea *N*-allylation/[2,3]-rearrangement relay catalysis is proposed by analogy to related reactions (Figure 8) [9,14]. The active palladium catalyst [Pd] is formed in situ from Pd_2_(dba)_3_·CHCl_3_ and P(2-furyl)_3_. Coordination of the allylic phosphate followed by oxidative addition generates η^3^-Pd-π-allyl intermediate **II**. Nucleophilic attack of free-based glycine ester **45** and subsequent dissociation from the palladium catalyst releases allylic ammonium salt **III** as the key intermediate. Displacement of the aryl oxide by the free-based isothiourea TM gives dication **IV**, which can be deprotonated to form ammonium ylide **V**. Subsequent [2,3]-rearrangement under catalyst-directed stereocontrol followed by aryloxide-facilitated catalyst turnover yields product **VII**. The stereodirecting effect of the catalyst can be rationalized by *endo*-TS **VI**, displaying several key features. Ammonium ylide **V** is thought to have significant enolate character, favoring the (*Z*)-configuration. In addition, a stabilizing 1,5 O•••S interaction resulting from n_O_ to σ*_C-S_ overlap between the carbonyl oxygen and the isothiourea sulfur atom restricts the conformational freedom, locking the ylide in this position [14,21,22,23,24]. The stereodirecting phenyl group adopts a pseudoaxial position to minimize 1,2-strain, which forces the allylic fragment onto the opposite face of the catalyst, where the [2,3]-rearrangement takes place. The preference for the formation of the *syn* product via *endo*-TS is assumed to be a result of a favorable, predominantly electrostatic C=O•••cation interaction between the positively charged catalyst and the carbonyl substituent on the allylic fragment [15].

## 3. Materials and Methods

### 3.1. General Procedure for the Tandem Palladium and Isothiourea Relay Catalysis

A Schlenk tube was charged with FurCat **3** (5 mol%) or Pd_2_(dba)_3_·CHCl_3_ (2.5 mol%) and P(2-furyl)_3_ (10 mol%), (*S*)-TM·HCl (20 mol%) and PNP Ester **13** (1.0 eq). The tube was then evacuated and flushed with argon three times; degassed MeCN (0.06 M) was added and the mixture stirred for 10 min at room temperature. Subsequently, phosphate or mesylate (1.0–2.0 eq) and *i*Pr_2_NEt (2.4 eq) were added in this order and the reaction mixture stirred at room temperature for 16 h. An aliquot was taken, the solvent removed under reduced pressure and ^1^H-NMR spectroscopy of the crude mixture used to determine the dr. The reaction mixture was then filtered over a short plug of silica with MeCN and the filtrate concentrated under reduced pressure. The residue was purified by flash silica chromatography as specified or directly derivatized with NaOBn.

Derivatization with NaOBn: The crude reaction mixture was dissolved in anhydrous THF (0.05 M), freshly prepared NaOBn (1 M in anhydrous THF, 1.5 eq) added dropwise at room temperature and the reaction monitored by TLC. After complete conversion (ca. 3 h) the reaction was quenched with sat. NaHCO_3_ solution (equal volume) and diluted with EtOAc. The phases were separated, the aqueous phase extracted with EtOAc (3× equal volume) and the combined organic phases washed with sat. NaHCO_3_ (2× equal volume) and brine (equal volume). The organic phase was dried over MgSO_4_, filtered and the solvent removed under reduced pressure to afford the crude product, which was purified by silica column chromatography as specified.

### 3.2. Representative Synthesis and Characterization of Compound 29

**(2*R*,3*S*)-2-(Dimethylamino)-3-(methoxy(methyl)carbamoyl)pent-4-enoic acid, benzyl ester** Following the general procedure, PNP ester **13** (78.2 mg, 0.30 mmol, 1.0 eq), Pd_2_dba_3_·CHCl_3_ (7.7 mg, 7.5 µmol, 2.5 mol%), P(2-furyl)_3_ (6.9 mg, 30 µmol, 10 mol%), (*S*)-TM·HCl (14.4 mg, 0.06 mmol, 20 mol%), phosphate **26** (105 mg, 0.37 mmol, 1.25 eq) and *i*Pr_2_NEt (125 µL, 0.7 mmol, 2.4 eq) in MeCN (5.0 mL) gave the crude product, which was used directly for derivatization with NaOBn (0.45 mL, 0.45 mmol, 1.5 eq) in THF (6.0 mL). Subsequent purification of the crude derivatized product via silica column chromatography (petrol:EtOAc 4:1 to EtOAc) gave:

**(2*R*,3*R*)-29_min_** (a*nti-***29**) (R_f_ 0.33 in petrol: EtOAc 1:1) as colorless glass (21 mg, 22%). ${\left[\alpha \right]}_{D}^{20}$ + 8.5 (*c* 1.4 in CHCl_3_); chiral HPLC analysis Chiralcel OD-H (99:1 hexane: *i*PrOH, flow rate 1 mL min^−1^, 211 nm, 40 °C) t_R,1_: 16.3 min, t_R,2_: 19.0 min, 77:23 er; ^1^H-NMR (500 MHz, CDCl_3_) δ_H_: 2.33 (6H, s, N(C*H*_3_)_2_), 3.12 (3H, s, C(O)NC*H*_3_), 3.69 (3H, s, OC*H*_3_), 3.83 (1H, d, *J* 11.3, C(2)*H*), 3.98–4.07 (1H, m, C(3)*H*), 5.05 (1H, d, *J* 12.3, OC*H^A^*H^B^), 5.20–5.26 (3H, m, OCH^A^*H^B^*, C(5)*H*_2_), 5.82 (1H, ddd, *J* 17.0, 10.5, 8.4, C(4)*H*), 7.28–7.38 (5H, m, 5 × Ar*H*); ^13^C{^1^H} NMR (126 MHz, CDCl_3_) δ_C_: 32.1 (C(O)N*C*H_3_), 41.9 (N(*C*H_3_)_2_), 45.6 (*C*(3)H), 61.0 (O*C*H_3_), 66.1 (O*C*H_2_), 68.0 (*C*(2)H), 118.2 (*C*(5)H_2_), 128.2 (Ar*C*H), 128.4 (Ar*C*H), 128.5 (Ar*C*H), 134.1 (*C*(4)H), 135.9 (Ar*C*), 170.5 (C=O_ester_), 172.4 (C=O_amide_); HRMS (ESI^+^) C_17_H_24_N_2_O_4_Na [M+Na]^+^ found 343.1616, requires 343.1628 (−3.6 ppm); **ν_max_** (CHCl_3_, cm^−1^) 3016 (=CH), 2943 (C-H), 2789 (N(C-H)), 1720 (C=O_ester_), 1651 (C=O_amide_), 1631 (C=C).

**(2*R,*3*S*)-29_maj_***(syn-***29)** (R_f_ 0.12 in petrol:EtOAc 1:1) as yellow oil (33 mg, 34%). ${\left[\alpha \right]}_{D}^{20}$ − 1.6 (*c* 0.9 in CHCl_3_); chiral HPLC analysis Chiralcel OD-H (95:5 hexane:*i*PrOH, flow rate 1 mlmin^−1^, 211 nm, 40 °C) t_R,1_: 8.3 min, t_R,2_: 9.8 min, 87:13 er; ^1^H-NMR (500 MHz, CDCl_3_) δ_H_: 2.31 (6H, s, N(C*H*_3_)_2_), 3.20 (3H, s, C(O)NC*H*_3_), 3.70 (3H, s, OCH_3_), 3.80 (1H, d, *J* 11.4, C(2)*H*), 4.10–4.19 (1H, m, C(3)*H*), 5.10 (1H, dd, *J* 10.1, 1.3, C(5)*H^A^*H^B^), 5.10 (1H, d, *J* 12.2, OC*H^A^*H^B^), 5.14 (1H, d, *J* 12.2, OCH^A^*H^B^*), 5.21 (1H, dt, *J* 17.2, 0.8, C(5)H^A^*H^B^*), 5.76 (1H, ddd, *J* 17.2, 10.2, 8.8, C(4)*H*), 7.29–7.37 (5H, m, 5 × Ar*H*); ^13^C{^1^H} NMR (126 MHz, CDCl_3_) δ_C_: 32.2 (C(O)N*C*H_3_), 42.0 (N(*C*H_3_)_2_), 45.9 (*C*(3)H), 61.7 (O*C*H_3_), 65.8 (O*C*H_2_), 68.9 (*C*(2)H), 119.9 (*C*(5)H_2_), 128.2 (Ar*C*H), 128.5 (Ar*C*H), 128.5 (Ar*C*H), 133.3 (*C*(4)H), 135.8 (Ar*C*), 169.4 (C=O_ester_), 172.1 (C=O_amide_); HRMS (ESI^+^) C_17_H_25_N_2_O_4_ [M + H]^+^ found 321.1805 requires 321.1809 (−1.2 ppm); **ν_max_** (CHCl_3_, cm^−1^) 3008 (=CH), 2943 (C-H), 2792 (N(C-H)), 1724 (C=O_ester_), 1654 (C=O_amide_), 1635 (C=C).

## 4. Conclusions

In summary, in this manuscript, the compatibility of different functional groups within the previously developed *N*-allylation/[2,3]-rearrangement relay catalysis has been probed. In particular, electron-withdrawing ester and amide groups showed good reactivity, leading to functionalized α-amino acid derivatives, featuring a 1,4-dicarbonyl motif. The observed diastereo- and enantioselectivities were reduced compared to the use of aryl-containing allylic fragments in this protocol, but still led to the formation of desirable α-amino acid derivatives. Future work from this laboratory will probe further enantioselective reaction processes using isothioureas and alternative Lewis bases in dual catalytic protocols.

## Figures and Tables

**Table 1:**
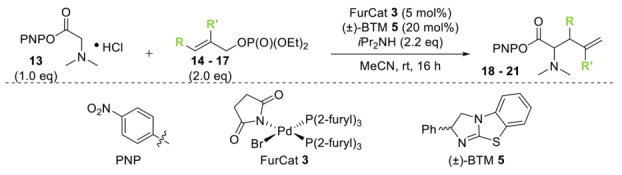
Initial functional group assessment in palladium/isothiourea relay catalysis.

Entry ^a^	Allyl Precursor	Product	Yield (%) ^b^	dr ^c^
1	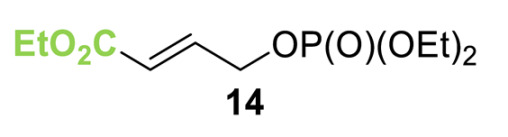	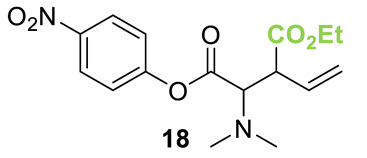	70 (61)	60:40
2	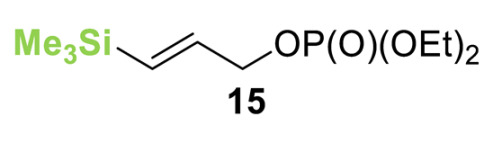	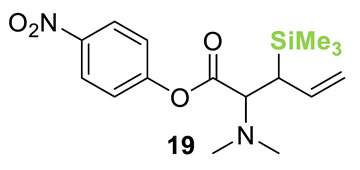	-	-
3	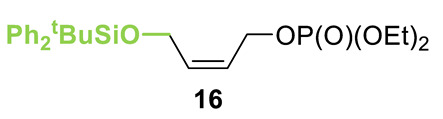	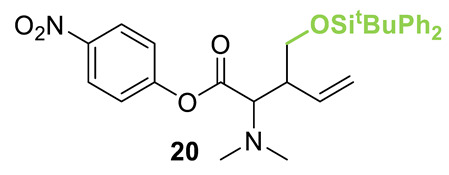	33 (30)	55:45
4	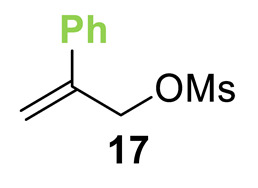	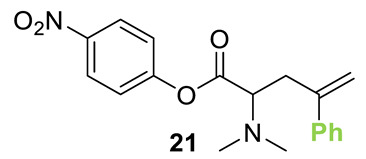	32 (-)	-

^a^ Reactions performed on a 0.25-mmol scale. ^b^ Combined yield of diastereoisomers determined by ^1^H-NMR analysis using 1,4-dinitrobenzene as internal standard. Values in parenthesis are isolated yields. ^c^ Determined by ^1^H-NMR of the crude material.

**Table 2:**
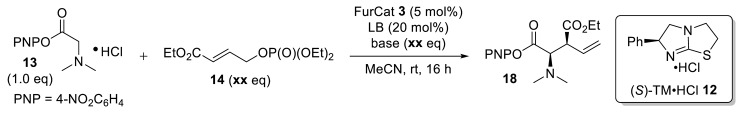
Optimization of the relay process for ethyl-ester-containing allylic phosphate.

Entry ^a^	LB catalyst	Base	Phosphate	Yield (%) ^b^	dr ^c^
1	(±)-BTM	*i*Pr_2_NH (2.2 eq)	2.0 eq	70	60:40
2	(±)-BTM	*i*Pr_2_NEt (2.2 eq)	2.0 eq	65 (17)	56:44
3	(±)-TM·HCl	*i*Pr_2_NEt (2.4 eq)	2.0 eq	87 (56)	67:33
4	(±)-TM·HCl	*i*Pr_2_NEt (2.4 eq)	1.25 eq	(62)	55:45
5	(*S*)-TM·HCl	*i*Pr_2_NEt (2.4 eq)	1.25 eq	(70)	60:40 ^d^

^a^ Reactions performed on a 0.25-mmol scale. ^b^ Combined yield of diastereoisomers determined by ^1^H-NMR analysis using 1,4-dinitrobenzene as internal standard. Values in parentheses are isolated yields. ^c^ Determined by ^1^H-NMR of the crude material. ^d^ 90:10 er_maj_, 70:30 er_min_; enantiomeric ratios determined by chiral stationary phase HPLC analysis.

## Supplementary Materials

The following are available online. Full experimental procedures, characterization data, NMR spectra and HPLC chromatograms for all new compounds, as well as crystallographic data for rearrangement products **28_maj_** (CCDC 2000698), **(*E*) -37** (CCDC 2000699) and **40_min_** (CCDC 2000700) are available online. The research data underpinning this publication can be found at DOI: https://doi.org/10.17630/1991da1b-e4f2-4a3c-8364-271023c723e2.
