# Supplementary material for: Exploring the Scope of Tandem Palladium and Isothiourea Relay Catalysis for the Synthesis of α-Amino Acid Derivatives
Source: Molecules. 2020 May 25;25(10):2463. doi: 10.3390/molecules25102463 (PMC7287902; doi:10.3390/molecules25102463)
Supplement: Supplementary file 1 [file molecules-25-02463-s001.pdf]

Exploring the Scope of Tandem Palladium and Isothiourea Relay Catalysis for the Synthesis of α-Amino Acid Derivatives

Jacqueline Bitai, Alexandra M. Z. Slawin, David B. Cordes and Andrew D. Smith*

Supporting Information

1. General Information.. 2

1. General Procedures 5

2. Preparation of Allylic Phosphate Starting Materials 8

3. Tandem Pd/ITU Relay Catalysis Products 26

4. Identified Side Products 43

5. Determination of Product Configuration 45

6. References... 49

Appendix I. Proposed Mechanisms for the Formation of Side Products...................... 52

Appendix II. ^{1}H, $^{13}C\{^{1}H\}$ and $^{31}P\{^{1}H\}$ NMR Spectra of Novel Compounds................... 54

Appendix III. HPLC traces.. 132

1. General Information

All reagents and solvents were obtained from commercial suppliers and were used without further purification unless otherwise stated. Purification was carried out according to standard laboratory methods. Tetramisole·HCl **12** was obtained from Sigma-Aldrich, benzotetramisole (BTM) **5** was synthesised in house.[1] *N*,*N*-Dimethylglycine, 4-nitrophenyl ester hydrochloride **13**, 2-(piperidin-1-yl)acetic acid, 4-nitrophenyl ester hydrochloride **30** and *N*-allyl-*N*-methylglycine, 4-nitrophenol ester hydrochloride **31** were prepared according to literature procedures.[2] Palladium precatalyst FurCat **3** was prepared according to the literature.[3] $Pd_2dba_3·CHCl_3$ was purchased from Strem Chemicals Inc. and recrystallized from $CHCl_3$/Acetone following the procedure of Ananikov and co-workers.[4] Racemic products were obtained using (±)-TM·HCl catalyst. Sodium benzylate (NaOBn, 1 M in THF) was prepared by treating BnOH with NaH in anhydrous THF.

Reactions involving moisture sensitive reagents were carried out in flame-dried glassware under an inert atmosphere (N_2 or Ar) using standard vacuum line techniques. Anhydrous solvents (Et_2O, CH_2Cl_2, THF and toluene) were obtained after passing through an alumina column (Mbraun SPS-800). Petrol is defined as petroleum ether 40–60 °C.
Room temperature (rt) refers to 20–25 °C. Temperatures of 0 °C and –78 °C were obtained using ice/water and CO_2(s)/acetone baths, respectively. Reactions involving heating were performed using DrySyn blocks and a contact thermocouple.

Under reduced pressure refers to the use of either a Büchi Rotavapor R-200 with a Büchi V-491 heating bath and Büchi V-800 vacuum controller, a Büchi Rotavapor R-210 with a Büchi V-491 heating bath and Büchi V-850 vacuum controller, a Heidolph Laborota 4001 with vacuum controller, an IKA RV10 rotary evaporator with an IKA HB10 heating bath and ILMVAC vacuum controller, or an IKA RV10 rotary evaporator with an IKA HB10 heating bath and Vacuubrand CVC3000 vacuum controller. Rotary evaporator condensers are fitted to Julabo FL601 Recirculating Coolers filled with ethylene glycol and set to –5 °C.

Analytical thin layer chromatography was performed on pre-coated aluminium plates (Kieselgel 60 F254 silica) and visualisation was achieved using ultraviolet light (254 nm)

and/or staining with either aqueous $KMnO_4$ solution, ethanolic phosphomolybdic acid, or ethanolic Vanillin solution followed by heating. Manual column chromatography was performed in glass columns fitted with porosity 3 sintered discs over Kieselgel 60 silica using the solvent system stated. Automated chromatography was performed on a Biotage Isolera Four running Biotage OS578 with a UV/Vis detector using the method stated and cartridges filled with Kieselgel 60 silica.

Melting points were recorded on an Electrothermal 9100 melting point apparatus, (dec) refers to decomposition.
Optical rotations $[\alpha]_D^{20}$ were measured on a Perkin Elmer Precisely/Model-341 polarimeter operating at the sodium D line with a 100 mm path cell at 20 °C.

HPLC analyses were obtained on either a Shimadzu HPLC consisting of a DGU-20A5 degassing unit, LC-20AT liquid chromatography pump, SIL-20AHT autosampler, CMB-20A communications bus module, SPD-M20A diode array detector and a CTO-20A column oven or a Shimadzu HPLC consisting of a DGU-20A5R degassing unit, LC-20AD liquid chromatography pump, SIL-20AHT autosampler, SPD-20A UV/Vis detector and a CTO-20A column oven. Separation was achieved using either DAICEL CHIRALCEL OD-H column or DAICEL CHIRALPAK AD-H, and ID columns using the method stated. HPLC traces of enantiomerically enriched compounds were compared with authentic racemic spectra.

Infrared spectra were recorded on a Shimadzu IRAffinity-1 Fourier transform IR spectrophotometer fitted with a Specac Quest ATR accessory (diamond puck). Spectra were recorded of either thin films or solids, with characteristic absorption wavenumbers (ν_{max}) reported in cm^{-1}.

^{1}H, $^{13}C\{^{1}H\}$, and $^{31}P\{^{1}H\}$ NMR spectra were acquired on either a Bruker AV300 with a BBFO probe (^{1}H 300 MHz), a Bruker AV400 with a BBFO probe (^{1}H 400 MHz; $^{13}C\{^{1}H\}$ 101 MHz; $^{31}P\{^{1}H\}$ 162 MHz), a Bruker AVII 400 with a BBFO probe (^{1}H 400 MHz; $^{13}C\{^{1}H\}$ 101 MHz; $^{31}P\{^{1}H\}$ 162 MHz), a Bruker AVIII-HD 500 with a SmartProbe BBFO+ probe (^{1}H 500 MHz, $^{13}C\{^{1}H\}$ 126 MHz), a Bruker AVIII 500 with a CryoProbe Prodigy BBO probe (^{1}H 500 MHz, $^{13}C\{^{1}H\}$ 126 MHz) in the deuterated solvent stated. All chemical shifts are quoted in parts per

million (ppm) relative to the residual solvent peak. All coupling constants, J, are quoted in Hz. Multiplicities are indicated as s (singlet), br s (broad singlet), d (doublet), t (triplet), q (quartet), m (multiplet), and multiplets thereof. The abbreviation Ar denotes aromatic. NMR peak assignments were confirmed using 2D ^{1}H correlated spectroscopy (COSY), 2D ^{1}H–^{13}C heteronuclear multiple-bond correlation spectroscopy (HMBC), and 2D ^{1}H–^{13}C heteronuclear single quantum coherence (HSQC) where necessary.

Mass spectrometry (*m*/*z*) data were acquired by either electrospray ionisation (ESI) or nanospray ionisation (NSI) at either the University of St Andrews Mass Spectrometry Facility or at the EPSRC UK National Mass Spectrometry Facility at Swansea University.

1. General Procedures

1.1 General Procedure for the Synthesis of Allylic Phosphates

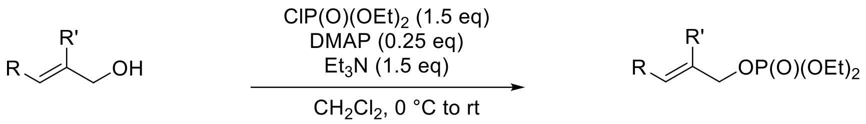

Following the procedure of Smith and co-workers[2], allylic alcohol (1.0 eq) was added to a flame dried round bottom flask and dissolved in anhydrous CH_2Cl_2 (0.1 M) under argon atmosphere. Et_3N (1.5 eq) and DMAP (0.25 eq) were added, the mixture was cooled to 0 °C and diethyl chlorophosphate (1.5 eq) was added dropwise. Stirring was continued at rt until TLC analysis indicated complete conversion. The reaction mixture was quenched with sat. aq. $NaHCO_3$ (equal volume), the phases were separated, and the aqueous phase was extracted with CH_2Cl_2 (3 × equal volume). The combined organic phases were dried over $MgSO_4$, filtered and the solvent was removed under reduced pressure. The crude residue was purified by flash silica chromatography as specified.

General Scheme for the Synthesis of Amide containing Allylic Alcohols

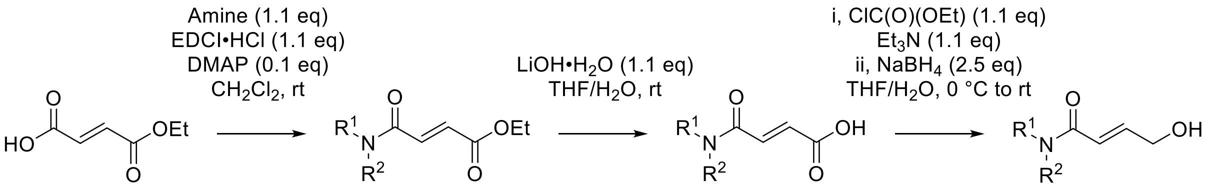

1.2 General Procedure for Amide Coupling

Adapting the procedure of Snaddon and co-workers,[5] monoethyl fumarate (1.0 eq) and amine (1.1 eq) were dissolved in CH_2Cl_2 (0.4 M), followed by the addition of EDCI·HCl (1.1 eq) and DMAP (0.1 eq) at 0 °C. The reaction mixture was allowed to warm to room temperature overnight and subsequently washed with 1 M HCl (2 × equal volume) and brine (2 × equal volume). The organic phase was dried over $MgSO_4$, filtered and the solvent removed under reduced pressure to yield the corresponding amide ester, which was used without further purification.

1.3 General Procedure for Ester Hydrolysis with LiOH

Adapting the procedure of Smith and co-workers,[6] $LiOH·H_2O$ (1.1 eq) was added in portions to a stirred solution of ethyl ester (1.0 eq) in H_2O : THF 1:1 (1.0 M). The reaction was stirred overnight at room temperature followed by acidification with 2 M HCl to pH 2 and extraction with CH_2Cl_2 (3 ×). The combined organic phases were dried over $MgSO_4$ and the solvent removed under reduced pressure to yield the corresponding acid.

1.4 General Procedure for the Reduction of unsaturated Acids

Adapting the procedure of Jacobi and co-workers,[7] to a stirred solution of unsaturated acid (1.0 eq) and Et_3N (1.1 eq) in anhydrous THF (0.4 M) under inert atmosphere at 0 °C was added dropwise ethyl chloroformate (1.1 eq). The resulting suspension was stirred at 0 °C for 1 h, filtered and the solid washed twice with anhydrous THF. The combined filtrates were added dropwise to a vigorously stirred solution of $NaBH_4$ (2.5 eq) in H_2O (0.7 M) at 0 °C. The reaction mixture was allowed to warm to room temperature and stirred until TLC indicated complete conversion. The mixture was adjusted to pH 5 with 1 M HCl, extracted with EtOAc (4 ×) and the combined organic phases dried over $MgSO_4$, filtered and the solvent removed under reduced pressure. The crude product was purified by silica column chromatography as specified.

1.5 General Procedure for Tandem Pd/ITU Relay Catalysis

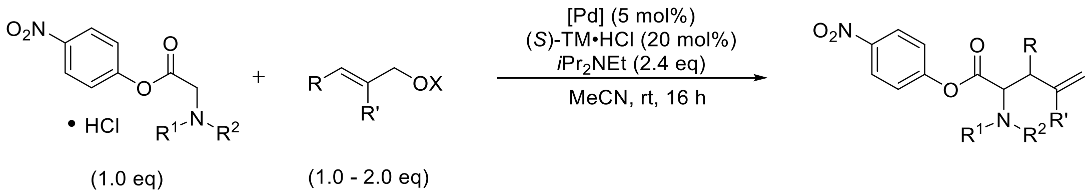

A Schlenk tube was charged with PdFurCat (5 mol%) or $Pd_2(dba)_3·CHCl_3$ (2.5 mol%) and $P(2\text{-furyl})_3$ (10 mol%), (*S*)-TM·HCl (10 mol%) and PNP Ester (1.0 eq). The tube was then evacuated and flushed with argon three times, degassed MeCN (0.06 M) was added and the mixture stirred for 10 min at room temperature. Subsequently phosphate or mesylate (1.0 – 2.0 eq) and $i\text{Pr}_2\text{NEt}$ (2.4 eq) were added in this order and the reaction mixture stirred at room temperature for 16 h. An aliquot was taken, the solvent removed under reduced pressure and ^{1}H NMR spectroscopy of the crude mixture used to determine the dr. The reaction

mixture was then filtered over a short plug of silica with MeCN and the filtrate concentrated under reduced pressure. The residue was purified by flash silica chromatography as specified or directly derivatised with NaOBn.

Derivatisation with NaOBn:

The crude reaction mixture was dissolved in anhydrous THF (6.0 mL), freshly prepared NaOBn (1 M in anhydrous THF, 0.45 mL, 0.45 mmol, 1.5 eq) added dropwise at room temperature and the reaction monitored by TLC. After complete conversion (ca. 3h) the reaction was quenched with sat. $NaHCO_3$ solution (equal volume) and diluted with EtOAc. The phases were separated, the aqueous phase extracted with EtOAc (3 × equal volume) and the combined organic phases washed with sat. $NaHCO_3$ (2 × equal volume) and brine (equal volume). The organic phase was dried over $MgSO_4$, filtered and the solvent removed under reduced pressure to afford the crude product, which was purified by silica column chromatography as specified.

2. Preparation of Allylic Phosphate Starting Materials

2.1 (*E*)-4-Hydroxy-2-butenoic acid, ethyl ester (SI-1)

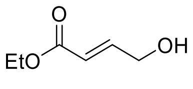

$C_6H_{10}O_3$
130.14
SI-1

Following the procedure of Smith and co-workers,[8] borane (1.0 M in THF, 14 mL, 14 mmol, 1.0 eq) was added dropwise to a stirred solution of monoethyl fumarate (2.02 g, 14 mmol, 1.0 eq) in anhydrous THF (10 mL) at 0 °C. After complete addition, the reaction mixture was allowed to warm to rt and stir for 12 h. The reaction was quenched with 50% aq. AcOH (10 mL) and then THF was removed under reduced pressure. The remaining solution was neutralized with sat. aq. $NaHCO_3$ solution and extracted with EtOAc (4 × 30 mL). The combined organic phases were dried over $MgSO_4$, filtered and the solvent removed under reduced pressure. Purification by silica column chromatography (Petrol : EtOAc 4:1 to 1:1, R_f 0.47 in Petrol : EtOAc 1:1) gave the title compound as a pale yellow liquid (905 mg, 49%).

^{1}H NMR (400 MHz, $CDCl_3$) δ_H: 1.28 (3H, t, *J* 7.1, OCH_2CH_3), 4.19 (2H, q, *J* 7.1, OCH_2CH_3), 4.34 (2H, dd, *J* 4.0, 2.1, C(4)H_2), 6.09 (1H, dt, *J* 15.7, 2.1, C(2)*H*), 7.02 (1H, dt, *J* 15.7, 4.0, C(3)*H*).

Data in accordance with literature.[9]

2.2 (*E*)-4-[(Diethoxyphosphinyl)oxy]-2-butenoic acid, ethyl ester (14)

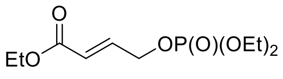

$C_{10}H_{19}O_6P$
266.23
14

Following General Procedure 1.1 using **SI-1** (195 mg, 1.5 mmol, 1.0 eq), diethyl chlorophosphate (325 μL, 2.25 mmol, 1.5 eq), Et_3N (314 μL, 2.25 mmol, 1.5 eq) and DMAP (46.0 mg, 0.38 mmol, 0.25 eq) in anhydrous CH_2Cl_2 (10 mL) gave the title compound following purification by flash silica chromatography (*n*-hexane : EtOAc 1:1 to 1:2, R_f 0.19 in *n*-hexane : EtOAc 1:1) as a pale yellow oil (283 mg, 71%).

^{1}H NMR (400 MHz, $CDCl_3$) δ_H: 1.28 (3H, t, *J* 7.1, CH_2CH_3), 1.34 (6H, td, *J* 7.0, 1.0, $P(OCH_2CH_3)_2$), 4.13 (4H, dq, *J* 8.1, 7.1, $P(OCH_2CH_3)_2$), 4.20 (2H, q, *J* 7.2, CH_2CH_3), 4.69 (2H, ddd, *J* 7.3, 4.2, 2.1, C(4)H_2), 6.11 (1H, dt, *J* 15.6, 2.0, C(2)*H*), 6.92 (1H, dtd, *J* 15.7, 4.3, 1.8, C(3)*H*).

^{13}C {^{1}H} NMR (126 MHz, $CDCl_3$) δ_C: 14.2 (CH_2CH_3), 16.1 (d, $^3J_{CP}$ 6.7, $P(OCH_2CH_3)_2$), 60.6 (CH_2CH_3), 64.1 (d, $^2J_{CP}$ 5.9, $P(OCH_2CH_3)_2$), 65.3 (d, $^2J_{CP}$ 4.9, *C*(4)H_2), 122.0 (C(2)H), 141.2 (d, $^3J_{CP}$ 7.7, *C*(3)H), 165.8 (C=O).

^{31}**P {^{1}H} NMR** (162 MHz, $CDCl_3$) δ_P: −1.09 (*P*(O)(OEt)$_2$).

HRMS (NSI$^+$) $C_{10}H_{20}O_6P$ [M+H]$^+$ found 267.0992, requires 267.0992 (± 0.0 ppm).

ν_{max} (film, cm^{-1}) 2983 (C-H), 1718 (C=O), 1666 (C=C), 1263 (P=O), 1018 (P-OEt).

2.3 3-(Trimethylsilyl)prop-2-yn-1-ol (SI-2)

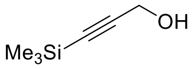

$C_6H_{12}OSi$
128.25
SI-2

Adapting the procedure of Hoveyda and co-workers[10], in a flame dried two-necked flask under inert atmosphere, propargylic alcohol (0.26 mL, 4.46 mmol, 1.0 eq) was dissolved in anhydrous THF (30 mL). The solution was cooled to −78 °C before *n*-BuLi (2.5 M in *n*-hexane, 3.8 mL, 9.37 mmol, 2.1 eq) was added slowly. After stirring the mixture for 45 min at −78 °C trimethylsilylchloride (1.24 mL, 9.81 mmol, 2.2 eq) was added dropwise. The reaction mixture was allowed to warm to rt over 3 h. The reaction was quenched by the addition of H_2O (5 mL) and 1 M HCl (15 mL) and stirred at rt until TLC (*n*-hexane : EtOAc 9:1) indicated complete conversion of the intermediate TMS protected alcohol. The phases were separated, and the aqueous phase extracted with Et_2O (3 × 15 mL). The combined organic phases were washed with brine (40 mL), dried over $MgSO_4$, filtered and the solvent was removed under reduced pressure to afford the title compound as a yellow oil (529 mg, 92%).

^{1}**H NMR** (400 MHz, $CDCl_3$) δ_H: 0.19 (9H, s, Si-(C*H*$_3$)$_3$), 1.98 (1H, br s, CH_2-O*H*), 4.28 (2H, s, C*H*$_2$-OH).

Data in accordance with literature.[11]

2.4 (*E*)-3-(Trimethylsilyl)prop-2-en-1-ol (SI-3)

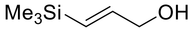

$C_6H_{14}OSi$
130.26
SI-3

Following the procedure of Hoveyda and co-workers[10], in a flame dried flask, compound **SI-2** (250 mg, 1.95 mmol, 1.0 eq) was dissolved in anhydrous Et_2O (5 mL) under an inert atmosphere and cooled to 0 °C. Red-Al® (65 wt% in toluene, 1.22 mL, 3.9 mmol, 2.0 eq) was diluted with anhydrous Et_2O (equal volume) and added slowly. After complete addition, the reaction mixture was allowed to warm to rt and stir until TLC (Petrol : EtOAc 6:1) indicated complete conversion. The reaction mixture was quenched with H_2O (1 mL) and H_2SO_4 (3.6 M,

2 mL) and diluted by the addition of H_2O (10 mL) and Et_2O (10 mL). The phases were separated, and the aqueous phase extracted with Et_2O (3 × 10 mL). The combined organic phases were washed with brine (15 mL), dried over $MgSO_4$, filtered and the solvent removed under reduced pressure to afford the title compound as a colourless oil (253 mg, 99%).

^{1}H NMR (400 MHz, $CDCl_3$) δ_H: 0.08 (9H, s, Si-(C*H*$_3$)$_3$), 4.18 (2H, dd, *J* 4.4, 1.8, C(1)*H*$_2$), 5.92 (1H, dt, *J* 18.8, 1.7, C(3)*H*), 6.19 (1H, dt, *J* 18.8, 4.4, C(2)*H*).

Data in accordance with literature.[12]

2.5 (*E*)-3-(Trimethylsilyl)prop-2-en-1-yl-phosphoric acid, diethyl ester (15)

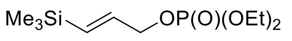

$C_{10}H_{23}O_4PSi$
266.35
15

The title compound was prepared following General Procedure 1.1 using **SI-3** (195 mg, 1.5 mmol, 1.0 eq), diethyl chlorophosphate (0.32 mL, 2.25 mmol, 1.5 eq), Et_3N (0.31 mL, 2.25 mmol, 1.5 eq) and DMAP (46.0 mg, 0.38 mmol, 0.25 eq) in anhydrous CH_2Cl_2 (15 mL). The crude mixture was purified by silica column chromatography (*n*-hexane/EtOAc 1:1) to yield **15** as a colourless oil (366 mg, 91%).

^{1}H NMR (400 MHz, $CDCl_3$) δ_H: 0.10 (9H, s, Si-(C*H*$_3$)$_3$), 1.36 (6H, td, *J* 7.0, 1.0, P(OCH$_2$C*H*$_3$)$_2$), 4.14 (4H, dq, *J* 7.9, 7.1, P(OC*H*$_2$CH$_3$)$_2$), 4.56 (2H, ddd, *J* 7.9, 4.4, 1.1, C(1)*H*$_2$), 6.02 (1H, dt, *J* 18.7, 1.1, C(3)*H*), 6.10 (1H, dtd, *J* 18.6, 4.4, 0.7, C(2)*H*).

^{31}P {^{1}H} NMR (162 MHz, $CDCl_3$) δ_P: −0.80 (*P*(O)(OEt)$_2$).

Data in accordance with literature.[13]

2.6 (*Z*)-4-[[*tert*-Butyldiphenylsilyl]oxy]-2-buten-1-ol (SI-4)

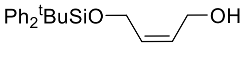

$C_{20}H_{26}O_2Si$
326.51
SI-4

Following the procedure of Takahata and co-workers,[14] in a flame dried round bottom flask under an inert atmosphere NaH (60 wt% suspension in oil, 240 mg, 6.0 mmol, 1.2 eq) was washed twice with *n*-hexane, dried under reduced pressure and subsequently suspended in anhydrous THF (10 mL). *cis*-Butene-1,4-diol (0.41 mL, 5.0 mmol, 1.0 eq) was added slowly and the mixture stirred for 1 h. *tert*-Butyldiphenylsilylchloride (1.43 mL, 5.5 mmol, 1.1 eq) was added at 0 °C and the reaction allowed to stir at rt until TLC (EtOAc) indicated complete

conversion. The mixture was quenched with sat. K_2CO_3 solution (10 mL), diluted with Et_2O (5 mL) and the phases separated. The aqueous phase was extracted with Et_2O (3 × 10 mL), the combined organic phases were washed with brine, dried over $MgSO_4$, filtered and the solvent removed under reduced pressure. The crude product was purified by silica column chromatography (Petrol/EtOAc 5:1 to 2:1, R_f 0.26 in Petrol : EtOAc 4:1) to yield the title compound as colourless liquid (1.53 g, 94%).

^{1}H NMR (400 MHz, $CDCl_3$) δ_H: 1.11 (9H, s, C(C*H*$_3$)$_3$), 1.86 (1H, br s, O*H*), 4.02 – 4.09 (2H, m, C*H*$_2$-OSi), 4.29 – 4.34 (2H, m, C*H*$_2$-OH), 5.63 – 5.80 (2H, m, C*H*=C*H*), 7.41 – 7.49 (6H, m, 2 × Ar(2,4,6)*H*), 7.71 – 7.78 (4H, m, 2 × Ar(3,5)*H*).

Data in accordance with literature.[14]

2.7 (*Z*)-4-[[(1,1-Dimethylethyl)diphenylsilyl]oxy]-2-buten-1-yl-phosphoric acid, diethyl ester (16)

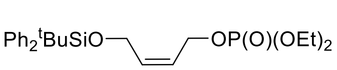

$C_{24}H_{35}O_5PSi$
462.60
16

Following General Procedure 1.1, compound **SI-4** (1.00 g, 3.06 mmol, 1.0 eq), diethyl chlorophosphate (0.66 mL, 4.6 mmol, 1.5 eq), Et_3N (0.64 mL, 4.6 mmol, 1.5 eq) and DMAP (93.0 mg, 0.7 mmol, 0.25 eq) in anhydrous CH_2Cl_2 (30 mL) gave the title compound after purification by silica column chromatography (*n*-hexane : EtOAc 3:1 to 1:1, R_f 0.33 in *n*-hexane : EtOAc 1:1) as colourless liquid (1.01 g, 73%).

^{1}H NMR (500 MHz, $CDCl_3$) δ_H: 1.07 (9H, s, C(C*H*$_3$)$_3$), 1.31 (6H, td, *J* 7.1, 1.0, P(OCH$_2$C*H*$_3$)$_2$), 4.08 (4H, dqd, *J* 8.5, 7.1, 1.5, P(OC*H*$_2$CH$_3$)$_2$), 4.30 (2H, ddt, *J* 5.9, 1.8, 0.9, C(4)*H*$_2$), 4.52 (2H, ddt, *J* 8.9, 6.4, 1.2, C(1)*H*$_2$), 5.64 (1H, dtt, *J* 11.2, 6.5, 1.7, C(2)*H*), 5.81 (1H, dtt, *J* 11.5, 5.8, 1.5, C(3)*H*), 7.39 – 7.46 (6H, m, 2 × Ar(2,4,6)*H*), 7.67 – 7.72 (4H, m, 2 × Ar(3,5)*H*).

^{13}C{^{1}H} NMR (126 MHz, $CDCl_3$) δ_C: 16.1 (d, $^3J_{CP}$ 6.7, P(OCH$_2$CH$_3$)$_2$), 19.1 (*C*(CH$_3$)$_3$), 26.7 (C(*C*H$_3$)$_3$), 60.3 (*C*(4)H$_2$), 63.2 (d, $^2J_{CP}$ 5.5, *C*(1)H$_2$), 63.7 (d, $^2J_{CP}$ 5.8, P(OCH$_2$CH$_3$)$_2$), 125.1 (d, $^3J_{CP}$ 7.2, *C*(2)H), 127.7 (2 × Ar*C*(2,6)H), 129.8 (2 × Ar*C*(4)H), 133.1 (*C*(3)H), 133.3 (2 × Ar*C*(1)), 135.5 (2 × Ar*C*(3,5)H).

^{31}P {^{1}H} NMR (162 MHz, $CDCl_3$) δ_P: −0.84 (*P*(O)(OEt)$_2$).

HRMS (NSI$^+$) $C_{24}H_{36}O_5PSi$ [M+H]$^+$ found 463.2057, requires 463.2064 (−1.5 ppm).

ν_{max} (film, cm^{-1}) 2931 (C-H), 2856 (C-H), 1427 (Si-Ph), 1263 (P=O), 1107 (Si-Ph), 1018 (P-OEt).

2.8 2-Phenylprop-2-en-1-ol (SI-5)

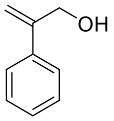

$C_9H_{10}O$
134.18
SI-5

Following the procedure from Snaddon and co-workers,[15] in a flame dried three necked flask Mg turnings (1.46 g, 60.0 mmol, 3.0 eq) were covered with anhydrous Et_2O under an inert atmosphere. Bromobenzene (5.32 mL, 50.0 mmol, 2.5 eq) was dissolved in 40 mL anhydrous Et_2O and added dropwise in the presence of a small amount of iodide to help start the reaction. The rate of addition was adjusted to keep a constant reflux. After complete addition the reaction mixture was heated at reflux for 1 h and subsequently allowed to cool to rt. CuI (571 mg, 3.0 mmol, 0.15 eq) was added, the mixture stirred for 30 min, then propargylic alcohol (1.16 mL, 20.0 mmol, 1.0 eq) in anhydrous Et_2O (10 mL) was added slowly, and after complete addition the reaction mixture heated at reflux for 24 h. The reaction was quenched with sat. NH_4Cl solution (25 mL) at 0 °C, allowed to warm to rt and stir until all solids had dissolved (usually o.n.). The phases were separated, the aqueous phase extracted with Et_2O (3 × 30 mL), the combined organic phases washed with brine, dried over $MgSO_4$, filtered and the solvent removed under reduced pressure. The crude product was purified by silica column chromatography (Petrol : EtOAc 6:1 to 4:1, R_f 0.26 in Petrol : EtOAc 4:1) to yield the title compound as a yellow liquid (1.81 g, 68%).

^{1}H NMR (400 MHz, $CDCl_3$) δ_H: 1.74 (1H, br s, O*H*), 4.57 (2H, s, C*H*$_2$-OH), 5.37 – 5.40 (1H, m, C=C*H*$_2$), 5.49 - 5.52 (1H, m, C=C*H*$_2$), 7.30 – 7.43 (3H, m, Ar(2,4,6)*H*), 7.45 – 7.51 (2H, m, Ar(3,5)*H*).

Data in accordance with literature.[15]

2.9 2-Phenyl-2-propen-1-yl-phosphoric acid, diethyl ester (SI-6)

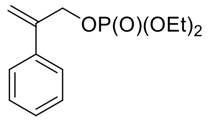

$C_{13}H_{19}O_4P$
270.26
SI-6

Following General Procedure 1.1, alcohol **SI-5** (1.00 g, 7.45 mmol, 1.0 eq), diethyl chlorophosphate (1.61 mL, 11.2 mmol, 1.5 eq), Et_3N (1.56 mL, 11.2 mmol, 1.5 eq) and DMAP (228 mg, 1.86 mmol, 0.25 eq) in anhydrous CH_2Cl_2 (50 mL) gave the title compound after

purification by silica column chromatography (*n*-hexane : EtOAc 1:1 to 1:3, R_f 0.24 in *n*-hexane : EtOAc 1:1) as a pale yellow liquid (1.75 g, 84%).

^{1}H NMR (400 MHz, $CDCl_3$) δ_H: 1.24 – 1.32 (6H, m, P(OCH$_2$C*H*$_3$)$_2$), 4.00 – 4.10 (4H, m, P(OC*H*$_2$CH$_3$)$_2$), 4.89 – 4.94 (2H, mz, C(1)*H*$_2$), 5.42 (1H, br s, C=C*H*$_2$), 5.55 (1H, br s, C=C*H*$_2$), 7.25 – 7.37 (3H, m, Ar(2,4,6)*H*), 7.40 – 7.47 (2H, m, Ar(3,5)*H*).

^{31}P {^{1}H} NMR (162 MHz, $CDCl_3$) δ_P: −1.09 (*P*(O)(OEt)$_2$).

Data in accordance with literature.[16]

2.10 2-Phenylallyl methanesulfonate (17)

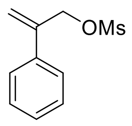

$C_{10}H_{12}O_3S$
212.26
17

Following the procedure of Snaddon and co-workers,[15] in a flame dried round bottom flask methanesulfonic anhydride (2.05 g, 11.8 mmol, 2.0 eq) was added to a stirred solution of alcohol **SI-5** (5.88 mmol, 790 mg, 1.0 eq) in anhydrous CH_2Cl_2 (15 mL). The mixture was cooled to 0 °C and *i*Pr_2NEt (2.05 mL, 11.8 mmol, 2.0 eq) added dropwise. The reaction mixture was allowed to warm to rt and stir until TLC (Petrol : EtOAc 4:1) indicated complete conversion. The solvent was removed under reduced pressure and the crude product purified by silica column chromatography (Petrol : Et_2O 5:1 to 2:1) to yield the title compound as a yellow liquid (748 mg, 60%).

^{1}H NMR (400 MHz, $CDCl_3$) δ_H: 2.97 (3H, s, C*H*$_3$), 5.16 (2H, d, *J* 1.1, C(1)*H*$_2$), 5.54 (1H, m, C=C*H*$_2$), 5.72 (1H, s, C=C*H*$_2$), 7.34 – 7.44 (3H, m, Ar(2,4,6)*H*), 7.46 – 7.52 (2H, m, Ar(3,5)*H*).

Data in accordance with literature.[15]

2.11 (*E*)-4-(Methoxymethylamino)-4-oxo-2-butenoic acid (SI-7)

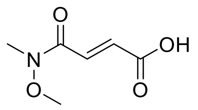

$C_6H_9NO_4$
159.14
SI-7

Following the procedure of Jacobi and co-workers,[17] a stirred solution of maleic anhydride (5.07 g, 51.7 mmol, 1.0 eq) and (*N*,*O*)-dimethylhydroxylamine hydrochloride (5.55 g, 56.8 mmol, 1.1 eq) in $CHCl_3$ (60 mL) was cooled to 0°C. Pyridine (9.2 mL, 113.7 mmol, 2.2 eq) was added slowly and the reaction allowed to warm to room temperature and stir for 24 h. The solvent was removed under reduced pressure, the residue diluted with H_2O (20 mL) and

brine (20 mL) and extracted with CH_2Cl_2 (4 × 30 mL). The combined extracts were washed with 1 M HCl (40 mL) and brine (40 mL), dried over $MgSO_4$, filtered and the solvent removed under reduced pressure. The crude product was recrystallised from CH_2Cl_2 to afford the title compound as yellow solid (2.74 g, 33%). **m.p.** (CH_2Cl_2) 129 – 131 °C.

^{1}H NMR (500 MHz, $CDCl_3$) δ_H: 3.31 (3H, s, NCH_3), 3.76 (3H, s, OCH_3), 6.91 (1H, d, *J* 15.6, C(2)*H*), 7.54 (1H, d, *J* 15.6, C(3)*H*).

^{13}C{^{1}H} NMR (126 MHz, $CDCl_3$) δ_C: 32.5 (NCH_3), 62.4 (OCH_3), 131.7 (*C*(2)H), 133.6 (*C*(3)H), 164.5 (*C*(4)=O), 170.0 (*C*(1)=O).

HRMS (NSI$^-$) $C_6H_8NO_4$ [M−H]$^-$ found 158.0461, requires 158.0459 (+1.4 ppm).

ν_{max} (film, cm^{-1}) 2943 (O-H), 1720 (C=O_{acid}), 1660 (C=O_{amide}), 1602 (C=C).

2.12 (*E*)-4-Hydroxy-*N*-methoxy-*N*-methyl-2-butenamide (SI-8)

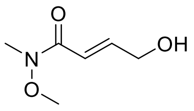

$C_6H_{11}NO_3$
145.16
SI-8

Following General Procedure 1.4, acid **SI-7** (3.0 g, 18.8 mmol, 1.0 eq), Et_3N (2.89 mL, 20.7 mmol, 1.1 eq) and ethylchloroformate (1.98 mL, 20.7 mmol, 1.1 eq) in anhydrous THF (50 mL) followed by $NaBH_4$ (1.78 g, 47.1 mmol, 2.5 eq) in H_2O (30 mL) gave the title compound after purification by silica column chromatography (EtOAc : Acetone 6:1, R_f 0.31) as pale yellow oil (1.22 g, 45%).

^{1}H NMR (400 MHz, $CDCl_3$) δ_H: 3.24 (3H, s, NCH_3), 3.70 (3H, s, OCH_3), 4.36 (2H, dd, *J* 4.0, 2.1, C(4)H_2), 6.67 (1H, br dt, *J* 15.5, C(2)*H*), 7.03 (1H, dt, *J* 15.5, 4.0, C(3)*H*).

^{13}C{^{1}H} NMR (126 MHz, $CDCl_3$) δ_C: 32.3 (NCH_3), 61.8 (OCH_3), 62.2 (*C*(4)H_2), 117.4 (*C*(2)H), 145.7 (*C*(3)H), 166.6 (*C*=O).

HRMS (NSI$^+$) $C_6H_{12}NO_3$ [M+H]$^+$ found 146.0809, requires 146.0812 (−1.8 ppm).

ν_{max} (film, cm^{-1}) 3390 (O-H), 2937 (C-H), 1660 (C=O), 1612 (C=C).

2.13 (*E*)-(4-(methoxy(methyl)amino)-4-oxobut-2-en-1-yl) phosphoric acid, diethyl ester (26)

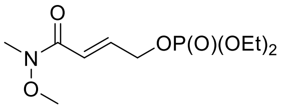

$C_{10}H_{20}NO_6P$
281.24
26

Following General Procedure 1.1, compound **29** (516 mg, 3.55 mmol, 1.0 eq), diethyl chlorophosphate (0.77 mL, 5.33 mmol, 1.5 eq), Et_3N (0.74 mL, 5.33 mmol, 1.5 eq) and DMAP (108 mg, 0.88 mmol, 0.25 eq) in anhydrous CH_2Cl_2 (35 mL) gave the title compound after purification by silica column chromatography (EtOAc : Acetone 4:1, R_f 0.33 in EtOAc : Acetone 2:1) as pale yellow oil (819 mg, 82 %).

^{1}H NMR (500 MHz, $CDCl_3$) δ_H: 1.36 (6H, td, *J* 7.1, 0.9, P(OCH$_2$C*H*$_3$)$_2$), 3.27 (3H, s, N-C*H*$_3$), 3.72 (3H, s, O-C*H*$_3$), 4.12 – 4.19 (4H, m, P(OC*H*$_2$CH$_3$)$_2$), 4.74 (2H, ddd, *J* 7.4, 4.3, 2.0, C(1)*H*$_2$), 6.68 – 6.76 (1H, m, C(3)*H*), 6.96 (1H, dtd, *J* 15.4, 4.3, 1.7, C(2)*H*).

^{13}C{^{1}H} NMR (126 MHz, $CDCl_3$) δ_C: 16.1 (d, $^3J_{CP}$ 6.6, P(OCH$_2$CH$_3$)$_2$), 32.3 (N-CH$_3$), 61.8 (O-CH$_3$), 64.0 (d, $^2J_{CP}$ 5.9, P(OCH$_2$CH$_3$)$_2$), 66.0 (d, $^2J_{CP}$ 5.2, *C*(1)H$_2$), 119.5 (*C*(3)H), 139.8 (d, $^3J_{CP}$ 7.4, *C*(2)H), 165.7 (*C*=O).

^{31}P{^{1}H} NMR (162 MHz, $CDCl_3$) δ_P: -1.07 (O*P*(O)(OEt)$_2$).

HRMS (NSI$^+$) $C_{10}H_{21}NO_6P$ [M+H]$^+$ found 282.1102, requires 282.1101 (+0.4 ppm).

ν_{max} ($CHCl_3$, cm^{-1}) 2983 (C-H), 2912 (C-H), 1670 (C=O), 1633 (C=C), 1261 (P=O), 1024 (P-O).

2.14 (*E*)-4-Oxo-4-(phenylamino)-2-butenoic acid, ethyl ester (SI-9)

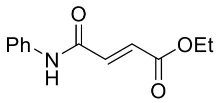

$C_{12}H_{13}NO_3$
219.24
SI-9

Following General Procedure 1.2 with modifications, monoethyl fumarate (6.0 g, 41.6 mmol, 1.0 eq), aniline (5.3 mL, 58.2 mmol, 1.4 eq), EDCI·HCl (10.3 g, 54.1 mmol, 1.3 eq) and DMAP (508 mg, 4.2 mmol, 0.1 eq) in CH_2Cl_2 (100 mL) gave the title compound after recrystallisation from Et_2O as off-white solid (7.4 g, 81 %). **m.p.** (Et_2O) 113-116 °C.

^{1}H NMR (500 MHz, $CDCl_3$) δ_H: 1.33 (3H, t, *J* 7.1, OCH$_2$C*H*$_3$), 4.27 (2H, q, *J* 7.1, OC*H*$_2$CH$_3$), 6.96 (1H, d, *J* 15.3, C(2)*H*), 7.12 – 7.20 (2H, m, C(3)*H*, ArC(4)*H*), 7.34 (2H, t, *J* 7.9, ArC(3,5)*H*), 7.62 (2H, d, *J* 7.7, ArC(2,6)*H*), 8.15 (1H, br s, N*H*).

^{13}**C{^{1}H} NMR** (126 MHz, $CDCl_3$) δ_C: 14.1 (OCH_2CH_3), 61.5 (OCH_2CH_3), 120.1 (Ar*C*(2,6)H), 125.0 (Ar*C*(4)H), 129.1 (Ar*C*(3,5)H), 131.2 (*C*(2)H), 137.0 (*C*(3)H), 137.4 (Ar*C*(1)), 161.6 (*C*(4)=O), 165.8 (*C*(1)=O).

HRMS (NSI^+) $C_{12}H_{14}NO_3$ $[M+H]^+$ found 220.0964, requires 220.0968 (−1.9 ppm).

ν_{max} ($CHCl_3$, cm^{-1}) 3350 (N-H), 2978 (C-H), 1705 ($C{=}O_{ester}$), 1678 ($C{=}O_{amide}$), 1649 (C=C).

2.15 (*E*)-4-oxo-4-(phenylamino)-2-butenoic acid (SI-10)

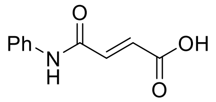

$C_{10}H_9NO_3$
191.19
SI-10

Following General Procedure 1.3, ester **SI-9** (2.7 g, 12.3 mmol, 1.0 eq) and $LiOH{\cdot}H_2O$ (568 mg, 13.5 mmol, 1.1 eq) in H_2O : THF 1:1 (13 mL) gave the title compound after extraction with EtOAc as off-white solid (2.97 g, 99%), which was used without further purification.

^{1}**H NMR** (400 MHz, DMSO-d$_6$) δ_H: 6.66 (1H, d, *J* 15.4, C*H*), 7.07 – 7.12 (1H, m, ArC(4)*H*), 7.15 (1H, d, *J* 15.4, C*H*), 7.31 – 7.37 (2H, m, ArC(2,6)*H*), 7.65 – 7.70 (2H, m, ArC(3,5)*H*), 10.52 (1H, s, C(O)O*H*).

2.16 (*E*)-4-hydroxy-*N*-phenyl-2-butenamide (SI-11)

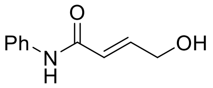

$C_{10}H_{11}NO_2$
177.20
SI-11

Following General Procedure 1.4, acid **SI-10** (2.5 g, 13.0 mmol, 1.0 eq), Et_3N (2.0 mL, 14.3 mmol, 1.1 eq) and ethyl chloroformate (1.4 mL, 14.3 mmol, 1.1 eq) in anhydrous THF (45 mL) followed by $NaBH_4$ (1.2 g, 32.7 mmol, 2.5 eq) in H_2O (25 mL) gave the title compound after recrystallisation from EtOAc as colourless solid (636 mg, 28%). **m.p.** (EtOAc) 160-163 °C.

^{1}**H NMR** (500 MHz, MeOH-*d$_4$*) δ_H: 4.29 (2H, dd, *J* 3.9, 2.1, C(4)*H*$_2$), 6.37 (1H, dt, *J* 15.3, 2.1, C(2)*H*), 6.99 (1H, dt, *J* 15.3, 3.9, C(3)*H*), 7.09 (1H, t, *J* 7.4, ArC(4)*H*), 7.28 – 7.34 (2H, m, ArC(2,6)*H*), 7.61 (d, *J* 7.7, ArC(3,5)*H*).

Data in accordance with literature.[18]

2.17 (*E*)-(4-oxo-4-(phenylamino)but-2-en-1-yl) phosphoric acid, diethyl ester (32)

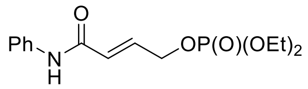

$C_{14}H_{20}NO_5P$
313.29
32

Following General Procedure 1.1, alcohol **SI-11** (550 mg, 3.1 mmol, 1.0 eq), diethyl chlorophosphate (0.67 mL, 4.6 mmol, 1.5 eq), Et_3N (0.65 mL, 4.6 mmol, 1.5 eq) and DMAP (95 mg, 0.8 mmol, 0.25 eq) in anhydrous CH_2Cl_2 (30 mL) gave the title compound after purification by Biotage® Isolera™ 4 [SNAP Ultra 25 g, 75 mL min^{-1}, petrol : EtOAc (50:50 5 CV, 50:50 to 0:100 14 CV, 0:100 6 CV), R_f 0.28 in EtOA] as pale yellow oil, which solidified upon freezing. Recrystallisation from toluene afforded the title compound as colourless crystalline solid (864 mg, 89%). **m.p.** (EtOAc) 76-79 °C.

^{1}H NMR (500 MHz, $CDCl_3$) δ_H: 1.33 (6H, t, *J* 7.1, $P(OCH_2CH_3)_2$), 4.13 (4H, app. p, *J* 7.3, $P(OCH_2CH_3)_2$), 4.70 (2H, ddd, *J* 7.0, 4.2, 1.9, C(1)H_2), 6.38 (1H, dt, *J* 15.1, 1.8, C(3)*H*), 6.95 (1H, dtd, *J* 15.2, 4.2, 1.8, C(2)*H*), 7.09 (1H, t, *J* 7.4, ArC(4)*H*), 7.30 (2H, t, *J* 7.9, ArC(3,5)*H*), 7.65 (2H, d, *J* 7.9, ArC(2,6)*H*), 8.63 (1H, s, N*H*).

^{13}C{^{1}H} NMR (126 MHz, $CDCl_3$) δ_C: 16.1 (d, $^3J_{CP}$ 6.6, $P(OCH_2CH_3)_2$), 64.2 (d, $^2J_{CP}$ 5.9, $P(OCH_2CH_3)_2$), 65.9 (d, $^2J_{CP}$ 5.4, *C*(1)H_2), 119.9 (Ar*C*(2,6)H), 124.2 (Ar*C*(4)H), 125.2 (*C*(3)H), 128.9 (Ar*C*(3,5)H), 137.7 (d, $^3J_{CP}$ 7.5, *C*(2)H), 138.3 (Ar*C*(1)), 163.2 (*C*=O).

^{31}P{^{1}H} NMR (121 MHz, $CDCl_3$) δ_P: -1.74 (O*P*(O)(OEt)$_2$).

HRMS (ESI$^+$) $C_{14}H_{20}NO_5PNa$ $[M+Na]^+$ found 336.0963, requires 336.0971 (−2.4 ppm).

ν_{max} (film, cm^{-1}) 3269 (N-H), 3130 (=C-H), 2991 (C-H), 1687 (C=O), 1651 (C=C), 1600 (C=C_{Ar}), 1492 (C=C_{Ar}), 1240 (P=O), 1018 (P-O).

2.18 (*E*)-4-(Methylphenylamino)-4-oxo-2-butenoic acid, ethyl ester (SI-12)

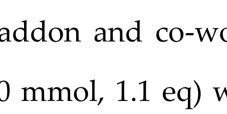

$C_{13}H_{15}NO_3$
233.27
SI-12

Adapting the procedure of Snaddon and co-workers,[5] in a flame dried flask under argon atmosphere, NaH (602 mg, 15.0 mmol, 1.1 eq) was activated by trituating with hexane (3 × 5 mL). Residual solvent was removed under reduced pressure and the grey powder subsequently suspended in anhydrous THF (35 mL). Ester **SI-9** (3.0 g, 13.6 mmol, 1.0 eq) dissolved in anhydrous THF (35 mL) was slowly added to the NaH suspension at 0 °C. After gas expulsion had ceased, the reaction mixture was allowed to warm to room temperature.

MeI (0.93 mL, 15.0 mmol, 1.1 eq) was added, the flask sealed and heated to 80 °C for 16 h. After cooling to room temperature, the reaction was quenched with sat. NH_4Cl solution (40 mL), the phases separated and the aqueous phase extracted with Et_2O (3 × 60 mL). The combined organic phases were dried over MgSO4, filtered and the solvent removed under reduced pressure to afford the crude product as black oil. Purification by Biotage® Isolera™ 4 [SNAP Ultra 50 g, 100 mL min^{-1}, petrol : EtOAc 4:1 (15 CV), R_f 0.16] afforded the title compound as off-white solid (1.73 g, 55 %). **m.p.** (petrol) 74-77 °C.

^{1}H NMR (500 MHz, $CDCl_3$) δ_H: 1.24 (3H, t, *J* 7.1, OCH_2CH_3), 3.38 (3H, s, NCH_3), 4.16 (2H, q, *J* 7.1, OCH_2CH_3), 6.85 (2H, s, C(2)*H*, C(3)*H*), 7.14 – 7.19 (2H, m, ArC(2,6)*H*), 7.34 – 7.38 (1H, m, ArC(4)*H*), 7.40 – 7.46 (2H, m, ArC(3,5)*H*).

^{13}C{^{1}H} NMR (126 MHz, $CDCl_3$) δ_C: 14.2 (OCH_2CH_3), 37.8 (NCH_3), 61.1 (OCH_2CH_3), 127.1 (ArC(2,6)H), 128.2 (ArC(4)H), 130.0 (ArC(3,5)H), 131.1 (=CH), 134.3 (=CH), 142.7 (ArC(1)), 164.1 (C(4)=O), 165.8 (C(1)=O).

HRMS (NSI$^+$) $C_{13}H_{16}NO_3$ [M+H]$^+$ found 234.1127, requires 234.1125 (+1.0 ppm).

ν_{max} (film, cm^{-1}) 3049 (=C-H), 2991 (C-H), 1716 (C=O_{ester}), 1660 (C=O_{amide}), 1631 (C=C), 1593 (C=C_{Ar}), 1492 (C=C_{Ar}).

2.19 (*E*)-4-(Methylphenylamino)-4-oxo-2-butenoic acid (SI-13)

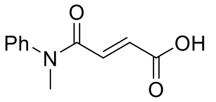

$C_{11}H_{11}NO_3$
205.21
SI-13

Following General Procedure 1.3, ester **SI-12** (1.5 g, 6.4 mmol, 1.0 eq) and LiOH·H_2O (297 mg, 7.0 mmol, 1.1 eq) in H_2O : THF 1:1 (7 mL) gave the title compound after extraction with CH_2Cl_2 as off-white solid (1.28 g, 97%), which was used without further purification.

^{1}H NMR (500 MHz, DMSO) δ_H: 3.28 (3H, s, NCH_3), 6.55 (1H, d, *J* 15.3, C(2)*H*), 6.64 (1H, d, *J* 15.3, C(3)*H*), 7.31 – 7.36 (2H, m, ArC(2,6)*H*), 7.38 – 7.44 (1H, m, ArC(4)*H*), 7.46 – 7.52 (2H, m, ArC(3,5)*H*).

2.20 (*E*)-4-Hydroxy-*N*-methyl-*N*-phenyl-2-butenamide (SI-14)

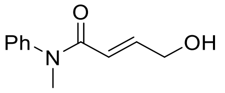

$C_{11}H_{13}NO_2$
191.23
SI-14

Following General Procedure 1.4, acid **SI-13** (2.6 g, 13.0 mmol, 1.0 eq), Et_3N (2.0 mL, 14.3 mmol, 1.1 eq) and ethyl chloroformate (1.4 mL, 14.3 mmol, 1.1 eq) in anhydrous THF (45 mL) followed by $NaBH_4$ (1.2 g, 32.7 mmol, 2.5 eq) in H_2O (25 mL) gave the title compound after purification by silica column chromatography (CH_2Cl_2 : Acetone 7:3, R_f 0.48 in CH_2Cl_2 : Acetone 1:1) as colourless oil (1.7 g, 70%), which solidified slowly. **m.p.** (EtOAc) 70-73 °C.

^{1}H NMR (500 MHz, $CDCl_3$) δ_H: 1.84 (1H, br s, O*H*), 3.34 (3H, s, NC*H*$_3$), 4.19 – 4.24 (2H, m, C(4)*H*$_2$), 6.00 (1H, d, *J* 15.1, C(2)*H*), 6.98 (1H, dt, *J* 15.2, 4.2, C(3)*H*), 7.15 – 7.19 (2H, m, ArC(2,6)*H*), 7.30 – 7.35 (1H, m, ArC(4)*H*), 7.38 – 7.43 (2H, m, ArC(3,5)*H*).

^{13}C{^{1}H} NMR (126 MHz, $CDCl_3$) δ_C: 37.5 (N*C*H$_3$), 62.1 (*C*(4)H$_2$), 120.1 (*C*(2)H), 127.2 (Ar*C*(2,6)H), 127.6 (Ar*C*(4)H), 129.6 (Ar*C*(3,5)H), 143.4 (Ar*C*(1)), 143.9 (*C*(3)H), 165.8 (C=O).

HRMS (NSI$^-$) $C_{11}H_{12}NO_2$ [M−H]$^-$ found 190.0877, requires 190.0874 (+1.8 ppm).

ν_{max} (film, cm^{-1}) 3385 (O-H), 3053 (Ar-H), 2864 (C-H), 1654 (C=O), 1600 (C=C), 1492 (C=C$_{Ar}$).

2.21 (*E*)-(4-(Methyl(phenyl)amino)-4-oxobut-2-en-1-yl) phosphoric acid, diethyl ester (33)

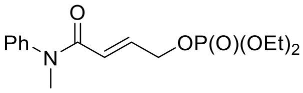

$C_{15}H_{22}NO_5P$
327.32
33

Following General Procedure 1.1, alcohol **SI-14** (516 mg, 2.7 mmol, 1.0 eq), diethyl chlorophosphate (0.58 mL, 4.0 mmol, 1.5 eq), Et_3N (0.56 mL, 4.0 mmol, 1.5 eq) and DMAP (82 mg, 0.7 mmol, 0.25 eq) in anhydrous CH_2Cl_2 (30 mL) gave the title compound after purification by silica column chromatography (EtOAc : 1% *i*PrOH to 5% *i*PrOH, R_f 0.41 in EtOAc : 10% *i*PrOH) as pale yellow oil (670 mg, 76%).

^{1}H NMR (500 MHz, $CDCl_3$) δ_H: 1.23 (6H, t, *J* 7.1, P(OCH$_2$C*H*$_3$)$_2$)), 3.34 (3H, s, NC*H*$_3$), 3.92 – 4.01 (4H, m, P(OC*H*$_2$CH$_3$)$_2$)), 4.53 – 4.62 (2H, m, C(1)*H*$_2$), 6.01 (1H, d, *J* 15.1, C(3)*H*), 6.88 (1H, dtd, *J* 15.1, 4.4, 1.4, C(2)*H*), 7.14 – 7.19 (2H, m, ArC(2,6)*H*), 7.30 – 7.35 (1H, ArC(4)*H*), 7.37 – 7.43 (2H, ArC(3,5)*H*).

^{13}**C{^{1}H} NMR** (126 MHz, $CDCl_3$) δ$_C$: 16.2 (d, $^3J_{CP}$ 6.6, P(OCH_2CH_3)$_2$), 37.6 (NCH_3), 64.0 (d, $^2J_{CP}$ 5.9, P(OCH_2CH_3)$_2$), 65.8 (d, $^2J_{CP}$ 5.0, *C*(1)H_2), 122.1 (*C*(3)H), 127.4 (Ar*C*(2,6)H), 127.8 (Ar*C*(4)H), 129.7 (Ar*C*(3,5)H), 138.3 (d, $^3J_{CP}$ 7.5, *C*(2)H), 143.4 (Ar*C*(1)), 165.1 (*C*=O).

^{31}**P{^{1}H} NMR** (202 MHz, $CDCl_3$) δ$_P$: -1.26 (O*P*(O)(OEt)$_2$).

HRMS (NSI$^+$) $C_{15}H_{23}NO_5P$ [M+H]$^+$ found 328.1310, requires 328.1308 (+0.5 ppm).

ν$_{max}$ (film, cm^{-1}) 2983 (C-H), 1670 (C=O), 1631 (C=C), 1595 (C=C$_{Ar}$), 1496 (C=C$_{Ar}$), 1263 (P=O).

2.22 (*E*)-4-[Methyl(phenylmethyl)amino]-4-oxo-2-butenoic acid, ethyl ester (SI-15)

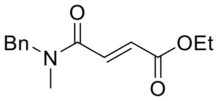

$C_{14}H_{17}NO_3$
247.29
SI-15

Following General Procedure 1.2 monoethyl fumarate (7.5 g, 52.0 mmol, 1.0 eq), *N*-benzylmethylamine (7.4 mL, 57.2 mmol, 1.1 eq), EDCI·HCl (10.9 g, 57.2 mmol, 1.1 eq) and DMAP (636 mg, 5.2 mmol, 0.1 eq) in CH_2Cl_2 (130 mL) gave a rotameric mixture (55:45) of the title compound as yellow oil, (12.7 g, 98%), which was used without further purification.

^{1}**H NMR** (400 MHz, $CDCl_3$) *major rotamer* δ$_H$: 1.31 (3H, t, *J* 7.1, OCH_2CH_3), 3.02 (3H, s, NCH_3), 4.24 (2H, app. dq, *J* 14.2, 7.1, OCH_2CH$_3$), 4.66 (2H, s, NCH_2), 6.85 (1H, d, *J* 15.3, C(2)*H* or C(3)*H*), 7.14 – 7.19 (1H, m, ArC(4)*H*), 7.23 – 7.39 (4H, m, ArC(2,3,5,6)*H*), 7.43 (1H, d, *J* 15.3, C(2)*H* or C(3)*H*); *minor rotamer (selected)* δ$_H$: 1.28 (3H, t, *J* 7.1, OCH_2CH_3), 3.00 (3H, s, NCH_3), 4.61 (2H, s, NCH_2), 6.85 (1H, d, *J* 15.3, C(2)*H* or C(3)*H*), 7.41 (1H, d, *J* 15.3, C(2)*H* or C(3)*H*).

2.23 (*E*)-4-[Methyl(phenylmethyl)amino]-4-oxo-2-butenoic acid (SI-16)

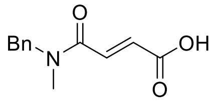

$C_{12}H_{13}NO_3$
219.24
SI-16

Following General Procedure 1.3, ester **SI-15** (12.1 g, 48.9 mmol, 1.0 eq) and LiOH·H_2O (2.3 g, 53.8 mmol, 1.1 eq) in H_2O : THF 1:1 (50 mL) gave a rotameric mixture (55:45) of the title compound after extraction with CH_2Cl_2 as yellow oil (9.9 g, 93%), which was used without further purification.

^{1}**H NMR** (400 MHz, MeOH-*d$_4$*) *major rotamer* δ$_H$: 3.08 (3H, s, NCH_3), 4.67 (2H, s, NCH_2), 6.71 (1H, d, *J* 15.3, C(2)*H* or C(3)*H*), 7.19 – 7.42 (5H, m, 5 × Ar*H*), 7.49 (1H, dd, *J* 15.4, 13.8 Hz, 1H), 7.51 (1H, d, *J* 15.3, C(2)*H* or C(3)*H*); *minor rotamer (selected)* δ$_H$: 3.01 (3H, s, NCH_3), 4.72 (2H, s, NCH_2), 6.70 (1H, d, *J* 15.3, C(2)*H* or C(3)*H*), 7.47 (1H, d, *J* 15.3, C(2)*H* or C(3)*H*).

2.24 (*E*)-4-Hydroxy-*N*-methyl-*N*-(phenylmethyl)-2-butenamide (SI-17)

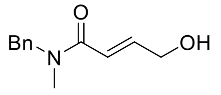

$C_{12}H_{15}NO_2$
205.26
SI-17

Following General Procedure 1.4, acid **SI-16** (9.8 g, 44.8 mmol, 1.0 eq), Et_3N (6.8 mL, 49.2 mmol, 1.1 eq) and ethyl chloroformate (4.7 mL, 49.2 mmol, 1.1 eq) in anhydrous THF (110 mL) followed by $NaBH_4$ (4.2 g, 112 mmol, 2.5 eq) in H_2O (70 mL) gave the title compound as a rotameric mixture (55:45) after purification by silica column chromatography (petrol : EtOAc 1:1 to EtOAc, R_f 0.20 in EtOAc) as yellow oil (6.2 g, 67%). Attempts to remove remaining impurities were unsuccessful, so alcohol **SI-17** was used as obtained after silica column chromatography.

^{1}H NMR (500 MHz, $CDCl_3$) *major rotamer* δ_H: 2.97 (3H, s, NC*H*$_3$), 4.31 – 4.36 (2H, m, C(4)*H*$_2$), 4.62 (2H, s, NC*H*$_2$), 6.52 – 6.63 (1H, m, C(2)*H* or C(3)*H*), 6.96 – 7.04 (1H, m, C(2)*H* or C(3)*H*), 7.12 – 7.18 (1H, m, Ar(4)*H*), 7.19 – 7.39 (4H, m, Ar(2,3,5,6)*H*); *minor rotamer (selected)* δ_H: 2.96 (3H, s, NC*H*$_3$), 4.26 – 4.31 (2H, m, C(4)*H*$_2$), 4.58 (2H, s, NC*H*$_2$).

^{13}C{^{1}H} NMR (126 MHz, $CDCl_3$) *major rotamer* δ_C: 35.0 (NCH_3), 51.1 (NCH_2), 62.0 (*C*(4)H_2), 118.8 (*C*(2)H or *C*(3)H), 126.5 (ArC(4)H), 128.0 (Ar*C*(2,6)H or Ar*C*(3,5)H), 128.6 (Ar*C*(2,6)H or Ar*C*(3,5)H), 137.1 (Ar*C*(1)), 145.3 (*C*(2)H or *C*(3)H), 166.8 (*C*=O); *minor rotamer (selected)* δ_C: 34.0 (NCH_3), 53.4 (NCH_2), 62.0 (*C*(4)H_2), 136.5 (Ar*C*(1)), 145.2 (*C*(2)H or *C*(3)H), 167.3 (*C*=O).

2.25 (*E*)-(4-(Benzyl(methyl)amino)-4-oxobut-2-en-1-yl) phosphoric acid, diethyl ester (34)

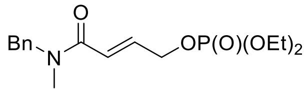

$C_{16}H_{24}NO_5P$
341.34
34

Following General Procedure 1.1, alcohol **SI-17** (560 mg, 2.7 mmol, 1.0 eq), diethyl chlorophosphate (0.59 mL, 4.1 mmol, 1.5 eq), Et_3N (0.57 mL, 4.1 mmol, 1.5 eq) and DMAP (83 mg, 0.7 mmol, 0.25 eq) in anhydrous CH_2Cl_2 (30 mL) gave the title compound as a rotameric mixture (55:45) after purification by silica column chromatography (EtOAc : 5% *i*PrOH to 10% *i*PrOH) as colourless oil (730 mg, 78%). Attempts to remove remaining impurities were unsuccessful, so phosphate **34** was used as obtained after silica column chromatography (ca. 25% impurities).

^{1}**H NMR** (500 MHz, $CDCl_3$) *major rotamer* δ_H: 1.28 – 1.33 (6H, m, P(OCH_2CH_3)$_2$), 2.96 (3H, s, NCH_3), 4.06 – 4.15 (4H, m, P(OCH_2CH_3)$_2$), 4.61 (2H, s, NCH_2), 4.69 (2H, ddd, *J* 7.5, 4.3, 2.0, C(1)H_2), 6.59 (1H, dt, *J* 15.0, 2.0, C(3)*H*), 6.84 – 6.94 (1H, m, C(2)*H*), 7.11 – 7.16 (1H, m, Ar(4)*H*), 7.17 – 7.35 (4H, m, Ar(2,3,5,6)*H*); *minor rotamer (selected)* δ_H: 1.20 – 1.25 (6H, m, P(OCH_2CH_3)$_2$), 2.96 (3H, s, NCH_3), 3.97 – 4.03 (4H, m, P(OCH_2CH_3)$_2$), 4.56 (2H, s, NCH_2), 4.63 (2H, ddd, *J* 7.6, 4.3, 2.0, C(1)H_2), 6.53 (1H, dt, *J* 15.0, 2.0, C(3)*H*).

^{13}**C{^{1}H} NMR** (126 MHz, $CDCl_3$) *major rotamer* δ_C: 16.0 (d, $^3J_{CP}$ 6.8, P(OCH_2CH_3)$_2$), 34.9 (NCH_3), 51.1 (NCH_2), 63.9 – 64.0 (m, P(OCH_2CH_3)$_2$), 66.0 (d, $^2J_{CP}$ 5.2, *C*(1)H_2), 121.1 (*C*(3)H), 126.5 (Ar*C*(4)H), 128.0 (Ar*C*(2,6)H or Ar*C*(3,5)H), 128.9 (Ar*C*(2,6)H or Ar*C*(3,5)H), 137.0 (Ar*C*(1)), 139.0 (*C*(2)H), 165.7 (*C*=O); *minor rotamer (selected)* δ_C: 16.1 (d, $^3J_{CP}$ 6.8, P(OCH_2CH_3)$_2$), 34.1 (NCH_3), 53.3 (NCH_2), 65.9 (d, $^2J_{CP}$ 5.1, *C*(1)H_2), 121.0 (*C*(3)H), 136.4 (Ar*C*(1)), 139.0 (*C*(2)H), 166.2 (*C*=O).

^{31}**P{^{1}H} NMR** (162 MHz, $CDCl_3$) *major rotamer* δ_P: -1.09 (O*P*(O)(OEt)$_2$); *minor rotamer* δ_P: -1.15 (O*P*(O)(OEt)$_2$).

2.26 (*E*)- 4-Oxo-4-(pyrrolidine-1-yl)-2-butenoic acid, ethyl ester (SI-18)

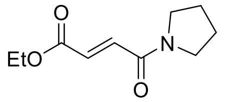

$C_{10}H_{15}NO_3$
197.23
SI-18

Adapting the procedure of Chen and co-workers,[19] monoethyl fumarate (2.0 g, 13.9 mmol, 1.0 eq) was dispersed in anhydrous toluene (30 mL) under an inert atmosphere. $SOCl_2$ (2.02 mL, 27.7 mmol, 2.0 eq) was added and the reaction mixture heated at reflux for 3 h. After cooling to rt, the solvent was removed under reduced pressure and the crude mixture dissolved in anhydrous THF (15 mL). Pyrrolidine (2.8 mL, 34.7 mmol, 2.5 eq) was added at 0 °C and the reaction stirred for 1 h at rt. The mixture was diluted with H_2O (10 mL) and acidified with 1 M HCl to pH 3. The aqueous mixture was extracted with CH_2Cl_2 (3 × 10 mL) and the combined organic phases washed with 1 M HCl (15 mL), sat. aq. $NaHCO_3$ (15 mL) and brine (15 mL), dried over $MgSO_4$, filtered and the solvent removed under reduced pressure. The crude product was purified by silica column chromatography (*n*-hexane : EtOAc 2:1 to 1:1, R_f 0.2 in *n*-hexane : EtOAc 1:1) to afford the title compound as a pale orange oil (2.06 g, 77%).

^{1}**H NMR** (400 MHz, MeOD-*d*$_4$) δ_H: 1.33 (3H, t, *J* 7.1, OCH$_2$C*H*$_3$), 1.90 – 2.00 (2H, m, C*H*$_{2(pyrr)}$), 2.00 – 2.09 (2H, m, C*H*$_{2(pyrr)}$), 3.53 (2H, t, *J* 6.9, NC*H*$_{2(pyrr)}$), 3.68 (2H, t, *J* 6.8, NC*H*$_{2(pyrr)}$), 4.27 (2H, q, *J* 7.1, OC*H*$_2$CH$_3$), 6.74 (1H, d, *J* 15.4, C(2)*H*), 7.34 (1H, d, *J* 15.4, C(3)*H*).

Data in accordance with literature.[6]

2.27 (*E*)-4-Oxo-4-(1-pyrrolidinyl)-2-butenoic acid (SI-19)

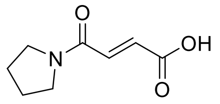

$C_8H_{11}NO_3$
169.18
SI-19

Following General Procedure 1.3, ester **SI-18** (1.0 g, 5.0 mmol, 1.0 eq) and LiOH·H$_2$O (234 mg, 5.5 mmol, 1.1 eq) in H$_2$O : THF 1:1 (6.0 mL) gave the title compound as a yellow solid (848 mg, 98%). **m.p.** 156 – 158 (*dec*).

^{1}**H NMR** (400 MHz, DMSO-*d*$_6$) δ_H: 1.75 – 1.84 (2H, m, C*H*$_{2(pyrr)}$), 1.85 – 1.94 (2H, m, C*H*$_{2(pyrr)}$), 3.36 (2H, t, *J* 6.8, NC*H*$_{2(pyrr)}$), 3.57 (2H, t, *J* 6.8, NC*H*$_{2(pyrr)}$), 6.52 (1H, d, *J* 15.3, C(2)*H* or C(3)*H*), 7.16 (1H, d, *J* 15.3, C(2)*H* or C(3)*H*).

Data in accordance with literature.[6]

2.28 (*E*)-4-hydroxy-1-(pyrrolidin-1-yl)but-2-en-1-one (SI-20)

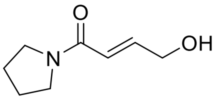

$C_8H_{13}NO_2$
155.20
SI-20

Following General Procedure 1.4, acid **SI-19** (6.8 g, 40.2 mmol, 1.0 eq), Et$_3$N (6.1 mL, 44.2 mmol, 1.1 eq) and ethyl chloroformate (4.2 mL, 44.2 mmol, 1.1 eq) in anhydrous THF (100 mL) followed by NaBH$_4$ (3.8 g, 100.4 mmol, 2.5 eq) in H$_2$O (60 mL) gave the title compound after purification by silica column chromatography (CH$_2$Cl$_2$: Acetone 1:1 to 3:7, R$_f$ 0.22 in CH$_2$Cl$_2$: Acetone 3:7) followed by recrystallisation from toluene as colourless solid (908 mg, 15%). **m.p.** (toluene) 86-88 °C.

^{1}**H NMR** (500 MHz, CDCl$_3$) δ_H: 1.86 (2H, p, *J* 6.8, C*H*$_{2(pyrr)}$), 1.95 (2H, p, *J* 6.8, C*H*$_{2(pyrr)}$), 2.93 (1H, br s, O*H*), 3.48 – 3.55 (4H, m, 2 × NC*H*$_{2(pyrr)}$), 4.29 – 4.36 (2H, m, C(4)*H*$_2$), 6.39 (1H, dt, *J* 15.2, 2.0, C(2)*H*), 6.96 (1H, dt, *J* 15.2, 3.9, C(3)*H*).

^{13}**C{^{1}H} NMR** (126 MHz, CDCl$_3$) δ_C: 24.4 (*C*H$_{2(pyrr)}$), 26.1 (*C*H$_{2(pyrr)}$), 46.0 (N*C*H$_{2(pyrr)}$), 46.7 (N*C*H$_{2(pyrr)}$), 62.1 (*C*(4)H$_2$), 120.2 (*C*(2)H), 144.1 (*C*(3)H), 164.8 (*C*=O).

HRMS (NSI$^+$) $C_8H_{14}NO_2$ [M+H]$^+$ found 156.1016, requires 156.1019 (−2.0 ppm).

ν_{max} (film, cm^{-1}) 3302 (O-H), 2872 (C-H), 1662 (C=O), 1591 (C=C).

2.29 (*E*)-(4-Oxo-4-(pyrrolidin-1-yl)but-2-en-1-yl) phosphoric acid, diethyl ester (35)

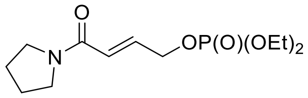

$C_{12}H_{22}NO_5P$
291.28
35

Following General Procedure 1.1, alcohol **SI-20** (770 mg, 4.96 mmol, 1.0 eq), diethyl chlorophosphate (1.0 mL, 7.44 mmol, 1.5 eq), Et_3N (1.0 mL, 7.44 mmol, 1.5 eq) and DMAP (151 mg, 1.24 mmol, 0.25 eq) in anhydrous CH_2Cl_2 (50 mL) gave the title compound after purification by silica column chromatography (CH_2Cl_2 : 2.5% MeOH, R_f 0.43 in CH_2Cl_2 : MeOH 9:1) as pale pink oil (1.3 g, 91 %).

^{1}H NMR (500 MHz, $CDCl_3$) δ_H: 1.30 (6H, t, *J* 7.1, $P(OCH_2CH_3)_2$), 1.84 (2H, p, *J* 6.7, $CH_{2(pyrr)}$), 1.93 (2H, p, *J* 6.7, $CH_{2(pyrr)}$), 3.49 (4H, td, *J* 6.8, 3.8, $NCH_{2(pyrr)}$), 4.09 (4H, app. p, *J* 7.2, $P(OCH_2CH_3)_2$), 4.66 (2H, ddd, *J* 7.0, 4.3, 1.9, $C(1)H_2$), 6.39 (1H, dt, *J* 15.1, 1.9, C(3)*H*), 6.84 (1H, dtd, *J* 15.1, 4.3, 1.7, C(2)*H*).

^{13}C{^{1}H} NMR (126 MHz, $CDCl_3$) δ_C: 16.1 (d, $^3J_{CP}$ 6.7, $P(OCH_2CH_3)_2$), 24.3 ($CH_{2(pyrr)}$), 26.1 ($CH_{2(pyrr)}$), 45.9 ($NCH_{2(pyrr)}$), 46.6 ($NCH_{2(pyrr)}$), 64.0 (d, $^2J_{CP}$ 5.8, $P(OCH_2CH_3)_2$), 66.0 (d, $^2J_{CP}$ 5.1, $C(1)H_2$), 122.4 (*C*(3)H), 137.8 (d, $^2J_{CP}$ 7.5, *C*(2)H), 163.7 (C(4)=O).

^{31}P{^{1}H} NMR (202 MHz, $CDCl_3$) δ_P: -1.12 ($OP(O)(OEt)_2$).

HRMS (ESI^+) $C_{12}H_{22}NO_5PNa$ $[M+Na]^+$ found 314.1120 , requires 314.1128 (−2.5 ppm).

ν_{max} (film, cm^{-1}) 2978 (C-H), 2873 (C-H), 1670 (C=O), 1616 (C=C), 1265 (P=O), 1020 (P-O).

2.30 (E)-4-(4-Morpholinyl)-4-oxo-2-butenoic acid, ethyl ester (SI-21)

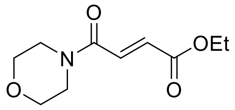

$C_{10}H_{15}NO_4$
213.23
SI-21

Following General Procedure 1.2, monoethyl fumarate (7.5 g, 52.0 mmol, 1.0 eq), morpholine (5.0 mL, 57.2 mmol, 1.1 eq), EDCI·HCl (10.9 g, 57.2 mmol, 1.1 eq) and DMAP (636 mg, 5.2 mmol, 0.1 eq) in CH_2Cl_2 (130 mL) gave the title compound as off-white solid (10.6 g, 95 %), which was used without further purification.

^{1}H NMR (400 MHz, $CDCl_3$) δ_H: 1.31 (3H, t, *J* 7.1, OCH_2CH_3), 3.56 – 3.60 (2H, m, $CH_{2(morph)}$), 3.67 – 3.73 (6H, m, 3 × $CH_{2(morph)}$), 4.25 (2H, q, *J* 7.1, OCH_2CH_3), 6.78 (1H, d, *J* 15.3, C*H*), 7.35 (1H, d, *J* 15.3, C*H*).

2.31 (*E*)-4-(4-Morpholinyl)-4-oxo-2-butenoic acid (SI-22)

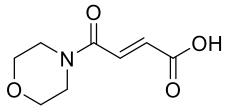

$C_8H_{11}NO_4$
185.18
SI-22

Following General Procedure 1.3, ester **SI-21** (9.8 g, 46.3 mmol, 1.0 eq) and LiOH·H_2O (2.1 g, 50.9 mmol, 1.1 eq) in H_2O : THF 1:1 (50 mL) gave the title compound as off-white solid (5.75 g, 67%), which was used without further purification.

^{1}H NMR (400 MHz, MeOH-d_4) δ_H: 3.62 – 3.72 (8H, m, 4 × C*H*$_{2(morph)}$), 6.65 (1H, d, *J* 15.4, C*H*), 7.45 (1H, d, *J* 15.4, C*H*).

2.32 (*E*)-4-Hydroxy-*N*-morpholinyl-2-butenamide (SI-23)

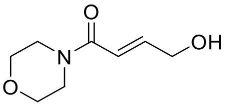

$C_8H_{13}NO_3$
171.20
SI-23

Following General Procedure 1.4, acid **SI-22** (5.4 g, 29.5 mmol, 1.0 eq), Et_3N (4.5 mL, 32.4 mmol, 1.1 eq) and ethyl chloroformate (3.1 mL, 32.4 mmol, 1.1 eq) in anhydrous THF (74 mL) followed by $NaBH_4$ (2.8 g, 73.7 mmol, 2.5 eq) in H_2O (46 mL) gave the title compound after purification by silica column chromatography (EtOAc : iPrOH 95:5 to 85:15, R_f 0.26 in EtOAc : iPrOH 85:15) followed by recrystallisation from toluene as colourless solid (808 mg, 16%). **m.p.** (toluene) 100-102 °C.

^{1}H NMR (500 MHz, $CDCl_3$) δ_H: 3.55 – 3.60 (2H, m, NC*H*$_{2(morph)}$), 3.64 – 3.71 (6H, m, 2 × OC*H*$_{2(morph)}$, NC*H*$_{2(morph)}$), 4.34 (2H, dd, *J* 3.7, 2.2, C(4)*H*$_2$), 6.52 (1H, dt, *J* 15.1, 2.1, C(2)*H*), 6.94 (1H, dt, *J* 15.2, 3.8, C(3)*H*).

^{13}C{^{1}H} NMR (126 MHz, $CDCl_3$) δ_C: 42.3 (N$CH_{2(morph)}$), 46.2 (N$CH_{2(morph)}$), 62.0 (*C*(4)H_2), 66.7 (O$CH_{2(morph)}$), 66.8 (O$CH_{2(morph)}$), 118.1 (*C*(2)H), 145.0 (*C*(3)H), 165.6 (*C*=O).

HRMS (NSI$^-$) $C_8H_{12}NO_3$ [M−H]$^-$ found 170.0827, requires 170.0823 (+2.5 ppm).

ν_{max} (film, cm^{-1}) 3412 (O-H), 2872 (C-H), 1666 (C=O), 1604 (C=C).

2.33 (*E*)-(4-Morpholino-4-oxobut-2-en-1-yl) phosphoric acid, diethyl ester (36)

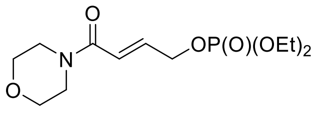

$C_{12}H_{22}NO_6P$
307.28
36

Following General Procedure 1.1, alcohol **SI-23** (417 mg, 2.43 mmol, 1.0 eq), diethyl chlorophosphate (0.53 mL, 3.64 mmol, 1.5 eq), Et_3N (0.51 mL, 3.64 mmol, 1.5 eq) and DMAP (74 mg, 0.6 mmol, 0.25 eq) in anhydrous CH_2Cl_2 (25 mL) gave the title compound after purification by silica column chromatography (CH_2Cl_2 : 2.5% MeOH, R_f 0.55 in CH_2Cl_2 : MeOH 9:1) as pale yellow oil (673 mg, 94 %).

^{1}H NMR (500 MHz, $CDCl_3$) δ_H: 1.30 (6H, td, *J* 7.1, 0.9, P(OCH$_2$C*H*$_3$)$_2$), 3.49 – 3.55 (2H, m, NC*H*$_{2(morph)}$), 3.60 – 3.68 (6H, m, NC*H*$_{2(morph)}$, 2 × OC*H*$_{2(morph)}$), 4.10 (4H, app. p, P(OC*H*$_2$CH$_3$)$_2$), 4.66 (2H, ddd, *J* 7.5, 4.2, 2.0, C(1)*H*$_2$), 6.51 (1H, dt, *J* 15.1, 2.0, C(3)*H*), 6.82 (1H, dtd, *J* 15.1, 4.2, 1.8, C(2)*H*).

^{13}C{^{1}H} NMR (126 MHz, $CDCl_3$) δ_C: 16.1 (d, $^3J_{CP}$ 6.6, P(OCH$_2$CH$_3$)$_2$), 42.3 (NCH$_{2(morph)}$), 46.2 (NCH$_{2(morph)}$), 64.0 (d, $^2J_{CP}$ 5.8, P(OCH$_2$CH$_3$)$_2$), 66.0 (d, $^2J_{CP}$ 5.2, *C*(1)H$_2$), 66.8 (2 × OCH$_{2(morph)}$), 120.4 (*C*(3)H), 139.1 (d, $^3J_{CP}$ 7.3, *C*(2)H), 164.6 (C=O).

^{31}P{^{1}H} NMR (202 MHz, $CDCl_3$) δ_P: -1.11 (O*P*(O)(OEt)$_2$).

HRMS (ESI$^+$) $C_{12}H_{22}NO_6PNa$ [M+Na]$^+$ found 330.1068, requires 330.1077 (−2.7 ppm).

ν_{max} (film, cm^{-1}) 2983 (C-H), 1668 (C=O), 1620 (C=C), 1265 (P=O), 1018 (P-O).

3. Tandem Pd/ITU Relay Catalysis Products

3.1 (2*R*,3*S*)-2-(Dimethylamino)-3-vinylbutanedioic acid, 4-ethyl 1-(4-nitrophenyl) ester (18$_{maj}$) and (2*R*,3*R*)-2-(Dimethylamino)-3-vinylbutanedioic acid, 4-ethyl 1-(4-nitrophenyl) ester (18$_{min}$)

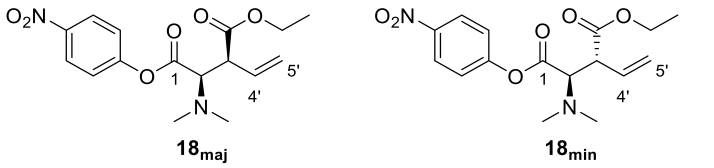

18$_{maj}$ **18$_{min}$**

$C_{16}H_{20}N_2O_6$
336.34

Following General Procedure 1.5, PNP ester **13** (65.0 mg, 0.25 mmol, 1.0 eq), FurCat **3** (9.3 mg, 12.5 μmol, 5 mol%), (*S*)-TM·HCl (12.0 mg, 0.05 mmol, 10 mol%), phosphate **14** (83.0 mg, 0.31 mmol, 1.25 eq) and *i*Pr$_2$NEt (0.1 mL, 0.6 mmol, 2.4 eq) in MeCN (4.4 mL) gave the title compound after purification by silica column chromatography (Petrol : EtOAc 6:1 to 4:1, R_f

0.23 in Petrol : EtOAc 6:1) as a yellow oil (60.0 mg, 71%) as an inseparable mixture of diastereomers (60:40 dr). $[\alpha]_D^{20}$ −0.5 (*c* 0.45 in $CHCl_3$).

HRMS (NSI^+) $C_{16}H_{21}N_2O_6$ $[M+H]^+$ found 337.1386, requires 337.1394 (−2.4 ppm).

ν_{max} (film, cm^{-1}) 3084 (C-H), 2981 (C-H), 1732 (C=O), 1716 (C=O), 1591 ($C{=}C_{Ar}$), 1523 (N=O), 1489 ($C{=}C_{Ar}$), 1344 (N=O).

Data for major diastereoisomer

Chiral HPLC analysis**,** Chiralcel OD-H, (*n*-hexane : *i*PrOH 99:1, flow rate 0.5 $mLmin^{-1}$, 254 nm, 30 °C) t_R (2*S*,3*R*): 26.7 min, t_R (2*R*,3*S*): 29.5 min, 10:90 er.

^{1}H NMR (300 MHz, $CDCl_3$) δ_H: 1.28 (3H, t, *J* 7.1, OCH_2CH_3), 2.47 (6H, s, $N(CH_3)_2$), 3.64 – 3.70 (1H, m, C(3)*H*), 3.85 (1H, d, *J* 11.4, C(2)*H*), 4.12 - 4.20 (2H, m, OCH_2CH_3), 5.28 – 5.40 (2H, m, C(5′)H_2), 5.89 (1H, ddd, *J* 17.1, 10.1, 8.9, C(4′)*H*), 7.21 – 7.26 (2H, m, Ar(2,6)*H*), 8.23 – 8.30 (2H, m, Ar(3,5)*H*).

$^{13}C\{^1H\}$ NMR (126 MHz, $CDCl_3$) δ_C: 14.1 (OCH_2CH_3), 41.8 ($N(CH_3)_2$), 51.3 (*C*(3)H), 61.0 (OCH_2CH_3), 69.2 (*C*(2)H), 120.9 (*C*(5′)H_2), 122.6 (Ar*C*(2,6)H), 125.3 (Ar*C*(3,5)H), 131.8 (*C*(4′)H), 145.5 (Ar*C*(1)), 154.9 (Ar*C*(4)), 166.7 (*C*(1)=O), 171.0 (*C*(4)=O).

Data for minor diastereoisomer

Chiral HPLC analysis**,** Chiralcel OD-H, (*n*-hexane : *i*PrOH 99:1, flow rate 0.5 $mLmin^{-1}$, 254 nm, 30 °C) t_R (2*R*,3*R*): 25.0 min, t_R (2*S*,3*S*): 41.7 min, 70:30 er.

^{1}H NMR (300 MHz, $CDCl_3$) δ_H: 1.23 (3H, t, *J* 7.1, OCH_2CH_3), 2.46 (6H, s, $N(CH_3)_2$), 3.60 – 3.68 (1H, m, C(3)*H*), 3.88 (1H, d, *J* 11.4, C(2)*H*), 4.16 - 4.26 (2H, m, OCH_2CH_3), 5.25 – 5.34 (2H, m, C(5′)H_2), 5.84 (1H, ddd, *J* 17.3, 10.0, 8.3, C(4′)*H*), 7.27 – 7.32 (2H, m, Ar(2,6)*H*), 8.23 – 8.30 (2H, m, Ar(3,5)*H*).

$^{13}C\{^1H\}$ NMR (126 MHz, $CDCl_3$) δ_C: 14.1 (OCH_2CH_3), 41.7 ($N(CH_3)_2$), 49.6 (*C*(3)H), 61.3 (OCH_2CH_3), 67.8 (*C*(2)H), 119.4 (*C*(5′)H_2), 122.6 (Ar*C*(2,6)H), 125.3 (Ar*C*(3,5)H), 132.5 (*C*(4′)H), 145.5 (Ar*C*(1)), 155.1 (Ar*C*(4)), 168.0 (*C*(1)=O), 172.0 (*C*(4)=O).

3.2 (2*R*,3*S*)-3-(((*tert*-Butyldiphenylsilyl)oxy)methyl)-2-(dimethylamino)pent-4-enoic acid, 4-nitrophenyl ester ($\mathbf{20_{maj}}$) and (2*R*,3*R*)-3-(((*tert*-butyldiphenylsilyl)oxy)methyl)-2-(dimethylamino)pent-4-enoic acid, 4-nitrophenyl ester ($\mathbf{20_{min}}$)

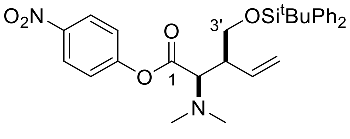

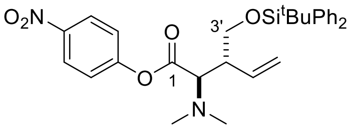

$C_{30}H_{36}N_2O_5Si$
532.71

Following General Procedure 1.5, PNP ester **13** (65.0 mg, 0.25 mmol, 1.0 eq), FurCat **3** (9.3 mg, 12.5 μmol, 5 mol%), (±)-TM·HCl (12.0 mg, 0.05 mmol, 10 mol%), phosphate **16** (143 mg, 0.31 mmol, 1.25 eq) and *i*Pr_2NEt (0.1 mL, 0.6 mmol, 2.4 eq) in MeCN (4.4 mL) gave crude product **20** (55:45 dr), which was purified by flash silica chromatography (Petrol : EtOAc 6:1 to 4:1) to give:

Major diastereoisomer $\mathbf{20_{maj}}$ (R_f 0.42 in Petrol : EtOAc 4:1, 24 mg, 18%) as a colourless solid.

^{1}H NMR (500 MHz, $CDCl_3$) δ_H: 1.05 (9H, s, C(C*H*$_3$)$_3$), 2.45 (6H, s, N(C*H*$_3$)$_2$), 2.82 (1H, ddd, *J* 14.3, 8.8, 4.0, C(3)*H*), 3.69 – 3.81 (2H, m, C*H*$_2$-OSi), 3.82 (1H, d, *J* 10.9, C(2)*H*), 5.16 – 5.28 (2H, m, C(5)*H*$_2$), 5.99 (1H, dt, *J* 17.2, 9.8, C(4)*H*), 6.99 – 7.05 (2H, m, Ar(2,6)*H*), 7.30 – 7.46 (6H, m, 2 × Ph(2,4,6)*H*), 7.59 – 7.66 (4H, m, 2 × Ph(3,5)*H*), 8.15 – 8.21 (2H, m, Ar(3,5)*H*).

^{13}C{^{1}H} NMR (126 MHz, $CDCl_3$) δ_C: 19.3 (*C*(CH$_3$)$_3$), 26.8 (C(*C*H$_3$)$_3$), 41.5 (N(*C*H$_3$)$_2$), 46.3 (*C*(3)H), 65.6 (*C*H$_2$-OSi), 67.5 (*C*(2)H), 117.2 (*C*(5)H$_2$), 122.6 (Ar*C*(2,6)H), 125.1 (Ar*C*(3,5)H), 127.7 (2 × Ph*C*(2,6)H), 129.8 (2 × Ph*C*(4)H), 133.2 (Ph*C*(1)), 135.6 (2 × Ph*C*(3,5)H), 136.9 (*C*(4)H), 145.2 (Ar*C*(1)), 155.0 (Ar*C*(4)), 168.1 (*C*=O).

Minor diastereoisomer $\mathbf{20_{min}}$ (R_f 0.59 in Petrol : EtOAc 4:1, 23 mg, 17%) as an off-white solid.

^{1}H NMR (400 MHz, $CDCl_3$) δ_H: 1.09 (9H, s, C(C*H*$_3$)$_3$), 2.43 (6H, s, N(C*H*$_3$)$_2$), 2.72 – 2.80 (1H, m, C(3)*H*), 3.72 – 3.79 (2H, m, C(2)*H*, C*H*^{A}H^B-OSi), 4.00 (1H, dd, *J* 9.7, 4.6, CH^A*H*^{B}-OSi), 5.24 – 5.32 (2H, m, C(5)*H*$_2$), 6.09 (1H, ddd, *J* 16.7, 10.8, 9.0, C(4)*H*), 7.23 – 7.27 (2H, m, Ar(2,6)*H*), 7.38 – 7.47 (6H, m, 2 × Ph(2,4,6)*H*), 7.70 – 7.79 (3H, m, 2 × Ph(3,5)*H*), 8.26 – 8.31 (2H, m, Ar(3,5)*H*).

^{13}C{^{1}H} NMR (126 MHz, $CDCl_3$) δ_C: 19.3 (*C*(CH$_3$)$_3$), 26.8 (C(*C*H$_3$)$_3$), 41.4 (N(*C*H$_3$)$_2$), 45.4 (*C*(3)H), 63.2 (*C*H$_2$-OSi), 67.0 (*C*(2)H), 118.7 (*C*(5)H$_2$), 122.7 (Ar*C*(2,6)H), 125.2 (Ar*C*(3,5)H), 127.6 (2 × Ph*C*(2,6)H), 129.6 (2 × Ph*C*(4)H), 133.0 (Ph*C*(1)), 135.6 (2 × Ph*C*(3,5)H), 136.4 (*C*(4)H), 145.3 (Ar*C*(1)), 155.2 (Ar*C*(4)), 168.5 (*C*=O).

3.3 (2*R*,3*S*)-2-(Dimethylamino)-3-(methoxy(methyl)carbamoyl)pent-4-enoic acid, 4-nitrophenyl ester (27maj) and (2*R*,3*R*)-2-(Dimethylamino)-3-(methoxy(methyl)carbamoyl)pent-4-enoic acid, 4-nitrophenyl ester (27min)

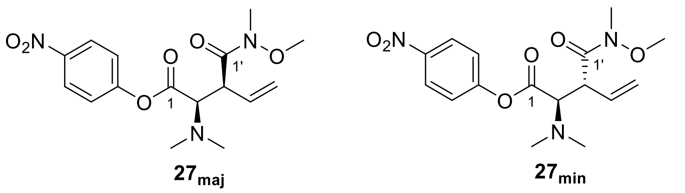

$C_{16}H_{21}N_3O_6$
351.36

Following General Procedure 1.5, PNP ester **13** (65.0 mg, 0.25 mmol, 1.0 eq), $Pd_2dba_3{\cdot}CHCl_3$ (6.5 mg, 6.25 μmol, 2.5 mol%), P(2-furyl)$_3$ (5.8 mg, 25 μmol, 10 mol%), (*S*)-TM·HCl (12.0 mg, 0.05 mmol, 20 mol%), phosphate **26** (77.4 mg, 0.27 mmol, 1.1 eq) and *i*Pr$_2$NEt (0.1 mL, 0.6 mmol, 2.4 eq) in MeCN (4.4 mL) gave the crude product (60:40 dr), which was purified by silica column chromatography (CH_2Cl_2 : Et_2O 15:1 to 4:1) to give:

Major diastereoisomer **27maj** *(R_f* 0.37 in CH_2Cl_2 : Et_2O 4:1) as pale yellow glass (30 mg, 34%).

$[\alpha]_D^{20}$ + 0.2 (*c* 0.5 in $CHCl_3$); **Chiral HPLC** analysis**,** Chiralcel OD-H, (*n*-hexane : *i*PrOH 95:5, flow rate 1.0 mLmin^{-1}, 211 nm, 40 °C) t_R (2*R*,3*S*) 19.5 min, t_R (2*S*,3*R*) 14.7 min, 91:9 er.

^{1}H NMR (500 MHz, $CDCl_3$) δ_H: 2.47 (6H, s, N(C*H*$_3$)$_2$), 3.25 (3H, s, C(O)NC*H*$_3$), 3.75 (3H, s, OC*H*$_3$), 4.01 – 4.07 (m, 1H, C(2)*H*), 4.17 – 4.30 (1H, m, C(3)*H*), 5.30 (1H, d, *J* 10.2, C(5)H^AH^B), 5.36 (1H, d, *J* 17.2, C(5)H^AH^B), 5.87 – 5.96 (1H, m, C(4)*H*), 7.23 – 7.26 (2H, m, Ar(2,6)*H*), 8.24 – 8.29 (2H, m, Ar(3,5)*H*).

^{13}C{^{1}H} NMR (126 MHz, $CDCl_3$) δ_C: 32.4 (C(O)NCH$_3$), 42.2 (N(CH$_3$)$_2$), 46.1 (*C*(3)H), 61.9 (OCH$_3$), 69.0 (*C*(2)H), 120.7 (*C*(5)H$_2$), 122.8 (Ar*C*(2,6)H), 125.3 (Ar*C*(3,5)H), 133.3 (*C*(4)H), 145.5 (Ar*C*(1)), 155.1 (Ar*C*(4)), 167.5 (*C*(1)=O), 171.6 (*C*(1')=O).

HRMS (NSI$^+$)$C_{16}H_{22}N_3O_6$ $[M+H]^+$ found 352.1506, requires 352.1503 (+0.8 ppm).

ν_{max} ($CDCl_3$, cm^{-1}) 2941 (C-H), 1755 (C=O), 1654 (C=O), 1593 (C=C$_{Ar}$), 1525 (N=O), 1489 (C=C$_{Ar}$), 1346 (N=O).

Minor diastereoisomer **27min** *(R_f* 0.61 in CH_2Cl_2 : Et_2O 4:1) as pale yellow glass (20 mg, 23%).

$[\alpha]_D^{20}$ +15.8 (*c* 0.25 in $CHCl_3$); **Chiral HPLC** analysis**,** Chiralcel OD-H, (*n*-hexane : *i*PrOH 95:5, flow rate 1.0 mLmin^{-1}, 211 nm, 40 °C) t_R (2*R*,3*R*): 10.1 min, t_R (2*S*,3*S*): 13.3 min, 83:17 er.

^{1}H NMR (500 MHz, $CDCl_3$) δ_H: 2.50 (6H, s, N(C*H*$_3$)$_2$), 3.18 (3H, s, C(O)NC*H*$_3$), 3.75 (3H, s, OC*H*$_3$), 3.99 – 4.04 (1H, m, C(2)*H*), 4.04 – 4.12 (1H, m, C(3)*H*), 5.24 – 5.34 (2H, m, C(5)*H*$_2$), 5.83 – 5.93 (1H, m, C(4)*H*), 7.26 – 7.31 (2H, m, Ar(2,6)*H*), 8.23 – 8.28 (2H, m, Ar(3,5)*H*).

$^{13}C\{^1H\}$ NMR (126 MHz, $CDCl_3$) δ_C: 32.3 (C(O)NCH_3), 42.1 (N$(CH_3)_2$), 45.8 (*C*(3)H), 61.3 (OCH_3), 68.3 (*C*(2)H), 118.9 (*C*(5)H_2), 122.8 (Ar*C*(2,6)H), 125.3 (Ar*C*(3,5)H), 133.5 (*C*(4)H), 145.5 (Ar*C*(1)), 155.3 (Ar*C*(4)), 168.8 (*C*(1)=O), 172.3 (*C*(1′)=O).

HRMS (NSI^+) $C_{16}H_{22}N_3O_6$ $[M+H]^+$ found 352.1505, requires 352.1503 (+0.5 ppm).

ν_{max} ($CHCl_3$, cm^{-1}) 2941 (C-H), 1749 (C=O), 1651 (C=O), 1593 (C=C_{Ar}), 1525 (N=O), 1489 (C=C_{Ar}), 1346 (N=O).

3.4 (*S*)-2-((*R*)-1-(dimethylamino)-2-oxo-2-(pyrrolidin-1-yl)ethyl)-*N*-methoxy-*N*-methylbut-3-enamide (28_{maj})

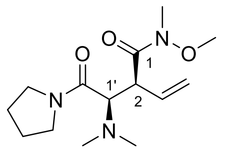

$C_{14}H_{25}N_3O_3$
283.37
28_{maj}

A solution of PNP ester **27_{maj}** (57.0 mg, 0.17 mmol, 1.0 eq) and pyrrolidine (43 μL, 0.51 mmol, 3.0 eq) in CH_2Cl_2 (3.5 mL) was stirred at room temperature overnight. The reaction was quenched by the addition of 1 M NaOH (3 mL), the phases separated and the aqueous phase extracted with CH_2Cl_2 (3 × 3 mL). The combined organic phases were washed with 1 M NaOH (2 × 5 mL), water (5 mL) and brine (5 mL), dried over $MgSO_4$, filtered and the solvent removed under reduced pressure. The crude product was purified by silica column chromatography (CH_2Cl_2 : 2% MeOH to CH_2Cl_2 : 4% MeOH, R_f 0.47 in CH_2Cl_2 : MeOH 9:1) to yield the title compound **28_{maj}** as white solid (13 mg, 27%). **m.p.** (CH_2Cl_2) 115 – 118 °C.

$[\alpha]_D^{20}$ −7.0 (*c* 0.3 in $CHCl_3$); **Chiral HPLC** analysis**,** Chiralpak ID, (*n*-hexane : *i*PrOH 88:12, flow rate 1.5 mLmin^{-1}, 211 nm, 40 °C) t_R (2*S*,1′*R*): 20.0 min, t_R (2*R*,1′*S*) 13.9 min, 90:10 er.

^1H NMR (500 MHz, $CDCl_3$) δ_H: 1.76 - 1.86 (2H, m, C$H_{2(pyrr)}$), 1.86 – 1.97 (2H, m, C$H_{2(pyrr)}$), 2.37 (6H, s, N(CH_3)$_2$), 3.22 (3H, s, C(O)NCH_3), 3.45 (2H, td, *J* 7.0, 2.7, NC$H_{2(pyrr)}$), 3.52 (2H, td, *J* 6.8, 1.8, NC$H_{2(pyrr)}$), 3.71 (3H, s, OCH_3), 3.96 (1H, d, *J* 10.9, C(1′)*H*), 4.18 – 4.32 (1H, m, C(2)*H*), 5.12 (1H, dd, *J* 10.3, 1.1, C(4)H^AH^B), 5.22 – 5.29 (1H, m, C(4)H^AH^B), 5.70 – 5.80 (1H, m, C(3)*H*).

$^{13}C\{^1H\}$ NMR (126 MHz, $CDCl_3$) δ_C: 24.4 (*C*$H_{2(pyrr)}$), 26.3 (*C*$H_{2(pyrr)}$), 32.4 (C(O)NCH_3), 42.2 (N$(CH_3)_2$), 45.2 (N$CH_{2(pyrr)}$), 45.6 (*C*(2)H), 47.1 (N*C*$H_{2(pyrr)}$), 61.9 (OCH_3), 66.1 (*C*(1′)H), 119.5 (*C*(4)H_2), 133.7 (*C*(3)H), 168.1 (*C*(2′)=O), 173.0 (*C*(1)=O).

HRMS (NSI^+) $C_{14}H_{26}N_3O_3$ $[M+H]^+$ found 284.1971, requires 284.1969 (+0.8 ppm).

ν_{max} ($CHCl_3$, cm^{-1}) 2972 (C-H), 2875 (C-H), 1625 (C=O).

3.5 (*R*)-2-((*R*)-1-(dimethylamino)-2-oxo-2-(pyrrolidin-1-yl)ethyl)-*N*-methoxy-*N*-methylbut-3-enamide (**28$_{min}$**)

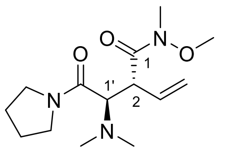

$C_{14}H_{25}N_3O_3$
283.37
28$_{min}$

A solution of PNP ester **27$_{min}$** (57.0 mg, 0.17 mmol, 1.0 eq) and pyrrolidine (43 μL, 0.51 mmol, 3.0 eq) in CH_2Cl_2 (3.5 mL) was stirred at room temperature overnight. The reaction was quenched by the addition of 1 M NaOH (3 mL), the phases separated and the aqueous phase extracted with CH_2Cl_2 (3 × 3 mL). The combined organic phases were washed with 1 M NaOH (2 × 5 mL), water (5 mL) and brine (5 mL), dried over $MgSO_4$, filtered and the solvent removed under reduced pressure. The crude product was purified by silica column chromatography (CH_2Cl_2 : 2% MeOH to CH_2Cl_2 : 4% MeOH, R_f 0.55 in CH_2Cl_2 : MeOH 9:1) to yield the title compound **28$_{min}$** as colourless glass (13 mg, 27%).

$[\boldsymbol{\alpha}]_D^{20}$ + 9.8 (*c* 0.2 in $CHCl_3$); **Chiral HPLC** analysis, Chiralpak ID, (*n*-hexane : *i*PrOH 88:12, flow rate 1.5 mLmin^{-1}, 211 nm, 40 °C) t_R (major) 12.5 min, t_R (minor) 6.2 min, 84:16 er.

^{1}H NMR (400 MHz, $CDCl_3$) δ_H: 1.75 – 1.88 (2H, m, C$H_{2(pyrr)}$), 1.88 – 1.96 (2H, m, C$H_{2(pyrr)}$), 2.42 (6H, s, N(CH_3)$_2$), 3.15 (3H, s, C(O)NCH_3), 3.36 – 3.48 (2H, m, NC$H_{2(pyrr)}$), 3.55 (1H, dt, *J* 10.0, 7.0, N(CH^AH^B)$_{(pyrr)}$), 3.74 (1H, dt, *J* 10.0, 6.6, N(CH^AH^B)$_{(pyrr)}$), 3.81 (3H, s, OCH_3), 3.94 (1H, d, *J* 10.8, C(1′)*H*), 4.21 – 4.35 (1H, m, C(2)*H*), 5.23 (1H, dd, *J* 10.2, 1.2, C(4)H^AH^B), 5.29 (1H, d, *J* 17.3, C(4)H^AH^B), 5.89 (1H, ddd, *J* 17.2, 10.2, 8.8, C(3)*H*).

^{13}C{^{1}H} NMR (126 MHz, $CDCl_3$) δ_C: 24.4 ($CH_{2(pyrr)}$), 26.2 ($CH_{2(pyrr)}$), 32.2 (C(O)NCH_3), 41.9 (N(CH_3)$_2$), 45.5 (N$CH_{2(pyrr)}$), 45.9 (*C*(2)H), 46.8 (N$CH_{2(pyrr)}$), 61.5 (OCH_3), 65.6 (*C*(1′)H), 118.6 (*C*(4)H_2), 135.1 (*C*(3)H), 168.7 (*C*(2′)=O), 172.6 (*C*(1)=O).

HRMS (NSI$^+$) $C_{14}H_{26}N_3O_3$ [M+H]$^+$ found 284.1972, requires 284.1969 (+1.2 ppm).

ν_{max} (film, cm^{-1}) 2976 (C-H), 1647 (C=O), 1629 (C=O).

3.6 (2*R*,3*S*)-2-(Dimethylamino)-3-(methoxy(methyl)carbamoyl)pent-4-enoic acid, benzyl ester ($\mathbf{29_{maj}}$) and (2*R*,3*R*)-2-(Dimethylamino)-3-(methoxy(methyl) carbamoyl)pent-4-enoic acid, benzyl ester ($\mathbf{29_{min}}$)

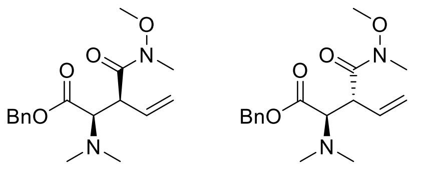

$\mathbf{29_{maj}}$ $\mathbf{29_{min}}$

$C_{17}H_{24}N_2O_4$
320.39

Following General Procedure 1.5, PNP ester 1**3** (78.2 mg, 0.30 mmol, 1.0 eq), $Pd_2dba_3{\cdot}CHCl_3$ (7.7 mg, 7.5 µmol, 2.5 mol%), P(2-furyl)$_3$ (6.9 mg, 30 µmol, 10 mol%), (*S*)-TM·HCl (14.4 mg, 0.06 mmol, 20 mol%), phosphate **26** (105 mg, 0.37 mmol, 1.25 eq) and *i*Pr_2NEt (125 µL, 0.7 mmol, 2.4 eq) in MeCN (5.0 mL) gave the crude product (63:37 dr), which was used directly for derivatisation with NaOBn (0.45 mL, 0.45 mmol, 1.5 eq) in THF (6.0 mL). Subsequent purification of the crude derivatised product via silica column chromatography (petrol : EtOAc 4:1 to EtOAc) gave:

Major diastereoisomer $\mathbf{29_{maj}}$ (R_f 0.12 in petrol : EtOAc 1:1) as yellow oil (33 mg, 34%).

$[\alpha]_D^{20}$ −1.6 (*c* 0.9 in $CHCl_3$); **chiral HPLC analysis,** Chiralcel OD-H (95:5 hexane : *i*PrOH, flow rate 1 mlmin^{-1}, 211 nm, 40 °C) t_R (2*R*,3*S*): 8.3 min, t_R (2*S*,3*R*): 9.8 min, 87:13 er.

^{1}H NMR (500 MHz, $CDCl_3$) δ_H: 2.31 (6H, s, N(C*H*$_3$)$_2$), 3.20 (3H, s, C(O)NC*H*$_3$), 3.70 (3H, s, OCH$_3$), 3.80 (1H, d, *J* 11.4, C(2)*H*), 4.10 – 4.19 (1H, m, C(3)*H*), 5.10 (1H, dd, *J* 10.1, 1.3, C(5)H^AH^B), 5.10 (1H, d, *J* 12.2, OCH^AH^B), 5.14 (1H, d, *J* 12.2, OCH^AH^B), 5.21 (1H, dt, *J* 17.2, 0.8, C(5)H^AH^B), 5.76 (1H, ddd, *J* 17.2, 10.2, 8.8, C(4)*H*), 7.29 – 7.37 (5H, m, 5 × Ar*H*).

^{13}C{^{1}H} NMR (126 MHz, $CDCl_3$) δ_C: 32.2 (C(O)NCH$_3$), 42.0 (N(CH$_3$)$_2$), 45.9 (*C*(3)H), 61.7 (OCH$_3$), 65.8 (OCH$_2$), 68.9 (*C*(2)H), 119.9 (*C*(5)H$_2$), 128.2 (ArCH), 128.5 (ArCH), 128.5 (ArCH), 133.3 (*C*(4)H), 135.8 (ArC), 169.4 (C=O$_{ester}$), 172.1 (C=O$_{amide}$).

HRMS (ESI$^+$) $C_{17}H_{25}N_2O_4$ [M+H]$^+$ found 321.1805 requires 321.1809 (−1.2 ppm).

ν_{max} ($CHCl_3$, cm^{-1}) 3008 (=CH), 2943 (C-H), 2792 (N(C-H)), 1724 (C=O$_{ester}$), 1654 (C=O$_{amide}$), 1635 (C=C).

Minor diastereoisomer $\mathbf{29_{min}}$ (R_f 0.33 in petrol : EtOAc 1:1) as colourless glass (21 mg, 22%).

$[\alpha]_D^{20}$ +8.5 (*c* 1.4 in $CHCl_3$); **chiral HPLC analysis**, Chiralcel OD-H (99:1 hexane : *i*PrOH, flow rate 1 mlmin^{-1}, 211 nm, 40 °C) t_R (2*R*,3*R*): 16.3 min, t_R (2*S*,3*S*): 19.0 min, 77:23 er.

^{1}H NMR (500 MHz, $CDCl_3$) δ_H: 2.33 (6H, s, N(C*H*$_3$)$_2$), 3.12 (3H, s, C(O)NC*H*$_3$), 3.69 (3H, s, OC*H*$_3$), 3.83 (1H, d, *J* 11.3, C(2)*H*), 3.98 – 4.07 (1H, m, C(3)*H*), 5.05 (1H, d, *J* 12.3, OCH^AH^B),

5.20 – 5.26 (3H, m, OCH^{A}*H*^{B}, C(5)*H*$_2$), 5.82 (1H, ddd, *J* 17.0, 10.5, 8.4, C(4)*H*), 7.28 – 7.38 (5H, m, 5 × Ar*H*).

^{13}C{^{1}H} NMR (126 MHz, CDCl$_3$) δ$_C$: 32.1 (C(O)NCH$_3$), 41.9 (N(CH$_3$)$_2$), 45.6 (*C*(3)H), 61.0 (OCH$_3$), 66.1 (OCH$_2$), 68.0 (*C*(2)H), 118.2 (*C*(5)H$_2$), 128.2 (ArCH), 128.4 (ArCH), 128.5 (ArCH), 134.1 (*C*(4)H), 135.9 (Ar*C*), 170.5 (C=O$_{ester}$), 172.4 (C=O$_{amide}$).

HRMS (ESI$^+$) $C_{17}H_{24}N_2O_4Na$ [M+Na]$^+$ found 343.1616, requires 343.1628 (−3.6 ppm).

ν$_{max}$ (CHCl$_3$, cm^{-1}) 3016 (=CH), 2943 (C-H), 2789 (N(C-H)), 1720 (C=O$_{ester}$), 1651 (C=O$_{amide}$), 1631 (C=C).

3.7 (*E*)-3-(Dimethylamino)-4-ethylidene-1-phenylpyrrolidine-2,5-dione ((*E*)-37) and (*Z*)-3-(Dimethylamino)-4-ethylidene-1-phenylpyrrolidine-2,5-dione ((*Z*)-37)

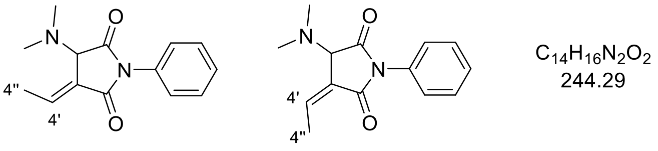

(*E*)-37 (*Z*)-37

Following General Procedure 1.5, PNP ester **13** (78.2 mg, 0.30 mmol, 1.0 eq), Pd$_2$dba$_3$·CHCl$_3$ (7.7 mg, 7.5 μmol, 2.5 mol%), P(2-furyl)$_3$ (6.9 mg, 30 μmol, 10 mol%), (*S*)-TM·HCl (14.4 mg, 0.06 mmol, 20 mol%), phosphate **32** (118 mg, 0.37 mmol, 1.25 eq) and *i*Pr$_2$NEt (125 μL, 0.7 mmol, 2.4 eq) in MeCN (5.0 mL) gave the crude product (80:20 (*E*):(*Z*)). Purification via silica column chromatography (pentane : EtOAc 4:1 to 1:1) gave:

(*E*)-37 (R$_f$ 0.36 in pentane : EtOAc 2:1) as colourless solid (23 mg, 32%).

$[\alpha]_D^{20}$ +1.7 (*c* 1.15 in CHCl$_3$); **chiral HPLC analysis,** Chiralcel OD-H (98:2 hexane : *i*PrOH, flow rate 1 mlmin^{-1}, 254 nm, 30 °C) t$_R$ (minor): 18.0 min, t$_R$ (major): 21.0 min, 45:55 er.

^{1}H NMR (500 MHz, CDCl$_3$) δ$_H$: 2.07 (3H, dd, *J* 7.3, 1.4, C(4'')*H*$_3$), 2.52 (6H, s, N(C*H*$_3$)$_2$), 4.22 – 4.26 (1H, m, C(3)*H*), 7.21 (1H, qd, *J* 7.3, 2.2, C(4')*H*), 7.28 – 7.32 (2H, m, Ar(2,6)*H*), 7.36 – 7.40 (1H, m, Ar(4)*H*), 7.44 – 7.49 (2H, m, Ar(3,5)*H*).

^{13}C{^{1}H} NMR (126 MHz, CDCl$_3$) δ$_C$: 14.9 (*C*(4'')H$_3$), 40.9 (N(*C*H$_3$)$_2$), 63.2 (*C*(3)H), 126.5 (Ar*C*(2,6)H), 128.1 (*C*(4)), 128.5 (Ar*C*(4)H), 129.0 (Ar*C*(3,5)H), 131.6 (Ar*C*(1)), 140.4 (*C*(4')H), 168.0 (*C*(5)=O), 174.1 (*C*(2)=O).

HRMS (ESI$^+$) $C_{14}H_{17}N_2O_2$ [M+H]$^+$ found 245.1276, requires 245.1284 (−3.4 ppm).

ν$_{max}$ (CHCl$_3$, cm^{-1}) 2939 (C-H), 1770, 1707 (C=O), 1674, 1597 (C=C$_{Ar}$), 1494 (C=C$_{Ar}$).

***(Z)*-37** (R_f 0.26 in pentane : EtOAc 1:1) as colourless oil (3 mg, 4%).

$[\alpha]_D^{20}$ −0.6 (*c* 0.15 in $CHCl_3$); **chiral HPLC analysis,** Chiralcel OD-H (99:1 hexane : *i*PrOH, flow rate 1 mlmin^{-1}, 254 nm, 30 °C) $t_{R,1}$: 20.1 min, $t_{R,2}$: 23.5 min, 50:50 er.

^{1}H NMR (500 MHz, $CDCl_3$) δ_H: 2.38 (3H, dd, *J* 7.5, 1.9, C(4'')H_3), 2.54 (6H, s, N(CH_3)$_2$), 4.10 – 4.13 (1H, m, C(3)*H*), 6.64 (1H, qd, *J* 7.4, 1.9, C(4')*H*), 7.27 – 7.31 (2H, m, Ar(2,6)*H*), 7.36 – 7.41 (1H, m, Ar(4)*H*), 7.45 – 7.49 (2H, m, Ar(3,5)*H*).

^{13}C{^{1}H} NMR (126 MHz, $CDCl_3$) δ_C: 15.1 (*C*(4'')H_3), 41.1 (N(CH_3)$_2$), 65.3 (*C*(3)H), 126.6 (*C*(4)), 126.6 (Ar*C*(2,6)H), 128.5 (Ar*C*(4)H), 129.0 (Ar*C*(3,5)H), 131.5 (Ar*C*(1)), 142.8 (*C*(4')H), 167.7 (*C*(5)=O), 173.8 (*C*(2)=O).

HRMS (ESI$^+$) $C_{14}H_{17}N_2O_2$ [M+H]$^+$ found 245.1277, requires 245.1284 (−3.1 ppm).

ν_{max} ($CHCl_3$, cm^{-1}) 2937 (C-H), 1766, 1705 (C=O), 1674, 1597 (C=C_{Ar}), 1496 (C=C_{Ar}).

3.8 (2*R*,3*S*)-2-(Dimethylamino)-3-(methyl(phenyl)carbamoyl)pent-4-enoic acid, benzyl ester (38$_{maj}$) and (2*R*,3*R*)-2-(Dimethylamino)-3-(methyl(phenyl) carbamoyl)pent-4-enoic acid, benzyl ester (38$_{min}$)

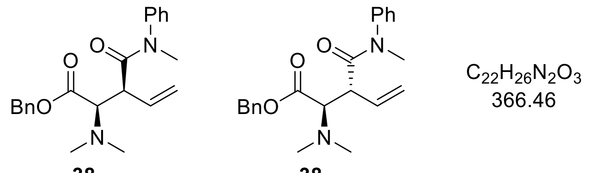

38$_{maj}$ **38$_{min}$**

Following General Procedure 1.5, PNP ester **13** (78.2 mg, 0.30 mmol, 1.0 eq), $Pd_2dba_3\cdot CHCl_3$ (7.7 mg, 7.5 μmol, 2.5 mol%), P(2-furyl)$_3$ (6.9 mg, 30 μmol, 10 mol%), (*S*)-TM·HCl (14.4 mg, 0.06 mmol, 20 mol%), phosphate **33** (122 mg, 0.37 mmol, 1.25 eq) and *i*Pr_2NEt (125 μL, 0.7 mmol, 2.4 eq) in MeCN (5.0 mL) gave the crude product (57:43 dr), which was used directly for derivatisation with NaOBn (0.45 mL, 0.45 mmol, 1.5 eq) in THF (6.0 mL). Subsequent purification of the crude derivatised product via silica column chromatography (CH_2Cl_2 : Et_2O 9:1 to 4:1) gave:

Major diastereoisomer **38$_{maj}$** (R_f 0.28 in CH_2Cl_2 : Et_2O 4:1) as yellow glass (19 mg, 17%).

$[\alpha]_D^{20}$ +1.8 (c 0.85 in $CHCl_3$); **chiral HPLC** analysis, Chiralpak AD-H (98.2:1.8 hexane : *i*PrOH, flow rate 1 mlmin^{-1}, 211 nm, 30 °C) t_R (2*R*,3*S*): 26.8 min, t_R (2*S*,3*R*): 31.4 min, 66:34 er.

^{1}H NMR (500 MHz, $CDCl_3$) δ_H: 2.19 (6H, s, N(CH_3)$_2$), 3.27 (3H, s, C(O)NCH_3), 3.40 (1H, dd, *J* 11.1, 8.8, C(3)*H*), 3.86 (1H, d, *J* 11.2, C(2)*H*), 4.94 (1H, d, *J* 17.2, C(5)H^AH^B), 5.00 (1H, d, *J* 12.4,

OC$H^A$$H^B$), 5.03 – 5.08 (2H, m, OC$H^A$$H^B$, C(5)$H^A$$H^B$), 5.69 (1H, ddd, *J* 17.3, 10.2, 8.9, C(4)*H*), 7.19 (2H, m, 2 × NAr*H*), 7.26 – 7.38 (6H, m, NAr*H*, 5 × OAr*H*), 7.38 – 7.43 (2H, m, 2 × NAr*H*).

^{13}C{^{1}H} NMR (126 MHz, $CDCl_3$) δ_C: 37.7 (C(O)NCH_3), 42.3 (N(CH_3)$_2$), 47.8 (*C*(3)H), 65.6 (OCH_2), 69.4 (*C*(2)H), 119.8 (*C*(5)H_2), 127.7 (NArCH), 128.0 (ArCH), 128.1 (ArCH), 128.3 (ArCH), 128.4 (ArCH), 129.5 (NArCH), 133.5 (*C*(4)H), 135.9 (OAr*C*(1)), 143.4 (NAr*C*(1)), 169.2 (*C*=O_{ester}), 170.8 (*C*=O_{amide}).

HRMS (ESI$^+$) $C_{22}H_{26}N_2O_3Na$ [M+Na]$^+$ found 389.1825, requires 389.1836 (−2.7 ppm).

ν_{max} ($CHCl_3$, cm^{-1}) 3012 (=CH), 2943 (C-H), 2789 (N(C-H)), 1728 (C=O_{ester}), 1651 (C=O_{amide}), 1635 (C=C), 1597 (C=C_{Ar}), 1496 (C=C_{Ar}).

Minor diastereoisomer **38$_{min}$** (R_f 0.42 in CH_2Cl_2 : Et_2O 4:1) as yellow glass (18 mg, 17%).

$[\alpha]_D^{20}$ +6.9 (*c* 0.6 in $CHCl_3$); **chiral HPLC** analysis, Chiralcel OD-H (99.5:0.5 hexane : *i*PrOH, flow rate 1 mlmin^{-1}, 211 nm, 30 °C) t_R (2*R*,3*R*): 33.1 min, t_R (2*S*,3*S*): 38.4 min, 67:33 er.

^{1}H NMR (500 MHz, $CDCl_3$) δ_H: 2.18 (6H, s, N(C*H*$_3$)$_2$), 3.18 (3H, s, C(O)NC*H*$_3$), 3.51 (1H, dd, *J* 10.9, 8.7, C(3)*H*), 3.85 (1H, d, *J* 10.9, C(2)*H*), 4.64 (1H, dt, *J* 17.2, 0.9, C(5)$H^A$$H^B$), 5.06 (1H, dd, *J* 10.2, 1.1, C(5)$H^A$$H^B$), 5.13 (1H, d, *J* 12.3, OC$H^A$$H^B$), 5.25 (1H, d, *J* 12.3, OC$H^A$$H^B$), 5.66 (1H, ddd, *J* 17.3, 10.1, 8.8), 7.18 – 7.23 (2H, m, 2 × NAr*H*), 7.30 – 7.43 (8H, m, 3 × NAr*H*, 5 × OAr*H*).

^{13}C{^{1}H} NMR (126 MHz, $CDCl_3$) δ_C: 37.5 (C(O)NCH_3), 41.9 (N(CH_3)$_2$), 47.2 (*C*(3)H), 66.1 (OCH_2), 68.8 (*C*(2)H), 118.2 (*C*(5)H_2), 127.8 (ArCH), 128.1 (ArCH), 128.2 (ArCH), 128.5 (ArCH), 128.5 (ArCH), 129.3 (ArCH), 134.3 (*C*(4)H), 136.0 (OAr*C*(1)), 143.4 (NArC(1)), 170.4 (*C*=O_{ester}), 171.1 (*C*=O_{amide}).

HRMS (ESI$^+$) $C_{22}H_{26}N_2O_3Na$ [M+Na]$^+$ found 389.1822, requires 389.1836 (−3.5 ppm).

ν_{max} ($CHCl_3$, cm^{-1}) 3012 (=CH), 2943 (C-H), 2789 (N(C-H)), 1724 (C=O_{ester}), 1647 (C=O_{amide}), 1631 (C=C), 1593 (C=C_{Ar}), 1496 (C=C_{Ar}).

3.9 (2*R*,3*S*)-3-(Benzyl(methyl)carbamoyl)-2-(dimethylamino)pent-4-enoic acid, benzyl ester (39$_{maj}$) and (2*R*,3*R*)-3-(Benzyl(methyl)carbamoyl)-2-(dimethylamino)pent-4-enoic acid, benzyl ester (39$_{min}$)

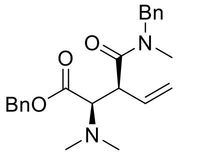

39$_{maj}$

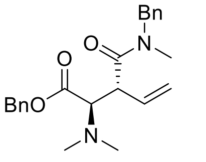

39$_{min}$

$C_{23}H_{28}N_2O_3$
380.49

Following General Procedure 1.5, PNP ester **13** (78.2 mg, 0.30 mmol, 1.0 eq), $Pd_2dba_3 \cdot CHCl_3$ (7.7 mg, 7.5 μmol, 2.5 mol%), $P(2\text{-furyl})_3$ (6.9 mg, 30 μmol, 10 mol%), (*S*)-TM·HCl (14.4 mg, 0.06 mmol, 20 mol%), phosphate **34** (205 mg, 0.6 mmol, 2.0 eq) and *i*Pr_2NEt (125 μL, 0.7 mmol, 2.4 eq) in MeCN (5.0 mL) gave the crude product (54:46 dr), which was used directly for derivatisation with NaOBn (0.45 mL, 0.45 mmol, 1.5 eq) in THF (6.0 mL). Subsequent purification of the crude derivatised product via silica column chromatography (petrol : EtOAc 3:1 to 1:1) gave:

Major diastereoisomer $\mathbf{39_{maj}}$ (R_f 0.18 in petrol : EtOAc 1:1) as yellow oil (36 mg, 32%) as a rotameric mixture (3:2).

$[\boldsymbol{\alpha}]_D^{20}$ −0.8 (*c* 0.5 in $CHCl_3$); **chiral HPLC** analysis, Chiralcel OD-H (98:2 hexane : *i*PrOH, flow rate 1 mlmin^{-1}, 211 nm, 40 °C) t_R (2*R*,3*S*): 22.4 min, t_R (2*S*,3*R*): 29.9 min, 71:29 er.

^{1}H NMR (500 MHz, $CDCl_3$) *major rotamer* δ_H: 2.35 (6H, s, N(C*H*$_3$)$_2$), 2.97 (3H, s, C(O)NC*H*$_3$), 3.89 (1H, dd, *J* 10.9, 8.5, C(3)*H*), 3.95 (1H, d, *J* 11.0, C(2)*H*), 4.54 (1H, d, *J* 14.9, NC*H*^{A}H^B), 4.75 (d, *J* 14.9, NCH^A*H*^{B}), 5.09 – 5.15 (2H, m, OC*H*^{A}H^B, C(5)*H*^{A}H^B), 5.17 (1H, d, *J* 12.3, OCH^A*H*^{B}), 5.22 (1H, d, *J* 17.2, C(5)H^A*H*^{B}), 5.78 (1H, ddd, *J* 17.3, 10.2, 8.5, C(4)*H*), 7.18 – 7.26 (3H, m, 3 × NBn*H*), 7.27 – 7.39 (7H, m, 2 × NBn*H*, 5 × OBn*H*); *minor rotamer (selected)* δ_H: 2.23 (6H, s, N(C*H*$_3$)$_2$), 3.75 (1H, dd, *J* 10.8, 8.6, C(3)*H*), 3.96 (1H, d, *J* 10.9, C(2)*H*), 4.49 (1H, d, *J* 16.8, NC*H*^{A}H^B), 4.68 (1H, d, *J* 16.8, NCH^A*H*^{B}), 5.07 (1H, d, *J* 12.3, OC*H*^{A}H^B), 5.76 (1H, ddd, J 17.3, 10.3, 8.6, C(4)*H*).

^{13}C{^{1}H} NMR (126 MHz, $CDCl_3$) *major rotamer* δ_C: 34.8 (C(O)N*C*H$_3$), 42.2 (N(*C*H$_3$)$_2$), 46.9 (*C*(3)H), 51.1 (NCH$_2$), 65.9 (OCH$_2$), 69.2 (*C*(2)H), 119.9 (*C*(5)H$_2$), 127.2 (NBnCH), 127.7 (NBnCH), 128.2 (OBnCH), 128.2 (OBnCH), 128.5 (OBnCH), 128.8 (OBnCH), 133.0 (*C*(4)H), 135.8 (Ar*C*$_{ester}$), 137.2 (Ar*C*$_{amide}$), 169.7 (C=O$_{ester}$), 171.6 (C=O$_{amide}$); *minor rotamer (selected)* δ_C: 34.5 (C(O)NCH$_3$), 42.4 (N(CH$_3$)$_2$), 46.7 (*C*(3)H), 53.0 (NCH$_2$), 65.8 (OCH$_2$), 119.9 (*C*(5)H$_2$), 126.5 (NBnCH), 127.6 (NBnCH), 133.3 (*C*(4)H), 136.6 (Ar*C*$_{amide}$), 169.4 (C=O$_{ester}$), 171.4 (C=O$_{amide}$).

HRMS (ESI$^+$) $C_{23}H_{29}N_2O_3$ [M+H]$^+$ found 381.2165, requires 381.2173 (−2.0 ppm).

ν_{max} ($CHCl_3$, cm^{-1}) 3012 (=CH), 2943 (C-H), 2789 (N(C-H)), 1724 (C=O$_{ester}$), 1643 (C=O$_{amide}$), 1631 (C=C).

Minor diastereoisomer $\mathbf{39_{min}}$ (R_f 0.32 in petrol : EtOAc 1:1) as yellow oil (30 mg, 26%) as a rotameric mixture (2:1).

$[\boldsymbol{\alpha}]_D^{20}$ +0.7 (*c* 0.8 in $CHCl_3$); **chiral HPLC** analysis, Chiralcel OD-H (98:2 hexane : *i*PrOH, flow rate 1 mlmin^{-1}, 211 nm, 40 °C) t_R (2*R*,3*R*): 13.4 min, t_R (2*S*,3*S*): 20.8 min, 53:47 er.

^{1}**H NMR** (500 MHz, $CDCl_3$) *major rotamer* δ_H: 2.36 (6H, s, N(CH_3)$_2$), 2.99 (3H, s, C(O)NCH_3), 3.81 - 3.88 (1H, m, C(3)*H*), 3.95 (1H, d, *J* 10.8, C(2)*H*), 4.57 (2H, s, NCH_2), 5.15 (1H, app. t, *J* 12.3, OCH^AH^B), 5.18 – 5.31 (3H, m, OCH^AH^B, C(5)H_2), 5.87 – 5.97 (1H, m, C(4)*H*), 7.20 (1H, d, *J* 7.5, ArC*H*), 7.24 – 7.29 (1H, m, ArC*H*), 7.29 – 7.44 (8H, m, ArC*H*). *minor rotamer (selected)* δ_H: 2.32 (6H, s, N(CH_3)$_2$), 2.82 (3H, s, C(O)NCH_3), 3.92 (1H, d, *J* 10.8, C(2)*H*), 4.48 (1H, d, *J* 16.4, NCH^AH^B), 4.65 (1H, d, *J* 16.4, NCH^AH^B), 5.07 (1H, d, *J* 17.4, C(5)H^AH^B), 5.15 (1H, app. t, *J* 12.3, OCH^AH^B).

^{13}**C{^{1}H} NMR** (126 MHz, $CDCl_3$) *major rotamer* δ_C: 34.7 (C(O)NCH$_3$), 42.0 (N(CH$_3$)$_2$), 46.6 (*C*(3)H), 50.9 (NCH$_2$), 66.1 (OCH$_2$), 68.8 (*C*(2)H), 118.1 (*C*(5)H$_2$), 127.1 (ArCH), 127.2 (ArCH), 127.5 (ArCH), 127.8 (ArCH), 128.1 (ArCH), 128.4 (ArCH), 128.4 (ArCH), 128.5 (ArCH), 128.5 (ArCH), 128.7 (ArCH), 134.1 (*C*(4)H), 136.0 (ArC_{ester}), 137.2 (ArC_{amide}), 170.6 (C=O_{ester}), 171.6 (C=O_{amide}); *minor rotamer (selected)* δ_C: 33.5 (C(O)NCH$_3$), 42.0 (N(CH$_3$)$_2$), 46.5 (*C*(3)H), 53.2 (NCH$_2$), 66.0 (OCH$_2$), 69.0 (*C*(2)H), 118.1 (*C*(5)H$_2$), 134.6 (*C*(4)H), 136.1 (ArC_{ester}), 136.5 (ArC_{amide}), 170.3 (C=O_{ester}), 171.7 (C=O_{amide}).

HRMS (ESI$^+$) $C_{23}H_{29}N_2O_3$ [M+H]$^+$ found 381.2160, requires 381.2173 (−3.3 ppm).

ν_{max} ($CHCl_3$, cm^{-1}) 3016 (=CH), 2943 (C-H), 2792 (N(C-H)), 1720 (C=O_{ester}), 1627 (C=C), 1593 (C=C_{Ar}).

3.10 (2*R*,3*S*)-2-(Dimethylamino)-3-(pyrrolidine-1-carbonyl)pent-4-enoic acid, benzyl ester (40$_{maj}$) and (2*S*,3*S*)-2-(Dimethylamino)-3-(pyrrolidine-1-carbonyl)pent-4-enoic acid, benzyl ester (40$_{min}$)

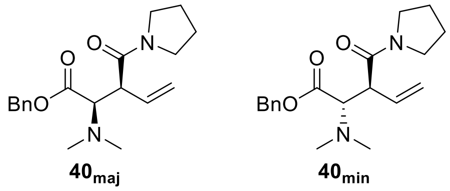

$C_{19}H_{26}N_2O_3$
330.43

Following General Procedure 1.5, PNP ester 13 (78.2 mg, 0.30 mmol, 1.0 eq), $Pd_2dba_3\cdot CHCl_3$ (7.7 mg, 7.5 μmol, 2.5 mol%), P(2-furyl)$_3$ (6.9 mg, 30 μmol, 10 mol%), (*S*)-TM·HCl (14.4 mg, 0.06 mmol, 20 mol%), phosphate **35** (109 mg, 0.37 mmol, 1.25 eq) and *i*Pr$_2$NEt (125 μL, 0.7 mmol, 2.4 eq) in MeCN (5.0 mL) gave the crude product (61:39 dr), which was used directly for derivatisation with NaOBn (0.45 mL, 0.45 mmol, 1.5 eq) in THF (6.0 mL). Subsequent purification of the crude derivatised product via silica column chromatography (petrol : EtOAc 1:2 to EtOAc : 15% *i*PrOH) gave:

Major diastereoisomer **40$_{maj}$** (R_f 0.09 in EtOAc) as yellow glass (28 mg, 28%).

m.p. (EtOAc) 58 – 60 °C; $[\alpha]_D^{20}$ −0.9 (*c* 0.9 in $CHCl_3$); **chiral HPLC** analysis, Chiralcel OD-H (95:5 hexane : *i*PrOH, flow rate 1 mlmin^{-1}, 211 nm, 40 °C) t_R (2*R*,3*S*): 12.4 min, t_R (2*S*,3*R*): 19.0 min, 71:29 er.

^{1}H NMR (500 MHz, $CDCl_3$) δ_H: 1.78 – 2.01 (4H, m, 2 × C$H_{2(pyrr)}$), 2.31 (6H, s, N(CH_3)$_2$), 3.40 – 3.56 (4H, m, 2 × NC$H_{2(pyrr)}$), 3.60 (1H, dd, *J* 11.0, 8.8, C(3)*H*), 3.90 (1H, d, *J* 11.0, C(2)*H*), 5.07 (1H, dd, *J* 10.1, 1.1, C(5)H^AH^B), 5.09 (1H, d, *J* 12.3, OCH^AH^B), 5.13 (1H, d, *J* 12.3, OCH^AH^B), 5.17 (1H, d, *J* 17.2, C(5)H^AH^B), 5.71 (1H, ddd, *J* 17.3, 10.1, 8.9 Hz, C(4)*H*), 7.29 – 7.37 (5H, m, 5 × ArH).

^{13}C{^{1}H} NMR (126 MHz, $CDCl_3$) δ_C: 24.3 ($CH_{2(pyrr)}$), 26.1 ($CH_{2(pyrr)}$), 42.2 (N(CH_3)$_2$), 46.0 (N$CH_{2(pyrr)}$), 46.3 (N$CH_{2(pyrr)}$), 49.4 (*C*(3)H), 65.8 (OCH_2), 68.9 (*C*(2)H), 119.6 (*C*(5)H_2), 128.3 (ArCH), 128.5 (ArCH), 133.2 (*C*(4)H), 135.9 (Ar*C*), 169.1 (C=O_{amide}), 169.8 (C=O_{ester}).

HRMS (ESI$^+$) $C_{19}H_{26}N_2O_3Na$ [M+Na]$^+$ found 353.1826, requires 353.1836 (−2.7 ppm).

ν_{max} ($CHCl_3$, cm^{-1}) 2981 (C-H), 2789 (N(C-H)), 1724 (C=O_{ester}), 1627 (C=O_{amide}).

Minor diastereoisomer **40$_{min}$** (R_f 0.37 in EtOAc) as yellow solid (30 mg, 30%). (JB-210-3)

m.p. (EtOAc) 97 – 99 °C; $[\alpha]_D^{20}$ +4.8 (*c* 0.45 in $CHCl_3$); **chiral HPLC** analysis, Chiralcel OD-H (95:5 hexane : *i*PrOH, flow rate 1 mlmin^{-1}, 211 nm, 40 °C) t_R (2*S*,3*S*): 7.9 min, t_R (2*R*,3*R*): 8.8 min, 66:34 er.

^{1}H NMR (500 MHz, $CDCl_3$) δ_H: 1.71 – 1.92 (4H, m, 2 × C$H_{2(pyrr)}$), 2.32 (6H, s, N(CH_3)$_2$), 3.33 – 3.43 (2H, m, NC$H_{2(pyrr)}$), 3.45 – 3.55 (2H, m, NC$H_{2(pyrr)}$), 3.60 (1H, m, C(3)*H*), 3.89 (1H, d, *J* 10.9, C(2)*H*), 5.04 (1H, d, *J* 12.3, OCH^AH^B), 5.16 (1H, d, *J* 17.3, C(5)H^AH^B), 5.21 (1H, d, *J* 10.1, C(5)H^AH^B), 5.22 (1H, d, *J* 12.3, OCH^AH^B), 5.82 (1H, dt, *J* 17.3, 9.4, C(4)*H*), 7.28 – 7.38 (5H, m, 5 × Ar*H*).

^{13}C{^{1}H} NMR (126 MHz, $CDCl_3$) δ_C: 24.2 ($CH_{2(pyrr)}$), 25.9 ($CH_{2(pyrr)}$), 41.9 (N(CH_3)$_2$), 45.8 (N$CH_{2(pyrr)}$), 46.1 (N$CH_{2(pyrr)}$), 49.0 (*C*(3)H), 66.1 (OCH_2), 68.2 (*C*(2)H), 117.7 (*C*(5)H_2), 128.1 (ArCH), 128.4 (ArCH), 128.4 (ArCH), 134.4 (*C*(4)H), 136.0 (Ar*C*), 169.6 (C=O_{amide}), 170.4 (C=O_{ester}).

HRMS (ESI$^+$) $C_{19}H_{26}N_2O_3Na$ [M+Na]$^+$ found 353.1823, requires 353.1836 (−3.6 ppm).

ν_{max} ($CHCl_3$, cm^{-1}) 2974 (C-H), 2789 (N(C-H)), 1720 (C=O_{ester}), 1627 (C=O_{amide}).

3.11 (2*R*,3*S*)-2-(Dimethylamino)-3-(morpholine-4-carbonyl)pent-4-enoic acid, benzyl ester (41_{maj}) and (2*R*,3*R*)-2-(Dimethylamino)-3-(morpholine-4-carbonyl)pent-4-enoic acid, benzyl ester (41_{min})

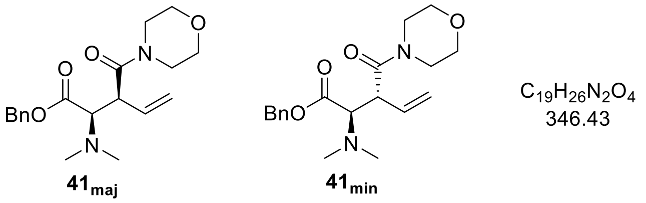

Following General Procedure 1.5, PNP ester **13** (78.2 mg, 0.30 mmol, 1.0 eq), $Pd_2dba_3{\cdot}CHCl_3$ (7.7 mg, 7.5 μmol, 2.5 mol%), P(2-furyl)$_3$ (6.9 mg, 30 μmol, 10 mol%), (*S*)-TM·HCl (14.4 mg, 0.06 mmol, 20 mol%), phosphate **36** (115 mg, 0.37 mmol, 1.25 eq) and *i*Pr_2NEt (125 μL, 0.7 mmol, 2.4 eq) in MeCN (5.0 mL) gave the crude product (64:36 dr), which was used directly for derivatisation with NaOBn (0.45 mL, 0.45 mmol, 1.5 eq) in THF (6.0 mL). Subsequent purification of the crude derivatised product via silica column chromatography (petrol : EtOAc 1:2 to EtOAc : 15% *i*PrOH) gave:

Major diastereoisomer $\mathbf{41_{maj}}$ (R_f 0.14 in EtOAc) as yellow glass (22 mg, 21%).

$[\alpha]_D^{20}$ −0.5 (*c* 1.1 in $CHCl_3$); **chiral HPLC** analysis, Chiralcel OD-H (95:5 hexane : *i*PrOH, flow rate 1 mlmin^{-1}, 211 nm, 40 °C) t_R (2*R*,3*S*): 15.4 min, t_R (2*S*,3*R*): 18.7 min, 75:25 er.

^{1}H NMR (500 MHz, $CDCl_3$) δ_H: 2.30 (6H, s, N(C*H*$_3$)$_2$), 3.50 – 3.74 (8H, m, 4 × C*H*$_{2(morph)}$), 3.77 (1H, dd, *J* 10.9, 8.6, C(3)*H*), 3.90 (1H, d, *J* 10.9, C(2)*H*), 5.09 (1H, d, *J* 12.3, OC*H*^{A}H^B), 5.11 (1H, dd, J 10.2, 0.8, C(5)*H*^{A}H^B), 5.14 (1H, d, *J* 12.3, OCH^A*H*^{B}), 5.15 (1H, br d, *J* 17.2, C(5)H^A*H*^{B}), 5.72 (1H, ddd, *J* 17.3, 10.2, 8.5, C(4)*H*), 7.29 – 7.37 (5H, m, 5 × Ar*H*).

^{13}C{^{1}H} NMR (126 MHz, $CDCl_3$) δ_C: 42.1 (N(CH$_3$)$_2$), 42.6 (NCH$_{2(morph)}$), 46.1 (*C*(3)H), 46.2 (NCH$_{2(morph)}$), 66.0 (OCH$_2$), 66.7 (OCH$_{2(morph)}$), 67.0 (OCH$_{2(morph)}$), 69.0 (*C*(2)H), 120.0 (*C*(5)H$_2$), 128.3 (ArCH), 128.6 (ArCH), 128.6 (ArCH), 133.1 (*C*(4)H), 135.8 (ArC), 169.5 (C=O$_{amide}$), 169.6 (C=O$_{ester}$).

HRMS (ESI$^+$) $C_{19}H_{27}N_2O_4$ [M+H]$^+$ found 347.1959, requires 347.1966 (−1.8 ppm).

ν_{max} ($CHCl_3$, cm^{-1}) 3012 (=CH), 2862 (C-H), 2789 (N(C-H)), 1724 (C=O$_{ester}$), 1643 (C=O$_{amide}$), 1631 (C=C).

Minor diastereoisomer $\mathbf{41_{min}}$ (R_f 0.42 in EtOAc) as yellow glass (12 mg, 11%). (JB-211-3)

$[\alpha]_D^{20}$ +1.6 (*c* 0.35 in $CHCl_3$); **chiral HPLC** analysis, Chiralcel OD-H (97:3 hexane : *i*PrOH, flow rate 1 mlmin^{-1}, 211 nm, 40 °C) t_R (2*S*,3*S*): 14.0 min, t_R (2*R*,3*R*): 15.9 min, 44:56 er.

^{1}H NMR (500 MHz, $CDCl_3$) δ_H: 2.31 (6H, s, N(CH_3)$_2$), 3.46 – 3.52 (3H, m, NC$H_{2(morph)}$, NC$H^AH^B_{(morph)}$), 3.55 – 3.66 (5H, m, 2 × OC$H_{2(morph)}$, NC$H^A$$H^B_{(morph)}$), 3.74 (1H, dd, *J* 10.8, 8.3, C(3)*H*), 3.86 (1H, d, *J* 10.8, C(2)*H*), 5.06 (1H, d, *J* 12.3, OCH^AH^B), 5.14 (1H, dt, *J* 17.3, 0.9, C(5)H^AH^B), 5.23 (1H, d, *J* 12.3, OCH^AH^B), 5.24 (1H, d, *J* 10.3, C(5)H^AH^B), 5.85 (1H, ddd, *J* 17.4, 10.3, 8.3, C(4)*H*), 7.29 – 7.38 (5H, m, 5 × Ar*H*).

$^{13}C\{^{1}H\}$ NMR (126 MHz, $CDCl_3$) δ_C: 42.0 (N(CH_3)$_2$), 42.1 (N$CH_{2(morph)}$), 45.8 (*C*(3)H), 46.0 (N$CH_{2(morph)}$), 66.1 (OCH_2), 66.4 (O$CH_{2(morph)}$), 66.8 (O$CH_{2(morph)}$), 68.6 (*C*(2)H), 118.0 (*C*(5)H_2), 128.2 (ArCH), 128.4 (ArCH), 128.5 (ArCH), 134.3 (*C*(4)H), 135.9 (ArC), 170.0 (C=O_{amide}), 170.3 (C=O_{ester}).

HRMS (ESI$^+$) $C_{19}H_{26}N_2O_4Na$ [M+Na]$^+$ found 369.1772, requires 369.1785 (−3.4 ppm).

ν_{max} ($CHCl_3$, cm^{-1}) 3012 (=CH), 2974 (C-H), 2792 (N(C-H)), 1720 (C=O_{ester}), 1627 (C=C).

3.12 (2*R*,3S)-3-(methoxy(methyl)carbamoyl)-2-(piperidin-1-yl)pent-4-enoic acid, benzyl ester (42$_{maj}$) and (2*R*,3*R*)-3-(methoxy(methyl)carbamoyl)-2-(piperidin-1-yl)pent-4-enoic acid, benzyl ester (42$_{min}$)

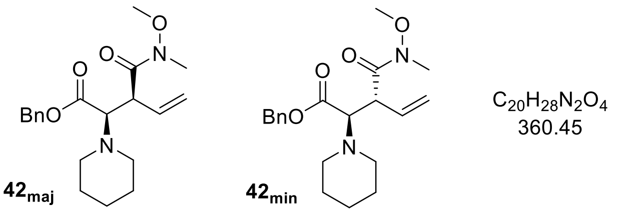

Following General Procedure 1.5, PNP ester **30** (90.2 mg, 0.30 mmol, 1.0 eq), $Pd_2dba_3{\cdot}CHCl_3$ (7.7 mg, 7.5 µmol, 2.5 mol%), P(2-furyl)$_3$ (6.9 mg, 30 µmol, 10 mol%), (*S*)-TM·HCl (14.4 mg, 0.06 mmol, 20 mol%), phosphate **26** (105 mg, 0.37 mmol, 1.25 eq) and *i*Pr_2NEt (125 µL, 0.7 mmol, 2.4 eq) in MeCN (5.0 mL) after 84 h gave the crude product (61:39 dr), which was used directly for derivatisation with NaOBn (0.45 mL, 0.45 mmol, 1.5 eq) in THF (6.0 mL). Subsequent purification of the crude derivatised product via silica column chromatography (CH_2Cl_2 to CH_2Cl_2:Et_2O 95:5, R_f 0.32 in CH_2Cl_2:Et_2O 95:5) gave the title compound as a colourless glass (31 mg, 29%) as an inseparable mixture of diastereoisomers. The enantiomeric ratios for the major and minor diastereoisomer could not be determined.

$[\alpha]_D^{20}$ +2.1 (*c* 1.3 in $CHCl_3$).

HRMS (ESI$^+$) $C_{20}H_{29}N_2O_4$ [M+H]$^+$ found 361.2109, requires 361.2122 (−3.5 ppm).

ν_{max} ($CHCl_3$, cm^{-1}) 2933 (C-H), 1726 (C=O_{ester}), 1658 (C=O_{amide}), 1635 (C=C), 1498 (C=C_{Ar}).

Data for major diastereoisomer **42maj**:

^{1}H NMR (500 MHz, $CDCl_3$) δ_H: 1.29 – 1.55 (6H, m, 3 × C$H_{2(pip)}$), 2.29 – 2.39 (2H, m, NCH_2), 2.66 – 2.75 (2H, m, NCH_2), 3.20 (3H, s, NCH_3), 3.68 (3H, s, OCH_3), 3.71 (1H, d, *J* 11.4, C(2)*H*), 4.14 – 4.26 (1H, m, C(3)*H*), 5.01 – 5.24 (4H, m, C(5)H_2, OCH_2), 5.79 (1H, ddd, *J* 17.2, 10.2, 8.7, C(4)*H*), 7.28 – 7.39 (5H, m, 5 × Ar*H*).

^{13}C{^{1}H} NMR (126 MHz, $CDCl_3$) δ_C: 24.5 (*C*(4)$H_{2(pip)}$), 26.7 (*C*(3,5)$H_{2(pip)}$), 32.4 (NCH_3), 45.7 (*C*(3)H), 51.2 (NCH_2), 61.8 (OCH_3), 65.8 (OCH_2), 70.2 (*C*(2)H), 119.6 (*C*(5)H_2), 128.2 (Ar*C*(4)H), 128.4 (ArC(2,6) and ArC(3,5)), 133.5 (*C*(4)H), 135.9 (Ar*C*(1)), 169.4 (*C*=O_{ester}),

Data for minor diastereomer **42min**:

^{1}H NMR (500 MHz, $CDCl_3$) (*selected*) δ_H: 3.12 (3H, s, NCH_3), 3.73 (1H, d, *J* 11.2, C(2)*H*), 3.98 – 4.08 (1H, C(3)*H*), 5.85 (1H, ddd, *J* 17.3, 10.3, 7.7, C(4)*H*).

^{13}C{^{1}H} NMR (126 MHz, $CDCl_3$) (*selected*) δ_C: 24.5 (*C*(4)$H_{2(pip)}$), 26.5 (*C*(3,5)$H_{2(pip)}$), 32.0 (NCH_3), 44.8 (*C*(3)H), 51.5 (NCH_2), 61.1 (OCH_3), 66.0 (OCH_2), 68.8 (*C*(2)H), 117.3 (*C*(5)H_2), 128.1 (Ar*C*(4)H), 128.4 (ArC(2,3,5,6)), 134.6 (*C*(4)H), 136.0 (Ar*C*(1)), 170.9 (*C*=O_{ester}), 172.7 (*C*=O_{amide})*.

* peak not visible in 1D ^{13}C NMR, but inferred from cross peaks in the HMBC spectrum.

3.13 (2*R*,3*S*)-2-(allyl(methyl)amino)-3-(methoxy(methyl)carbamoyl)pent-4-enoic acid, benzyl ester (43maj) and (2*R*,3*R*)-2-(allyl(methyl)amino)-3-(methoxy(methyl) carbamoyl)pent-4-enoic acid, benzyl ester (43min)

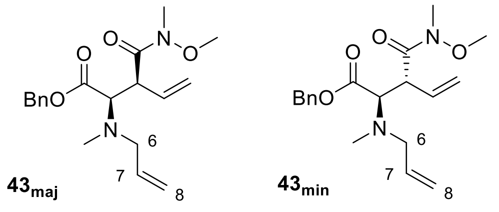

$C_{19}H_{26}N_2O_4$
346.43

Following General Procedure 1.5, PNP ester **31** (86.0 mg, 0.30 mmol, 1.0 eq), $Pd_2dba_3{\cdot}CHCl_3$ (7.7 mg, 7.5 μmol, 2.5 mol%), P(2-furyl)$_3$ (6.9 mg, 30 μmol, 10 mol%), (*S*)-TM·HCl (14.4 mg, 0.06 mmol, 20 mol%), phosphate **26** (105 mg, 0.37 mmol, 1.25 eq) and *i*Pr_2NEt (125 μL, 0.7 mmol, 2.4 eq) in MeCN (5.0 mL) gave the crude product (56:44 dr), which was used directly for derivatisation with NaOBn (0.45 mL, 0.45 mmol, 1.5 eq) in THF (6.0 mL). Subsequent purification of the crude derivatised product via silica column chromatography (CH_2Cl_2 to CH_2Cl_2:Et_2O 95:5) gave:

Major diastereoisomer **43maj** (R_f 0.22 in CH_2Cl_2 : Et_2O 95:5) as colourless oil (11 mg, 11%).

$[\alpha]_D^{20}$ −0.4 (*c* 0.45 in $CHCl_3$); **chiral HPLC** analysis, Chiralcel OD-H (99:1 hexane : *i*PrOH, flow rate 1 mlmin^{-1}, 211 nm, 30 °C) t_R (2*R*,3*S*): 17.1 min, t_R (2*S*,3*R*): 22.7 min, 85:15 er.

^{1}H NMR (500 MHz, $CDCl_3$) δ_H: 2.27 (3H, s, NC*H*$_3$), 2.88 (1H, dd, *J* 13.9, 7.2, C(6)*H*^{A}H^B), 3.19 (3H, s, CONC*H*$_3$), 3.31 (1H, dd, *J* 14.0, 5.2, C(6)H^A*H*^{B}), 3.69 (3H, s, OC*H*$_3$), 3.87 (1H, d, *J* 11.5, C(2)*H*), 4.13 – 4.23 (1H, m, C(3)*H*), 5.03 – 5.12 (4H, m, OC*H*^{A}H^B, C(5)*H*^{A}H^B, C(8)*H*$_2$), 5.16 (1H, d, *J* 12.3, OCH^A*H*^{B}), 5.21 (1H, ddd, *J* 17.1, 1.3, 0.7, C(5)H^A*H*^{B}), 5.69 (1H, dddd, *J* 17.3, 10.1, 7.2, 5.2, C(7)*H*), 5.78 (1H, ddd, *J* 17.2, 10.2, 8.7, C(4)*H*), 7.30 – 7.38 (5H, m, 5 × Ar*H*).

^{13}C{^{1}H} NMR (126 MHz, $CDCl_3$) δ_C: 32.2 (CONCH$_3$), 38.8 (NCH$_3$), 46.0 (*C*(3)H), 57.3 (*C*(6)H$_2$), 61.8 (OCH$_3$), 65.9 (OCH$_2$), 67.7 (*C*(2)H), 116.8 (*C*(8)H$_2$), 119.8 (*C*(5)H$_2$), 128.2 (Ar*C*(4)H), 128.5 (Ar*C*(2,6)H, Ar*C*(3,5)H), 133.4 (*C*(4)H), 135.8 (Ar*C*(1)), 136.2 (*C*(7)H), 169.7 (*C*=O$_{ester}$), 172.3 (*C*=O$_{amide}$)*.

* peak not visible in 1D ^{13}C NMR, but inferred from cross peaks in the HMBC spectrum.

HRMS (ESI$^+$) $C_{19}H_{27}N_2O_4$ [M+H]$^+$ found 347.1954, requires 347.1965 (−3.3 ppm).

ν_{max} ($CHCl_3$, cm^{-1}) 2939 (C-H), 1728 (C=O$_{ester}$), 1660 (C=O$_{amide}$), 1637 (C=C), 1496 (C=C$_{Ar}$).

Minor diastereoisomer **43$_{min}$** (R_f 0.30 in CH_2Cl_2 : Et_2O 95:5) as colourless oil (5 mg, 5%).

$[\alpha]_D^{20}$ +9.5 (*c* 0.2 in $CHCl_3$). (Enantiomeric ratio could not be determined.)

^{1}H NMR (500 MHz, $CDCl_3$) δ_H: 2.24 (3H, s, NC*H*$_3$), 3.05 – 3.11 (1H, m, C(6)*H*^{A}H^B), 3.13 (3H, s, CONC*H*$_3$), 3.22 (1H, ddt, *J* 13.9, 5.6, 1.6 Hz, 1H, m, C(6)H^A*H*^{B}), 3.70 (3H, s, OC*H*$_3$), 3.94 (1H, d, *J* 11.2, C(2)*H*), 3.99 – 4.09 (1H, m, C(3)*H*), 5.05 (1H, d, *J* 12.3, OC*H*^{A}H^B), 5.07 – 5.10 (1H, m, C(8)*H*^{A}H^B), 5.13 (1H, dq, *J* 17.2, 1.7, C(8)H^A*H*^{B}), 5.16 – 5.21 (2H, m, C(5)*H*$_2$), 5.24 (1H, d, *J* 12.3, OCH^A*H*^{B}), 5.72 (1H, dddd, *J* 17.2, 10.1, 7.1, 5.6, C(7)*H*), 5.82 (1H, ddd, *J* 17.1, 10.3, 8.1, C(4)*H*), 7.29 – 7.39 (5H, m, 5 × Ar*H*).

^{13}C{^{1}H} NMR (126 MHz, $CDCl_3$) δ_C: 32.1 (CONCH$_3$), 38.1 (NCH$_3$), 45.5 (*C*(3)H), 58.0 (*C*(6)H$_2$), 61.0 (OCH$_3$), 66.2 (OCH$_2$), 66.3 (*C*(2)H), 117.4 (*C*(8)H$_2$), 117.9 (*C*(5)H$_2$), 128.2 (Ar*C*(4)H), 128.4 (Ar*C*(2,6 or 3,5)H), 128.5 (Ar*C*(2,6 or 3,5)H), 134.4 (*C*(4)H), 135.9 (*C*(7)H), 135.9 (Ar*C*(1)), 171.1 (C=O$_{ester}$). C=O$_{amide}$ not visible.

HRMS (ESI$^+$) $C_{19}H_{27}N_2O_4$ [M+H]$^+$ found 347.1956, requires 347.1965 (−2.6 ppm).

ν_{max} ($CHCl_3$, cm^{-1}) 2978 (C-H), 1722 (C=O$_{ester}$), 1654 (C=O$_{amide}$), 1635 (C=C), 1498 (C=C$_{Ar}$).

4. Identified Side Products

4.1 (*E*)-4-(Diisopropylamino)but-2-enoic acid, ethyl ester (22)

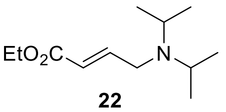

22

^{1}H NMR (400 MHz, $CDCl_3$) δ_H: 0.99 (12H, d, *J* 6.6, 2 × CH(C*H*$_3$)$_2$), 1.28 (3H, t, *J* 7.1, OCH_2CH_3), 3.02 (2H, hept, *J* 6.6, 2 × C*H*(CH_3)$_2$), 3.23 (2H, dd, *J* 5.2, 1.9, C(4)*H*$_2$), 4.18 (2H, q, *J* 7.1, OC*H*$_2$CH$_3$), 6.04 (1H, dt, *J* 15.5, 1.9, C(2)*H*), 6.98 (1H, dt, *J* 15.5, 5.2, C(3)*H*).

4.2 (*E*)-2-(1,2-Bis(benzylamino)-2-oxoethyl)but-2-enoic acid, ethyl ester (25)

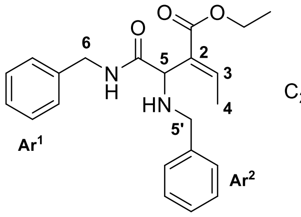

$C_{22}H_{26}N_2O_3$
366.46
25

PNP ester **18** (30.0 mg, 0.09 mmol, 1.0 eq) was dissolved in 2.0 mL CH_2Cl_2, benzylamine (48 μL, 0.45 mmol, 5.0 eq) added and the reaction mixture stirred at room temperature overnight. The reaction was quenched by the addition of 1M NaOH, diluted with CH_2Cl_2 and the phases separated. The aqueous phase was extracted with CH_2Cl_2 (3 × 5 mL). The combined organic phases were washed with 1M NaOH (2 × 5 mL), and brine (5 mL), dried over $MgSO_4$, filtered and the solvent removed under reduced pressure. The crude product was purified by silica column chromatography (CH_2Cl_2 : Et_2O 9:1 to CH_2Cl_2:Et_2O 4:1) to give the title compound as a yellow, glassy solid (10 mg, 30%) as rotameric mixture (6:1).

^{1}H NMR (500 MHz, $CDCl_3$) *major rotamer* δ_H: 1.26 (3H, t, *J* 7.1, OCH$_2$C*H*$_3$), 1.83 (3H, d, *J* 7.2, C(4)*H*$_3$), 3.61 (1H, d, *J* 13.2, C(5′)*H*^{A}H^B), 3.86 (1H, d, *J* 13.2, C(5′)H^A*H*^{B}), 4.11 (1H, s, C(5)*H*), 4.18 (2H, qd, *J* 7.1, 1.2, OC*H*$_2$CH$_3$), 4.49 (2H, d, *J* 6.0, C(6)*H*$_2$), 7.21 – 7.25 (1H, q, *J* 7.2, C(3)*H*), 7.26 – 7.37 (10H, m, Ar*H*), 7.96 (1H, t, *J* 5.5, C(O)N*H*); *minor rotamer (selected)* δ_H: 2.13 (3H, d, *J* 7.2, C(4)*H*$_3$), 3.68 (1H, s, C(5)*H*), 4.47 (2H, d, *J* 6.0, C(6)*H*$_2$), 6.20 (1H, q, *J* 7.2, C(3)*H*), 7.79 (1H, m, C(O)N*H*).

^{13}C{^{1}H} NMR (126 MHz, $CDCl_3$) *major rotamer* δ_C: 14.1 (OCH_2CH_3), 14.6 (*C*(4)H$_3$), 43.1 (*C*(6)H$_2$), 51.3 (*C*(5′)H$_2$), 57.6 (*C*(5)H), 60.7 (OCH_2CH_3), 127.2 (Ar^1*C*(4)H), 127.3 (Ar^2*C*(4)H), 127.6 (Ar^1*C*(2,6)H), 128.1 (Ar^2*C*(2,6)H), 128.5 (Ar^1*C*(3,5)H), 128.6 (Ar^2*C*(3,5)H), 130.0 (*C*(2)), 138.7

(Ar^1*C*(1)), 139.2 (Ar^2*C*(1)), 142.2 (*C*(3)H), 166.5 (*C*=O$_{amide}$), 171.7 (*C*=O$_{ester}$); *minor rotamer (selected)* δ$_C$: 16.0 (*C*(4)H$_3$), 66.2 (*C*(2)H), 143.0 (*C*(3)H).

HRMS (ESI$^+$) $C_{22}H_{27}N_2O_3$ [M+H]$^+$ found 367.2007, requires 367.2016 (−2.5 ppm).

ν$_{max}$ ($CHCl_3$, cm^{-1}) 3367 (N-H), 3028 (=C-H), 2980 (C-H), 1699 (C=O$_{Ester}$), 1674 (C=O$_{Amide}$), 1516 (C=O$_{Amide}$), 1496 (C=C$_{Ar}$).

4.3 2-(Allyl(methyl)amino)pent-4-enoic acid, benzyl ester (44)

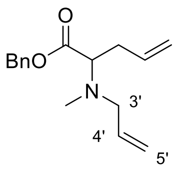

$C_{16}H_{21}NO_2$
259.35
44

Following General Procedure 1.5, PNP ester **31** (86.0 mg, 0.30 mmol, 1.0 eq), Pd_2dba_3·$CHCl_3$ (7.7 mg, 7.5 μmol, 2.5 mol%), P(2-furyl)$_3$ (6.9 mg, 30 μmol, 10 mol%), (*S*)-TM·HCl (14.4 mg, 0.06 mmol, 20 mol%), phosphate **26** (105 mg, 0.37 mmol, 1.25 eq) and *i*Pr$_2$NEt (125 μL, 0.7 mmol, 2.4 eq) in MeCN (5.0 mL) gave the crude product, which was used directly for derivatisation with NaOBn (0.45 mL, 0.45 mmol, 1.5 eq) in THF (6.0 mL). Subsequent purification of the crude derivatised product via silica column chromatography (CH_2Cl_2 to CH_2Cl_2:Et_2O 95:5, R$_f$ 0.44 in CH_2Cl_2:Et_2O 95:5) afforded the title compound as yellow glassy solid (10 mg, 13%).

^{1}H NMR (500 MHz, $CDCl_3$) δ$_H$: 2.31 (3H, s, NC*H*$_3$), 2.41 (1H, dtt, *J* 14.1, 6.9, 1.4, C(3)*H*^{A}H^B), 2.53 (1H, dddt, *J* 14.2, 8.3, 7.1, 1.3, C(3)H^A*H*^{B}), 3.10 (1H, ddt, *J* 13.8, 6.9, 1.2, C(3′)*H*^{A}H^B), 3.22 (1H, ddt, *J* 13.8, 5.9, 1.5, C(3′)H^A*H*^{B}), 3.43 (1H, dd, *J* 8.1, 7.0, C(2)*H*), 5.00 – 5.18 (6H, m, OC*H*$_2$, C(5)*H*$_2$, C(5′)*H*$_2$), 5.72 – 5.84 (2H, m, C(4)*H*, C(4′)*H*), 7.29 – 7.38 (5H, m, 5 × Ar*H*).

^{13}C{^{1}H} NMR (126 MHz, $CDCl_3$) δ$_C$: 34.1 (*C*(3)H$_2$), 37.8 (NCH$_3$), 57.5 (*C*(3′)H$_2$), 65.4 (*C*(2)H), 65.9 (OCH$_2$), 117.1 (*C*(5)H$_2$), 117.4 (*C*(5′)H$_2$), 128.2 (Ar*C*(4)H), 128.4 (Ar*C*(2,6)H), 128.5 (Ar*C*(3,5)H), 134.5 (*C*(4)H), 135.9 (*C*(4′)H, Ar*C*(1)), 171.8 (*C*=O).

5. Determination of Product Configuration

5.1 X-ray single crystal analysis

X-ray diffraction data were collected at 125 K using a Rigaku MM-007HF High Brilliance RA generator/confocal optics with XtaLAB P200 diffractometer [Cu Kα radiation (λ = 1.54187 Å)]. Data were collected using CrystalClear[1] and processed (including correction for Lorentz, polarization and absorption) using CrysAlisPro.[2] Structures were solved by dual-space (SHELXT[3]), direct (SIR2011[4]) or charge-flipping (Superflip[5]) methods and refined by full-matrix least-squares against F_2 (SHELXL-2018/3[6]). Non-hydrogen atoms were refined anisotropically, and all hydrogen atoms were refined using a riding model. All calculations were performed using the CrystalStructure[7] interface.

Data for **28**$_{\mathbf{maj}}$

	**28**$_{\mathbf{maj}}$
CCDC	2000698
empirical formula	$C_{14}H_{25}N_3O_3$
fw	283.37
crystal description	colourless, plate
crystal size [mm]	0.100 × 0.050 × 0.020
space group	C2/c (#15)
a [Å]	20.9650(8)
b [Å]	6.25949(18)
c [Å]	23.5608(11)
vol [Å]^3	3022.0(2)
β [°]	102.201(4)
Z	8
ρ (calc) [g/cm^3]	1.246
μ [mm^{-1}]	0.718
F(000)	1232.00
reflections collected	16059
independent reflections (R_{int})	3076 (0.0711)
data/parameters	3076/181
GOF on F^2	1.035
R_1 [$I > 2\sigma(I)$]	0.0951
wR_2 (all data)	0.2765
largest diff. peak/hole [e/Å^3]	0.84, −0.50

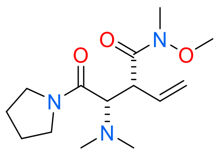

28$_{\mathbf{maj}}$

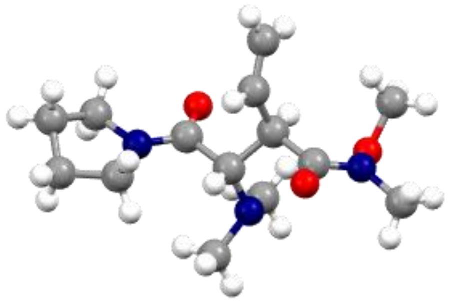

[1] CrystalClear-SM Expert v2.1. Rigaku Americas, The Woodlands, Texas, USA, and Rigaku Corporation, Tokyo, Japan, 2015

[2] CrysAlisPro v1.171.38.46. Rigaku Oxford Diffraction, Rigaku Corporation, Oxford, U.K. 2015

[3] G. M. Sheldrick, *Acta Crystallogr., Sect. A.* **2015**, *71*, 3–8.

[4] M. C. Burla, R. Caliandro, M. Camalli, B. Carrozzini, G. L. Cascarano, C. Giacovazzo, M. Mallamo, A. Mazzone, G. Polidori, R. Spagna, *J. Appl. Cryst.* **2012**, *45*, 357–361.

[5] Palatinus, L. Chapuis, G. *J. Appl. Cryst.* **2007**, *40*, 786–790.

[6] G. M. Sheldrick, *Acta Crystallogr., Sect. C.* **2015**, *71*, 3–8.

[7] CrystalStructure v4.3.0. Rigaku Americas, The Woodlands, Texas, USA, and Rigaku Corporation, Tokyo, Japan, 2018.

Data for **(*E*)-37**

	**(*E*)-37**
CCDC	2000699
empirical formula	$C_{14}H_{16}N_2O_2$
fw	244.29
crystal description	colourless, prism
crystal size [mm]	0.200 × 0.100 × 0.050
space group	P−1(#2)
a [Å]	8.1528(2)
b [Å]	9.51231(16)
c [Å]	16.9230(4)
vol [Å]^3	1256.30(5)
α [°]	101.2680(17)
β [°]	92.223(2)
γ [°]	101.6720(18)
Z	4
ρ (calc) [g/cm^3]	1.291
μ [mm^{-1}]	0.709
F(000)	520.00
reflections collected	13192
independent reflections (R_{int})	4892 (0.0154)
data/parameters	4892/332
GOF on F^2	1.064
R_1 [$I > 2\sigma(I)$]	0.0415
wR_2 (all data)	0.1093
largest diff. peak/hole [e/Å^3]	0.25, −0.27

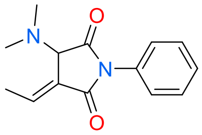

(*E*)-37

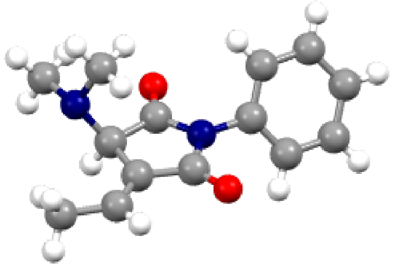

Data for **40**$_{min}$

	**40**$_{min}$
CCDC	2000700
empirical formula	$C_{19}H_{26}N_2O_3$
fw	330.42
crystal description	colourless, prism
crystal size [mm]	0.110 × 0.090 × 0.080
space group	P1(#1)
a [Å]	5.95743(14)
b [Å]	7.72174(8)
c [Å]	10.83510(9)
vol [Å]^3	445.939(13)
α [°]	101.1780(7)
β [°]	97.4202(15)
γ [°]	110.9130(17)
Z	1
ρ (calc) [g/cm^3]	1.230
μ [mm^{-1}]	0.670
F(000)	178.00
reflections collected	11042
independent reflections (R_{int})	3250 (0.0232)
data/parameters	3250/237
GOF on F^2	1.071
R_1 [$I > 2\sigma(I)$]	0.0315
wR_2 (all data)	0.0855
largest diff. peak/hole [e/Å^3]	0.15, −0.15
Flack parameter	0.08(9)

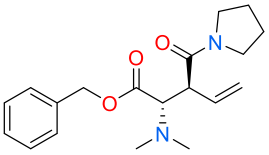

40$_{min}$

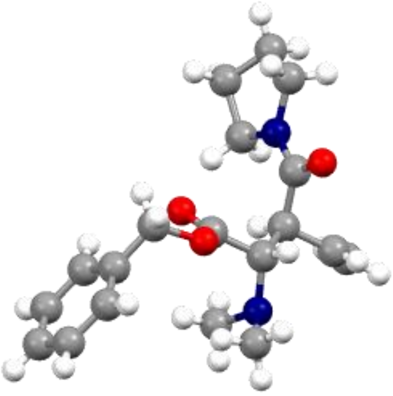

5.2 NOE Experiment

Experiments were performed on a 400 MHz spectrometer. For each experiment, a ^{1}H NMR spectrum was obtained and a NOE was obtained on the same NMR spectrometer with a selective pulse at the required chemical shift to 4 decimal places obtained from MestreNova 9.1 without referencing to solvent.

Determination of alkene configuration for **25**:

Amide **25** (approx. 10 mg) was dissolved in $CDCl_3$ (approx. 0.6 mL) and a ^{1}H NMR spectrum was obtained. The chemical shift of the alkene CH_3 signal (C(4)H_3, δ_H 1.8570 ppm, uncorrected) was determined and subsequently irradiated on the same NMR spectrometer. The resulting spectrum showed significant radiation transfer to protons with chemical shifts of 4.13 ppm (s, C(5)H) and 7.20–7.31 ppm (m, C(3)H). Then, the chemical shift of the C(5)H signal (C(5)H, δ_H 4.1359 ppm, uncorrected) was determined and irradiated on the same NMR

spectrometer. The resulting spectrum showed significant radiation transfer to protons with chemical shifts of 1.86 (d, *J* 7.2 Hz, C(4)H3) and 3.64 (d, J 13.2 Hz, C(5')H2).

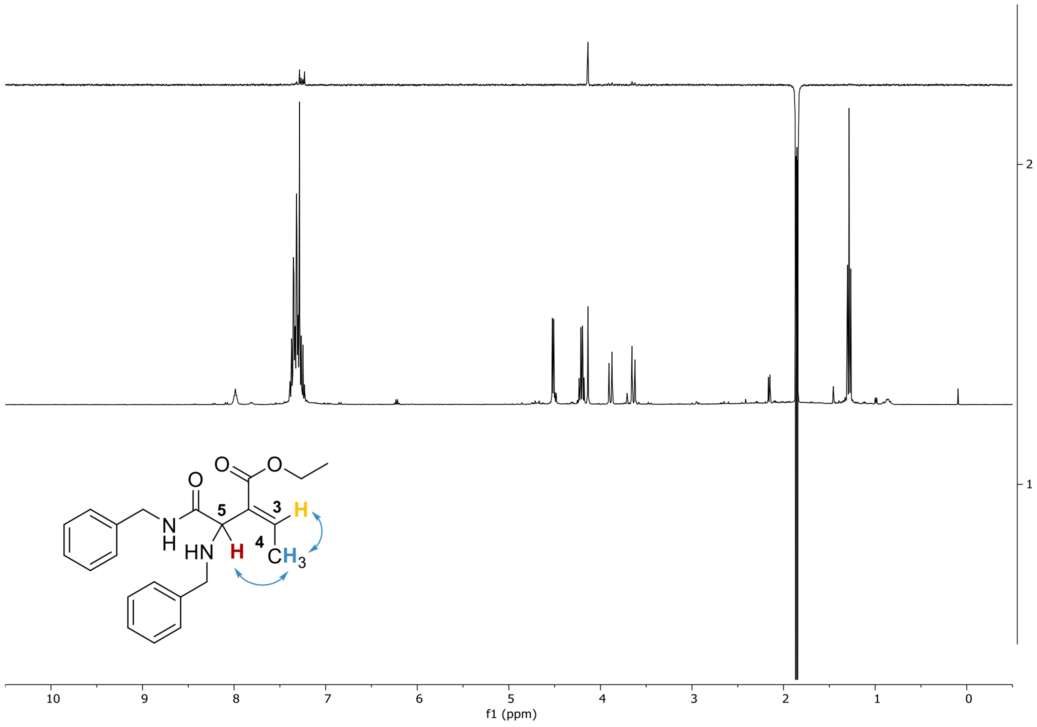

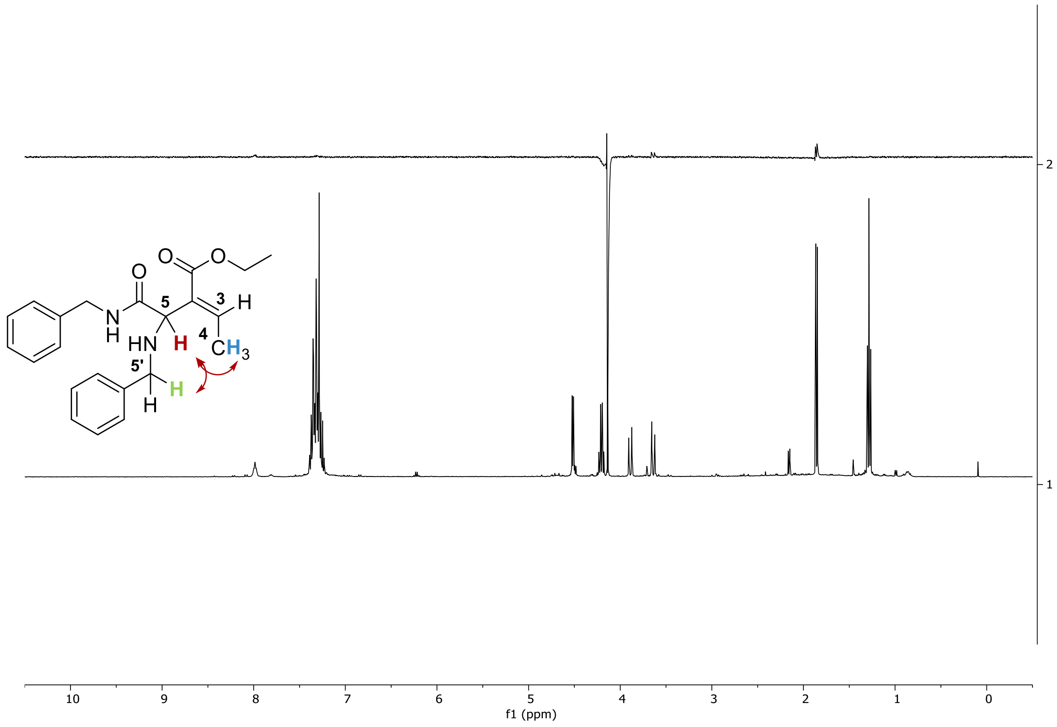

Figure S1: NOE spectra for ***25****. ^{1}H NMR, CDCl3, 400 MHz*

6. References

(1) Daniels, D.; Smith, S.; Lebl, T.; Shapland, P.; Smith, A. A Scalable, Chromatography-Free Synthesis of Benzotetramisole. *Synthesis (Stuttg).* **2014**, *47* (01), 34–41. https://doi.org/10.1055/s-0034-1378931.

(2) Spoehrle, S. S. M.; West, T. H.; Taylor, J. E.; Slawin, A. M. Z.; Smith, A. D. Tandem Palladium and Isothiourea Relay Catalysis: Enantioselective Synthesis of α-Amino Acid Derivatives via Allylic Amination and [2,3]-Sigmatropic Rearrangement. *J. Am. Chem. Soc.* **2017**, *139* (34), 11895–11902. https://doi.org/10.1021/jacs.7b05619.

(3) Ronson, T. O.; Carney, J. R.; Whitwood, A. C.; Taylor, R. J. K.; Fairlamb, I. J. S. AsCat and FurCat: New Pd Catalysts for Selective Room-Temperature Stille Cross-Couplings of Benzyl Chlorides with Organostannanes. *Chem. Commun.* **2015**, *51* (16), 3466–3469. https://doi.org/10.1039/C4CC09810B.

(4) Zalesskiy, S. S.; Ananikov, V. P. Pd 2(Dba) 3 as a Precursor of Soluble Metal Complexes and Nanoparticles: Determination of Palladium Active Species for Catalysis and Synthesis. *Organometallics* **2012**, *31* (6), 2302–2309. https://doi.org/10.1021/om201217r.

(5) Hutchings-Goetz, L.; Yang, C.; Snaddon, T. N. Enantioselective α-Allylation of Aryl Acetic Acid Esters via C1-Ammonium Enolate Nucleophiles: Identification of a Broadly Effective Palladium Catalyst for Electron-Deficient Electrophiles. *ACS Catal.* **2018**, *8* (11), 10537–10544. https://doi.org/10.1021/acscatal.8b03507.

(6) Matviitsuk, A.; Greenhalgh, M. D.; Antúnez, D.-J. B.; Slawin, A. M. Z.; Smith, A. D. Aryloxide-Facilitated Catalyst Turnover in Enantioselective α,β-Unsaturated Acyl Ammonium Catalysis. *Angew. Chem., Int. Ed.* **2017**, *56* (40), 12282–12287. https://doi.org/10.1002/anie.201706402.

(7) Jacobi, P. A.; Blum, C. A.; DeSimone, R. W.; Udodong, U. E. S. Bis-Heteroannulation. 15. Enantiospecific Syntheses of (+)- and (-)-Norsecurinine. *J. Am. Chem. Soc.* **1991**, *113* (14), 5384–5392. https://doi.org/10.1021/ja00014a033.

(8) Abraham, E.; Cooke, J. W. B.; Davies, S. G.; Naylor, A.; Nicholson, R. L.; Price, P. D.; Smith, A. D. Asymmetric Synthesis of 4-Amino-γ-Butyrolactones via Lithium Amide Conjugate Addition. *Tetrahedron* **2007**, *63* (26), 5855–5872. https://doi.org/10.1016/j.tet.2007.03.026.

(9) Tian, G.-Q.; Yang, J.; Rosa-Perez, K. γ-Oxygenation of α,β-Unsaturated Esters by Vinylogous O -Nitroso Mukaiyama Aldol Reaction. *Org. Lett.* **2010**, *12* (21), 5072–5074. https://doi.org/10.1021/ol1021433.

(10) Kacprzynski, M. A.; Kazane, S. A.; May, T. L.; Hoveyda, A. H. Cu-Catalyzed Asymmetric Conjugate Additions of Dialkyl- and Diarylzinc Reagents to Acyclic β-Silyl-α,β-Unsaturated Ketones. Synthesis of Allylsilanes in High Diastereo- and Enantiomeric Purity. *Org. Lett.* **2007**, *9* (16), 3187–3190. https://doi.org/10.1021/ol071331k.

(11) Yang, Q.; Njardarson, J. T.; Draghici, C.; Li, F. Total Synthesis of Vinigrol. *Angew. Chem., Int. Ed.* **2013**, *52* (33), 8648–8651. https://doi.org/10.1002/anie.201304624.

(12) Hwu, J. R.; Furth, P. S. Silicon-Directed Decarbonylation of Trimethylsilyl β, γ-Enals by Photolysis. *J. Am. Chem. Soc.* **1989**, *111* (24), 8834–8841. https://doi.org/10.1021/ja00206a011.

(13) Shintani, R.; Takatsu, K.; Takeda, M.; Hayashi, T. Copper-Catalyzed Asymmetric Allylic Substitution of Allyl Phosphates with Aryl- and Alkenylboronates. *Angew. Chem., Int. Ed.* **2011**, *50* (37), 8656–8659. https://doi.org/10.1002/anie.201103581.

(14) Yoshimura, Y.; Yamazaki, Y.; Kawahata, M.; Yamaguchi, K.; Takahata, H. Design and Synthesis of a Novel Ring-Expanded 4′-Thio-Apio-Nucleoside Derivatives. *Tetrahedron Lett.* **2007**, *48* (26), 4519–4522. https://doi.org/10.1016/j.tetlet.2007.04.139.

(15) Schwarz, K. J.; Pearson, C. M.; Cintron-Rosado, G. A.; Liu, P.; Snaddon, T. N. Traversing Steric Limitations by Cooperative Lewis Base/Palladium Catalysis: An Enantioselective Synthesis of α-Branched Esters Using 2-Substituted Allyl Electrophiles. *Angew. Chem., Int. Ed.* **2018**, *57* (26), 7800–7803. https://doi.org/10.1002/anie.201803277.

(16) Mailig, M.; Hazra, A.; Armstrong, M. K.; Lalic, G. Catalytic Anti-Markovnikov Hydroallylation of Terminal and Functionalized Internal Alkynes: Synthesis of Skipped Dienes and Trisubstituted Alkenes. *J. Am. Chem. Soc.* **2017**, *139* (20), 6969–6977. https://doi.org/10.1021/jacs.7b02104.

(17) Jacobi, P. A.; Blum, C. A.; DeSimone, R. W.; Udodong, U. E. S. Total Synthesis of (+)- and (−)-Norsecurinine. *Tetrahedron Lett.* **1989**, *30* (51), 7173–7176. https://doi.org/10.1016/S0040-4039(01)93926-7.

(18) Abdou, A. M.; Botros, S.; Hassan, R. A.; Kamel, M. M.; Taber, D. F.; Taher, A. T. Useful

Four-Carbon Synthons En Route to Monastrol Analogs. *Tetrahedron* **2015**, *71* (1), 139–146. https://doi.org/10.1016/j.tet.2014.11.022.

(19) Yang, Z.; Ma, M.; Yang, C.-H.; Gao, Y.; Zhang, Q.; Chen, Y. Determination of the Absolute Configurations of Microtermolides A and B. *J. Nat. Prod.* **2016**, *79* (9), 2408–2412. https://doi.org/10.1021/acs.jnatprod.5b01143.

Appendix I. Proposed Mechanisms for the Formation of Side Products

Proposed Mechanism for the Formation of **25**

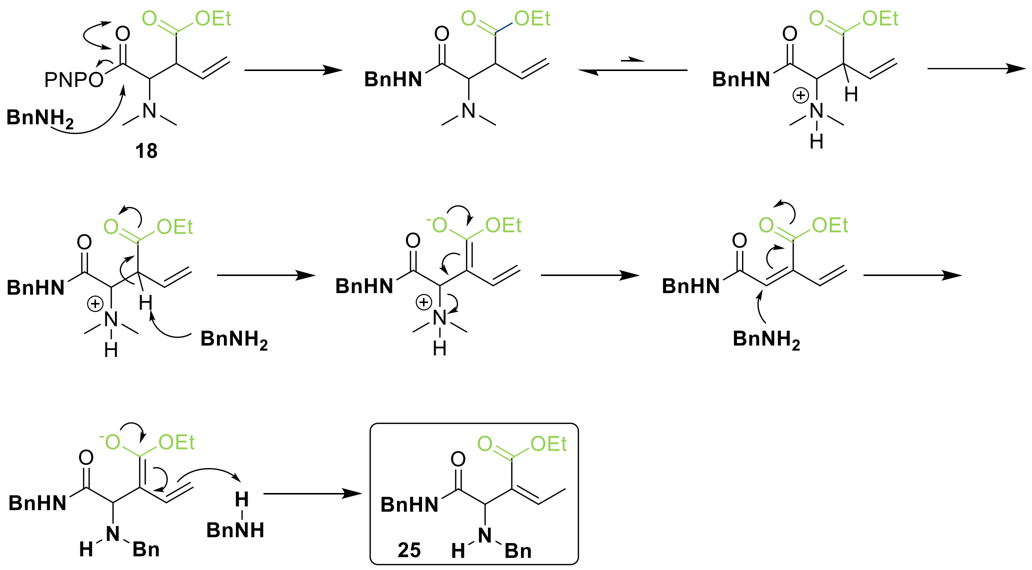

Figure A1: Proposed mechanism for the formation of 25

Proposed Mechanism for the Formation of **37**

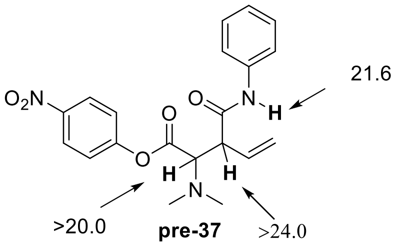

Imide **37** is proposed to be formed by intramolecular cyclisation of **pre-37**, formed *in situ* from the allylation/[2,3]-rearrangement sequence. Rough estimations of pKa values in DMSO for most acidic protons in **pre-37** based on values taken from Evan's pKa table[8] suggest that deprotonation of N-H is feasible under the reaction conditions.

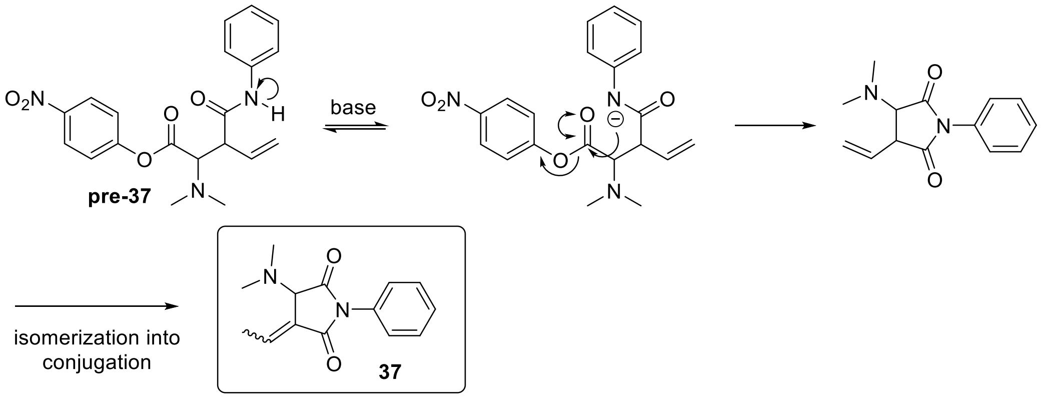

Figure A2: Proposed mechanism for the formation of 37

[8] Evan's pKa Table, http://evans.rc.fas.harvard.edu/pdf/evans_pKa_table.pdf (accessed: 01.05.2020)

Proposed Mechanism for the Formation of **44**

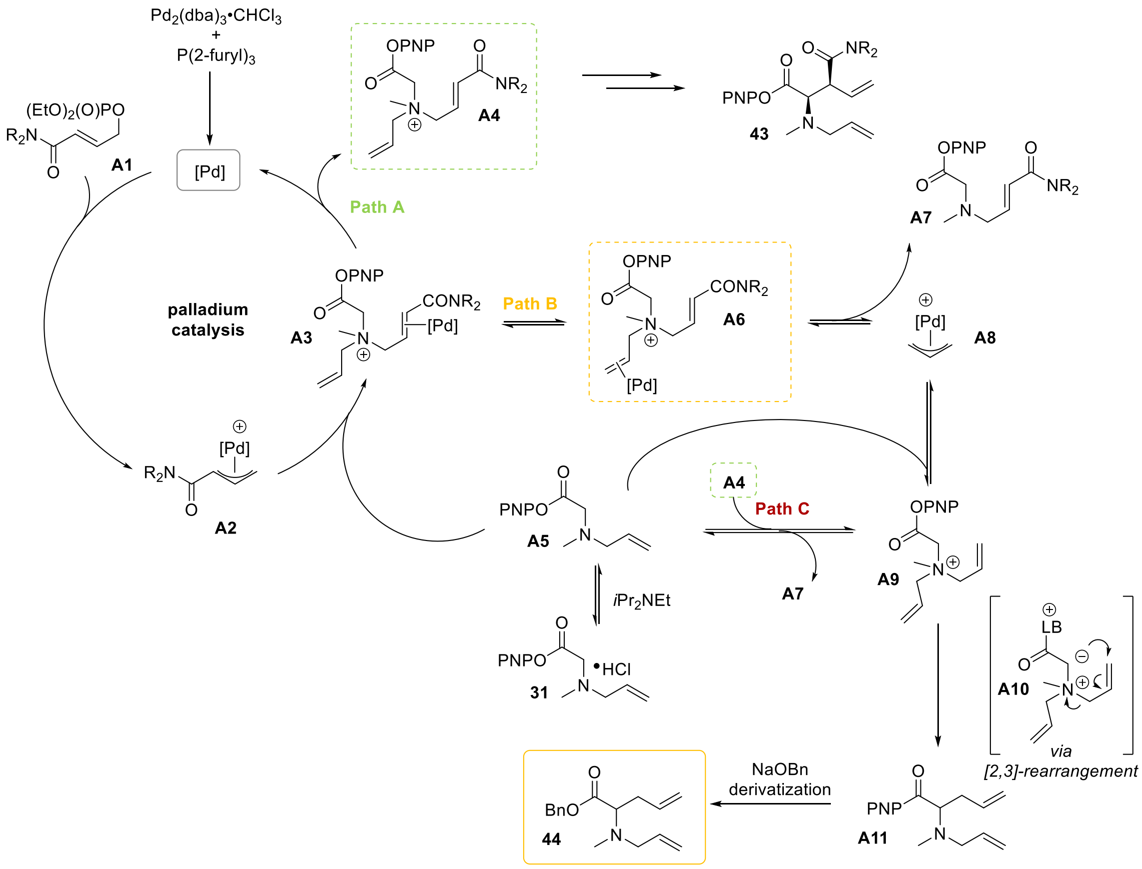

Figure A3: Proposed mechanism for the formation of 44. Counterions are omitted for simplification.

The presence of a *N*-methyl-*N*-allyl group in PNP ester **31** opens the possibility for the reaction to proceed in different pathways once the intermediate ammonium salt **A3** is formed. Path A follows the anticipated pathway for the allylation/[2,3]-rearrangement sequence, yielding intermediate ammonium salt **A4**, which upon rearrangement gives the desired product **43**. However, due to the proximity of the *N*-substituents in **A3** it is plausible that, once the palladium catalyst dissociates from the amide containing allylic fragment, it coordinates to the unsubstituted allylic fragment (**A6**, Path B). As the nucleophilic substitution step is reversible, Pd-π-allyl complex **A8** can form. This step also produces **A7** as an unproductive side product, however, this could not be isolated. The unsubstituted Pd-π-allyl complex **A8** can react with another molecule of PNP ester **A5**, giving ammonium salt **A9** bearing two unsubstituted allyl fragments. It is also conceivable that **A9** can be obtained from an uncatalyzed nucleophilic substitution of ammonium salt **A4** with a molecule of PNP ester **A5** (Path C). [2,3]-rearrangement of ylide **A10** formed from ammonium salt **A9** will lead to PNP ester **A11**, which upon derivatization with NaOBn gives the observed side product **44**.

Appendix II. ^{1}H, $^{13}C\{^{1}H\}$ and $^{31}P\{^{1}H\}$ NMR Spectra of Novel Compounds

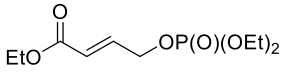

14

^1H, $CDCl_3$, 400 MHz

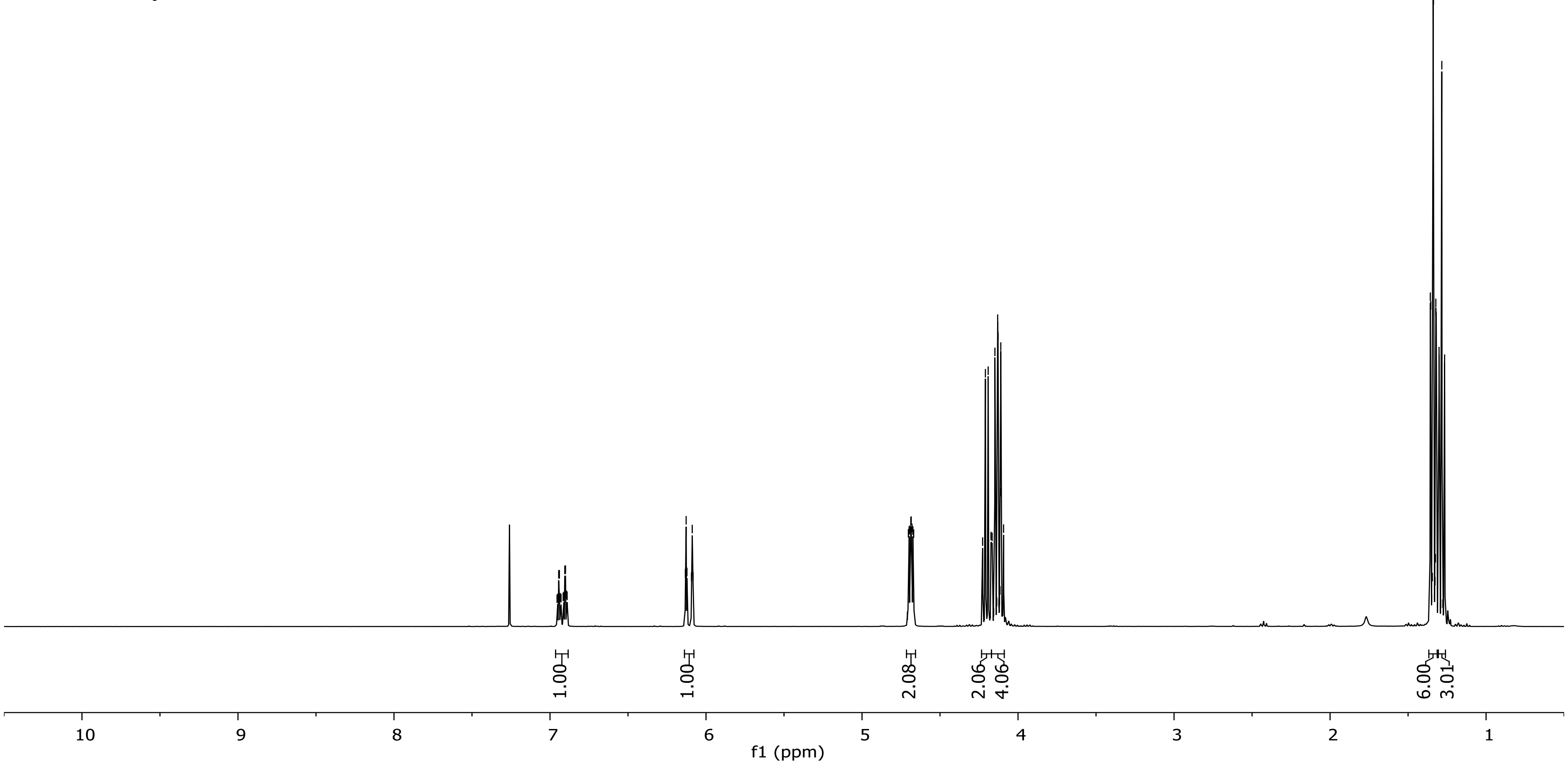

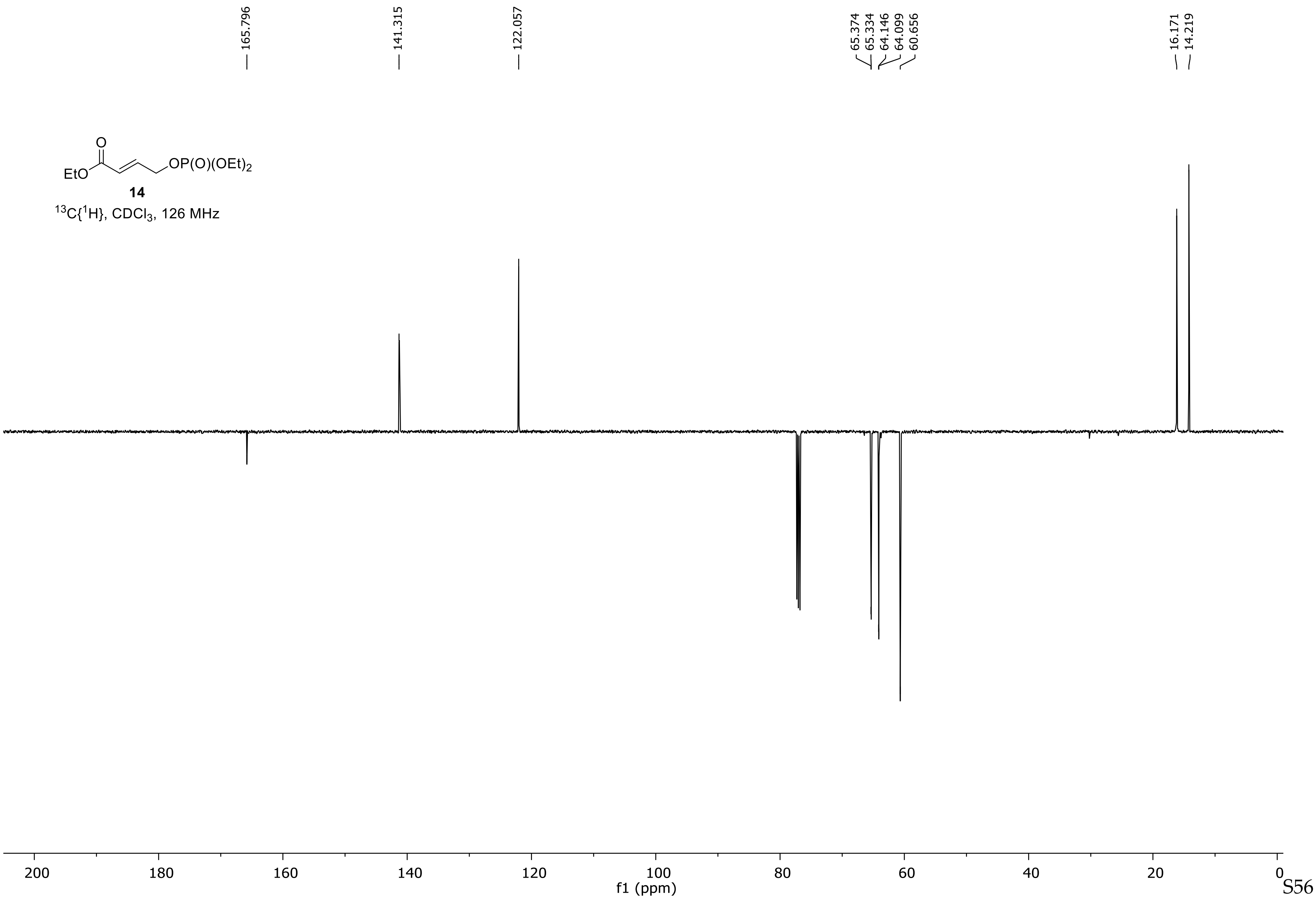

165.796
141.315
122.057
65.374
65.334
64.146
64.099
60.656
16.171
14.219
O
EtO
OP(O)(OEt)2
14
13C{1H}, CDCl3, 126 MHz
200
180
160
140
120
100
80
60
40
20
0
f1 (ppm)

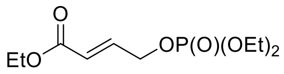

14

$^{31}P\{^{1}H\}$, $CDCl_3$, 162 MHz

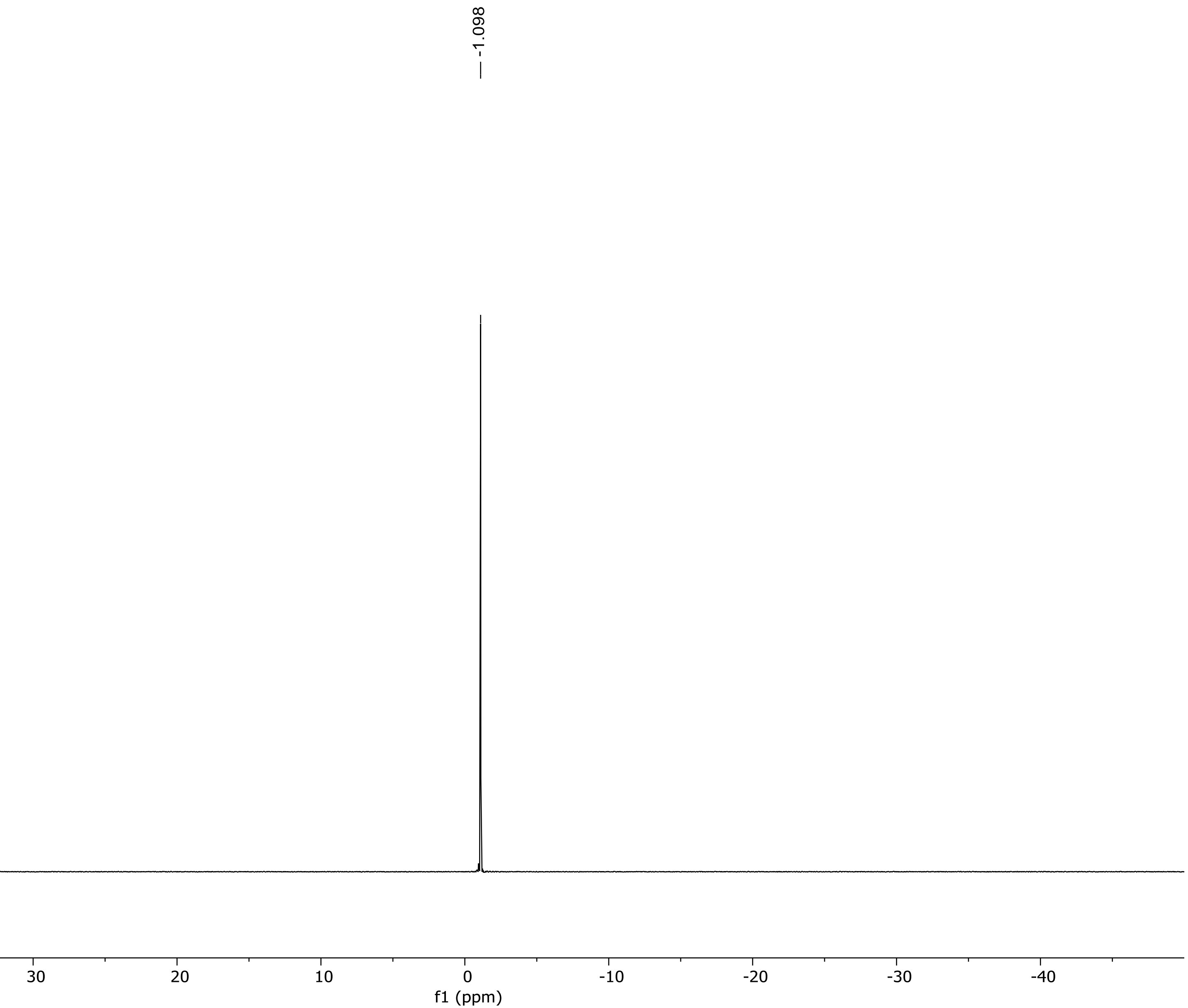

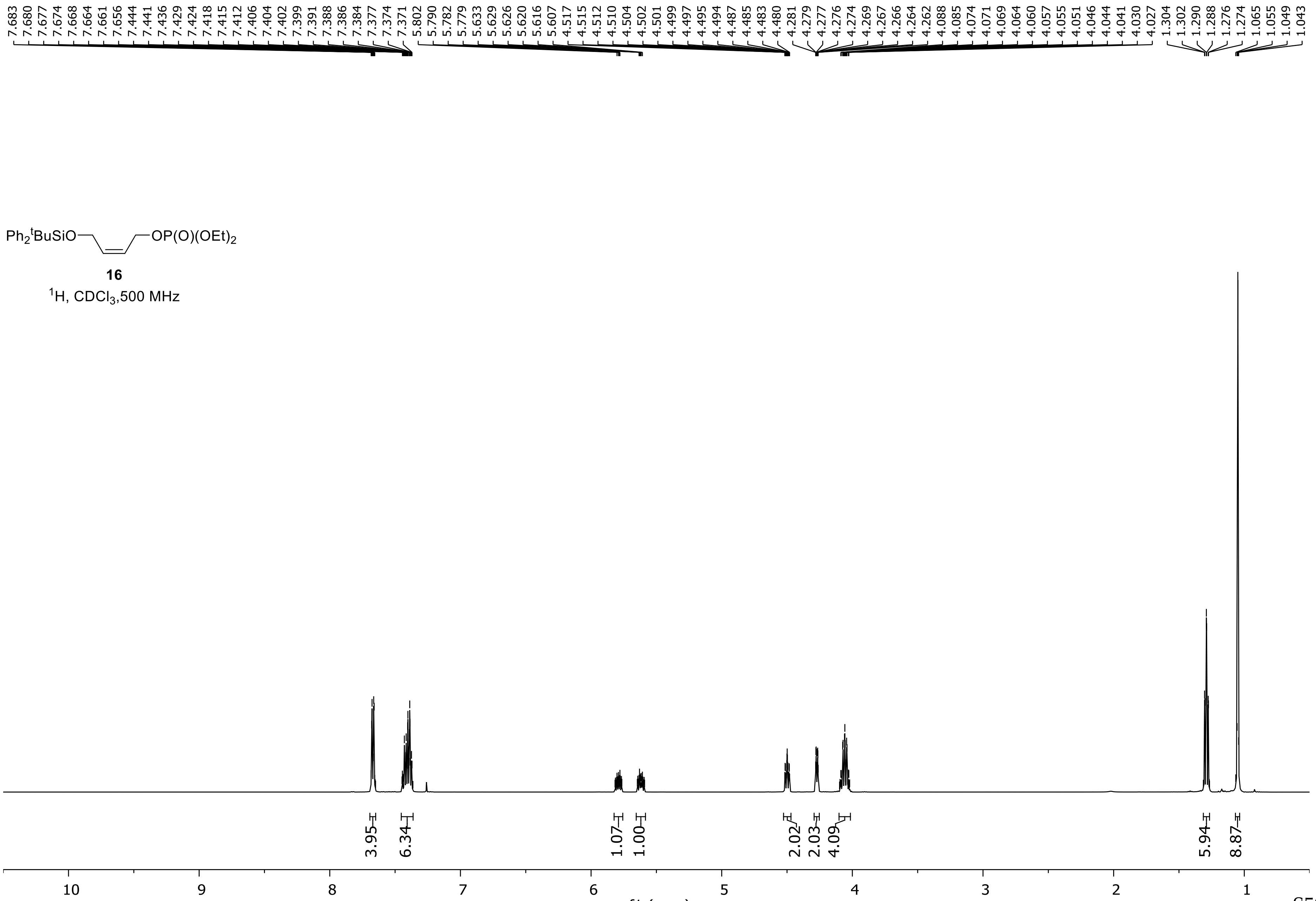

16

^1H, $CDCl_3$,500 MHz

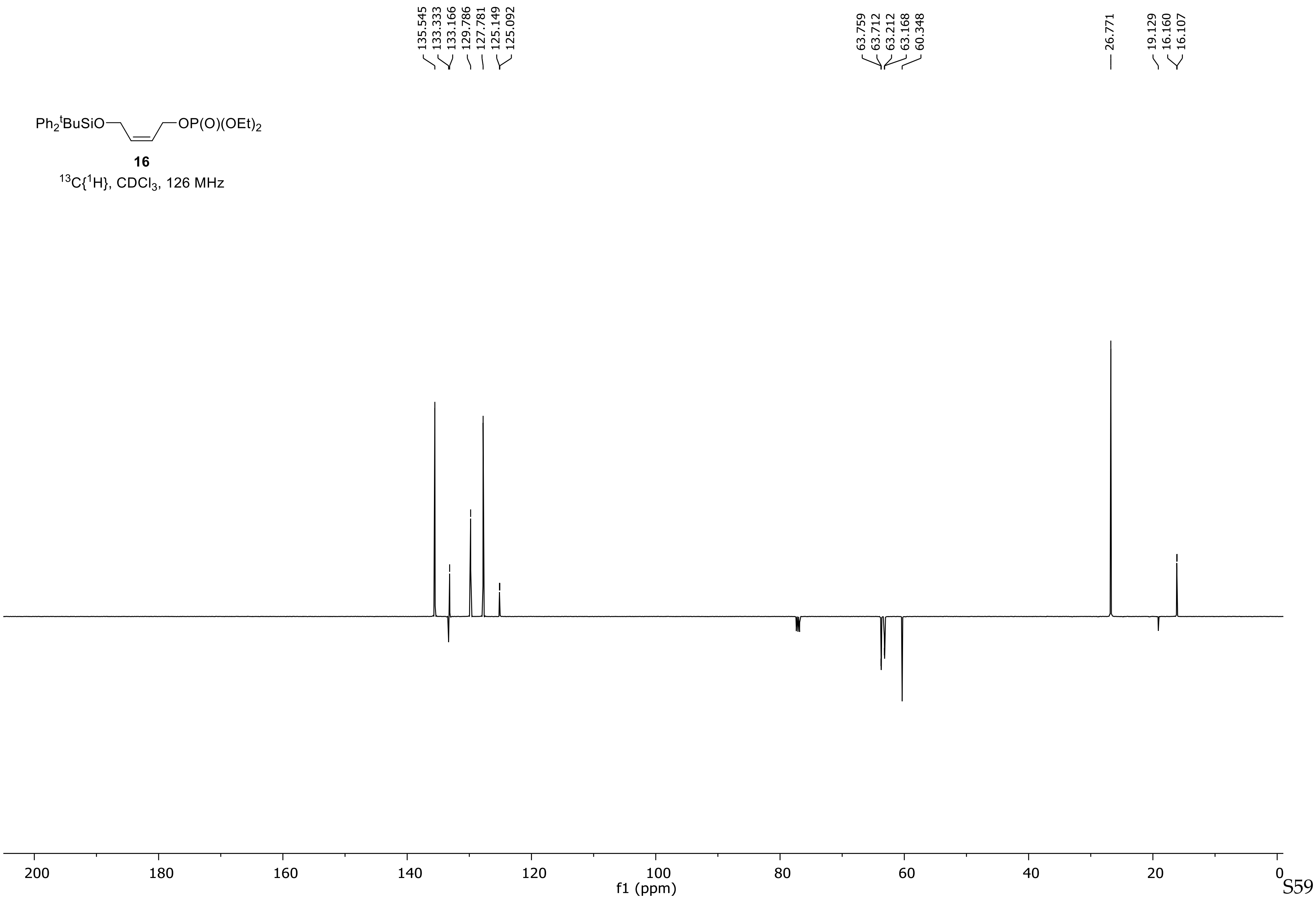

Ph2tBuSiO OP(O)(OEt)2
16
13C{1H}, CDCl3, 126 MHz
135.545
133.333
133.166
129.786
127.781
125.149
125.092
63.759
63.712
63.212
63.168
60.348
26.771
19.129
16.160
16.107
200
180
160
140
120
100
80
60
40
20
0
f1 (ppm)

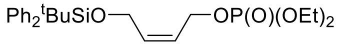

16

$^{31}P\{^{1}H\}$, $CDCl_3$, 162 MHz

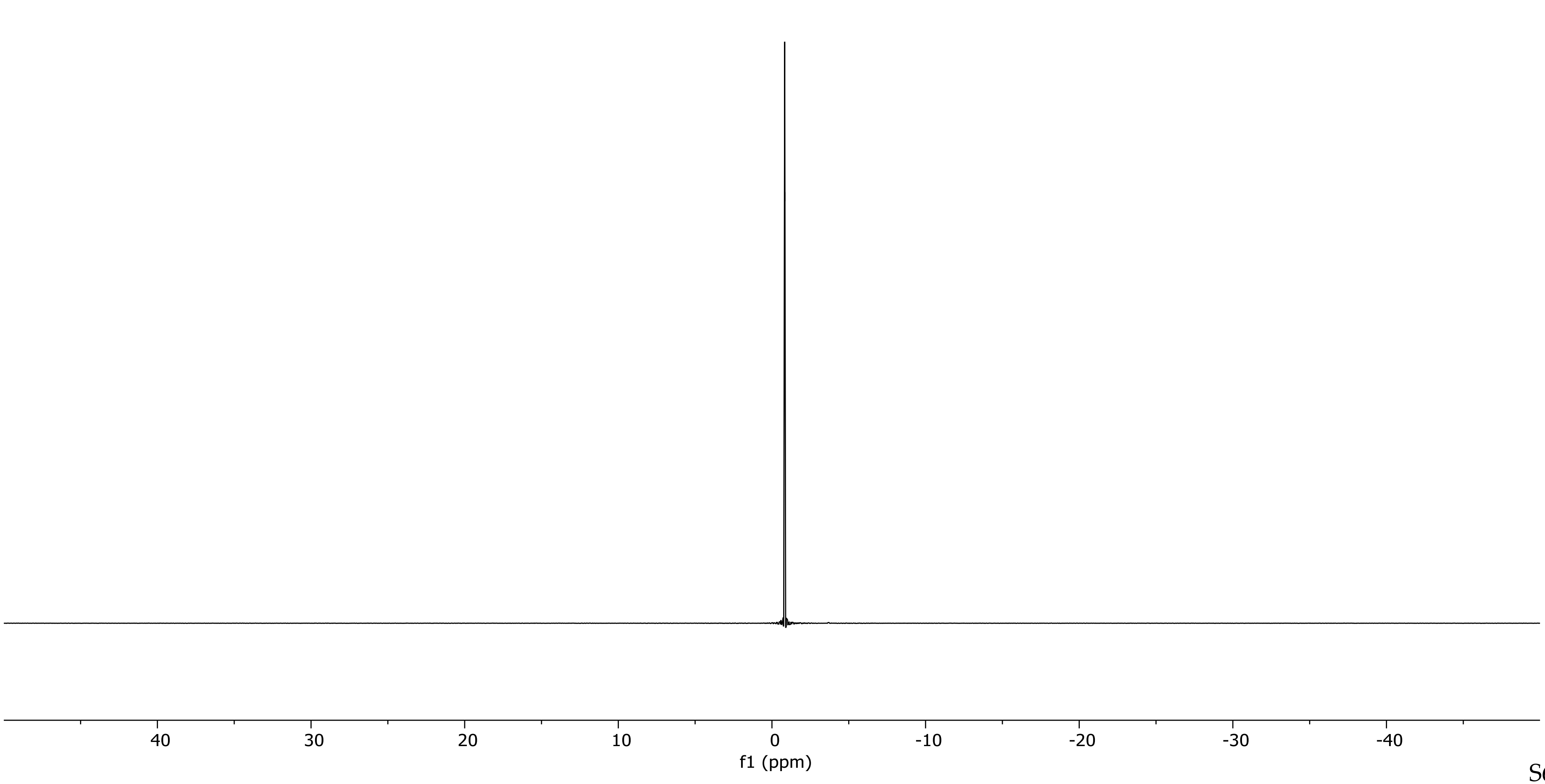

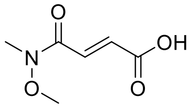

SI-7

^{1}H, $CDCl_3$,500 MHz

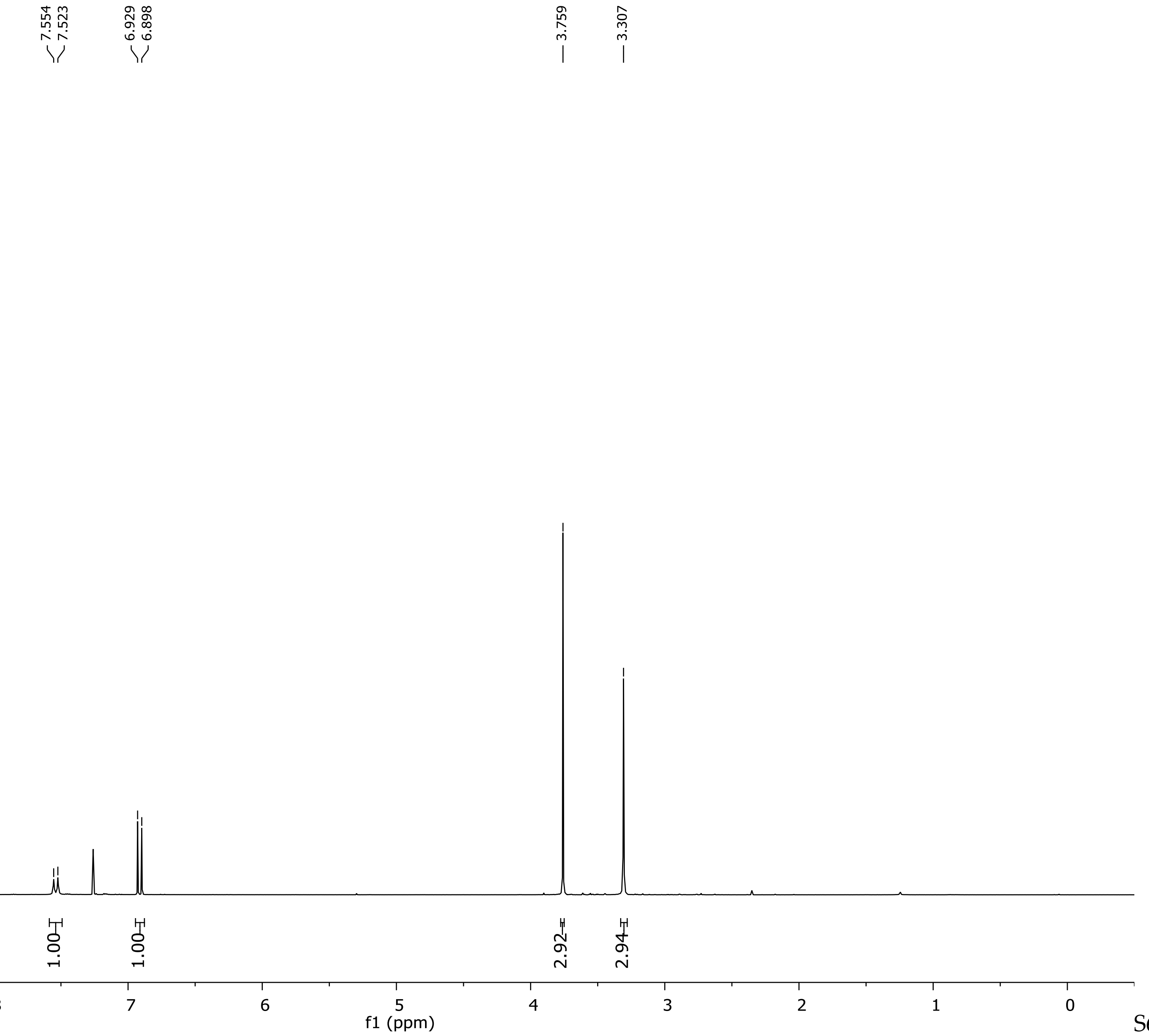

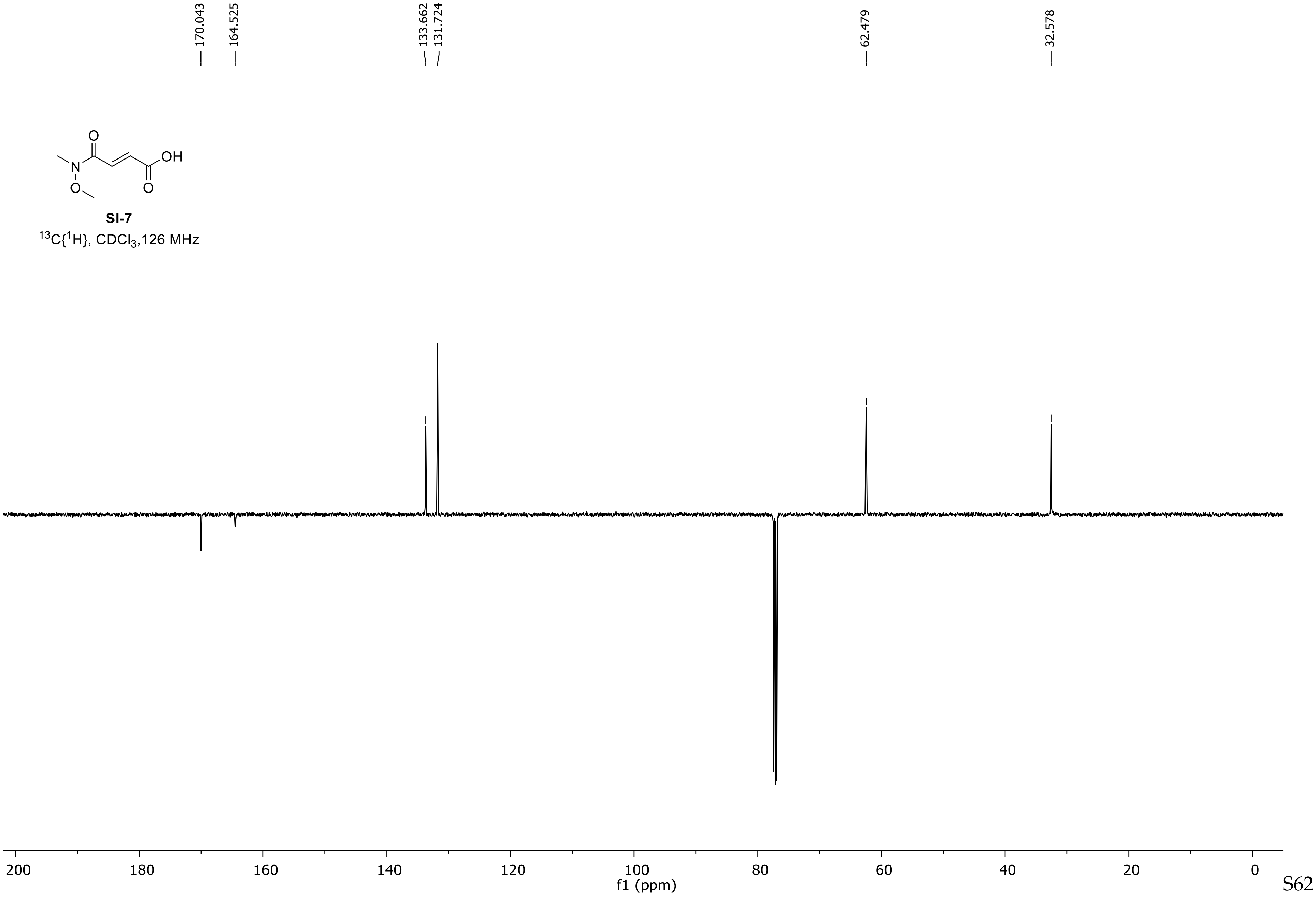

SI-7

$^{13}C\{^{1}H\}$, $CDCl_3$,126 MHz

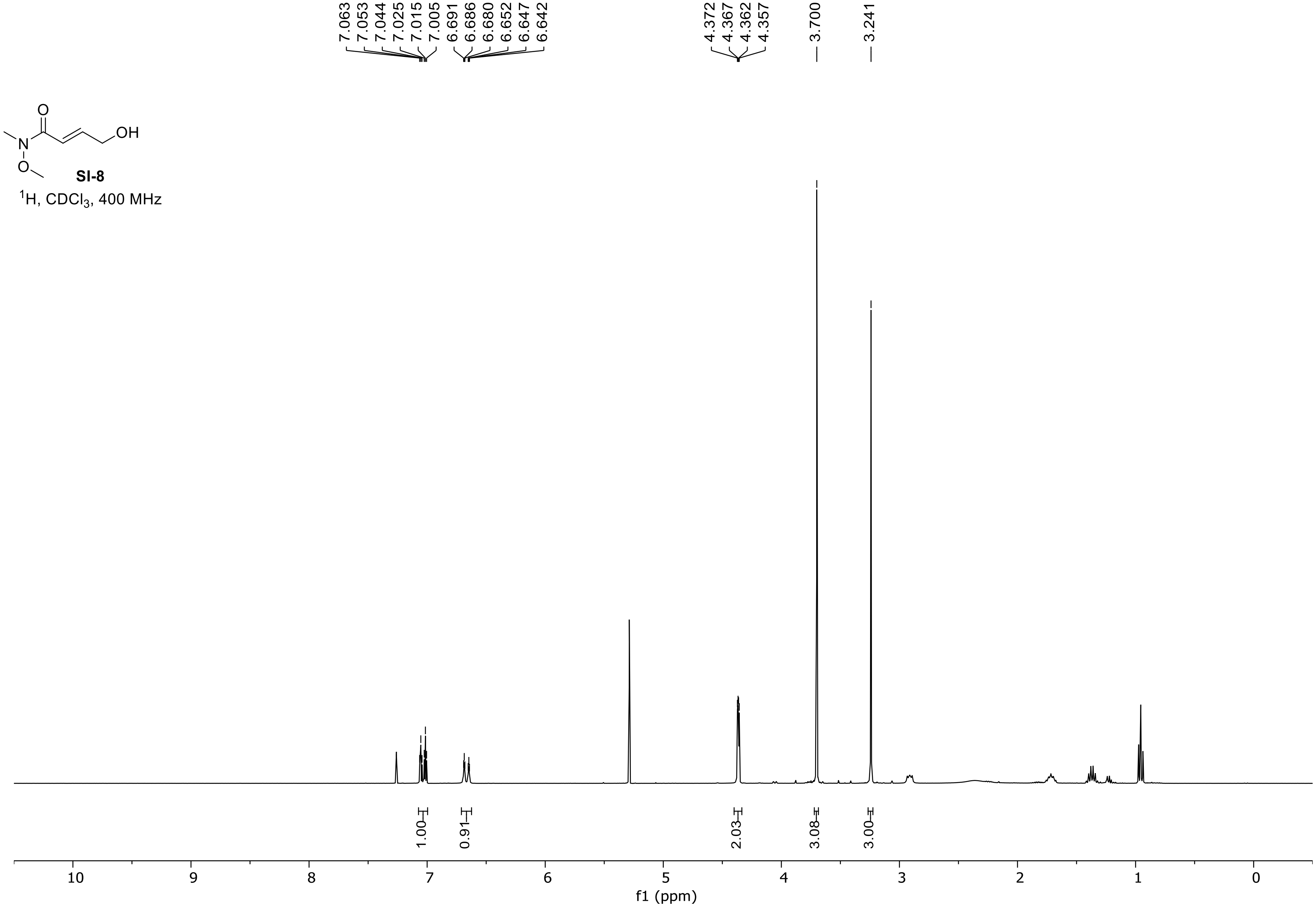

OH
O
N
SI-8
^{1}H, $CDCl_3$, 400 MHz
7.063
7.053
7.044
7.025
7.015
7.005
6.691
6.686
6.680
6.652
6.647
6.642
4.372
4.367
4.362
4.357
3.700
3.241
1.00
0.91
2.03
3.08
3.00
10
9
8
7
6
5
4
3
2
1
0
f1 (ppm)

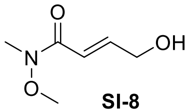

SI-8

$^{13}C\{^{1}H\}$, $CDCl_3$, 126 MHz

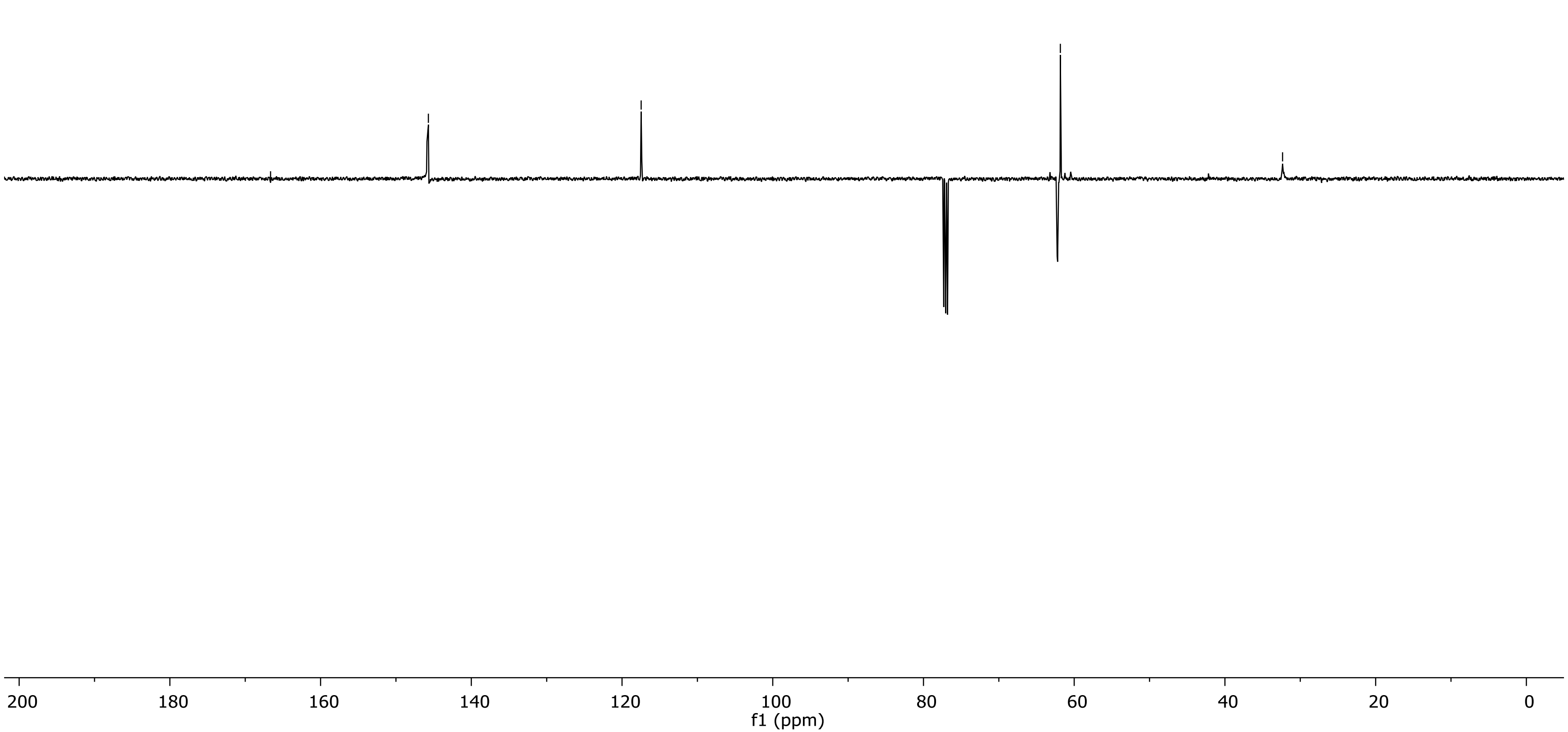

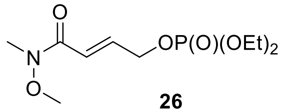

26

^{1}H, $CDCl_3$, 500 MHz

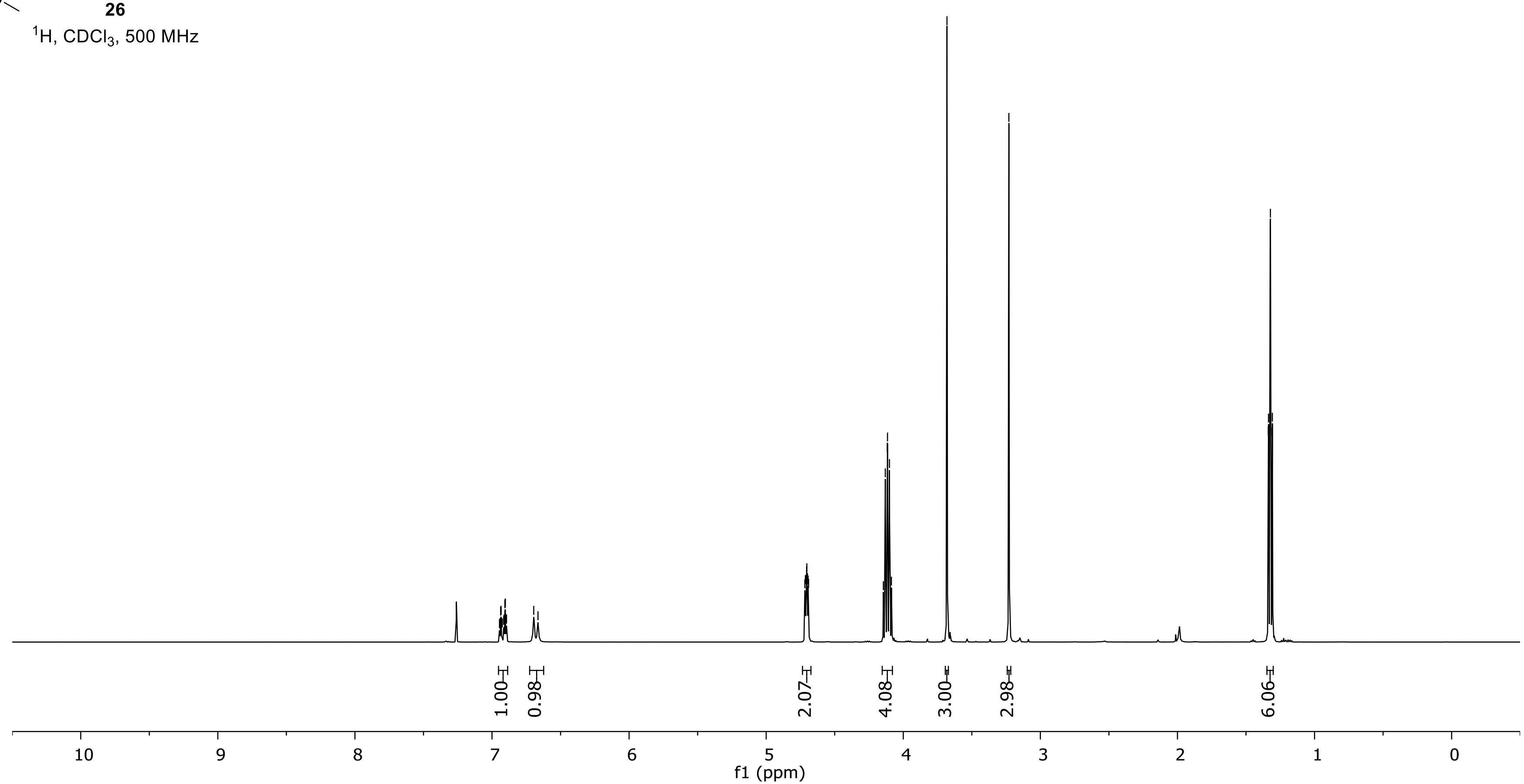

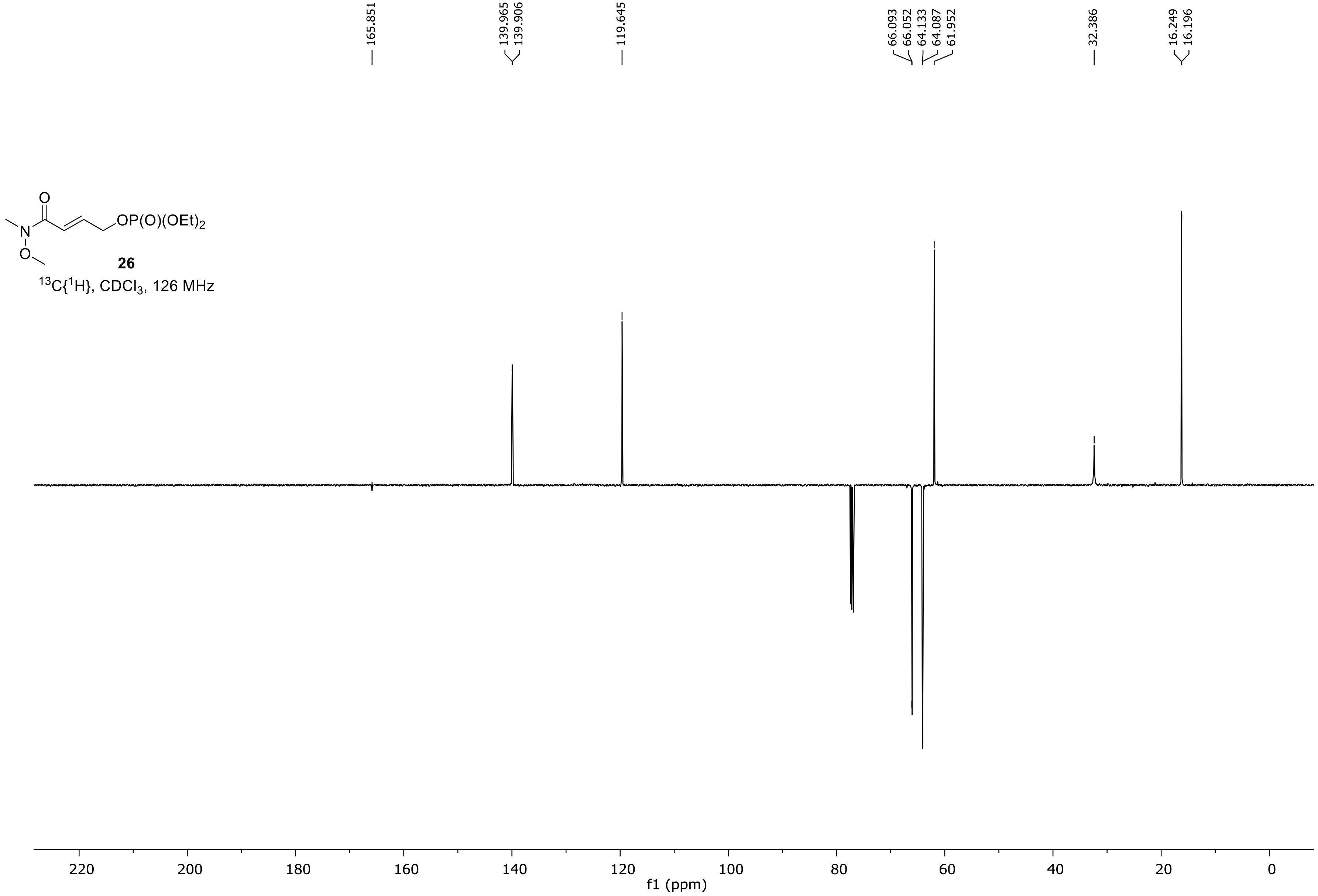
165.851
139.965
139.906
119.645
66.093
66.052
64.133
64.087
61.952
32.386
16.249
16.196
OP(O)(OEt)2
26
13C{1H}, CDCl3, 126 MHz
220 200 180 160 140 120 100 80 60 40 20 0
f1 (ppm)

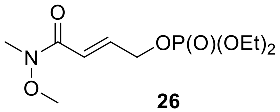

26

$^{31}P\{^1H\}$, $CDCl_3$, 162 MHz

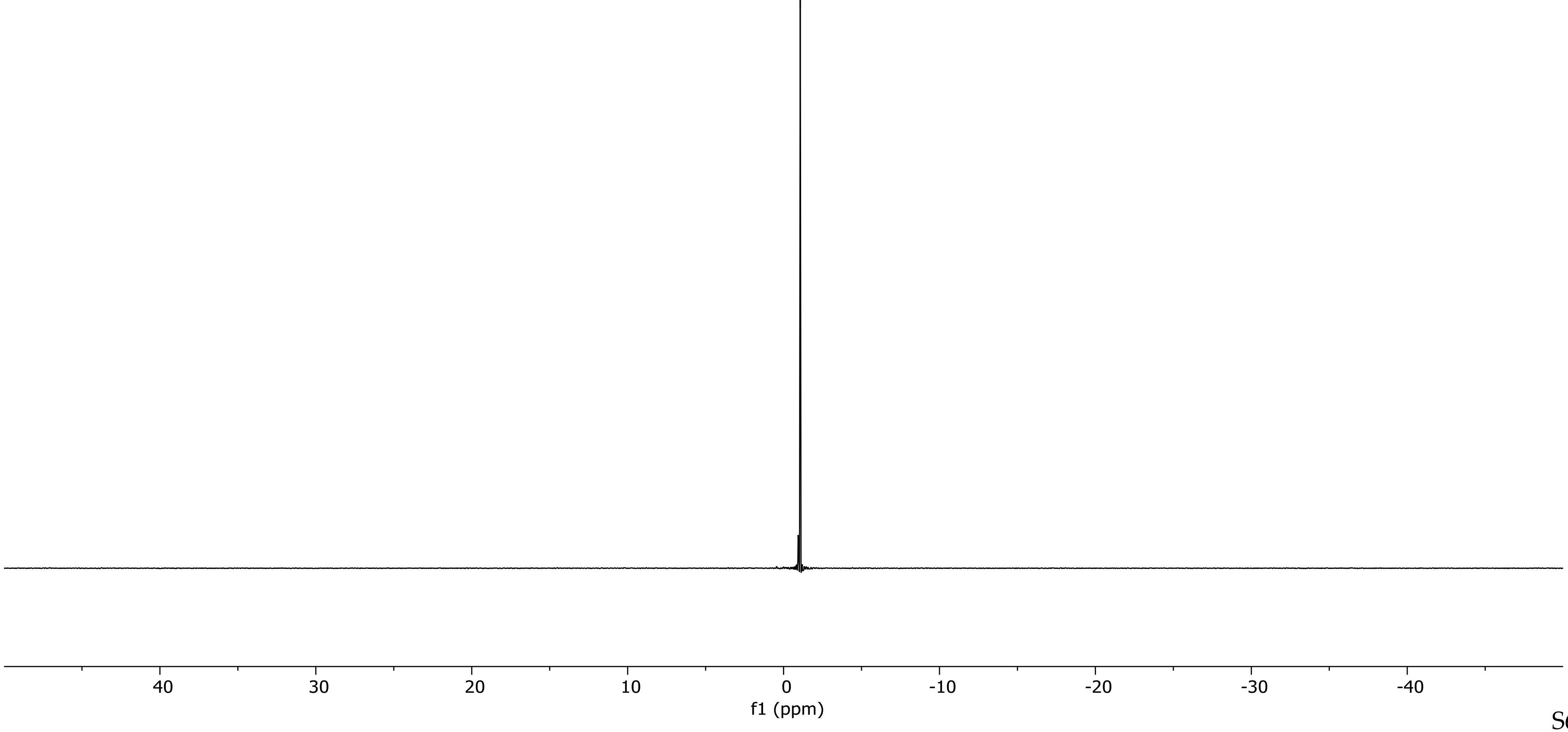

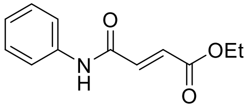

SI-9

^{1}H, $CDCl_3$, 500 MHz

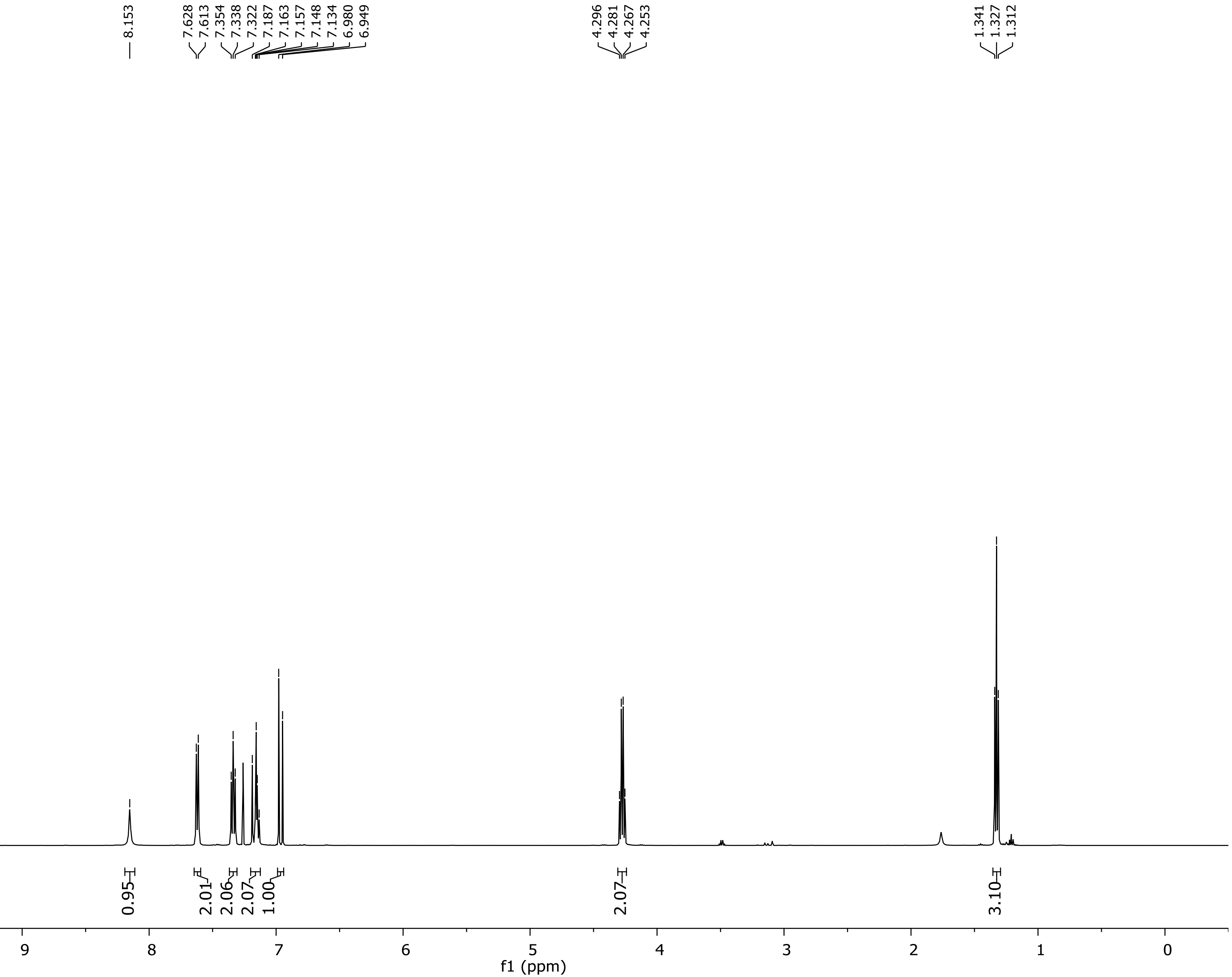

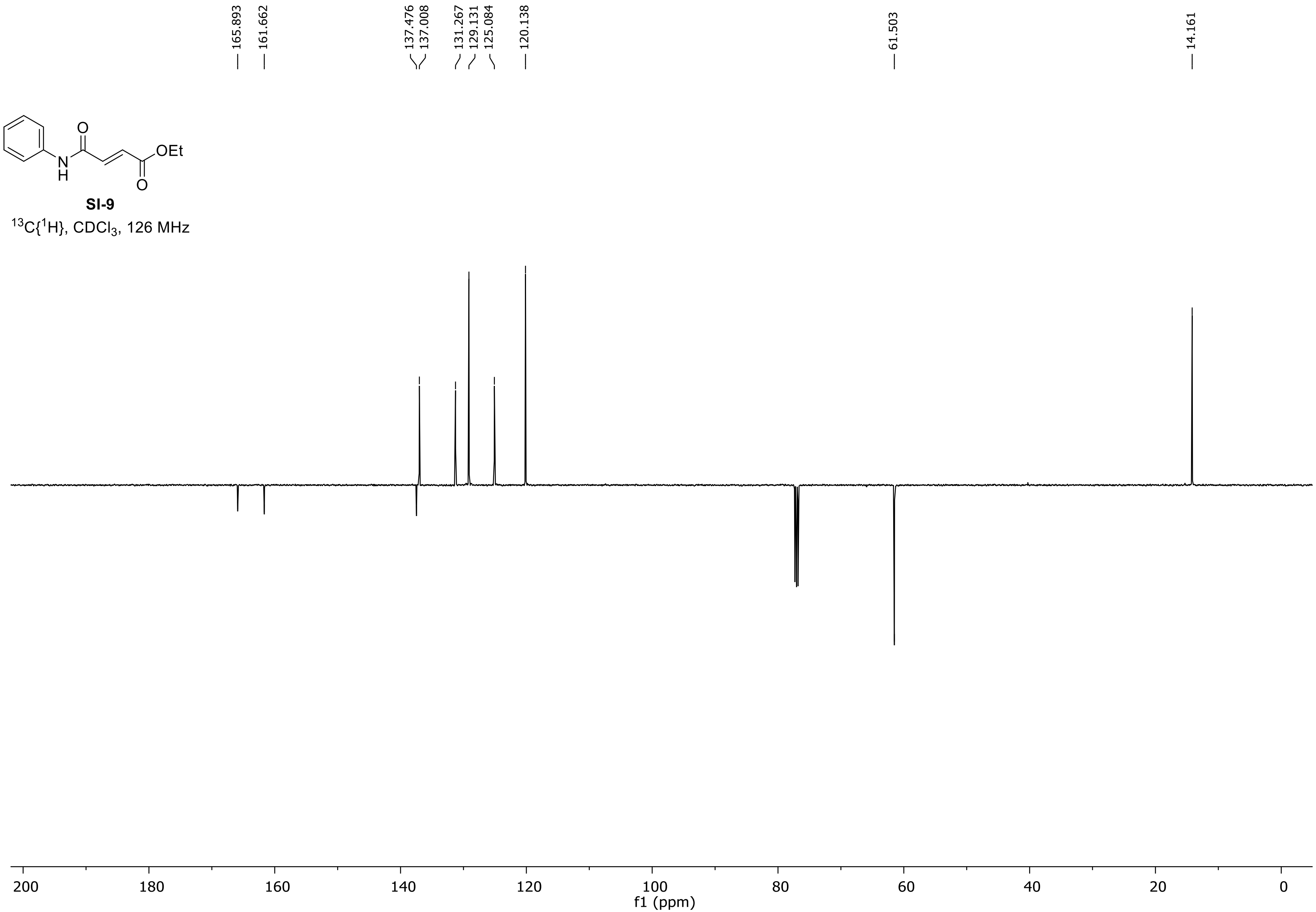

SI-9

$^{13}C\{^{1}H\}$, $CDCl_3$, 126 MHz

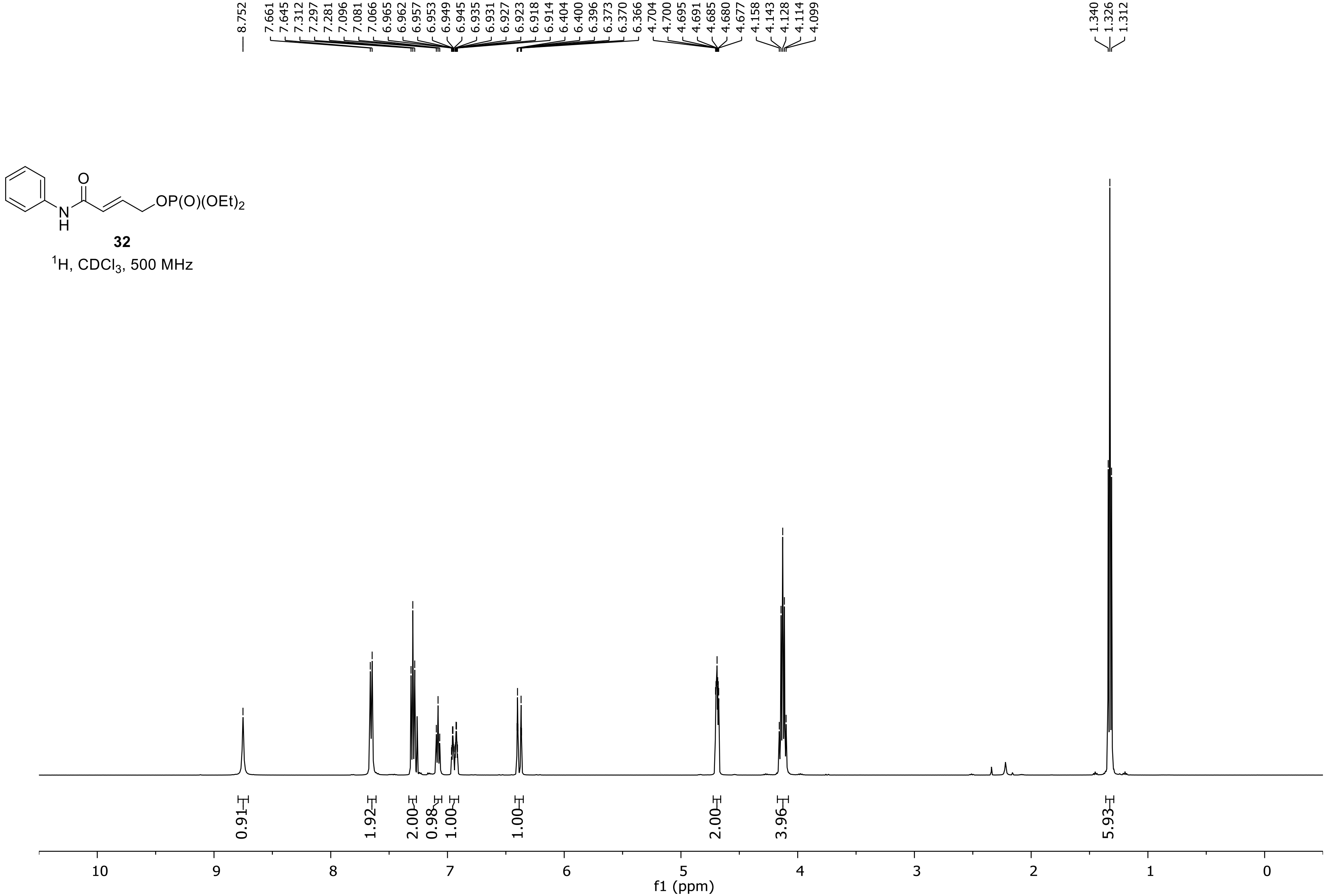
O
N
H
OP(O)(OEt)2
32
1H, CDCl3, 500 MHz
8.752
7.661
7.645
7.312
7.297
7.281
7.096
7.081
7.066
6.965
6.962
6.957
6.953
6.949
6.945
6.935
6.931
6.927
6.923
6.918
6.914
6.404
6.400
6.396
6.373
6.370
6.366
4.704
4.700
4.695
4.691
4.685
4.680
4.677
4.158
4.143
4.128
4.114
4.099
1.340
1.326
1.312
0.91
1.92
2.00
0.98
1.00
1.00
2.00
3.96
5.93
10
9
8
7
6
5
4
3
2
1
0
f1 (ppm)

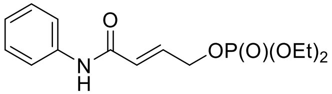

32

$^{13}C\{^1H\}$, $CDCl_3$, 126 MHz

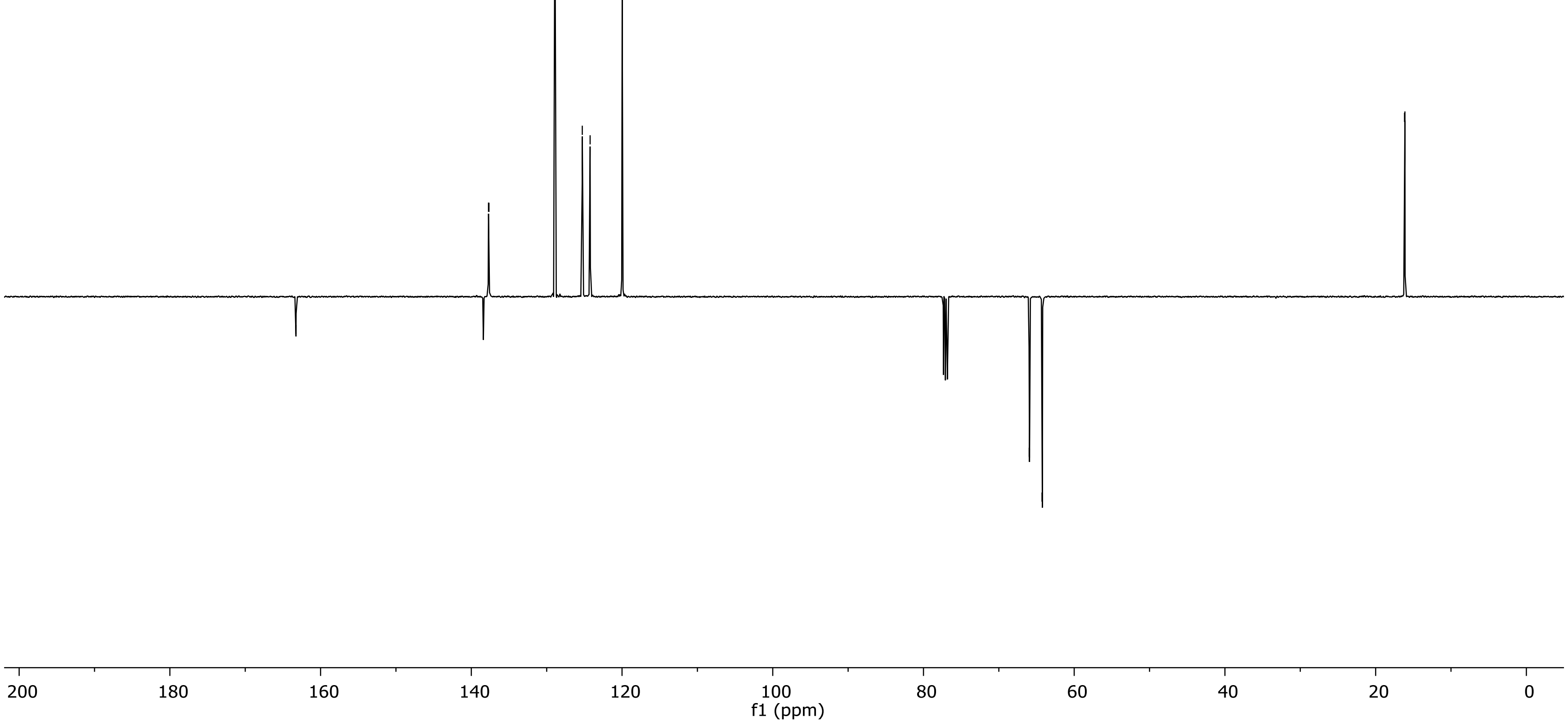

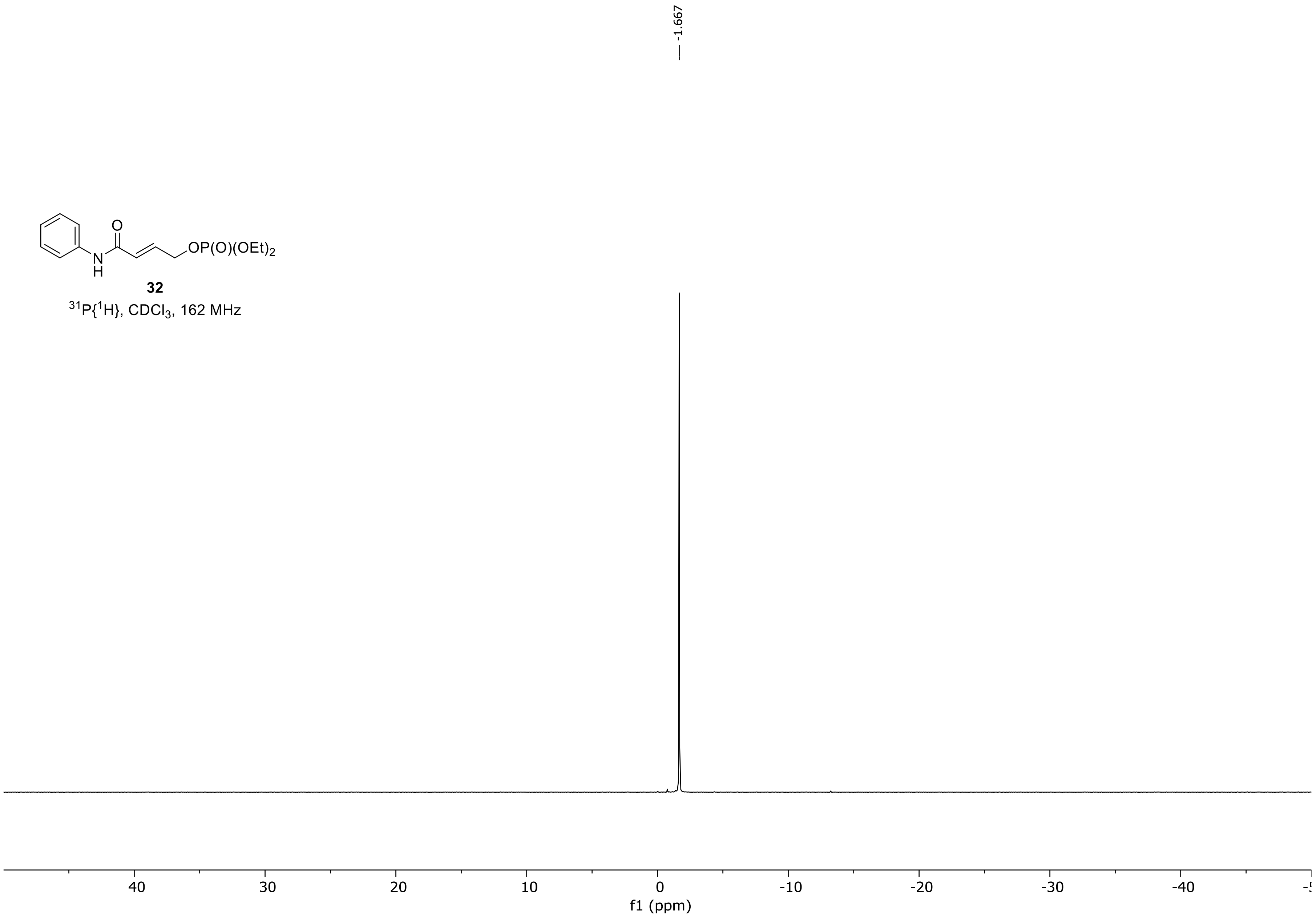

32

$^{31}P\{^{1}H\}$, $CDCl_3$, 162 MHz

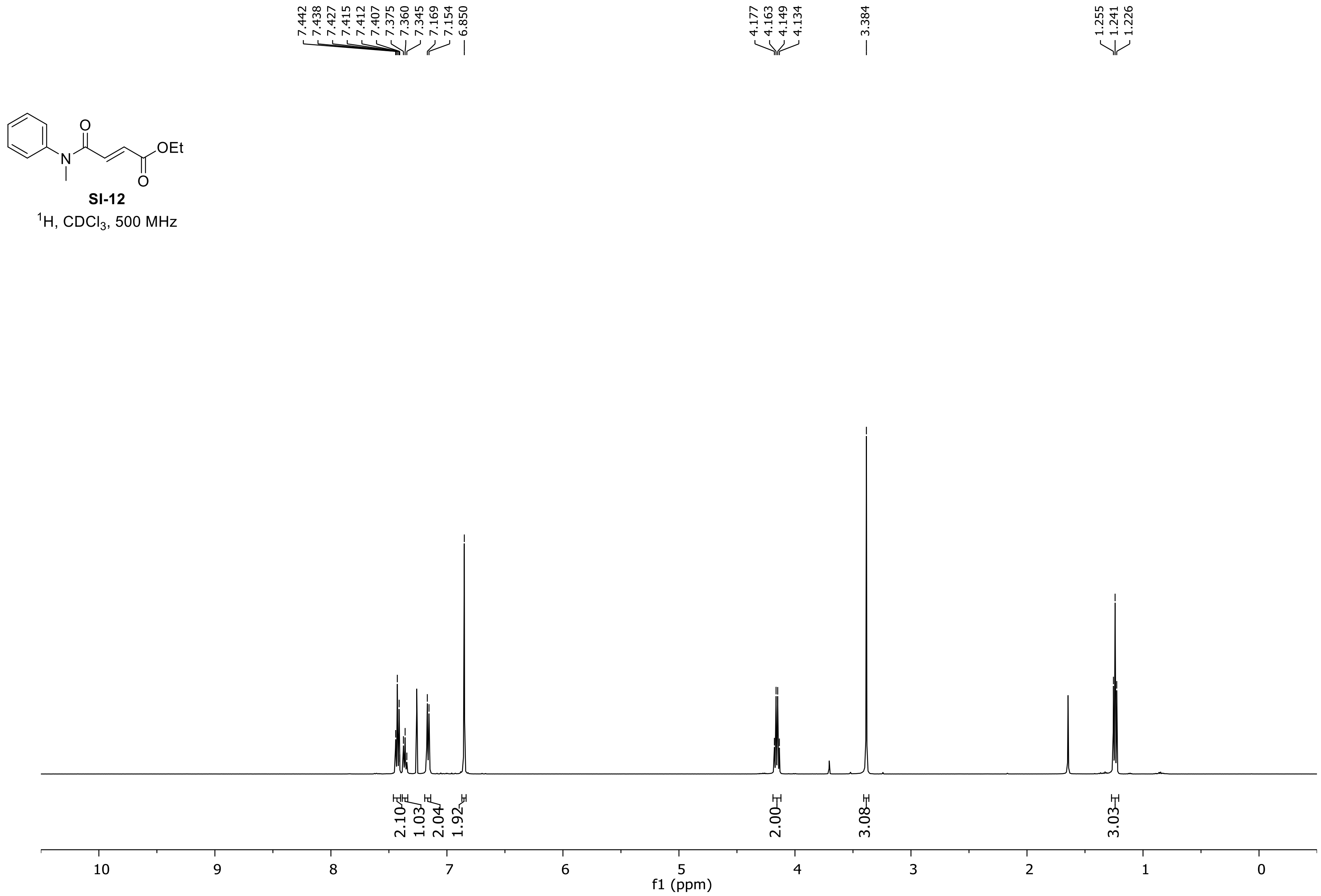
7.442
7.438
7.427
7.415
7.412
7.407
7.375
7.360
7.345
7.169
7.154
6.850
4.177
4.163
4.149
4.134
3.384
1.255
1.241
1.226
O
N
OEt
O
SI-12
¹H, CDCl₃, 500 MHz
2.10
1.03
2.04
1.92
2.00
3.08
3.03
10
9
8
7
6
5
4
3
2
1
0
f1 (ppm)

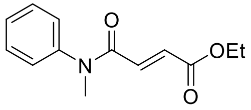

SI-12

$^{13}C\{^{1}H\}$, $CDCl_3$, 126 MHz

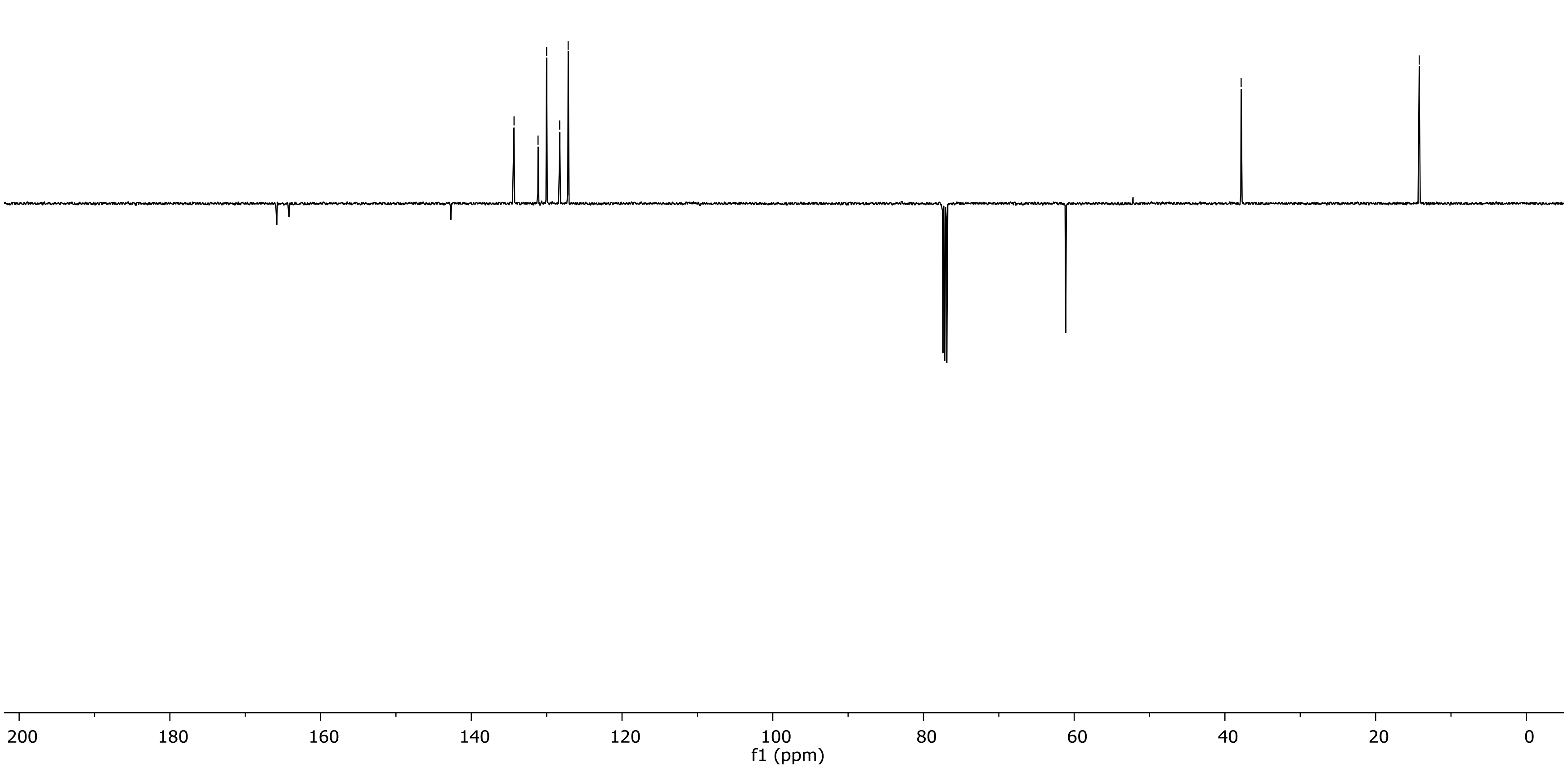

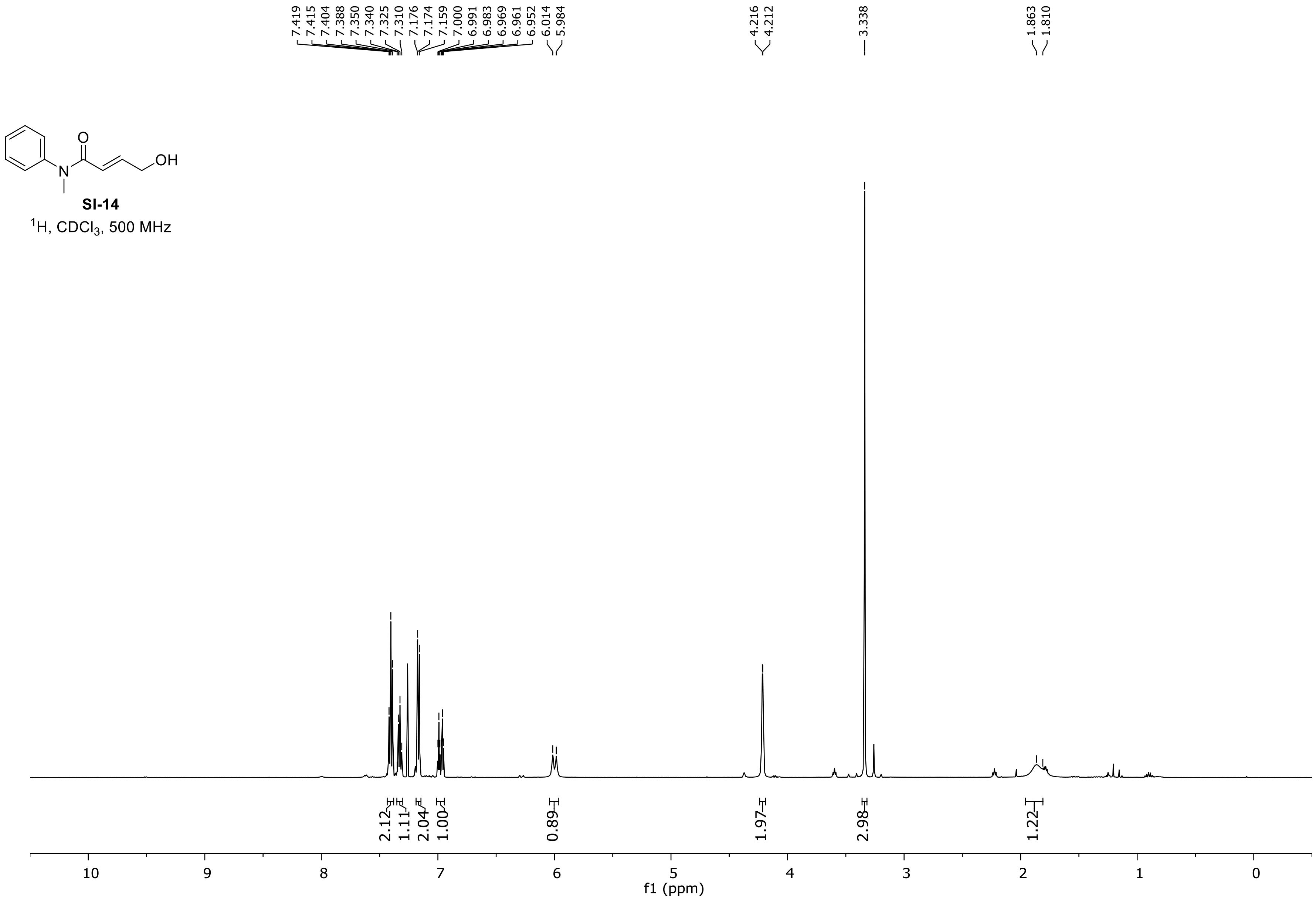
SI-14
^{1}H, CDCl$_3$, 500 MHz
7.419
7.415
7.404
7.388
7.350
7.340
7.325
7.310
7.176
7.174
7.159
7.000
6.991
6.983
6.969
6.961
6.952
6.014
5.984
4.216
4.212
3.338
1.863
1.810
2.12
1.11
2.04
1.00
0.89
1.97
2.98
1.22
f1 (ppm)

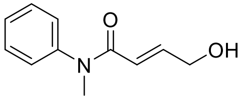

SI-14

$^{13}C\{^{1}H\}$, $CDCl_3$, 126 MHz

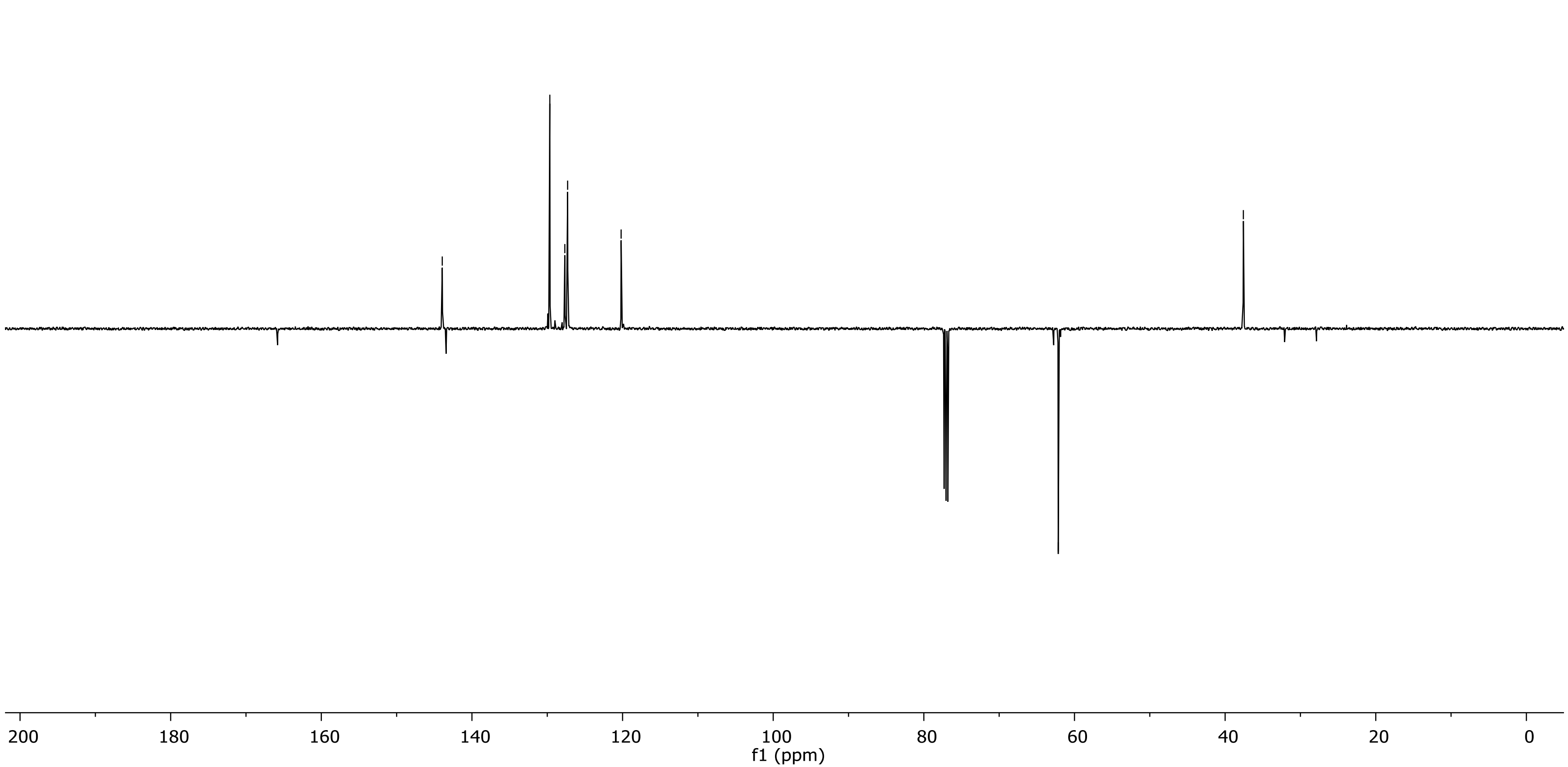

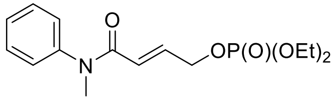

33

^{1}H, $CDCl_3$, 500 MHz

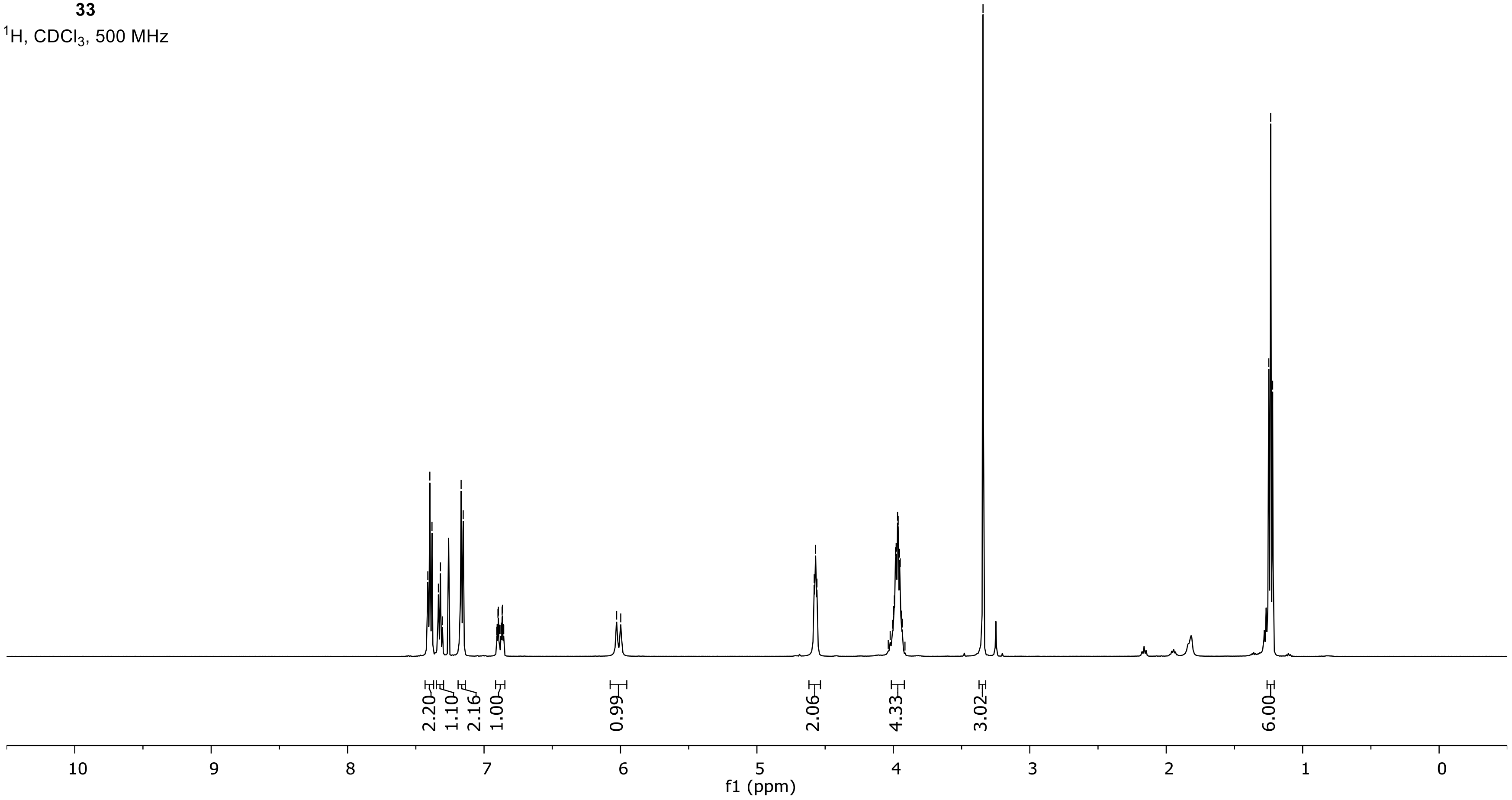

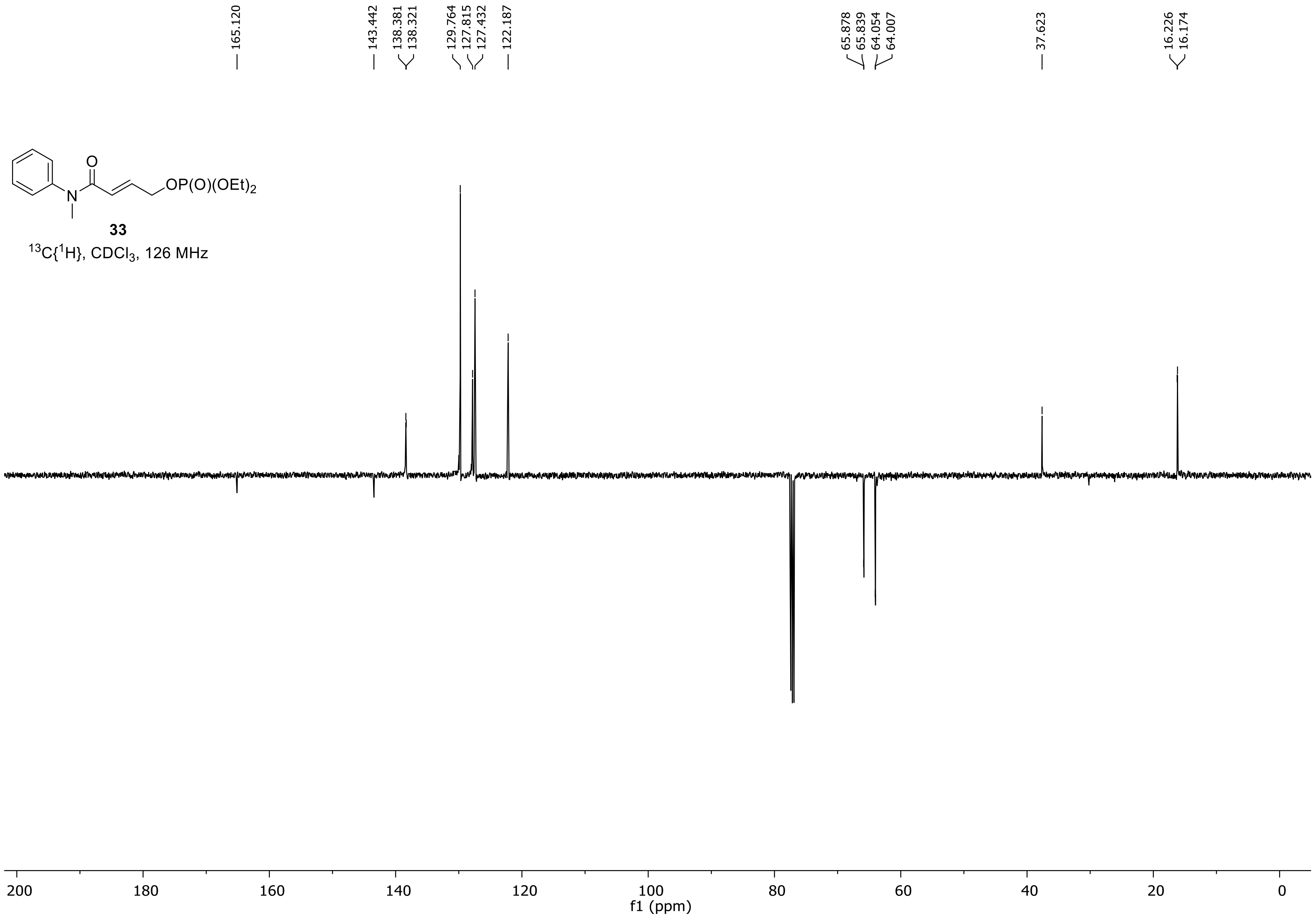
165.120
143.442
138.381
138.321
129.764
127.815
127.432
122.187
65.878
65.839
64.054
64.007
37.623
16.226
16.174
OP(O)(OEt)2
N
O
33
13C{1H}, CDCl3, 126 MHz
200
180
160
140
120
100
80
60
40
20
0
f1 (ppm)

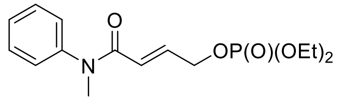

33

$^{31}P\{^{1}H\}$, $CDCl_3$, 202 MHz

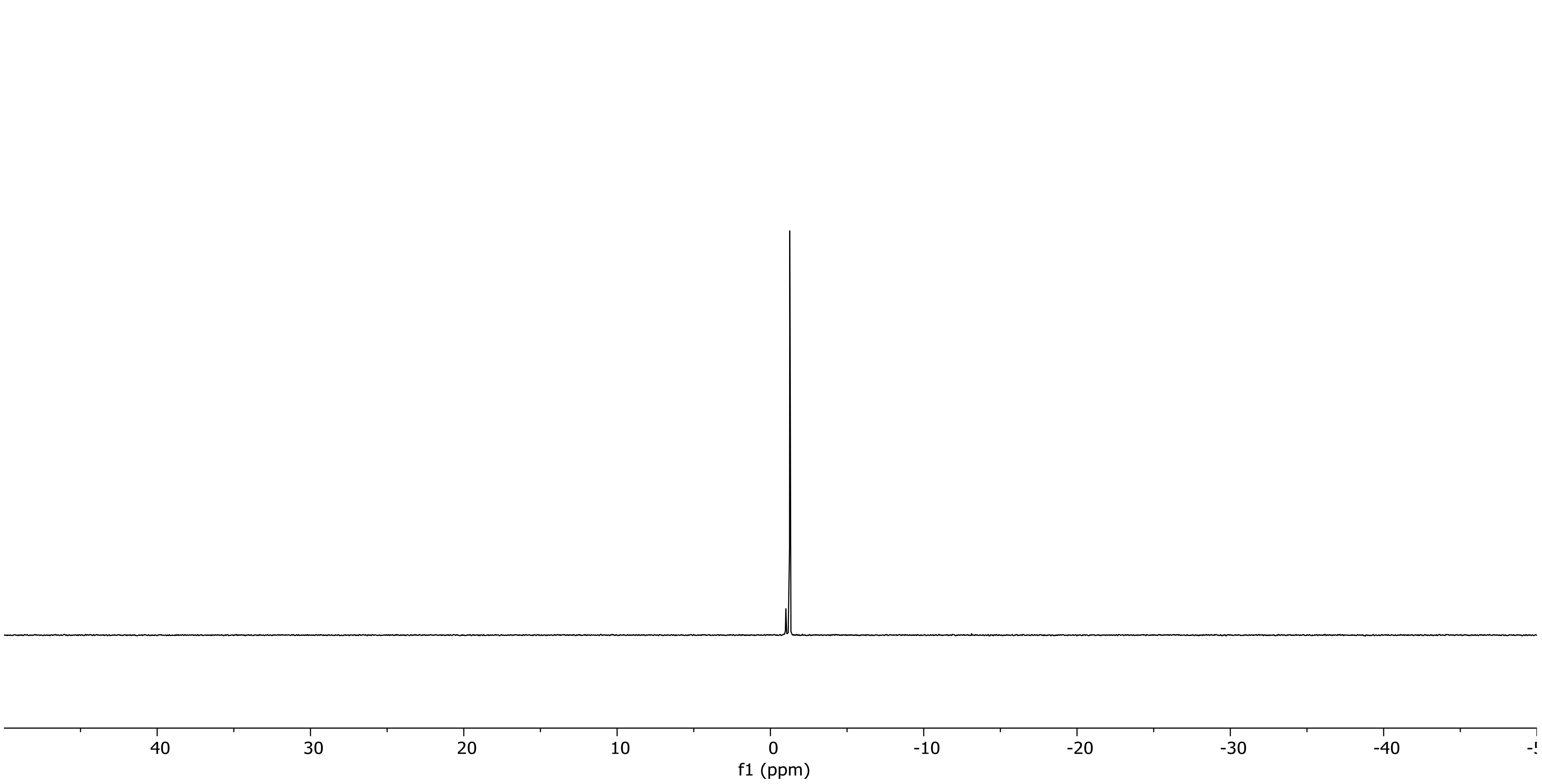

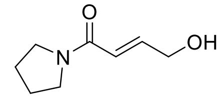

SI-20

^{1}H, $CDCl_3$, 500 MHz

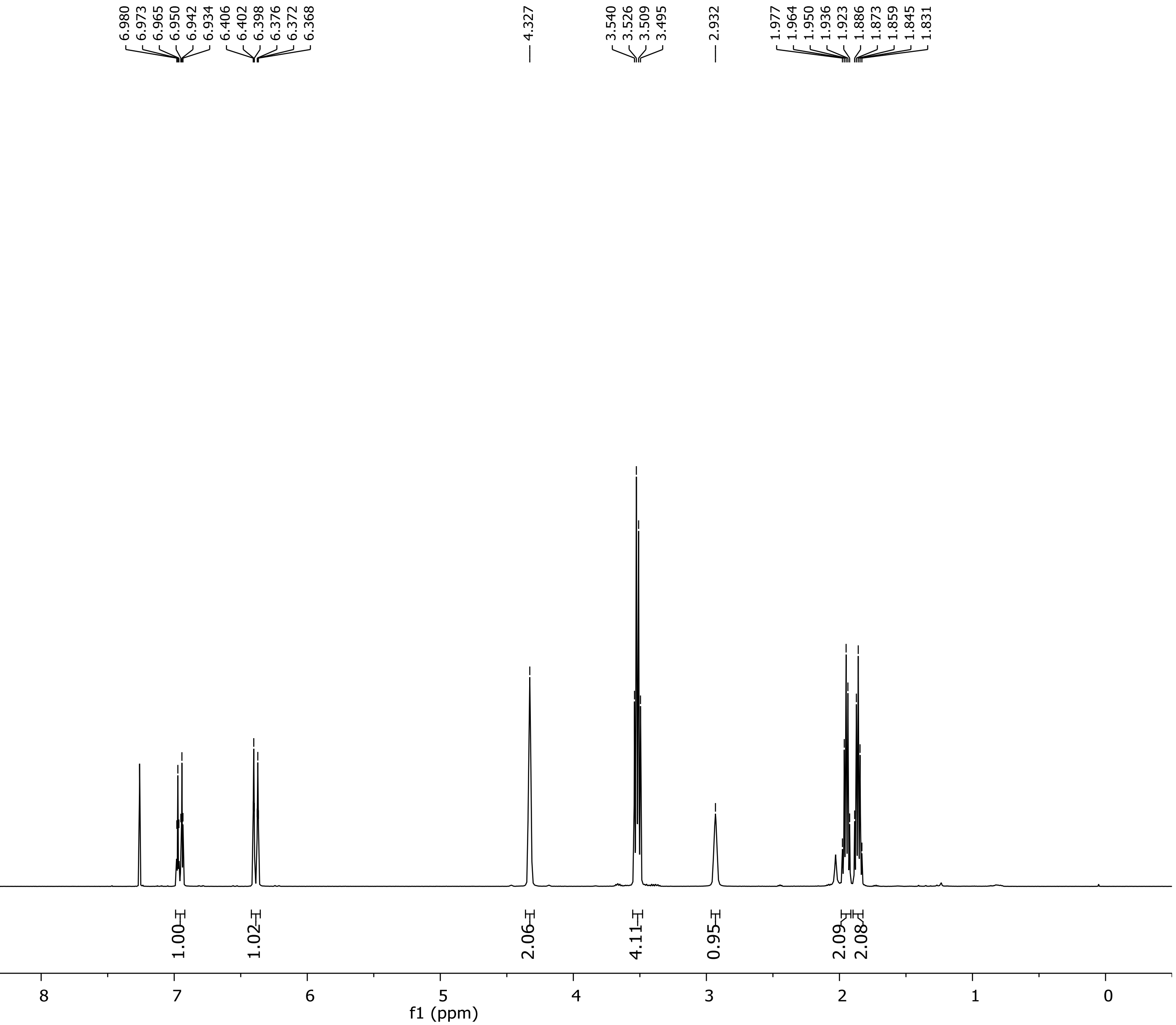

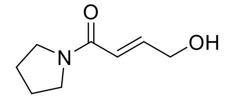

SI-20
$^{13}C\{^{1}H\}$, $CDCl_3$, 126 MHz

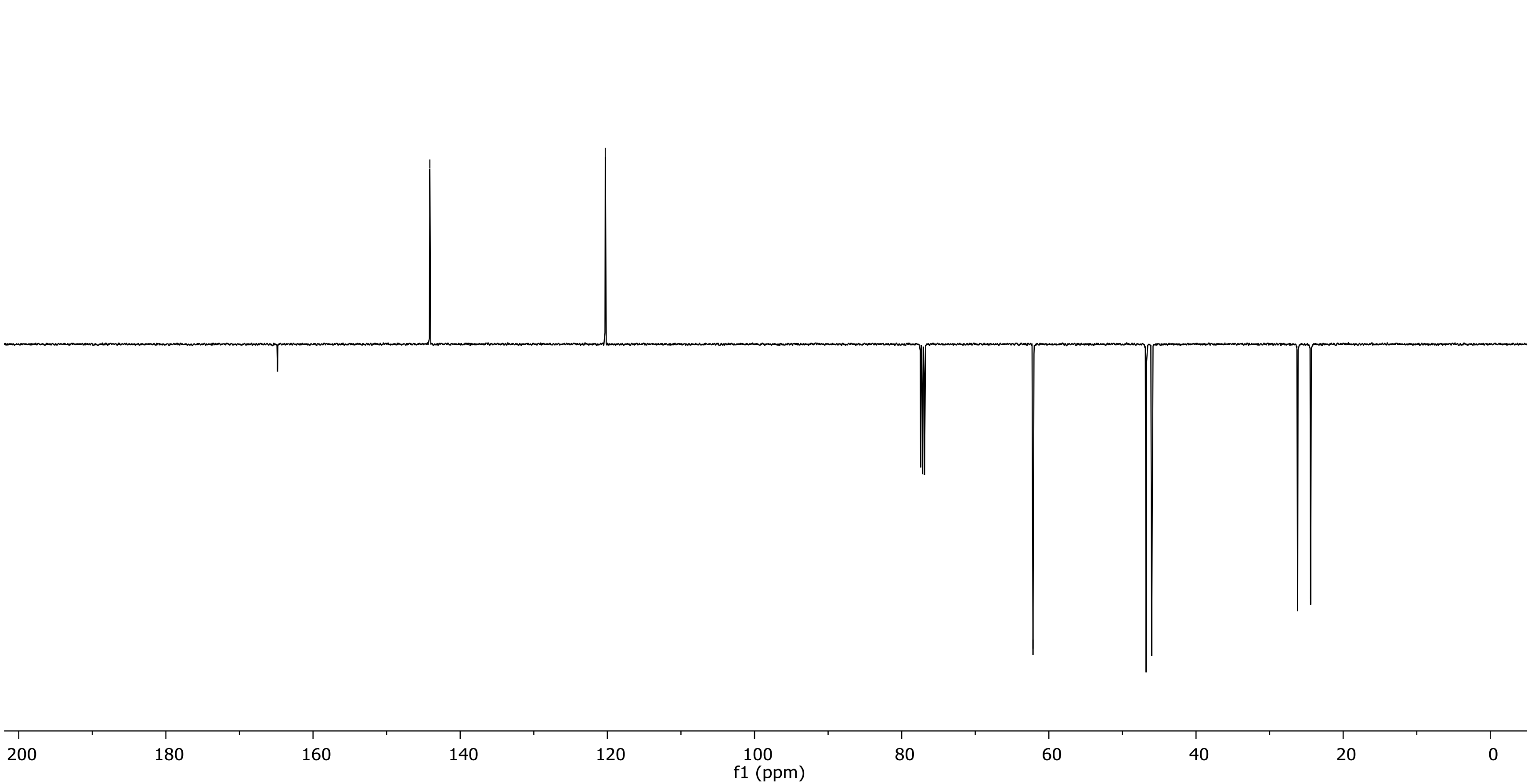

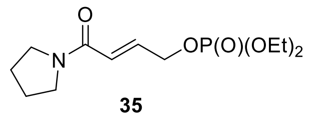

35

^1H, $CDCl_3$, 500 MHz

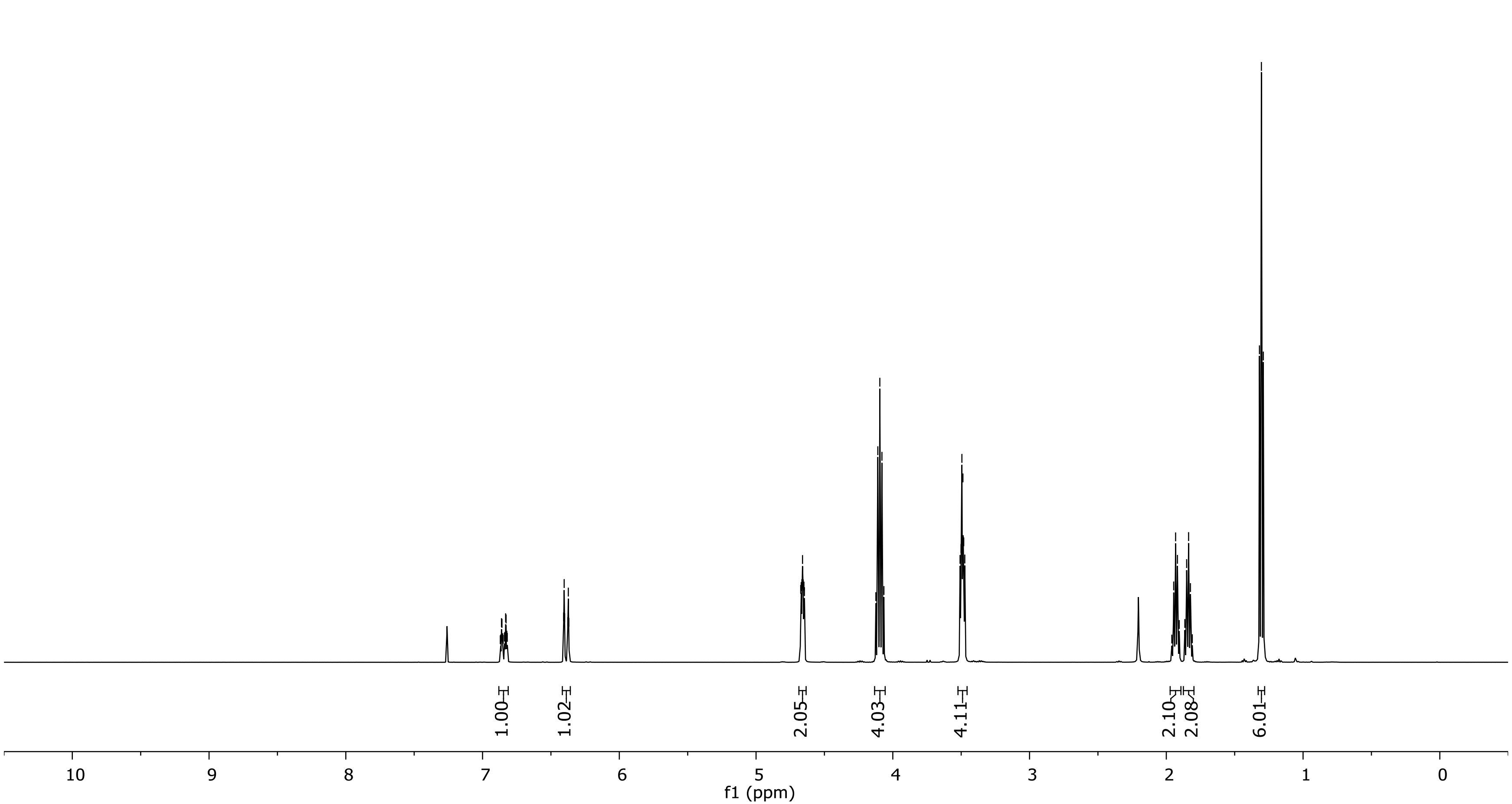

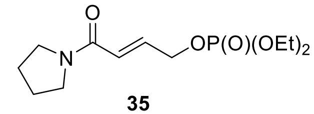

35

$^{13}C\{^{1}H\}$, $CDCl_3$, 126 MHz

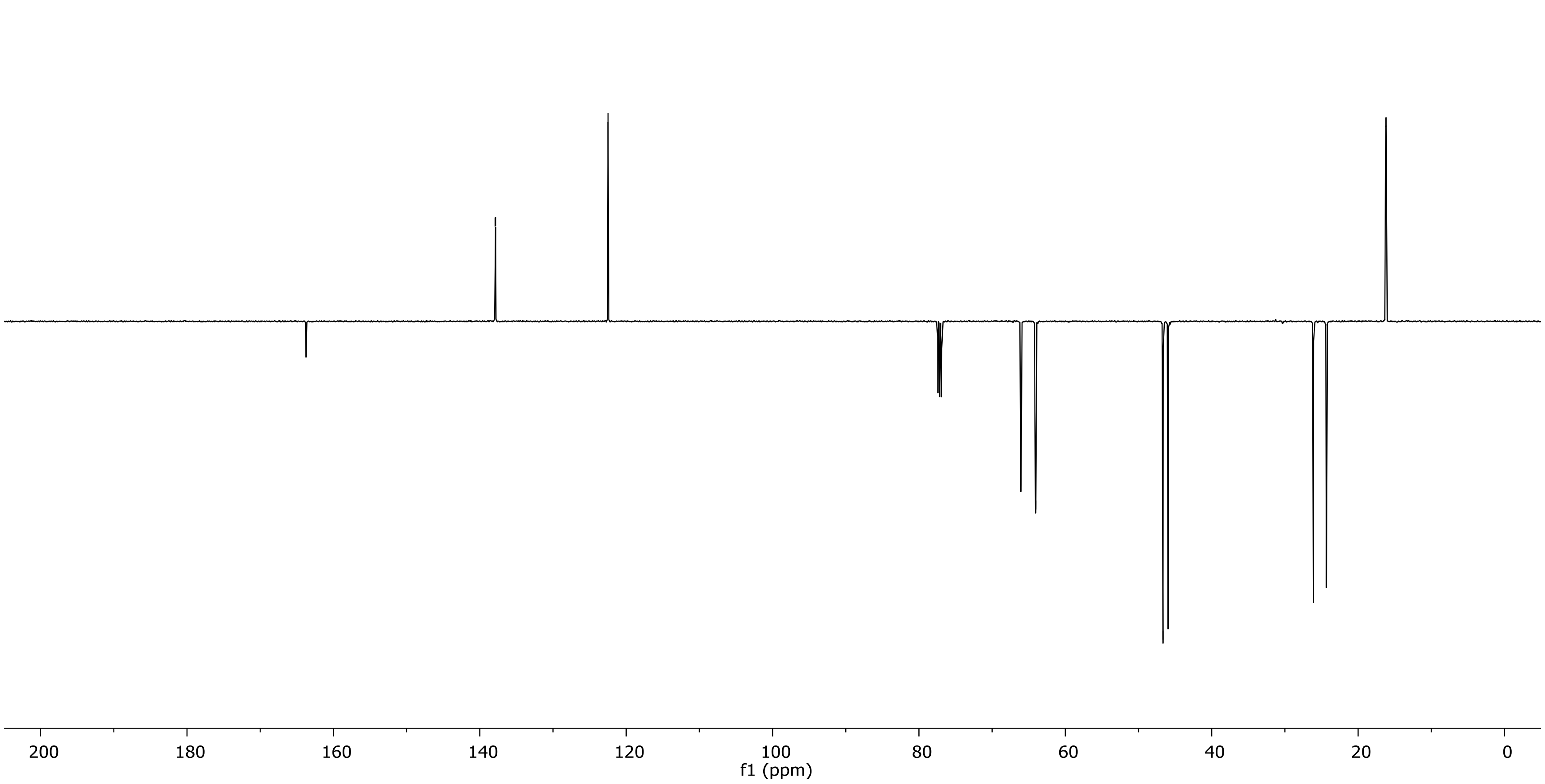

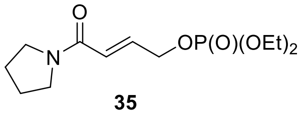

35

$^{31}P\{^1H\}$, $CDCl_3$, 202 MHz

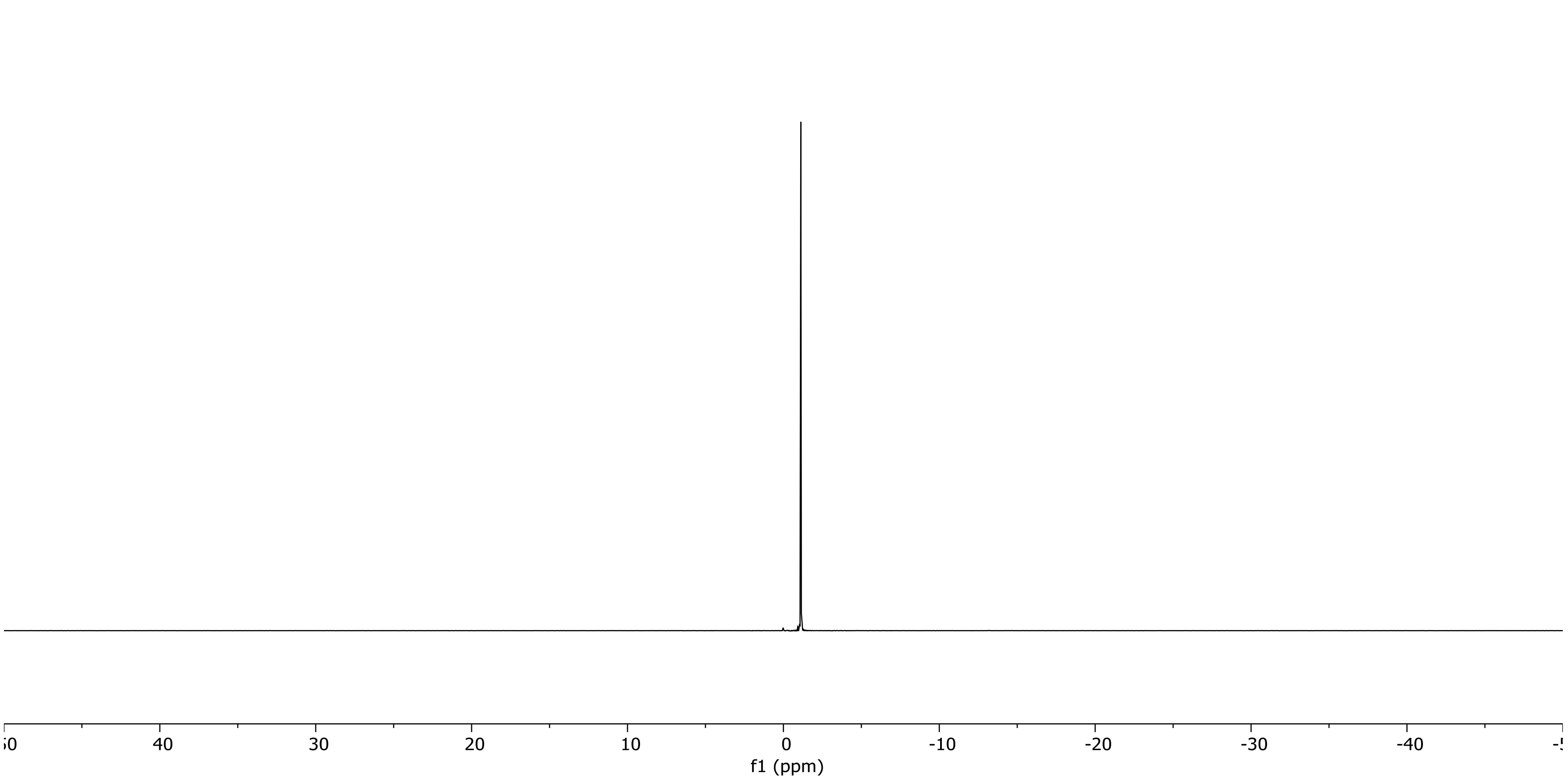

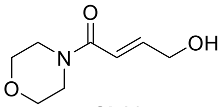

SI-23

^{1}H, $CDCl_3$, 500 MHz

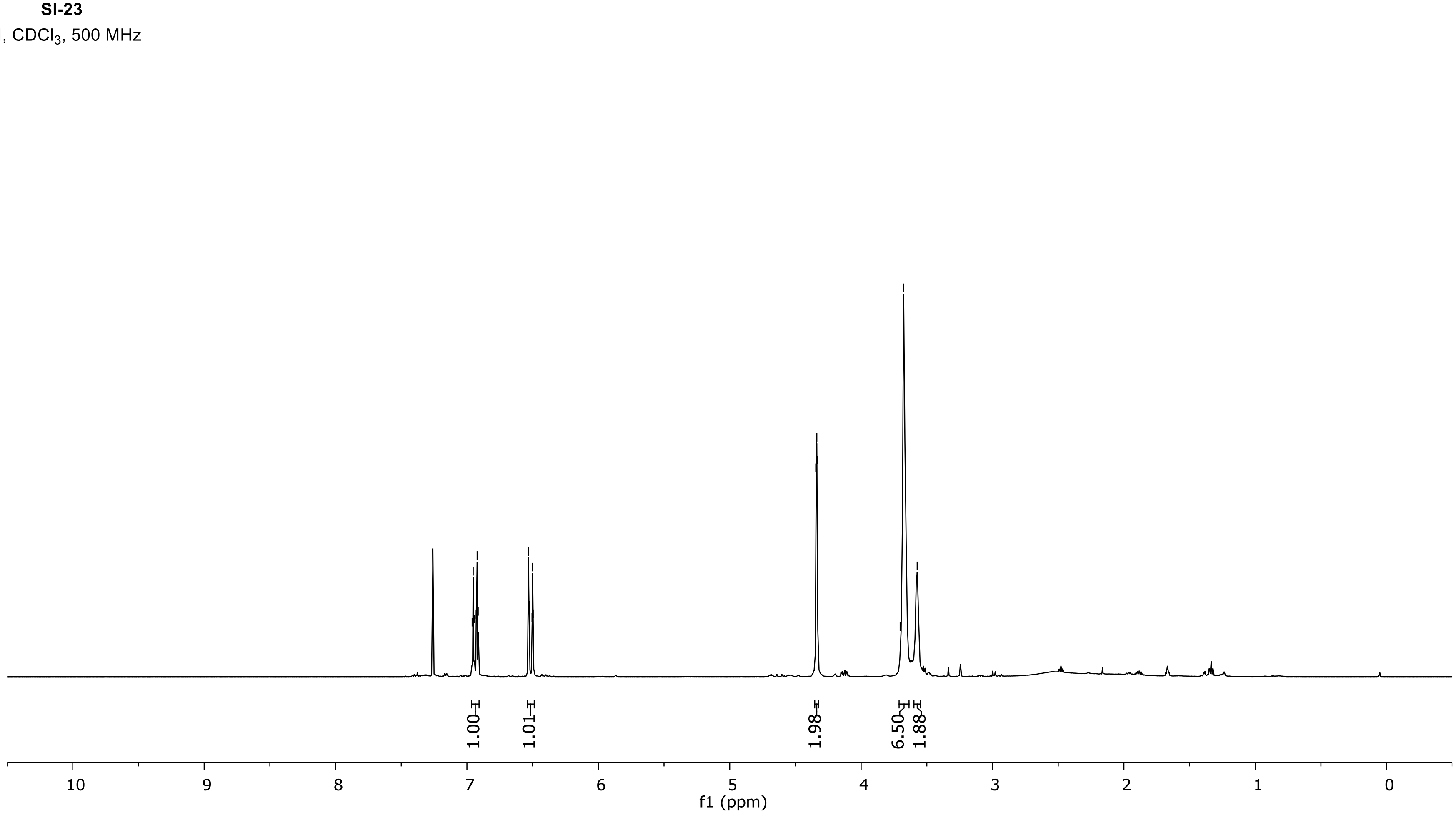

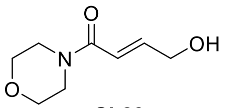

SI-23

$^{13}C\{^1H\}$, $CDCl_3$, 126 MHz

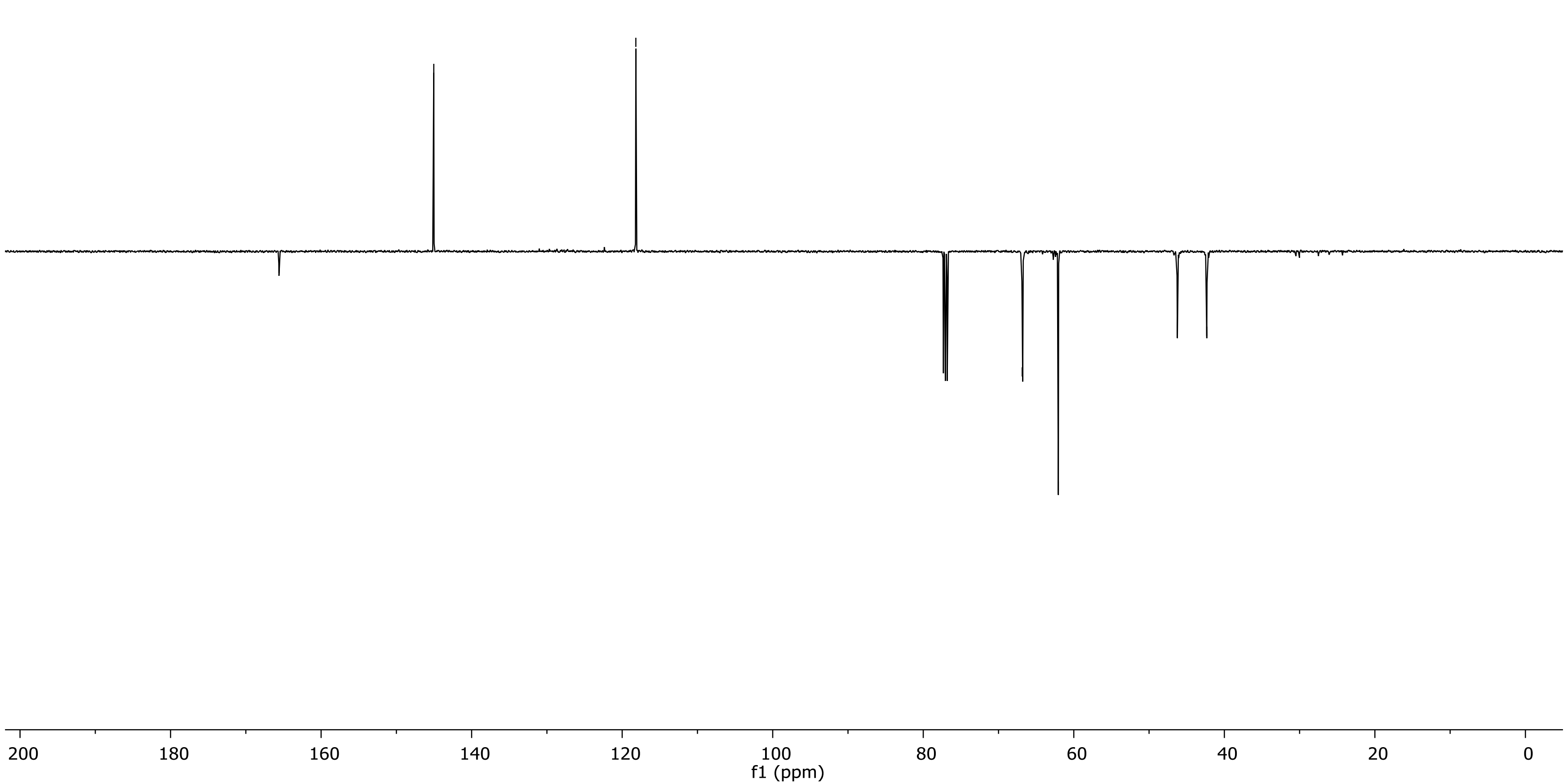

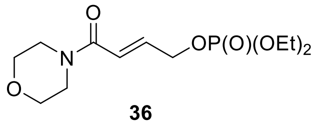

36

^1H, $CDCl_3$, 500 MHz

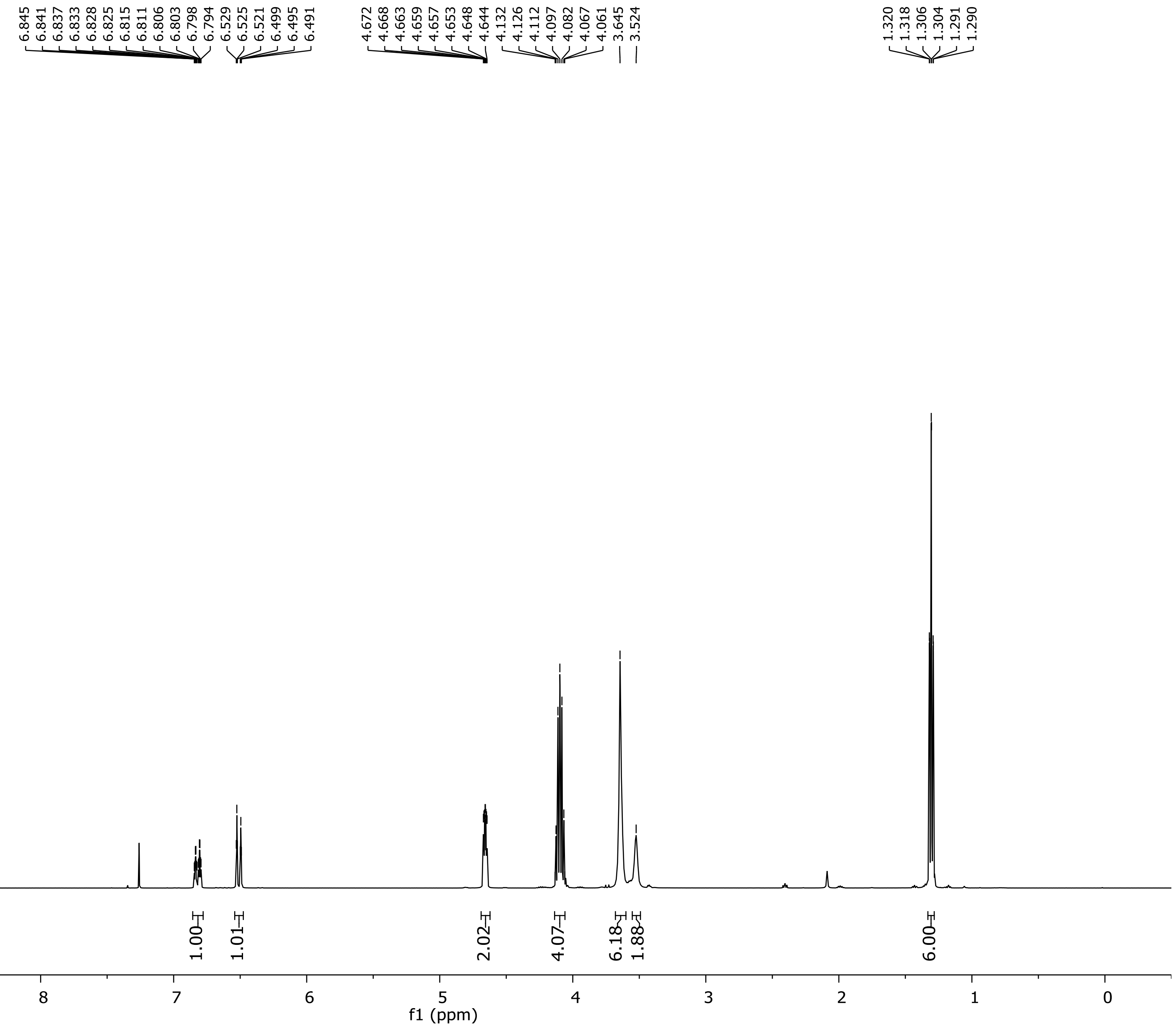

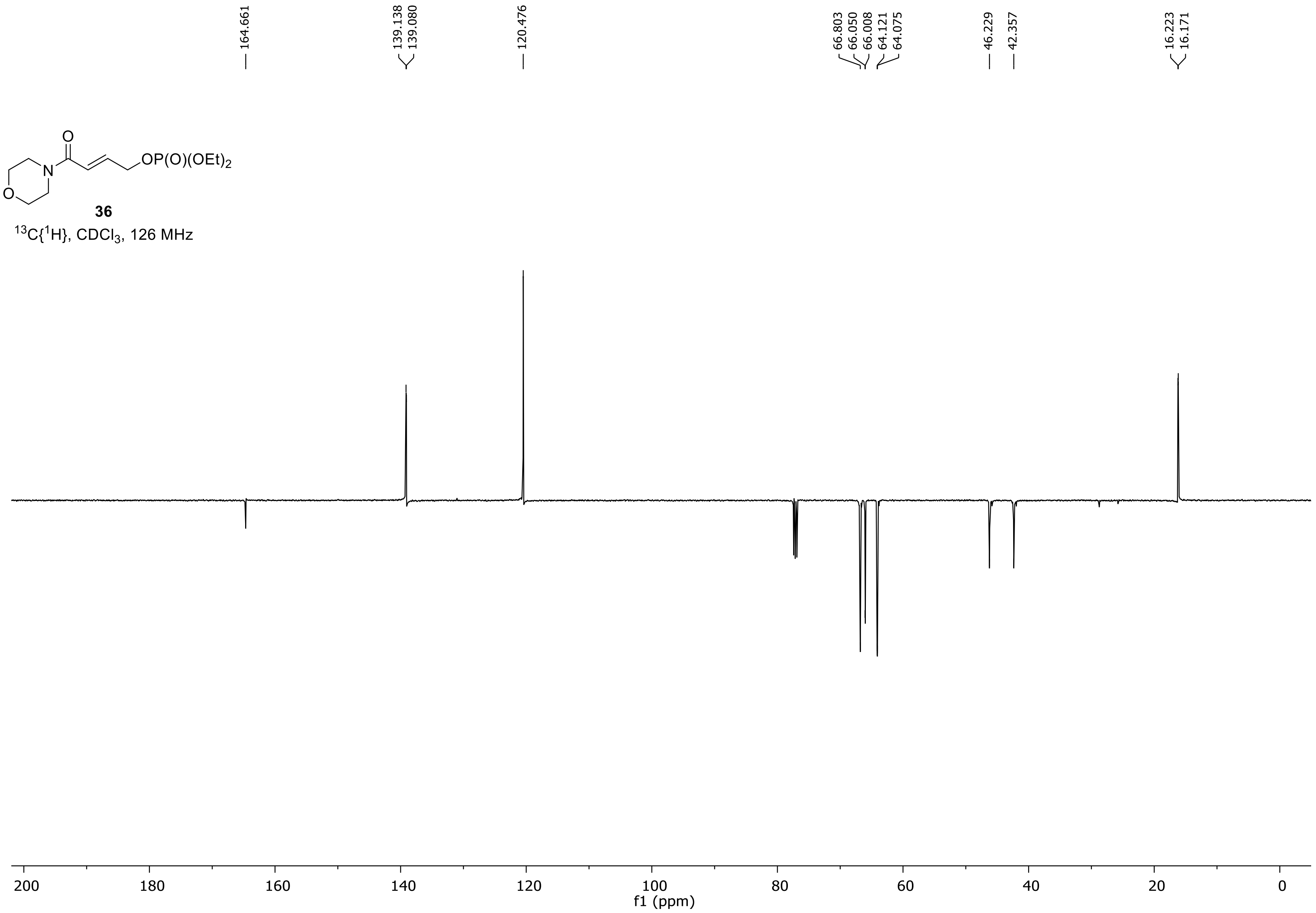
164.661
139.138
139.080
120.476
66.803
66.050
66.008
64.121
64.075
46.229
42.357
16.223
16.171
OP(O)(OEt)$_2$
36
$^{13}C\{^1H\}$, $CDCl_3$, 126 MHz
200 180 160 140 120 100 80 60 40 20 0
f1 (ppm)

-1.118

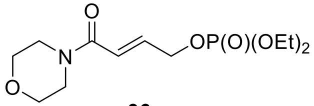

36

$^{31}P\{^{1}H\}$, $CDCl_3$, 202 MHz

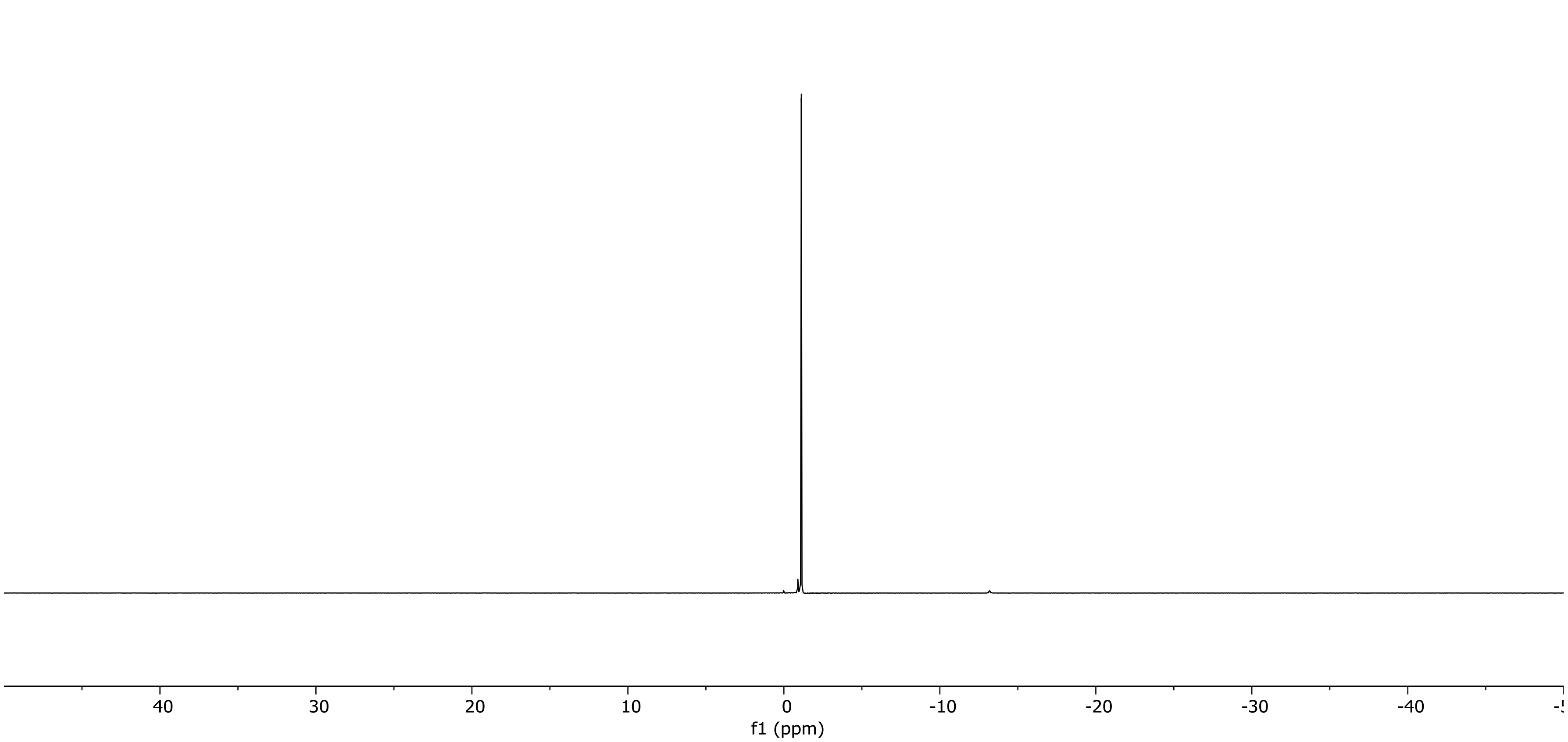

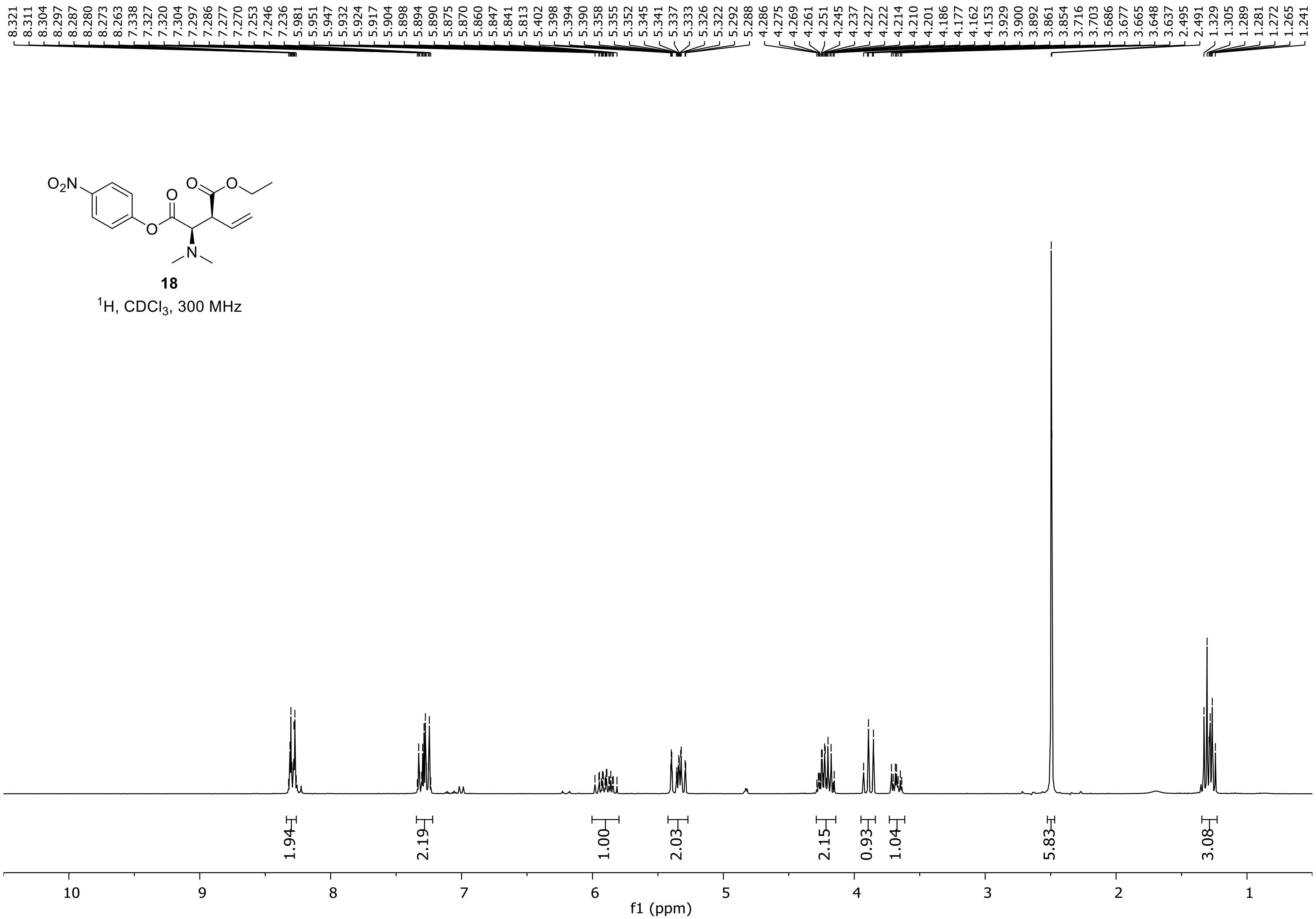

18

^{1}H, CDCl$_3$, 300 MHz

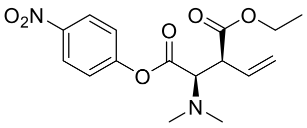

18

$^{13}C\{^{1}H\}$, $CDCl_3$, 126 MHz

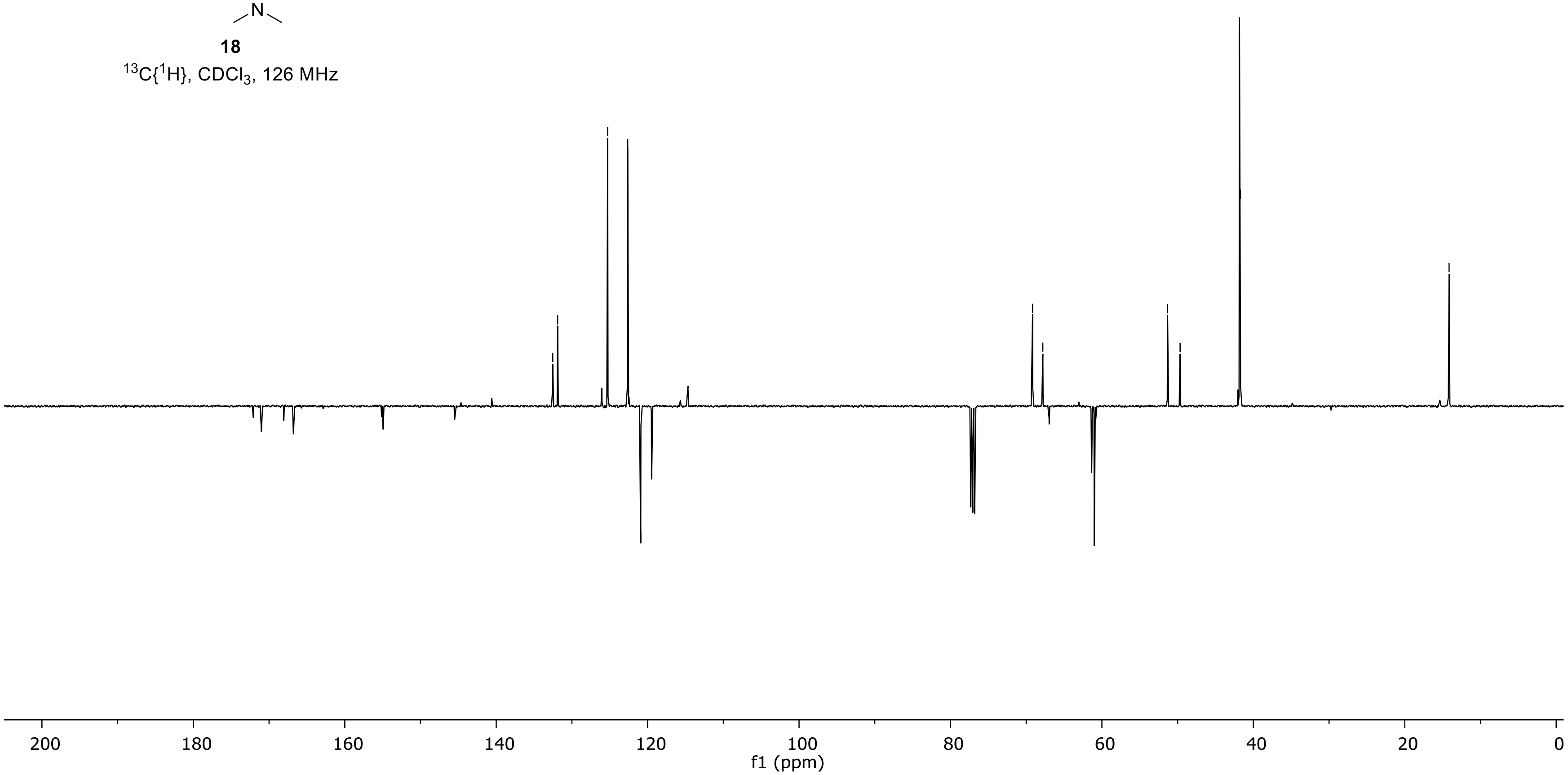

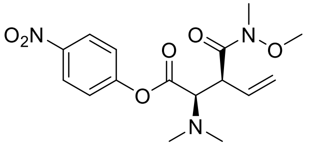

27$_{maj}$

^{1}H, $CDCl_3$, 500 MHz

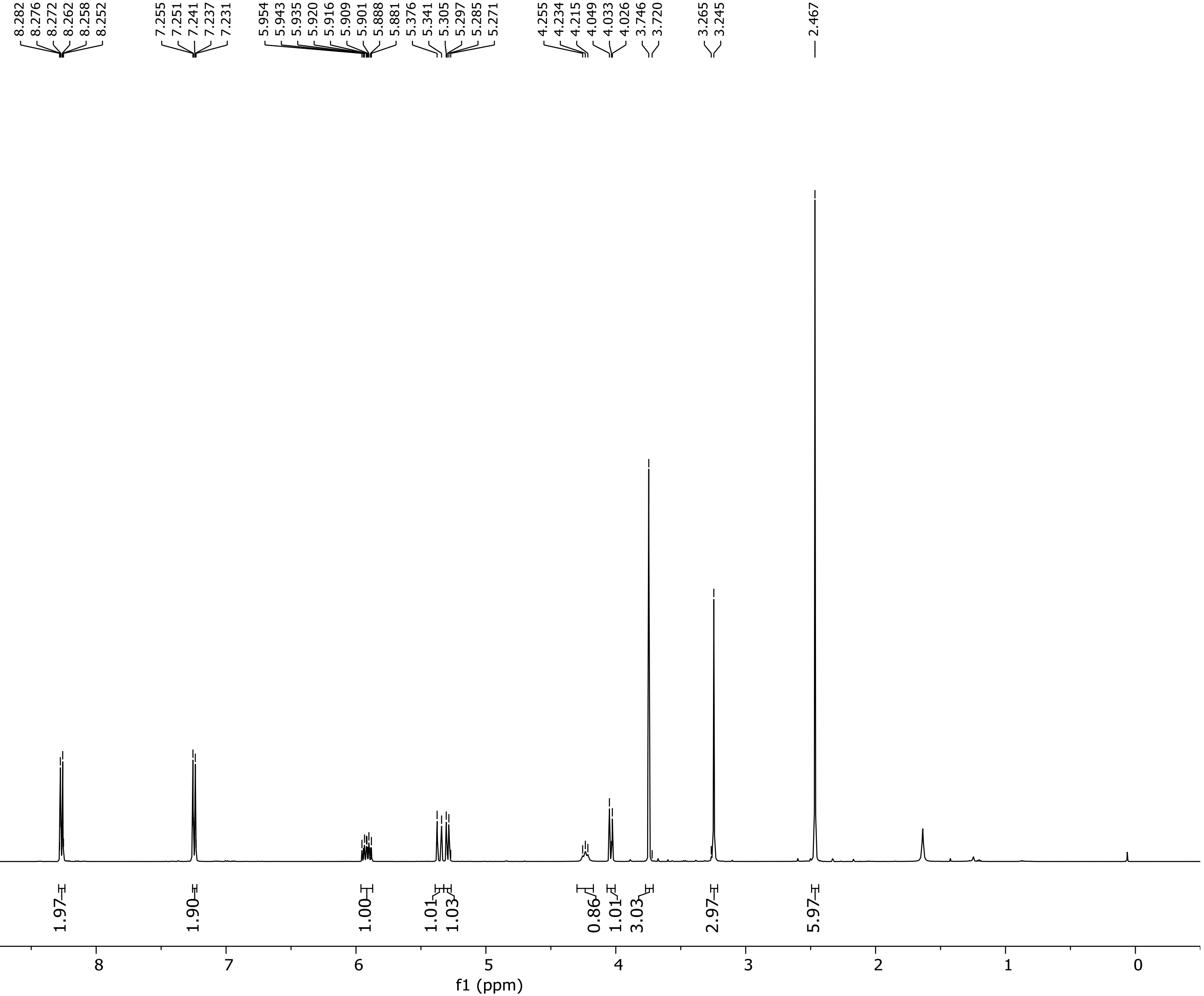

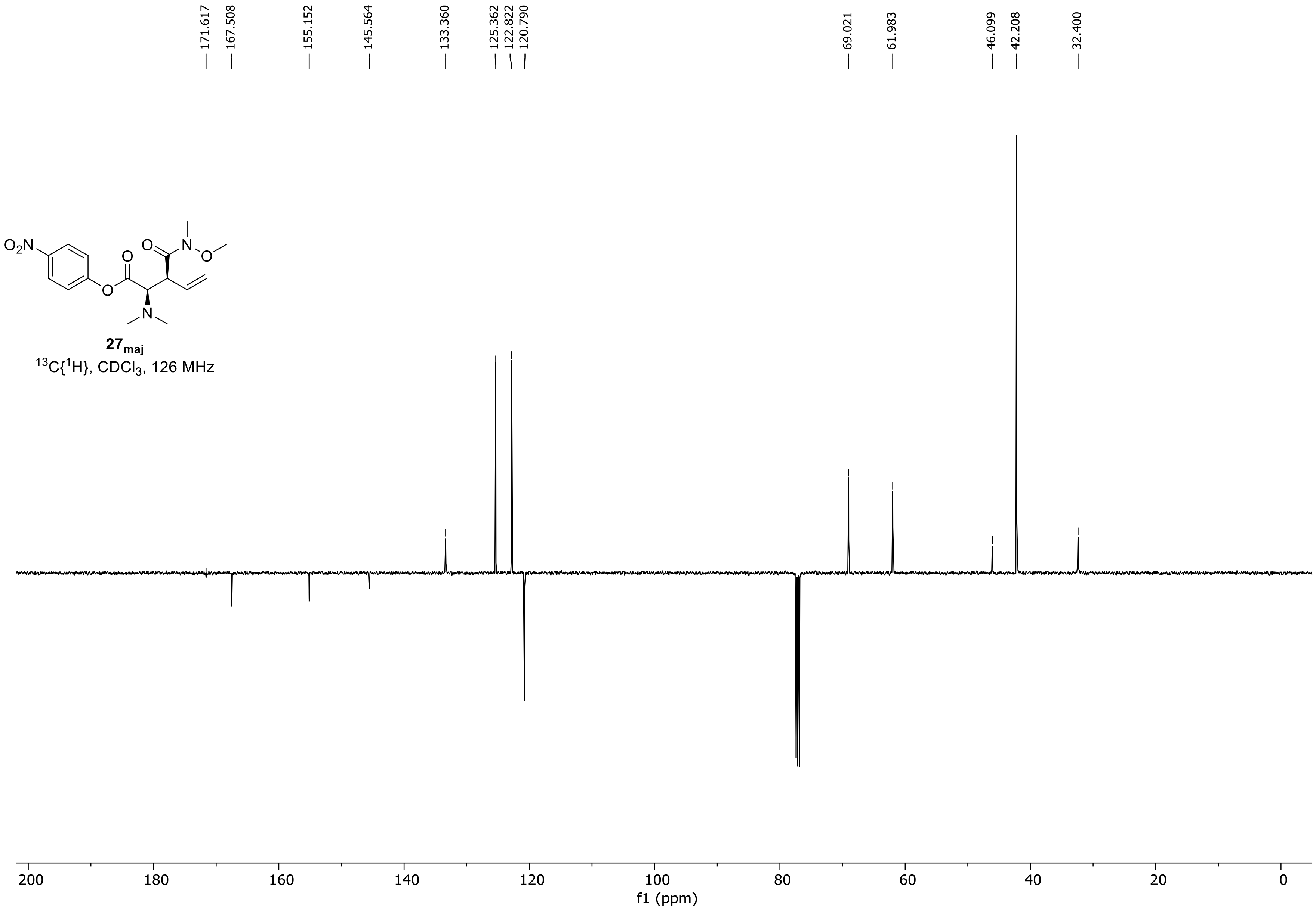
171.617
167.508
155.152
145.564
133.360
125.362
122.822
120.790
69.021
61.983
46.099
42.208
32.400
27_{maj}
$^{13}C\{^1H\}$, $CDCl_3$, 126 MHz
200
180
160
140
120
100
80
60
40
20
0
f1 (ppm)

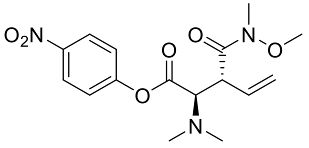

27$_{min}$

^{1}H, $CDCl_3$, 500 MHz

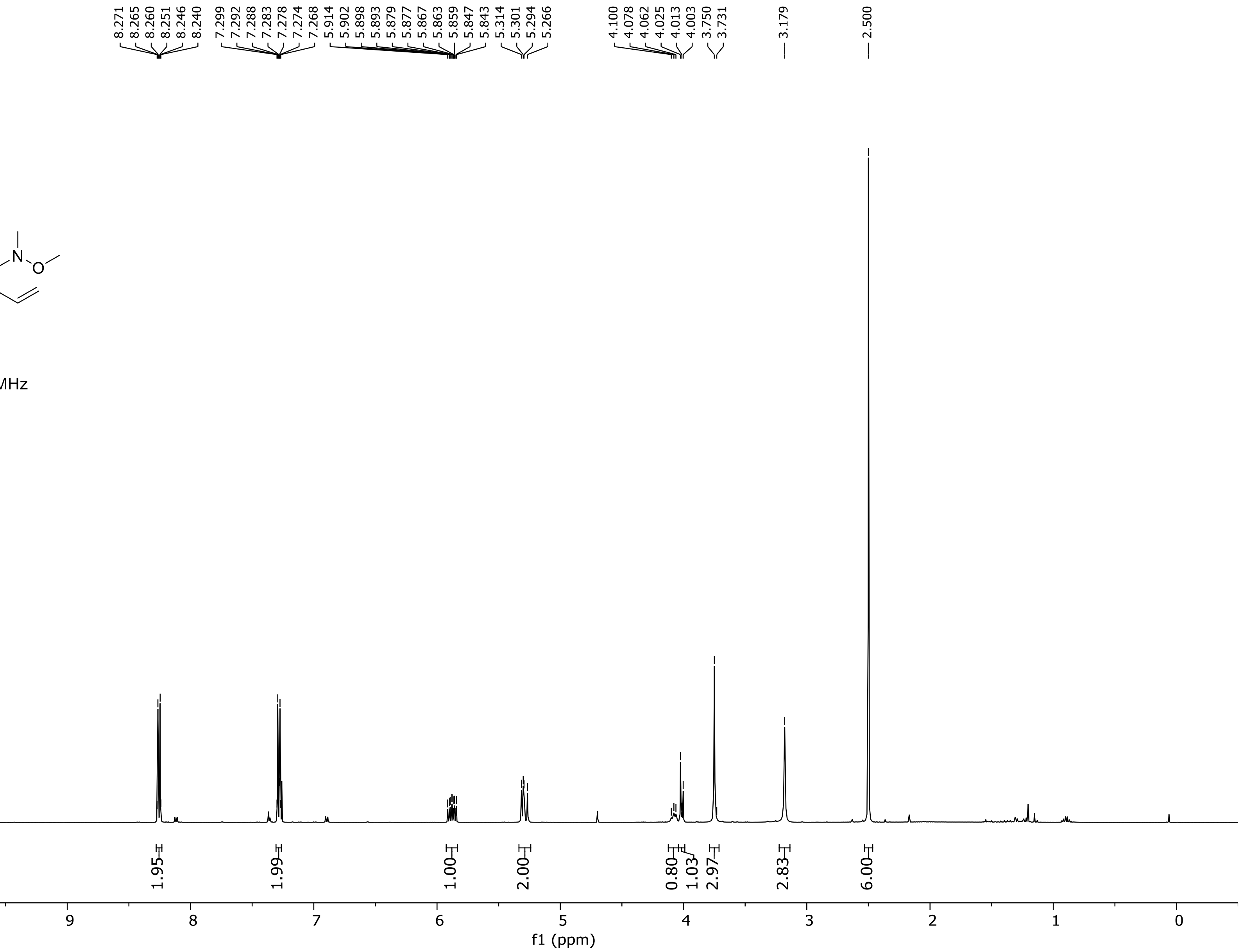

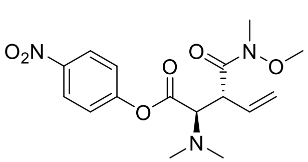

27$_{min}$

$^{13}C\{^{1}H\}$, $CDCl_3$, 126 MHz

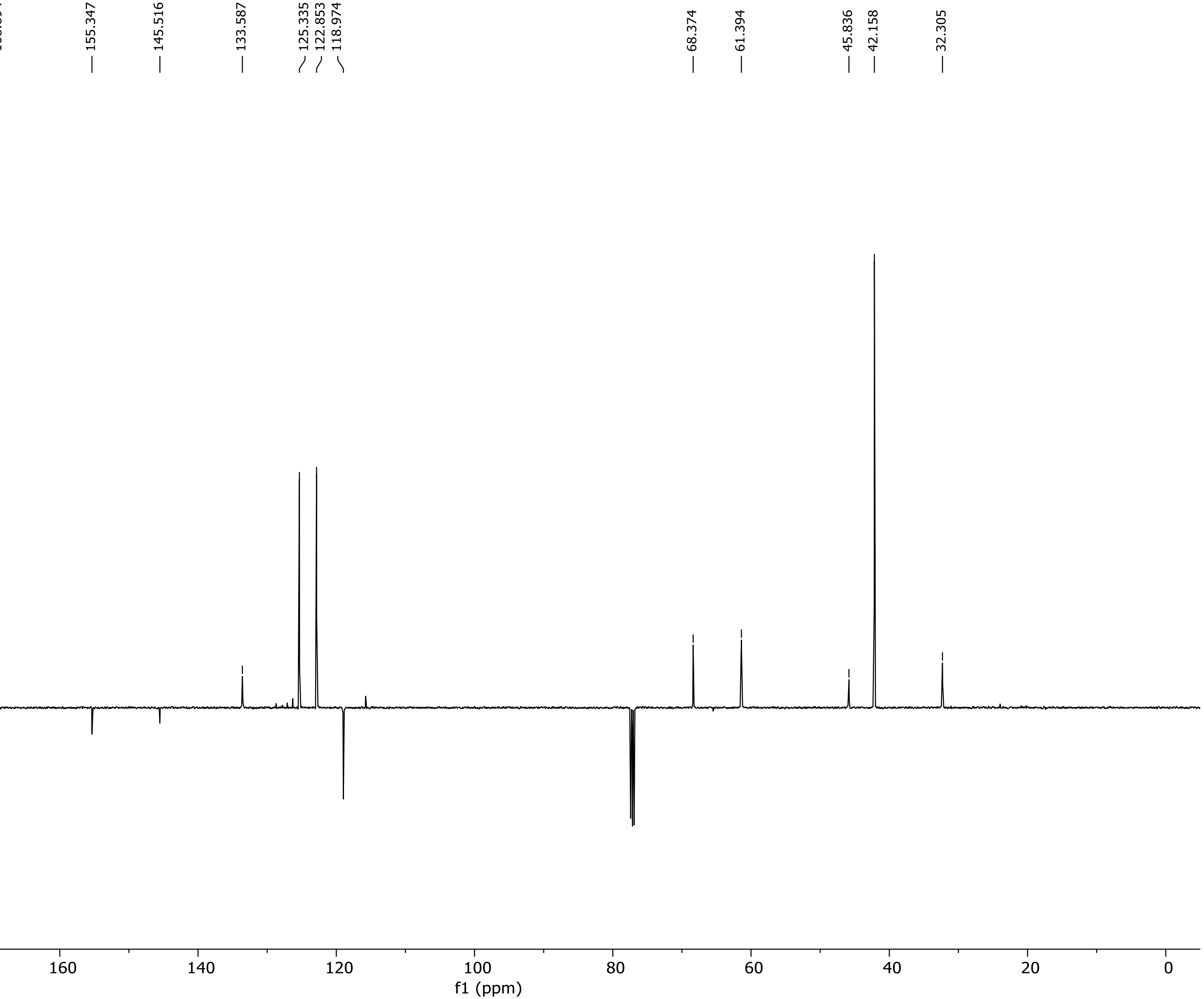

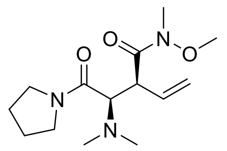

28_{maj}

^1H, $CDCl_3$, 500 MHz

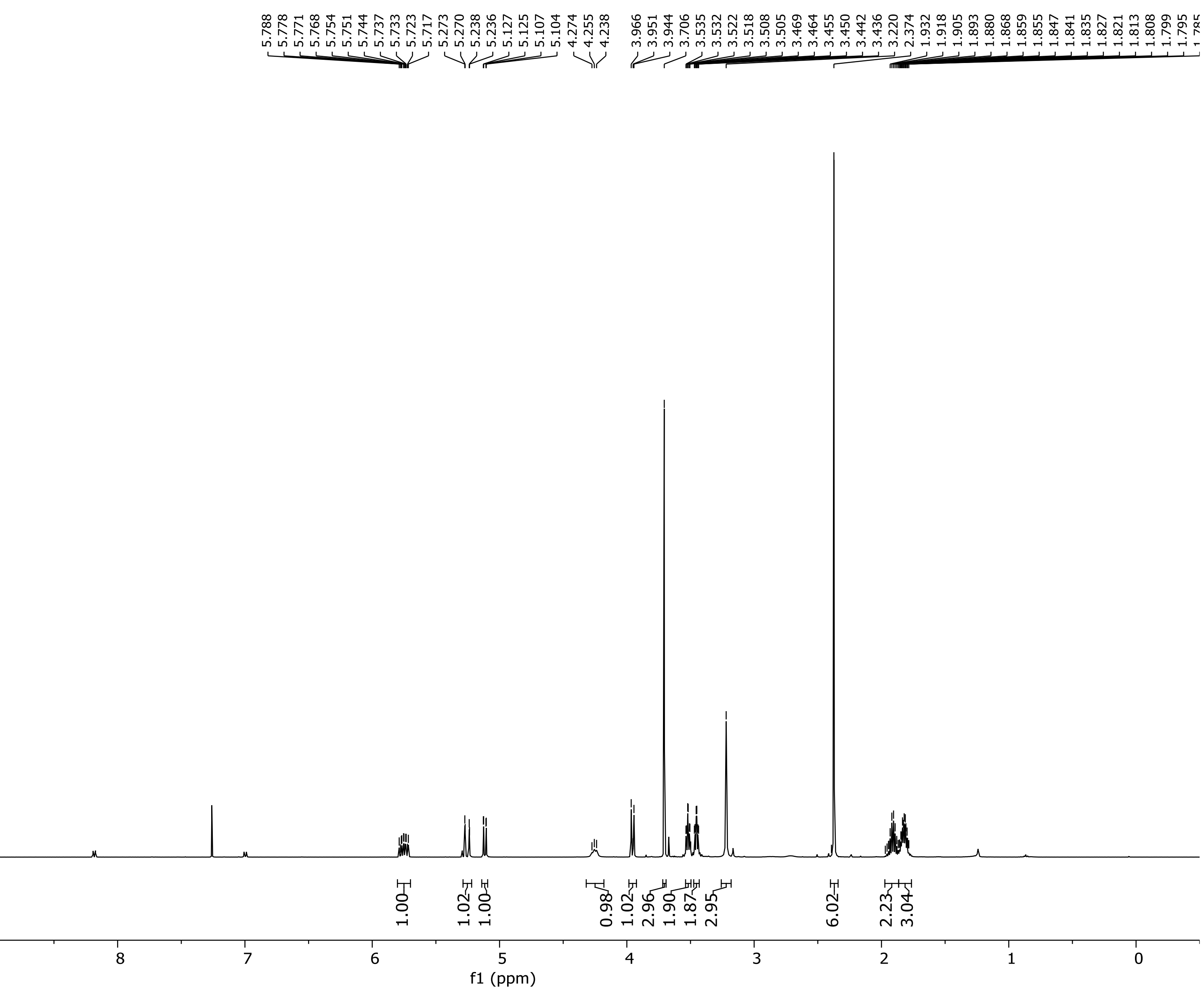

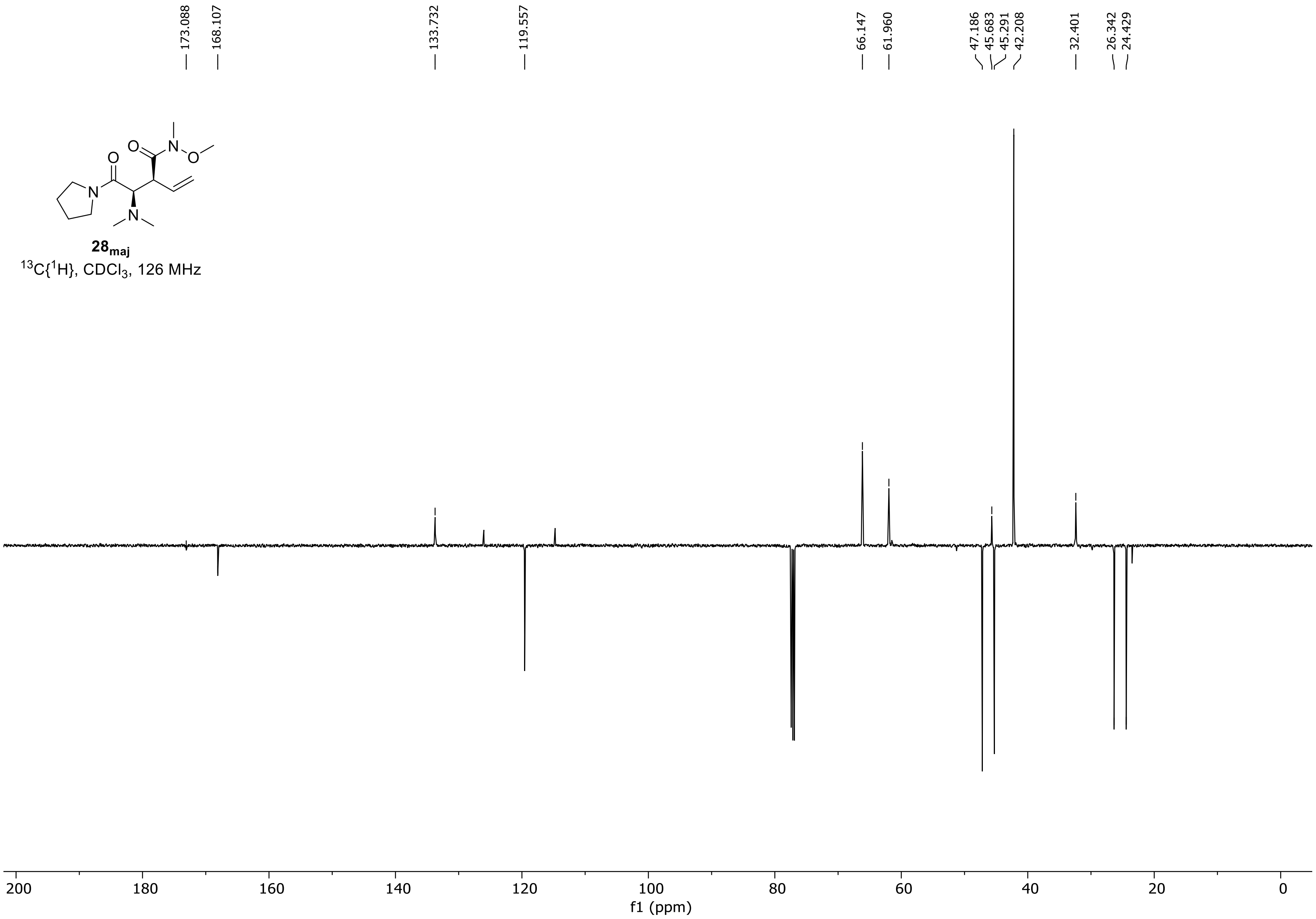

28$_{maj}$
$^{13}C\{^1H\}$, $CDCl_3$, 126 MHz

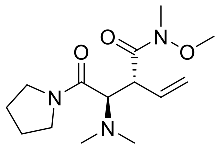

28$_{min}$

^1H, $CDCl_3$, 400 MHz

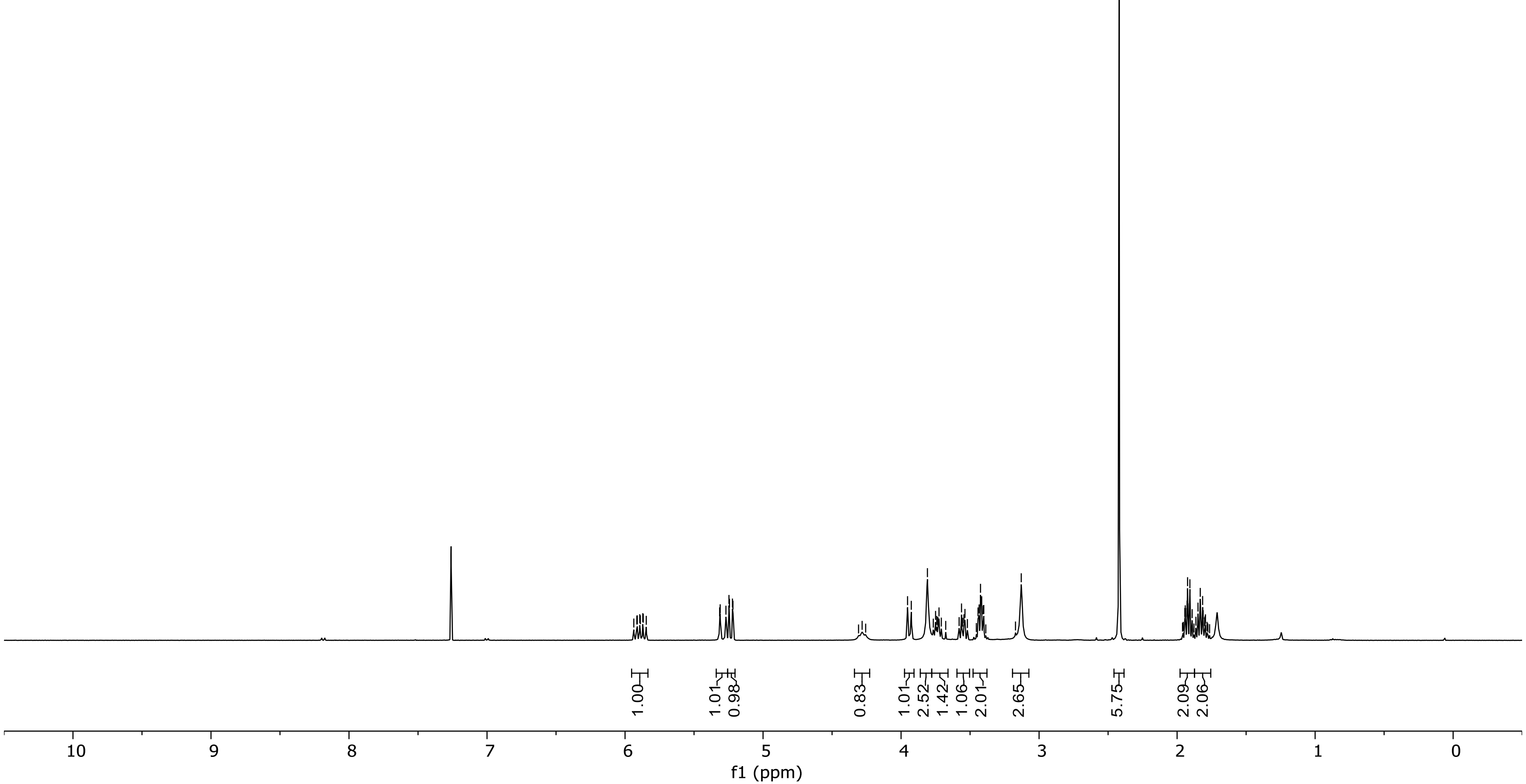

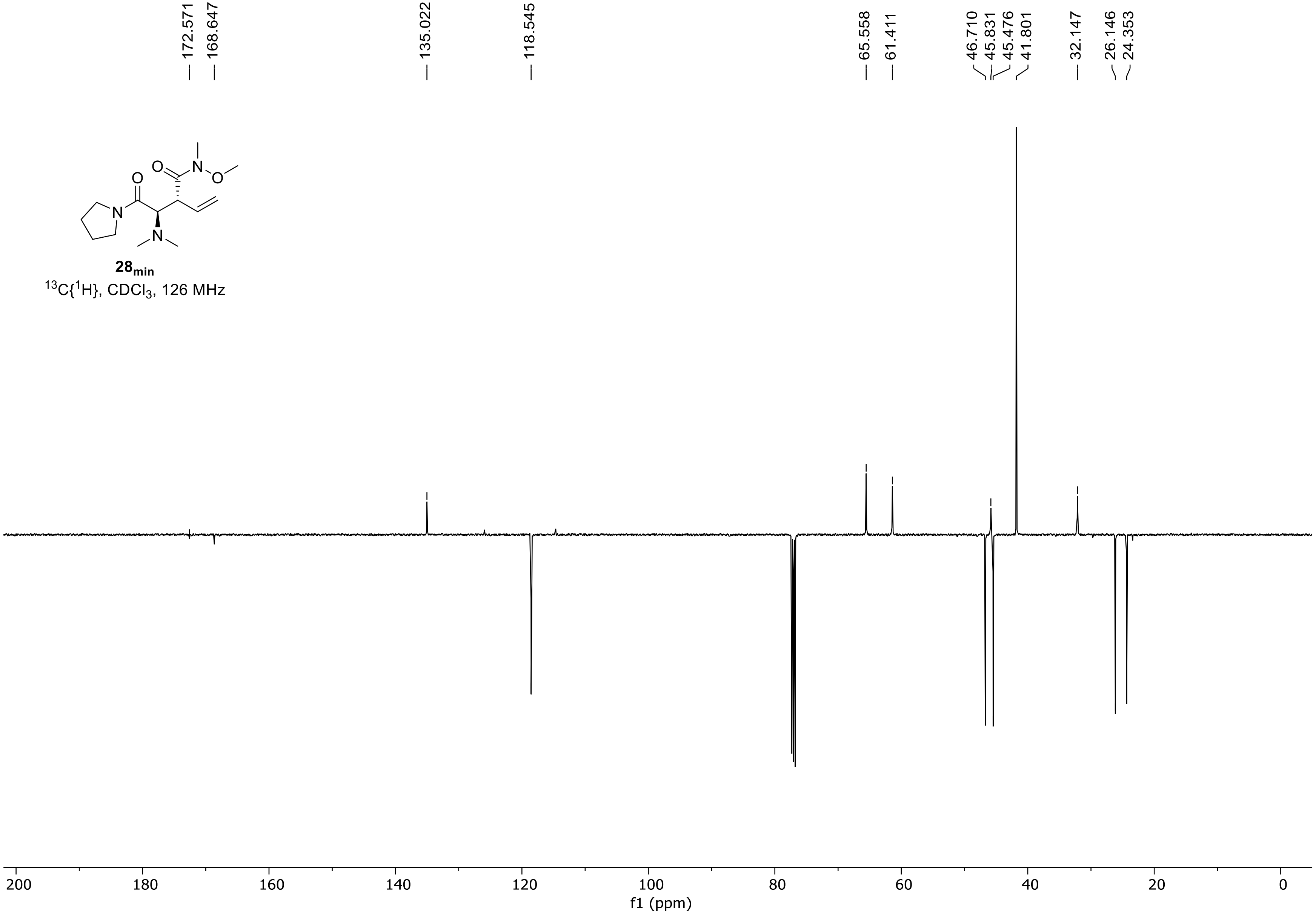

28$_{min}$

$^{13}C\{^1H\}$, $CDCl_3$, 126 MHz

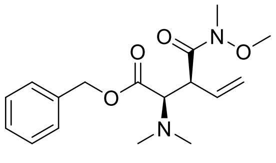

$\mathbf{29_{maj}}$

^{1}H, $CDCl_3$, 500 MHz

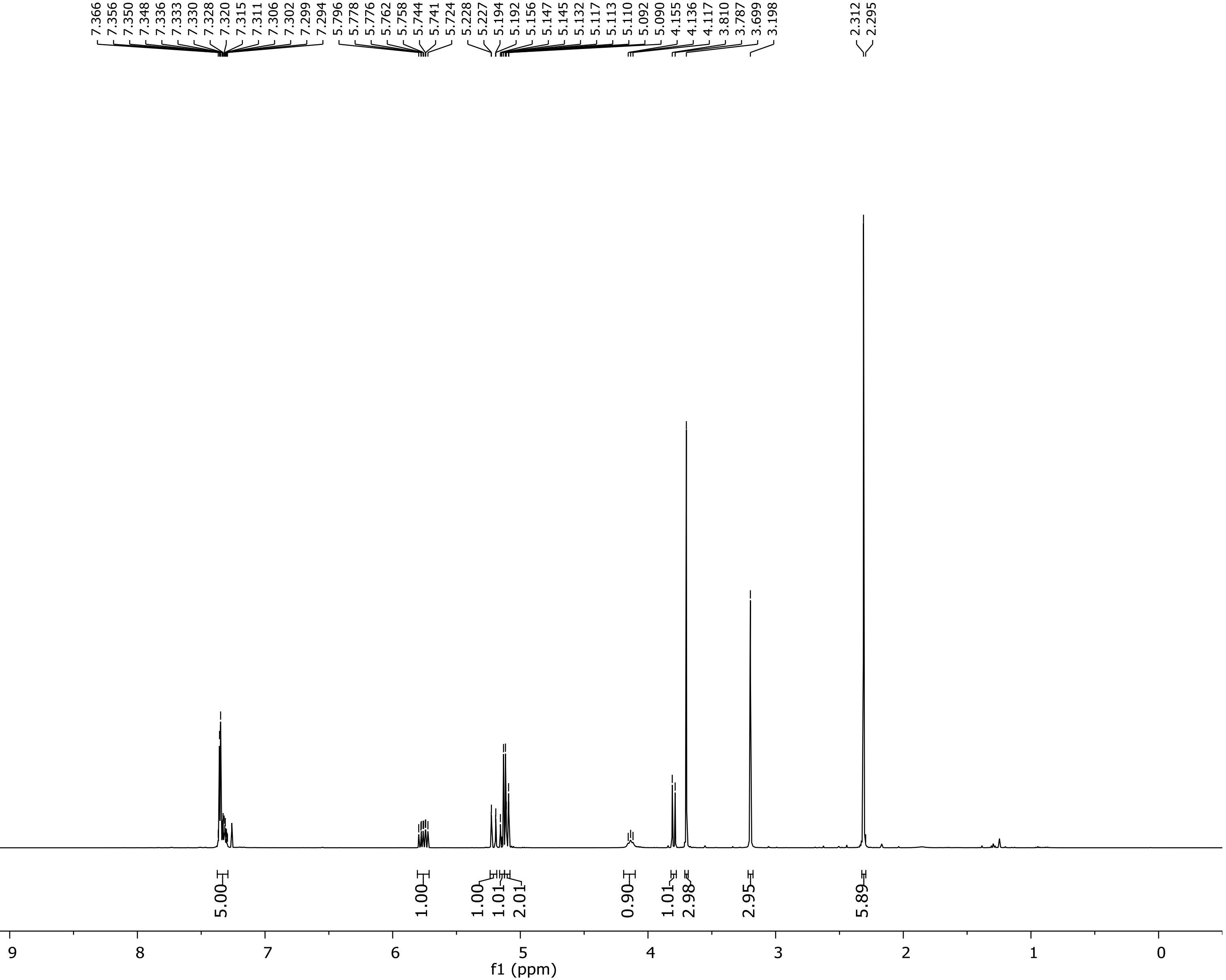

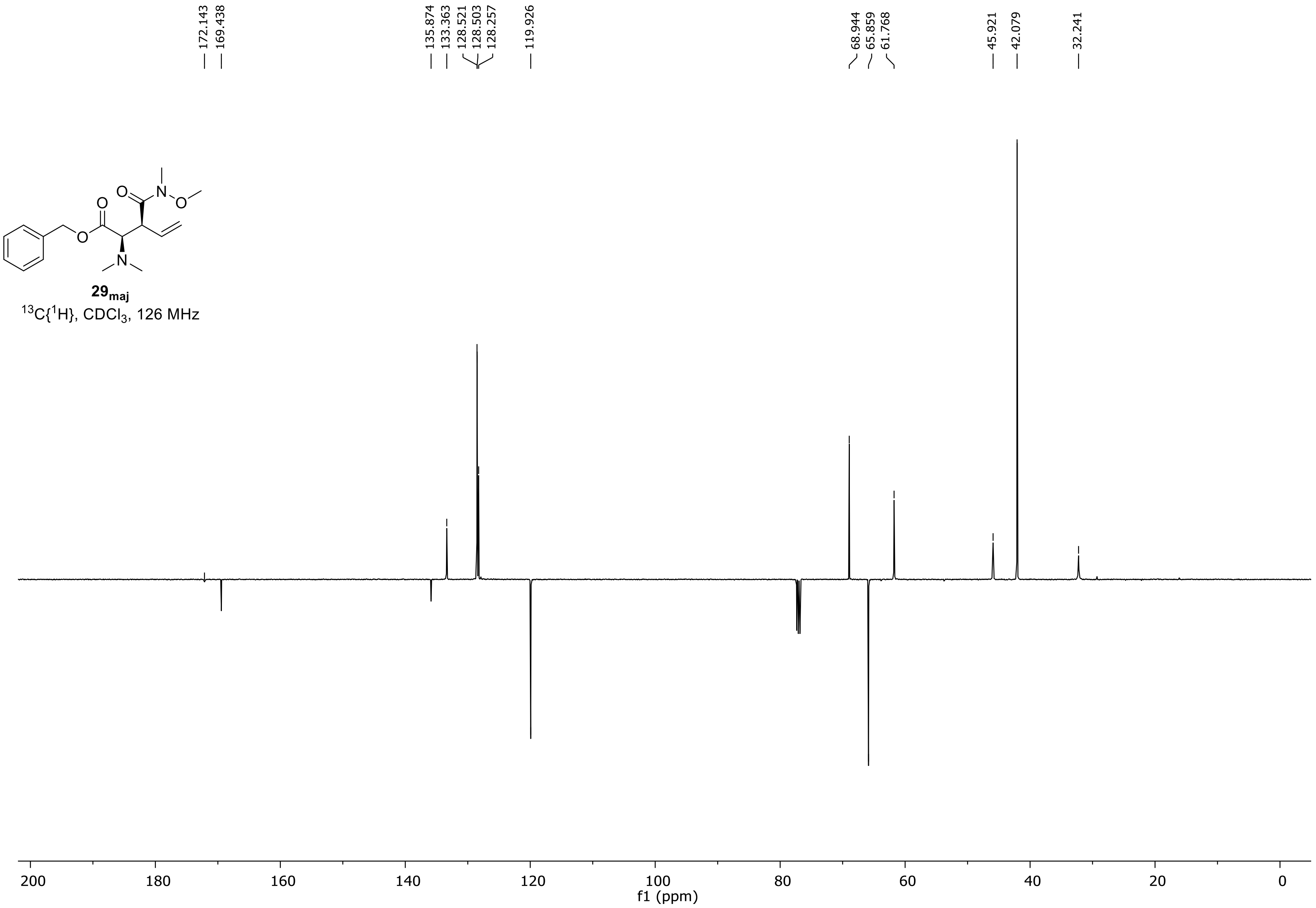
172.143
169.438
135.874
133.363
128.521
128.503
128.257
119.926
68.944
65.859
61.768
45.921
42.079
32.241
O
N
O
O
O
N
29maj
13C{1H}, CDCl3, 126 MHz
200
180
160
140
120
100
80
60
40
20
0
f1 (ppm)

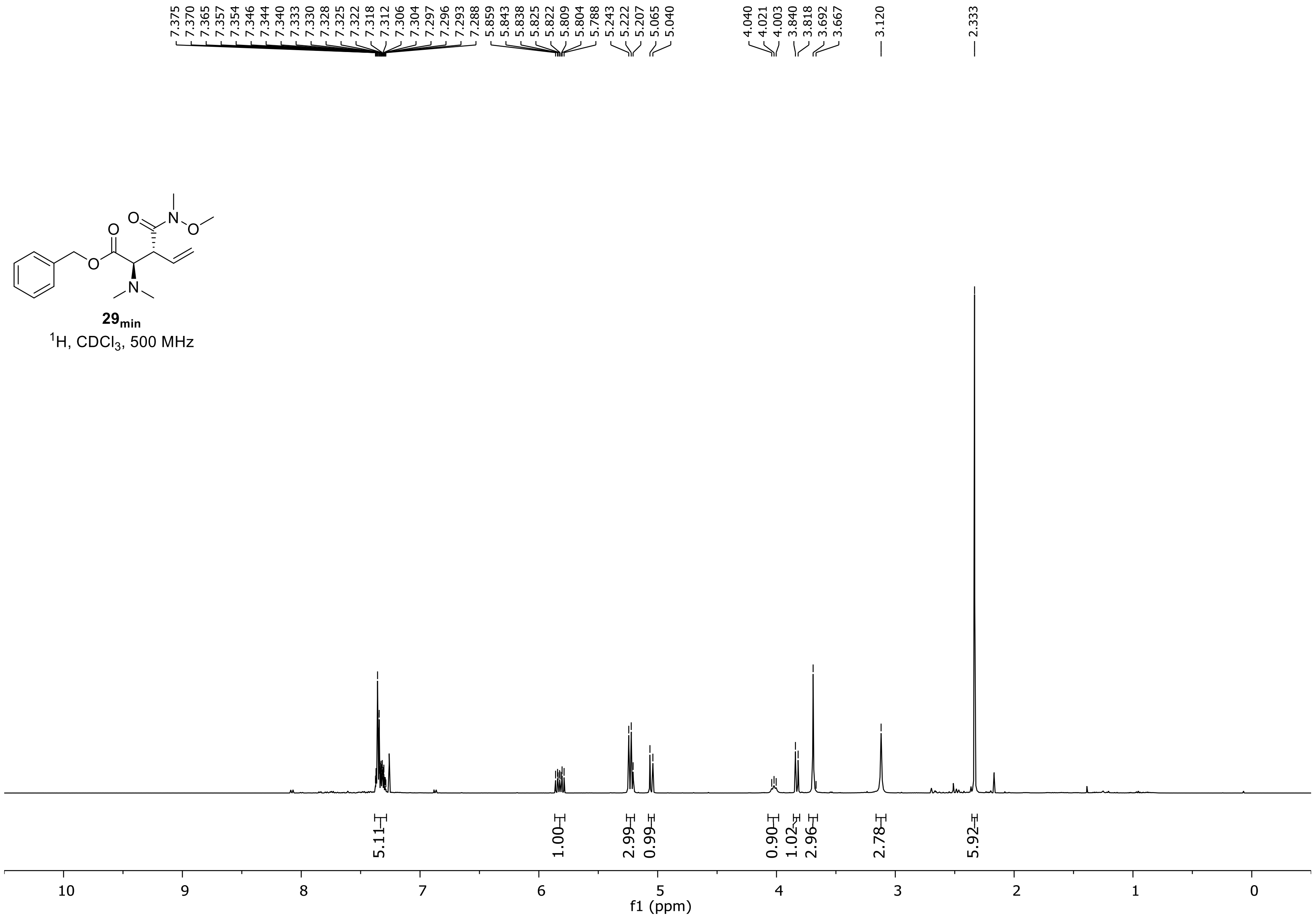
7.375 7.370 7.365 7.357 7.354 7.346 7.344 7.340 7.333 7.330 7.328 7.325 7.322 7.318 7.312 7.306 7.304 7.297 7.296 7.293 7.288 5.859 5.843 5.838 5.825 5.822 5.809 5.804 5.788 5.243 5.222 5.207 5.065 5.040 4.040 4.021 4.003 3.840 3.818 3.692 3.667 3.120 2.333
O
N
O
O
O
N
29min
1H, CDCl3, 500 MHz
5.11 1.00 2.99 0.99 0.90 1.02 2.96 2.78 5.92
10 9 8 7 6 5 4 3 2 1 0
f1 (ppm)

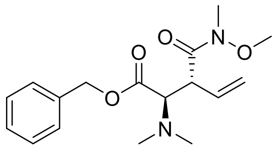

29$_{min}$

$^{13}C\{^1H\}$, $CDCl_3$, 126 MHz

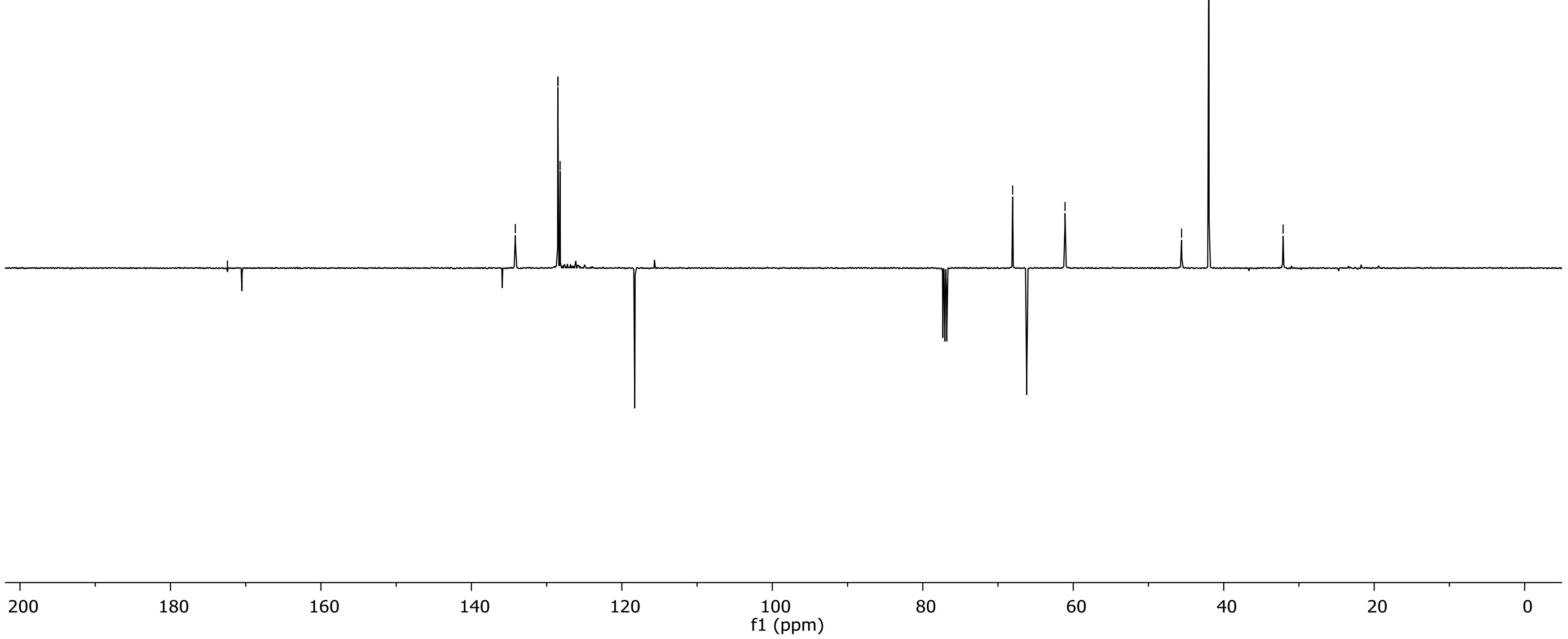

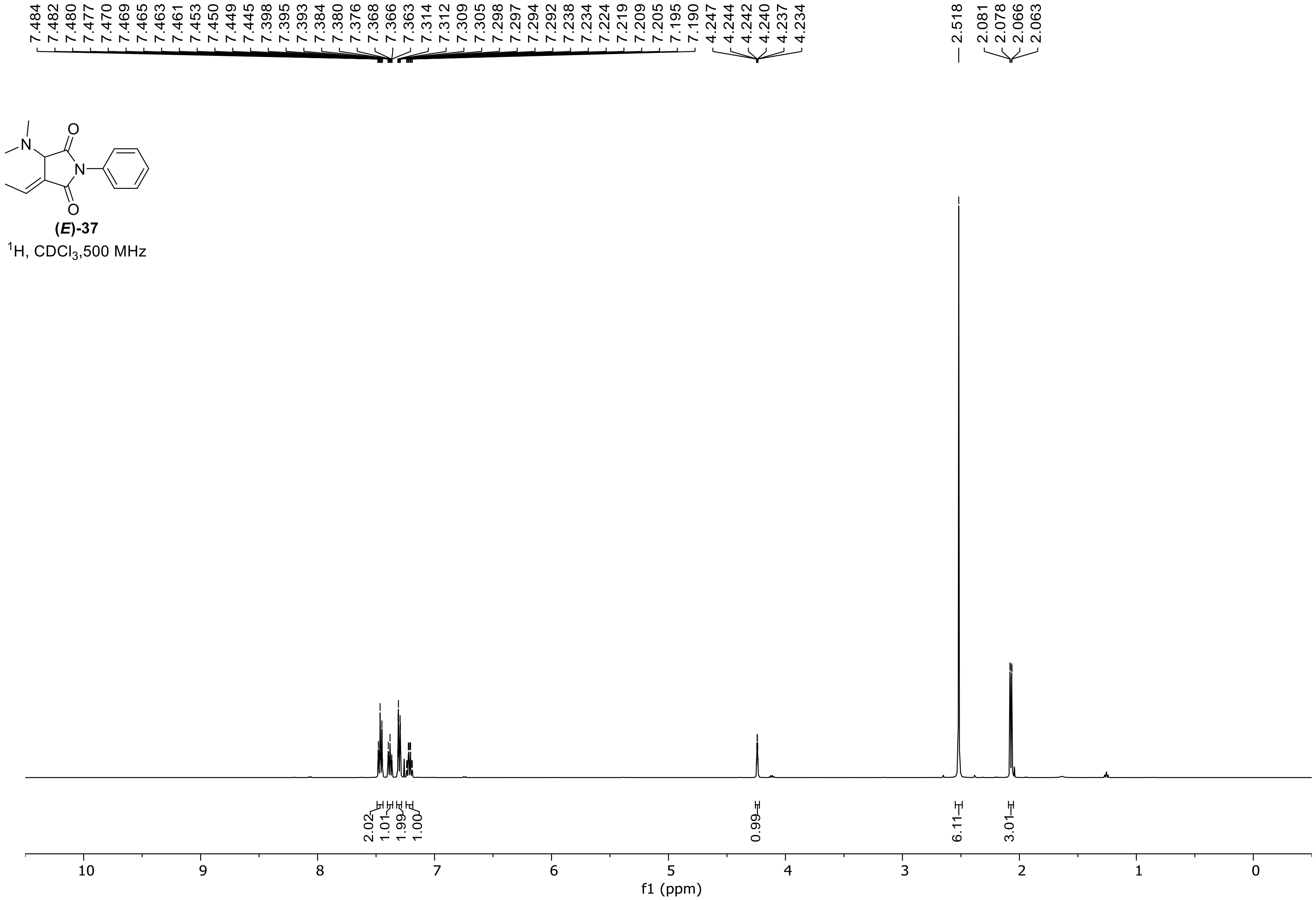
(E)-37
1H, CDCl3,500 MHz
7.484
7.482
7.480
7.477
7.470
7.469
7.465
7.463
7.461
7.453
7.450
7.449
7.445
7.398
7.395
7.393
7.384
7.380
7.376
7.368
7.366
7.363
7.314
7.312
7.309
7.305
7.298
7.297
7.294
7.292
7.238
7.234
7.224
7.219
7.209
7.205
7.195
7.190
4.247
4.244
4.242
4.240
4.237
4.234
2.518
2.081
2.078
2.066
2.063
2.02
1.01
1.99
1.00
0.99
6.11
3.01
10
9
8
7
6
5
4
3
2
1
0
f1 (ppm)

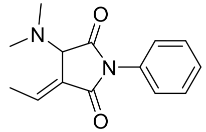

(*E*)-37

$^{13}C\{^1H\}$, $CDCl_3$, 126 MHz

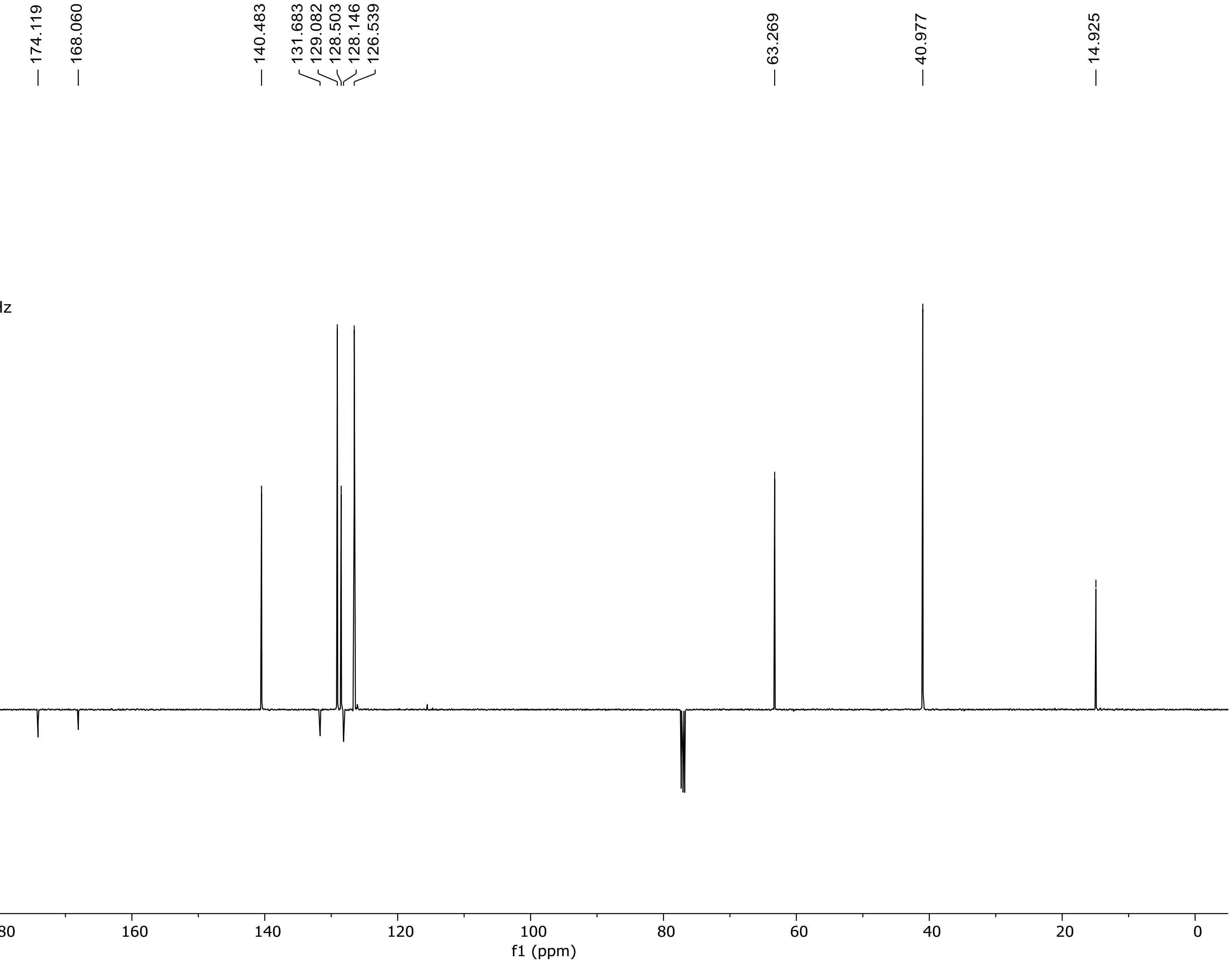

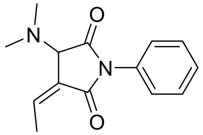

(*Z*)-37

^{1}H, $CDCl_3$, 500 MHz

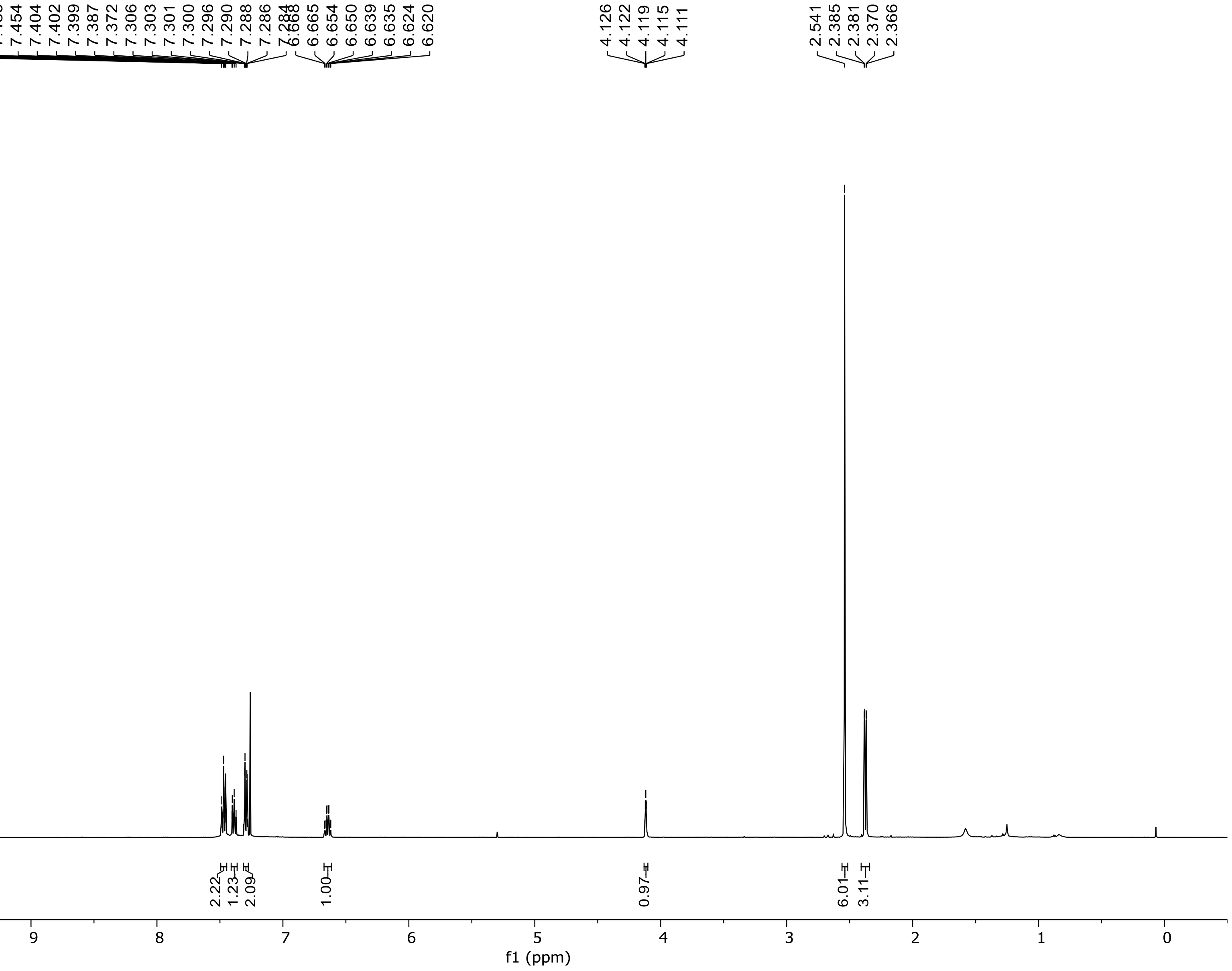

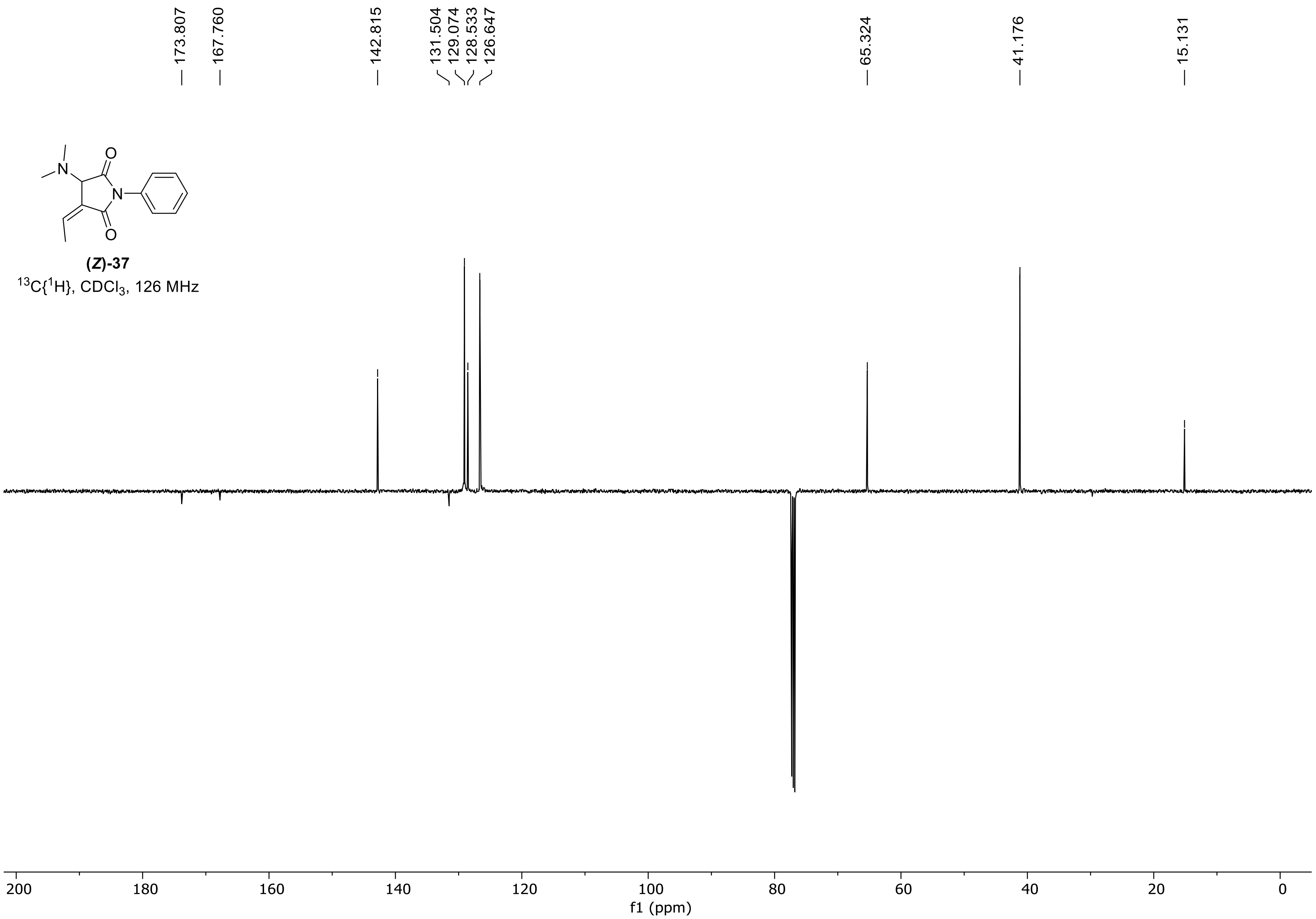

173.807
167.760
142.815
131.504
129.074
128.533
126.647
65.324
41.176
15.131
(Z)-37
$^{13}C\{^1H\}$, $CDCl_3$, 126 MHz
200 180 160 140 120 100 80 60 40 20 0
f1 (ppm)

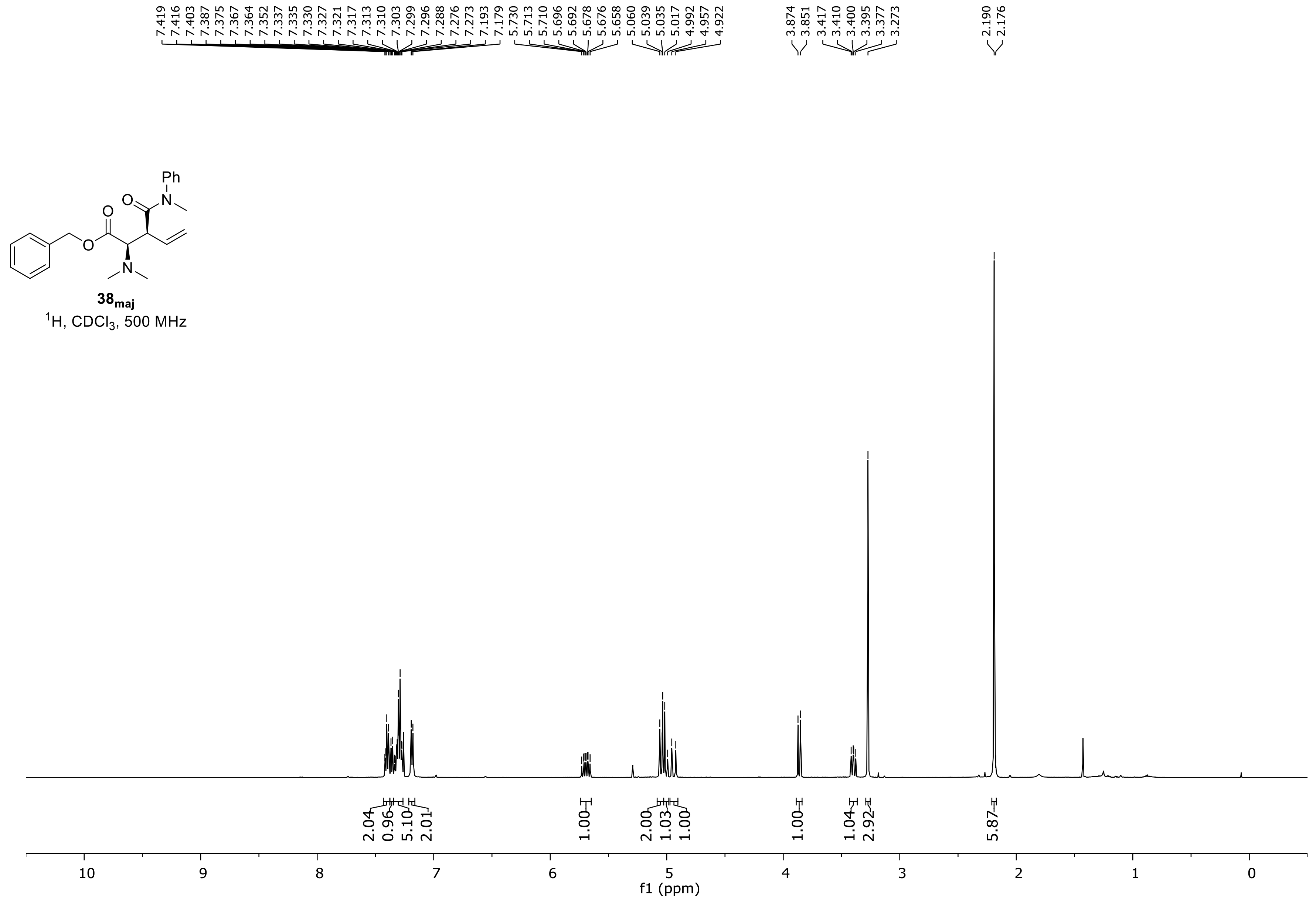
38maj
1H, CDCl3, 500 MHz
f1 (ppm)

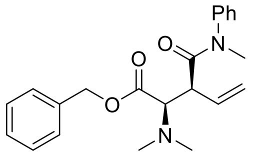

38$_{maj}$

$^{13}C\{^1H\}$, $CDCl_3$, 126 MHz

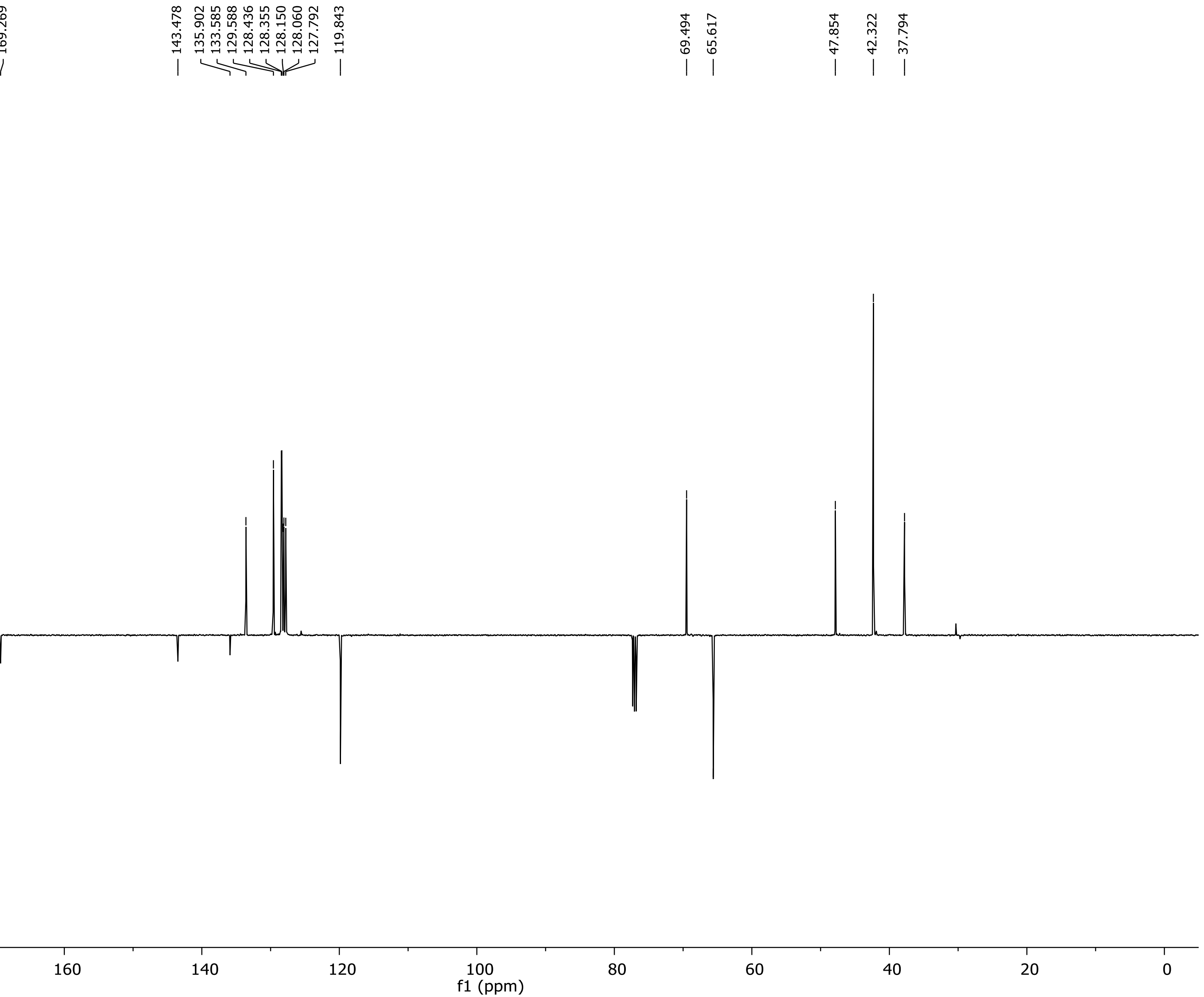

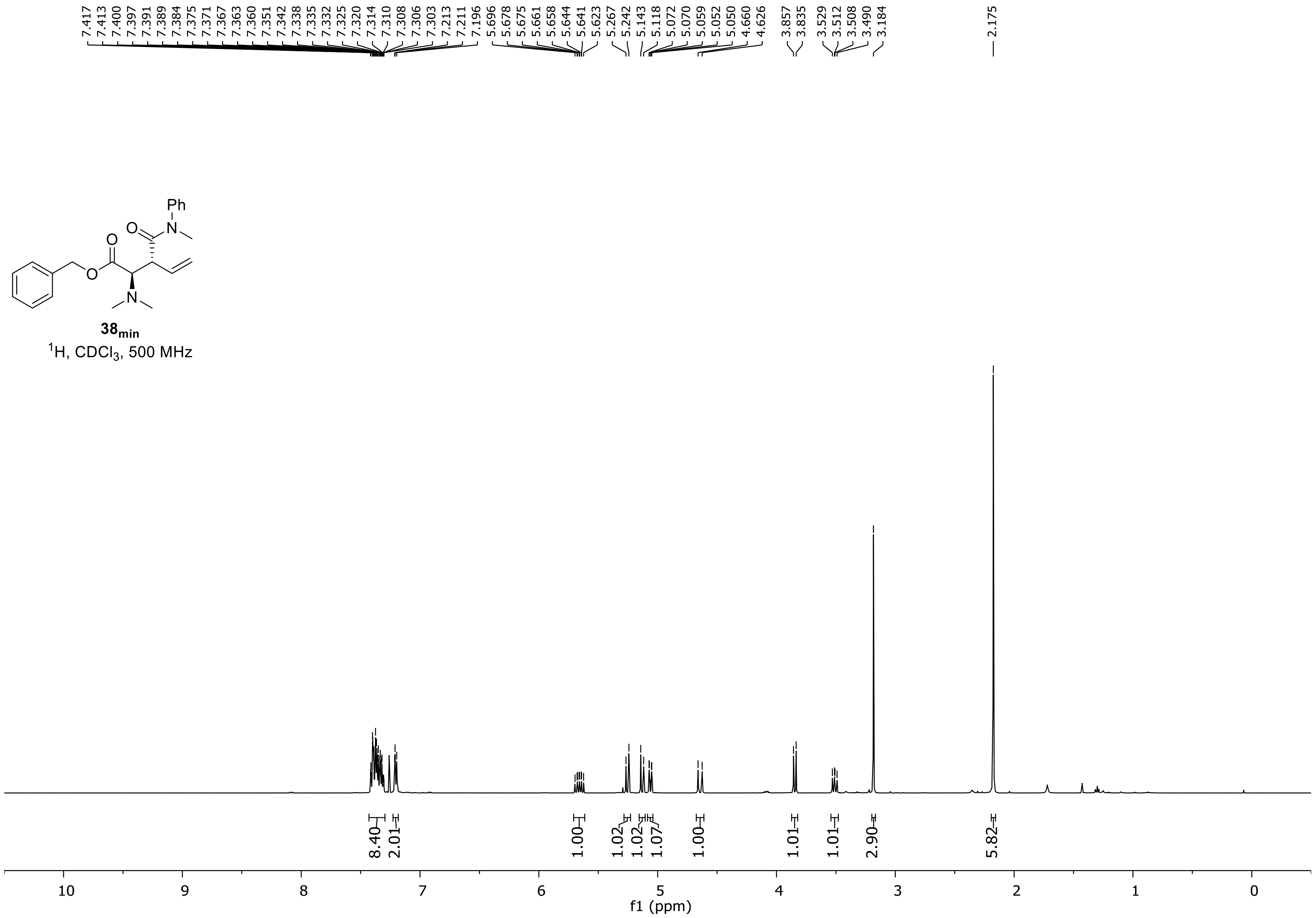

38$_{min}$

^{1}H, $CDCl_3$, 500 MHz

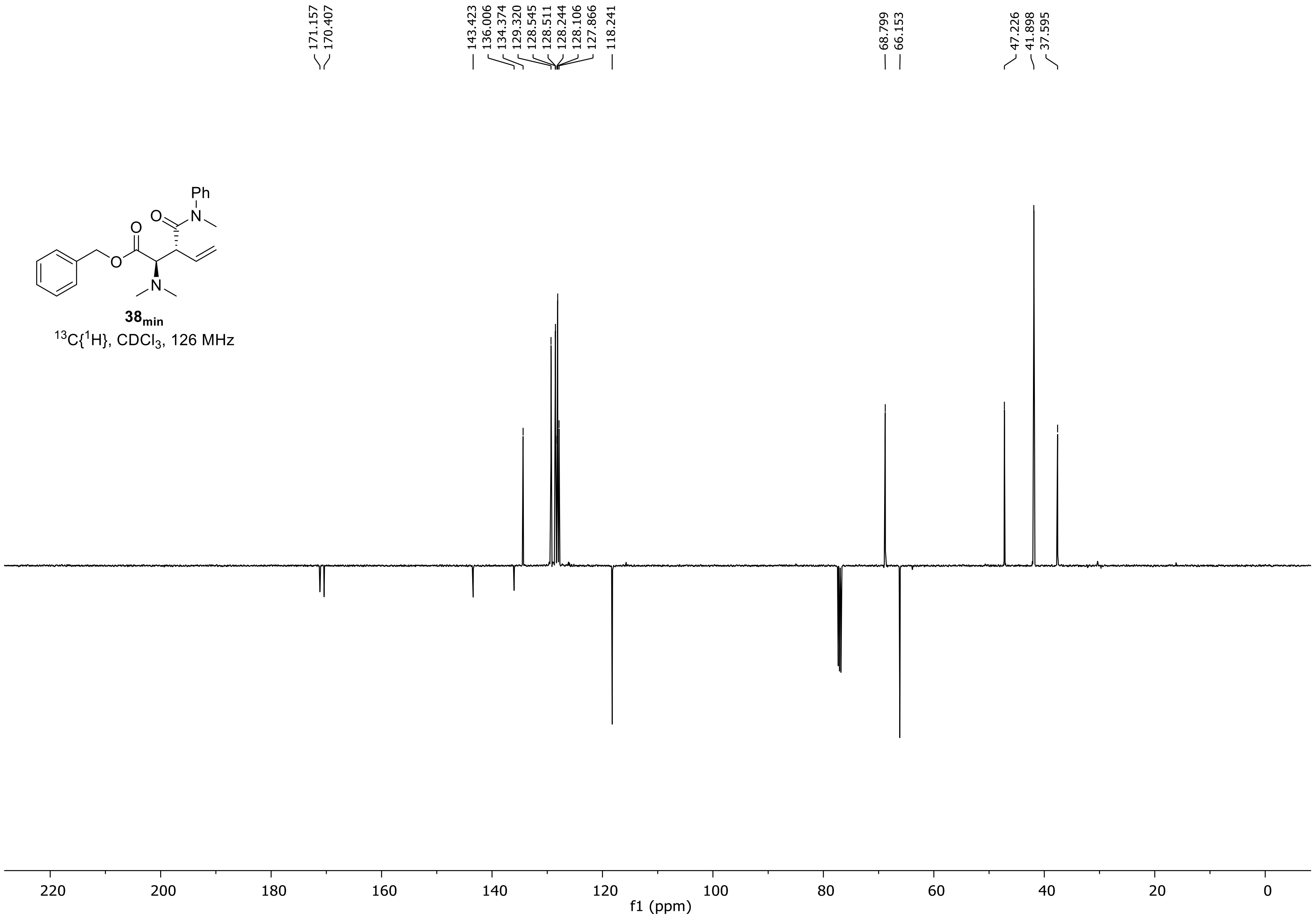
171.157
170.407
143.423
136.006
134.374
129.320
128.545
128.511
128.244
128.106
127.866
118.241
68.799
66.153
47.226
41.898
37.595
Ph
N
O
O
O
N
38min
13C{1H}, CDCl3, 126 MHz
220
200
180
160
140
120
100
80
60
40
20
0
f1 (ppm)

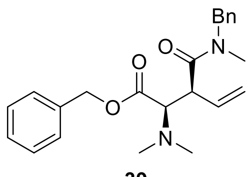

39_{maj}
^1H, $CDCl_3$, 500 MHz

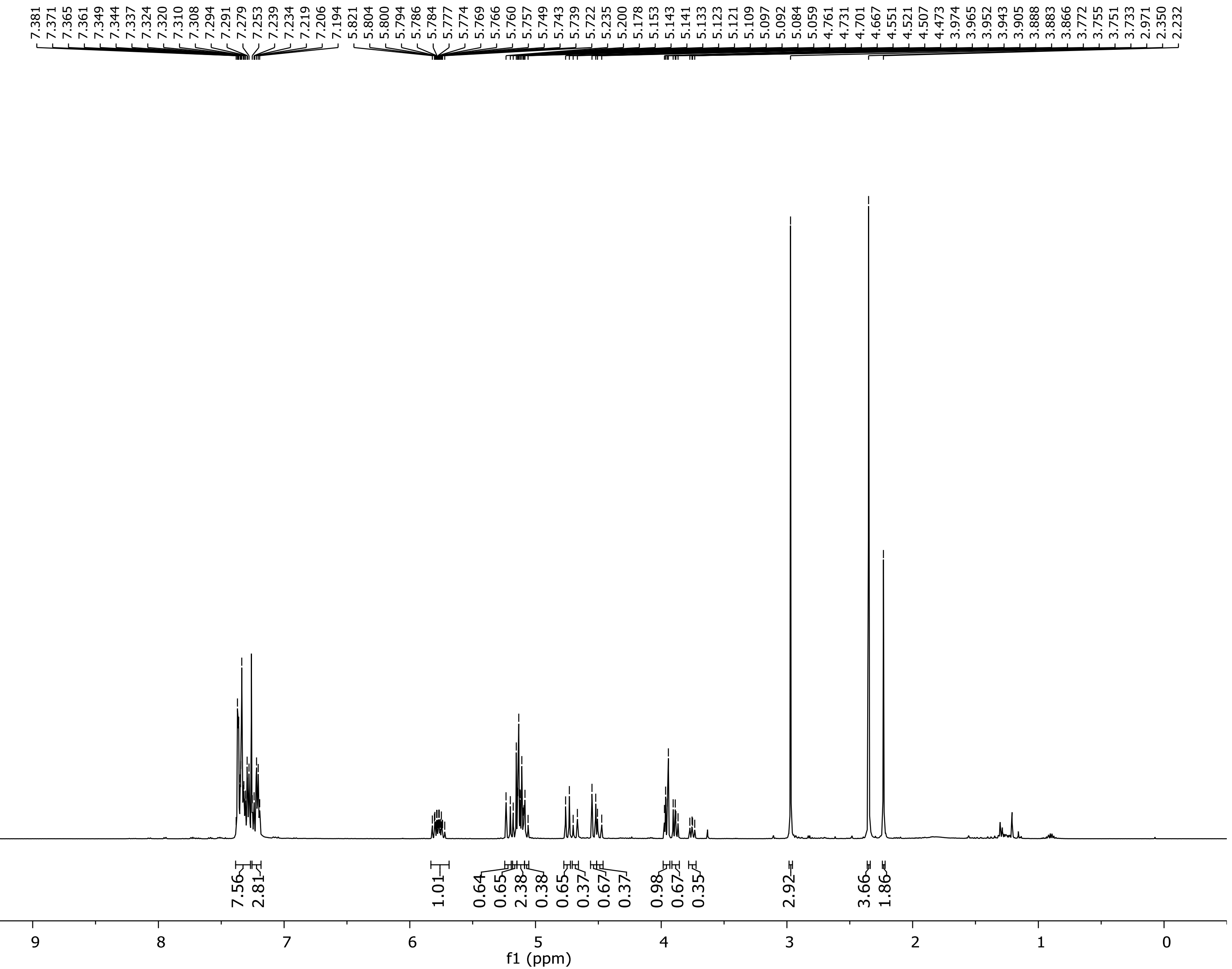

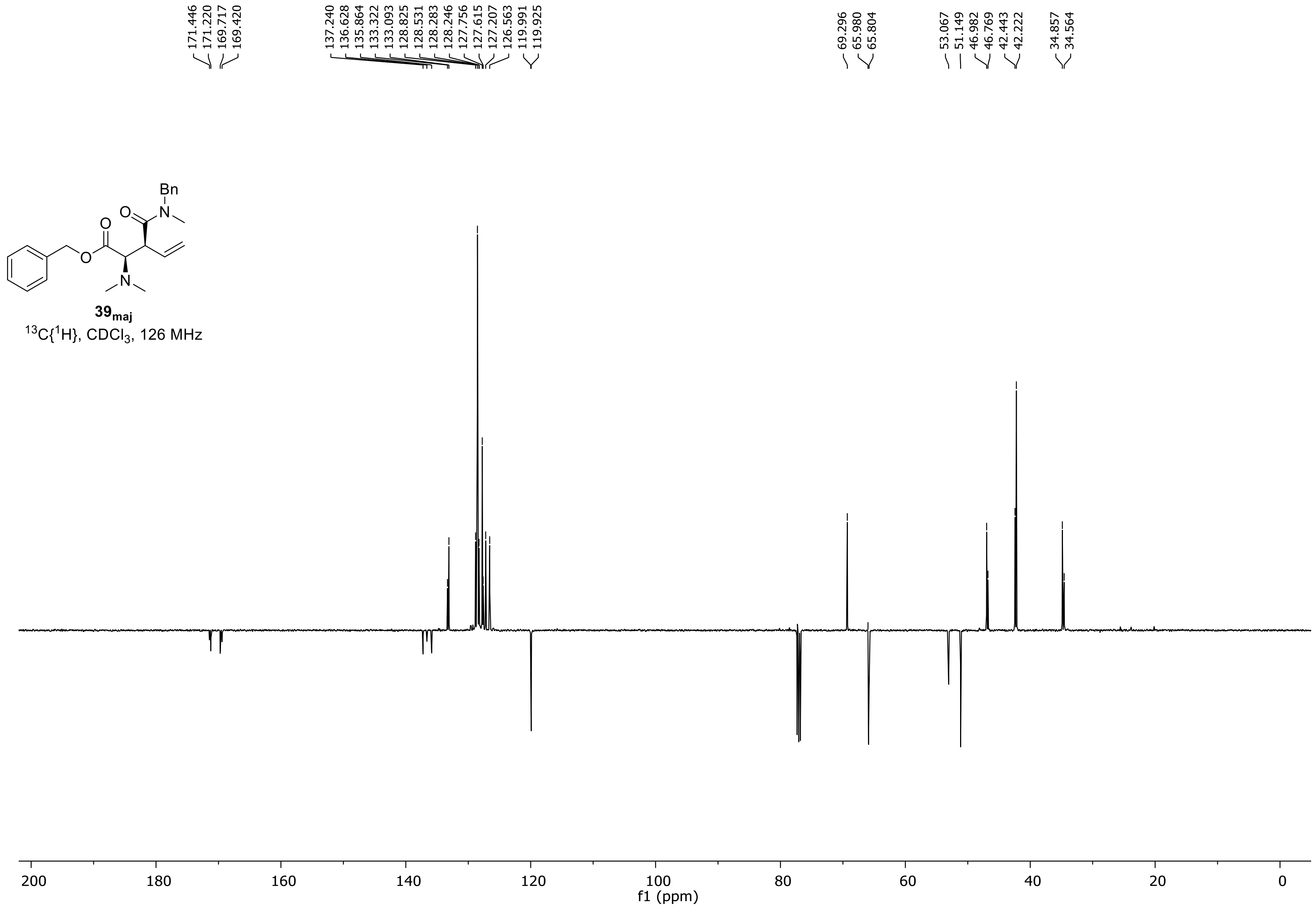
171.446
171.220
169.717
169.420
137.240
136.628
135.864
133.322
133.093
128.825
128.531
128.283
128.246
127.756
127.615
127.207
126.563
119.991
119.925
69.296
65.980
65.804
53.067
51.149
46.982
46.769
42.443
42.222
34.857
34.564
Bn
O
N
O
O
N
39maj
13C{1H}, CDCl3, 126 MHz
200
180
160
140
120
100
80
60
40
20
0
f1 (ppm)

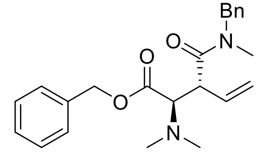

39_{min}

^1H, $CDCl_3$, 500 MHz

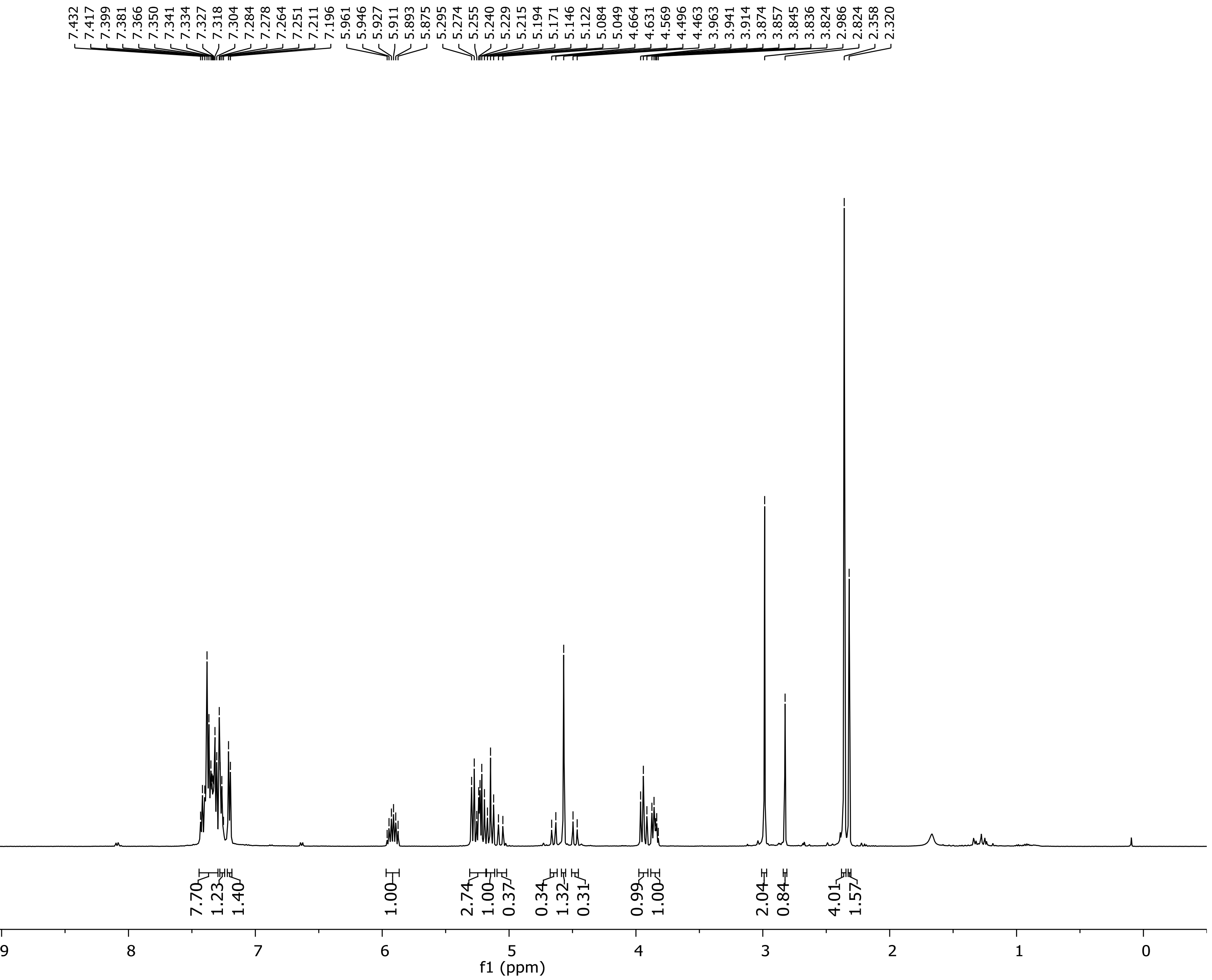

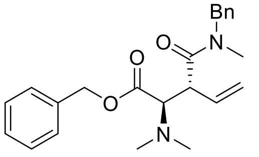

39_{min}

$^{13}C\{^{1}H\}$, $CDCl_3$, 126 MHz

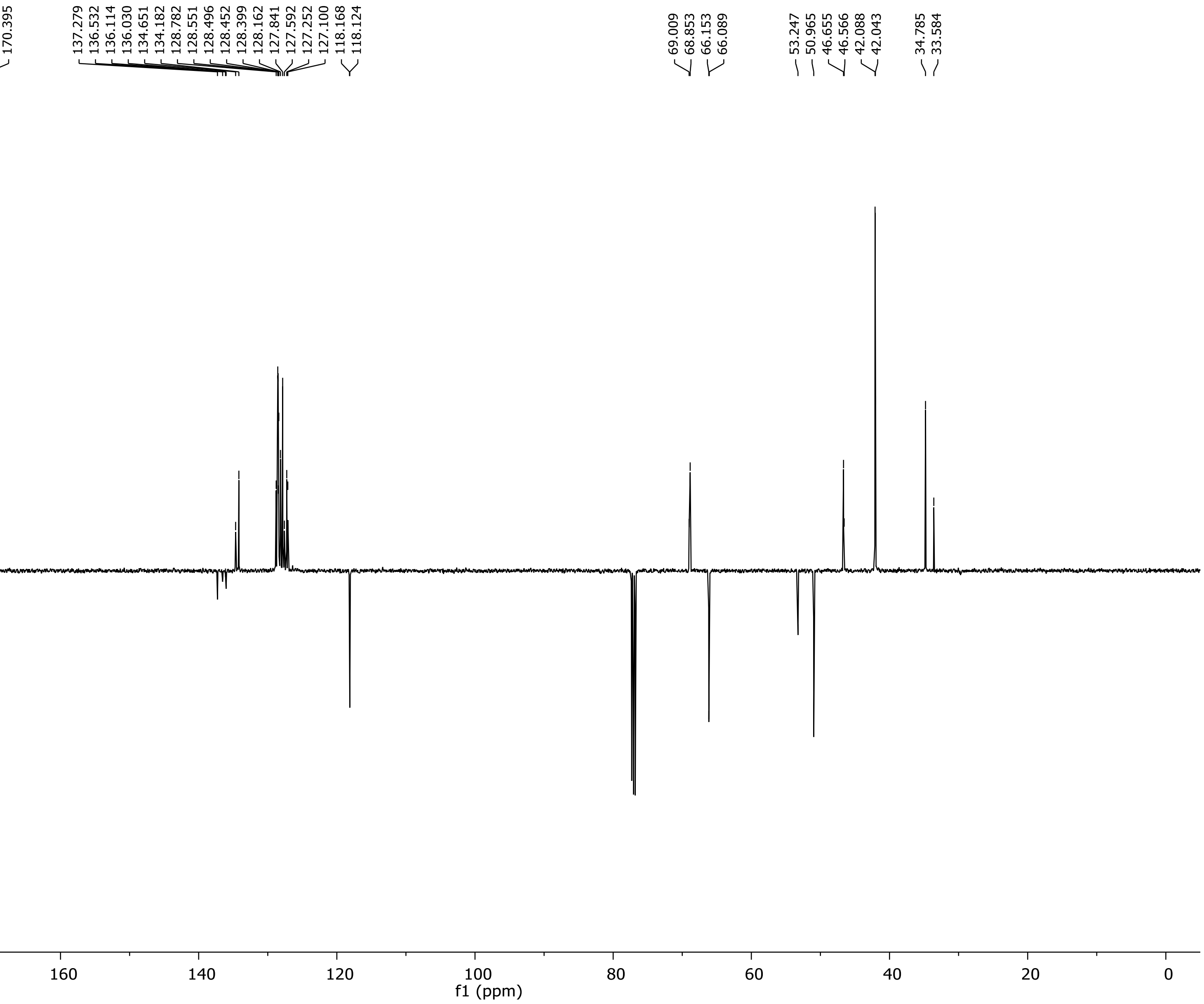

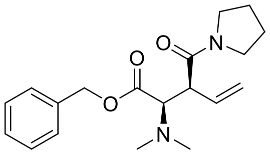

40_{maj}

^{1}H, $CDCl_3$, 500 MHz

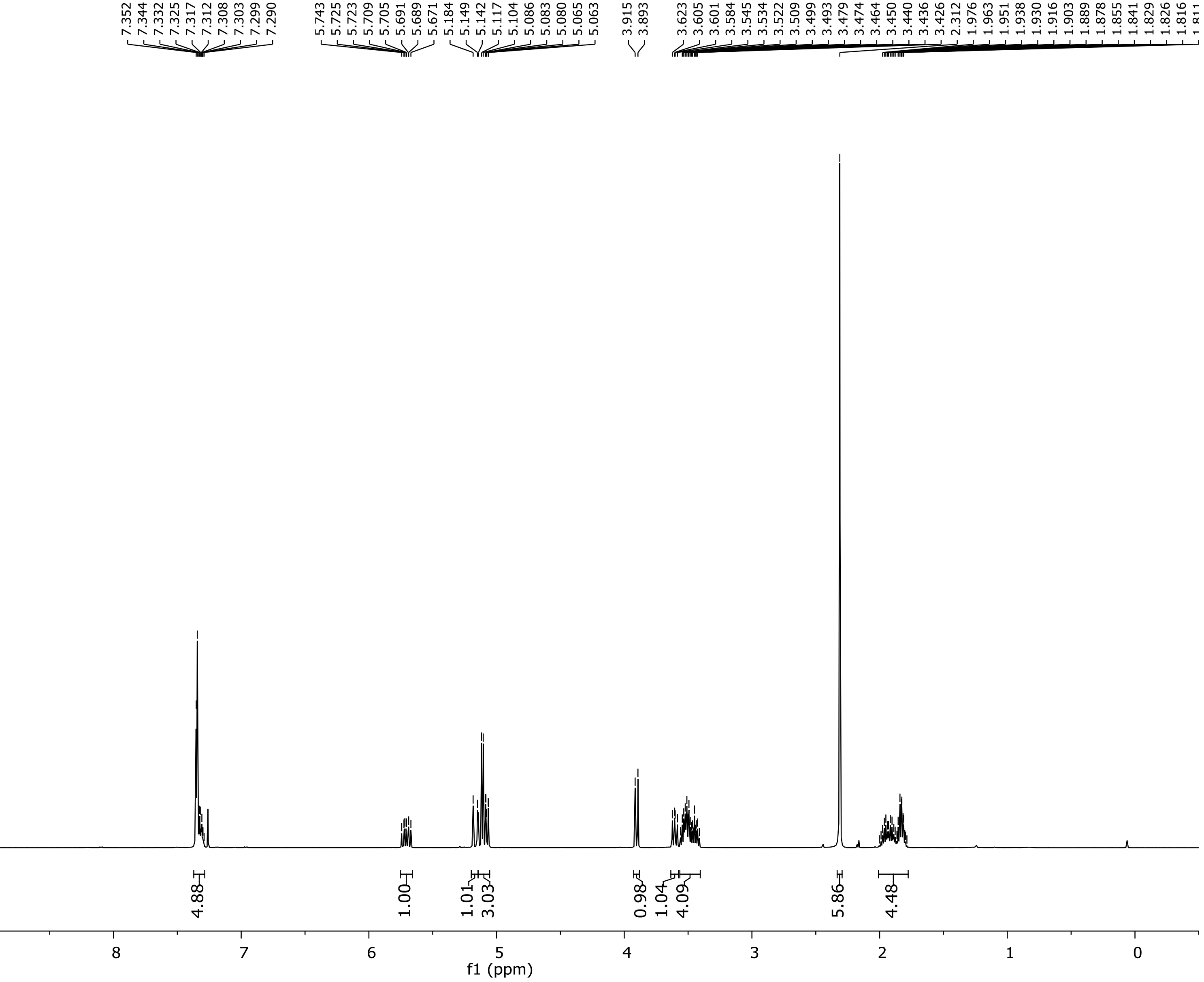

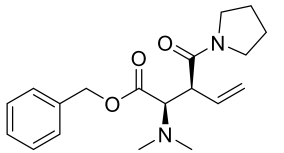

40$_{maj}$

$^{13}C\{^1H\}$, $CDCl_3$, 126 MHz

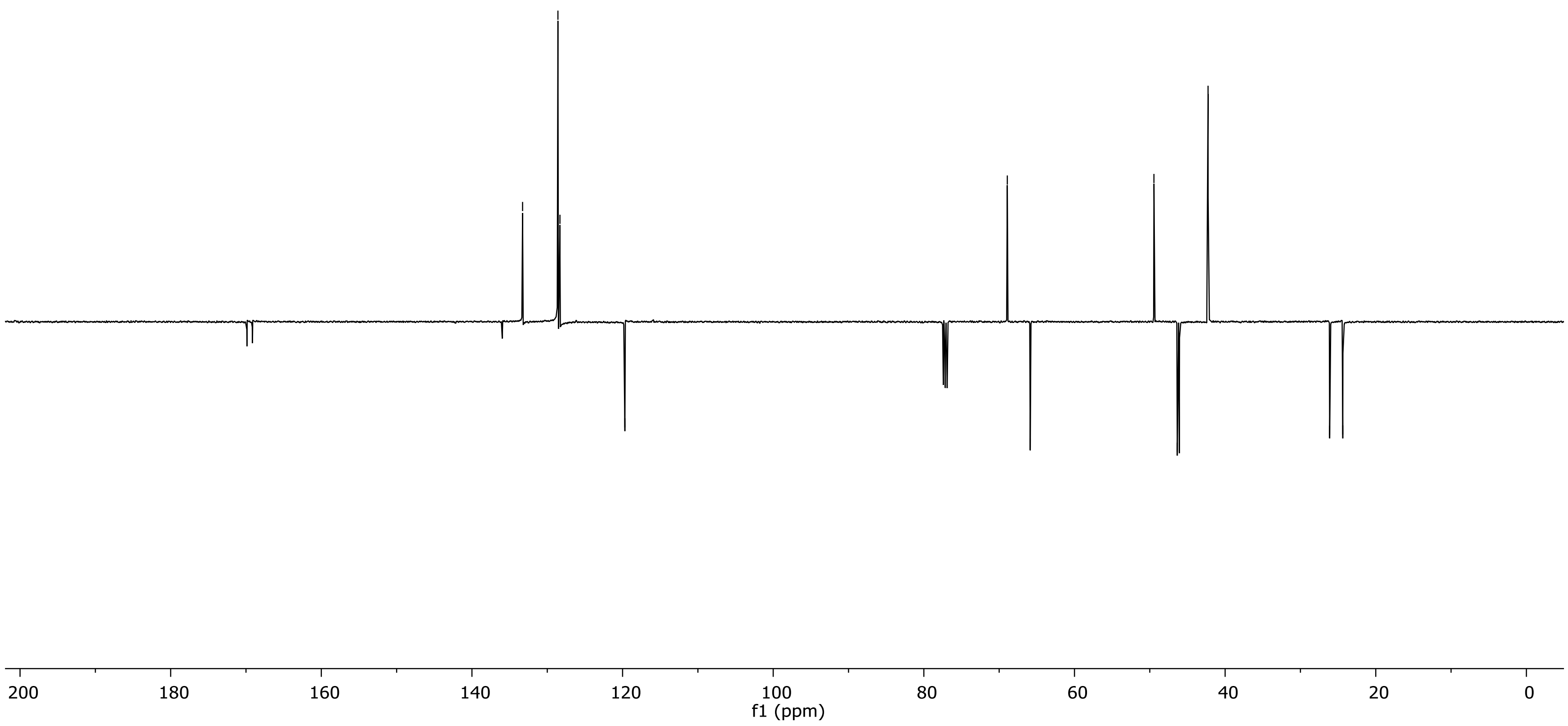

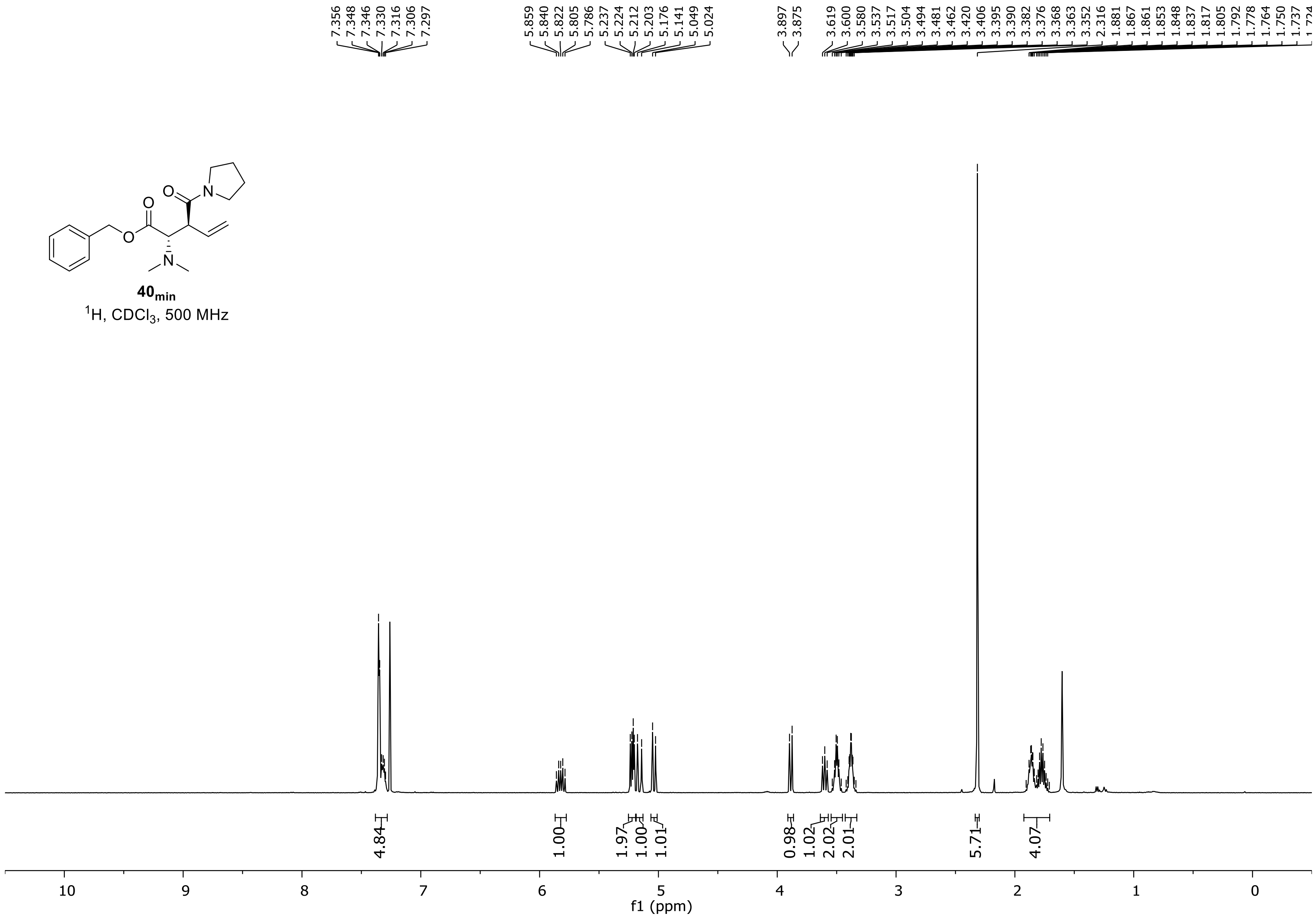

40_{min}

^{1}H, $CDCl_3$, 500 MHz

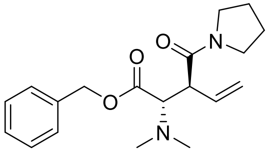

40$_{min}$

$^{13}C\{^1H\}$, $CDCl_3$, 126 MHz

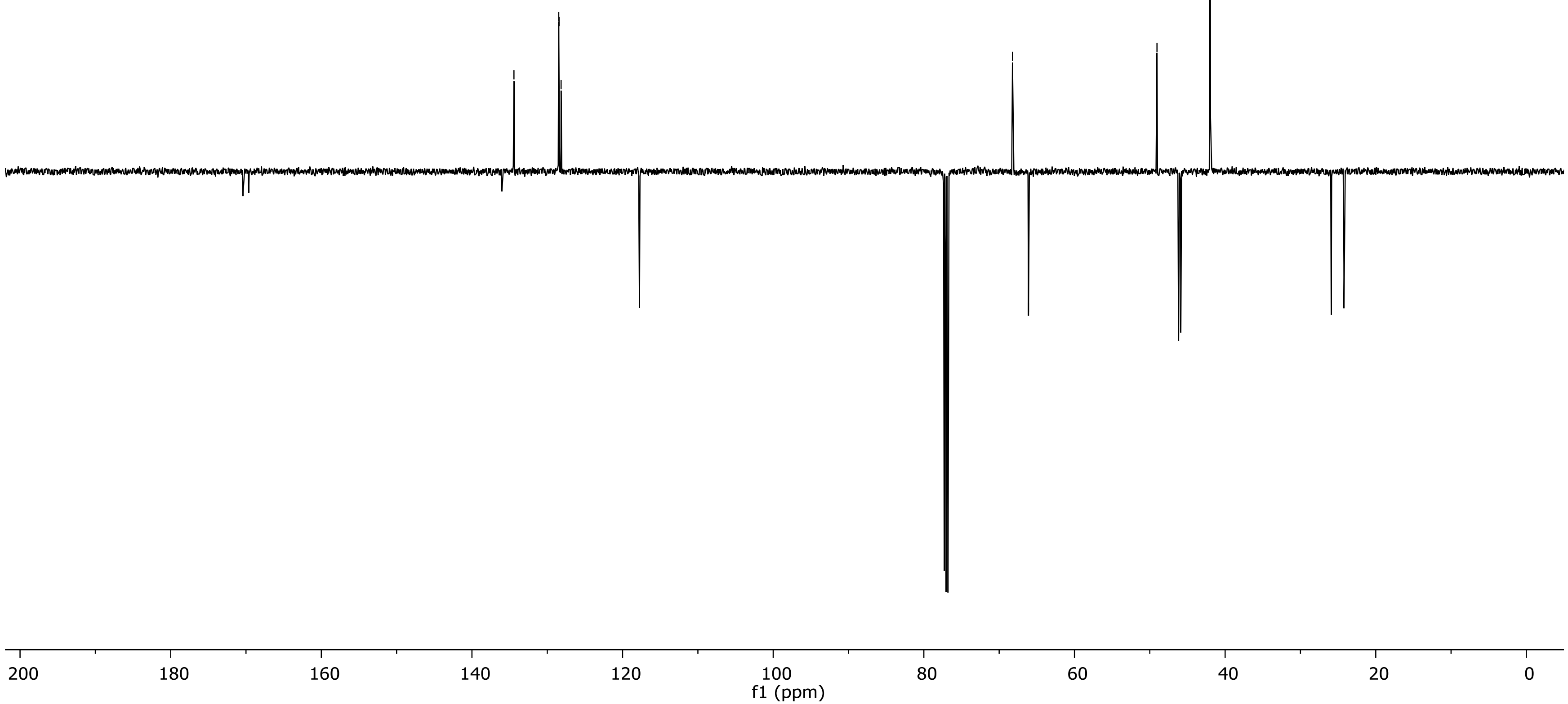

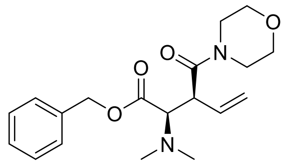

41$_{maj}$
^{1}H, $CDCl_3$, 500 MHz

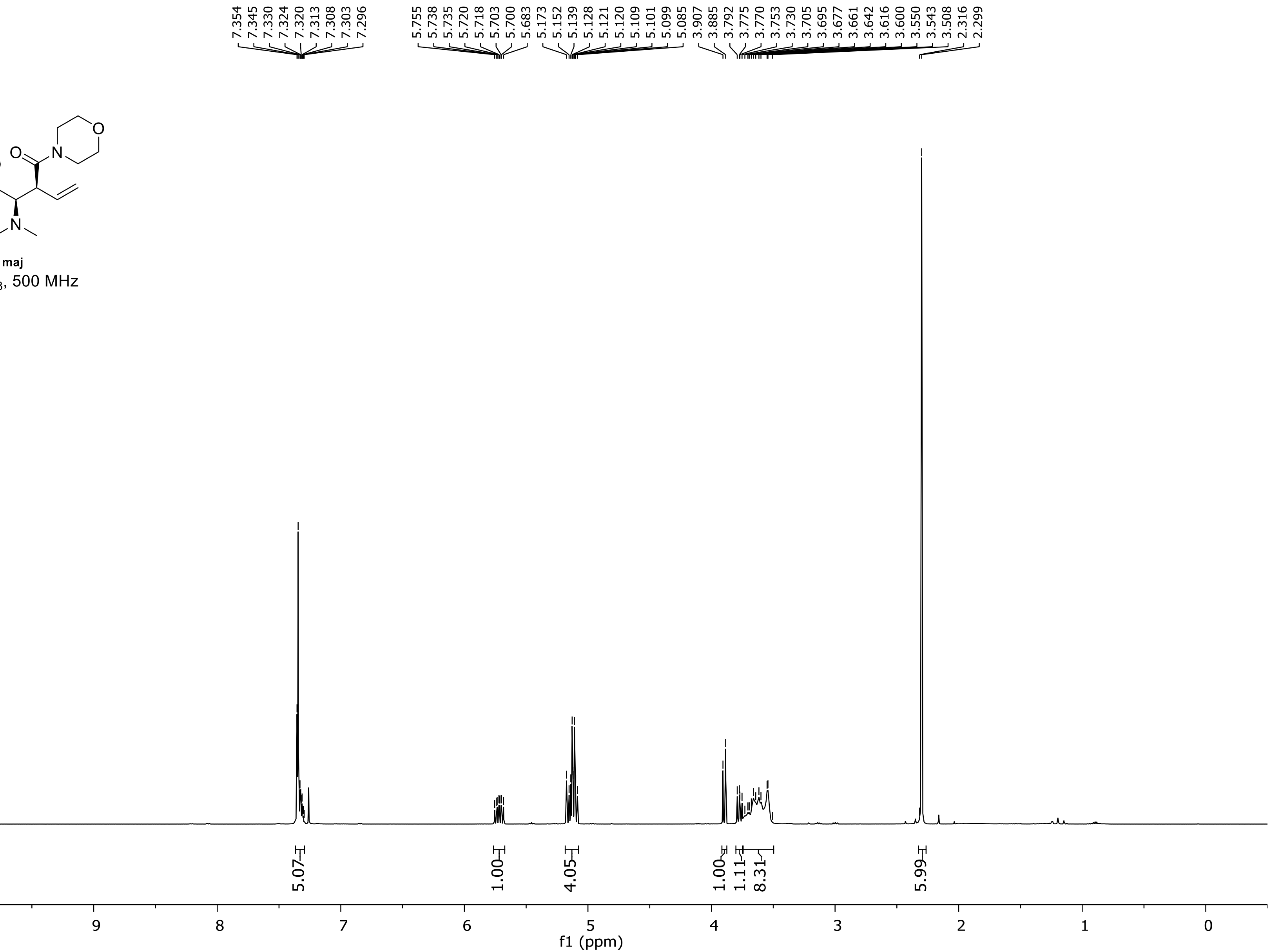

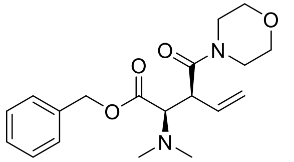

41$_{maj}$

$^{13}C\{^1H\}$, $CDCl_3$, 126 MHz

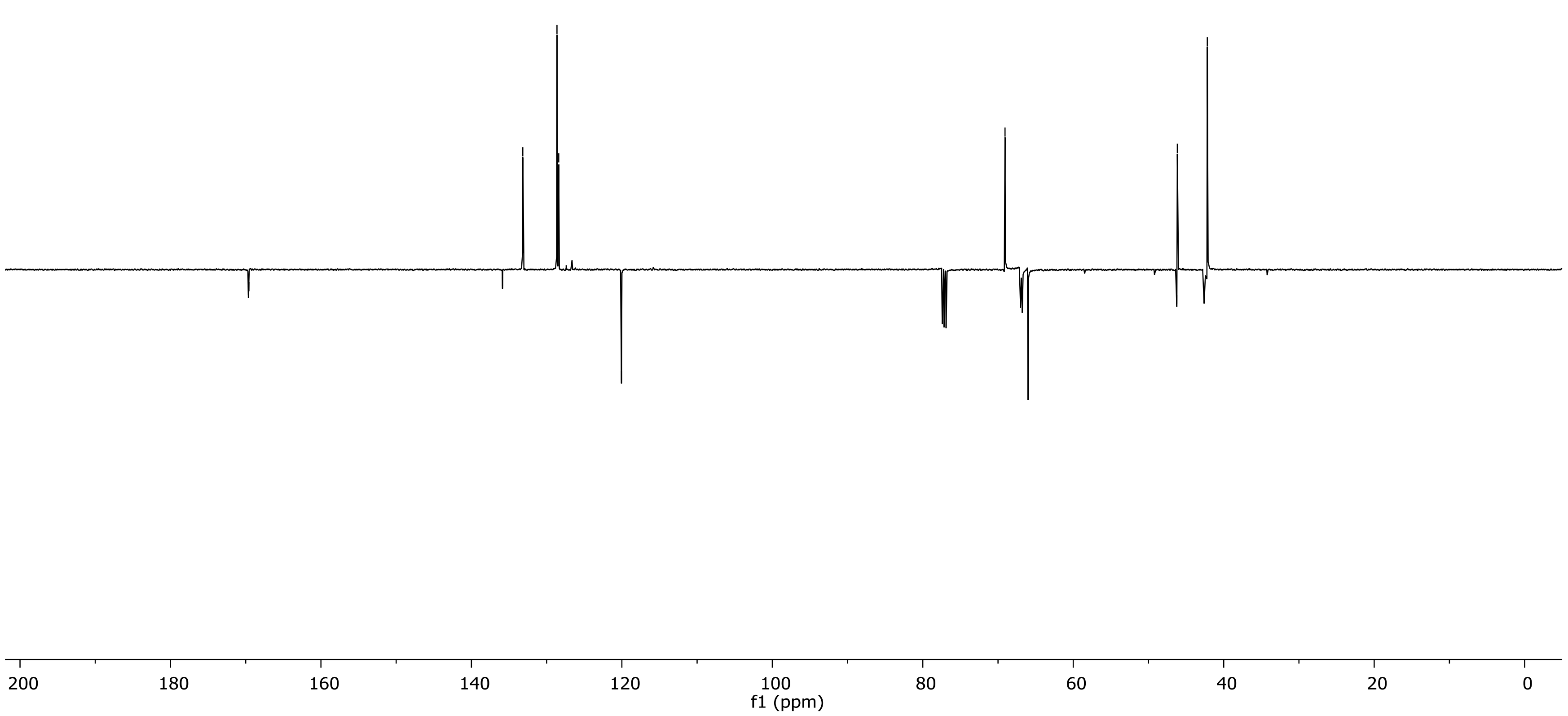

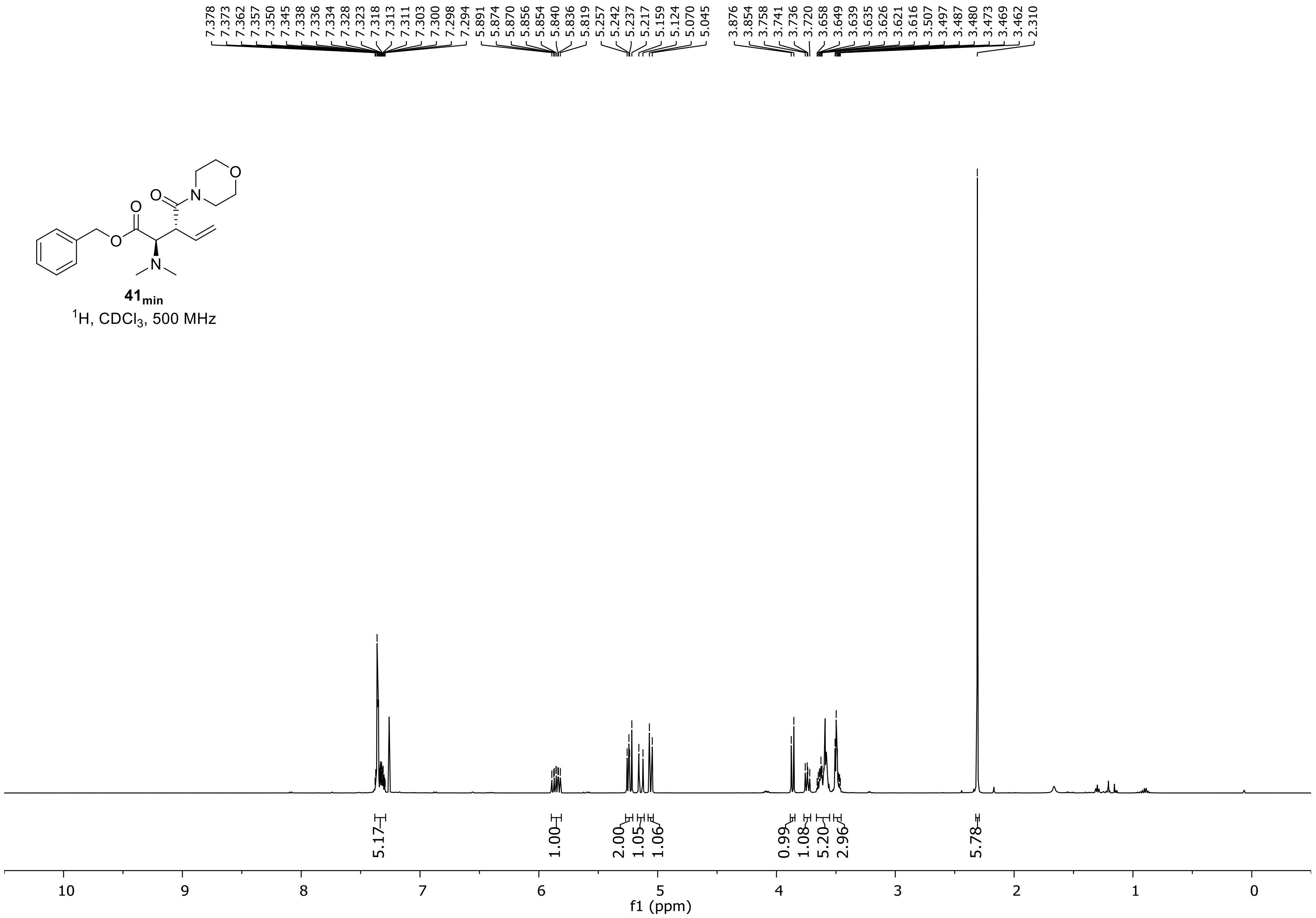
7.378 7.373 7.362 7.357 7.350 7.345 7.338 7.336 7.334 7.328 7.323 7.318 7.313 7.311 7.303 7.300 7.298 7.294 5.891 5.874 5.870 5.856 5.854 5.840 5.836 5.819 5.257 5.242 5.237 5.217 5.159 5.124 5.070 5.045 3.876 3.854 3.758 3.741 3.736 3.720 3.658 3.649 3.639 3.635 3.626 3.621 3.616 3.507 3.497 3.487 3.480 3.473 3.469 3.462 2.310
O
N
O
O
O
N
41min
1H, CDCl3, 500 MHz
5.17
1.00
2.00
1.05
1.06
0.99
1.08
5.20
2.96
5.78
10 9 8 7 6 5 4 3 2 1 0
f1 (ppm)

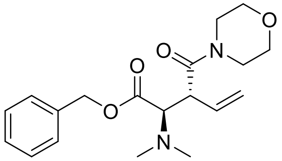

41_{min}

$^{13}C\{^1H\}$, $CDCl_3$, 126 MHz

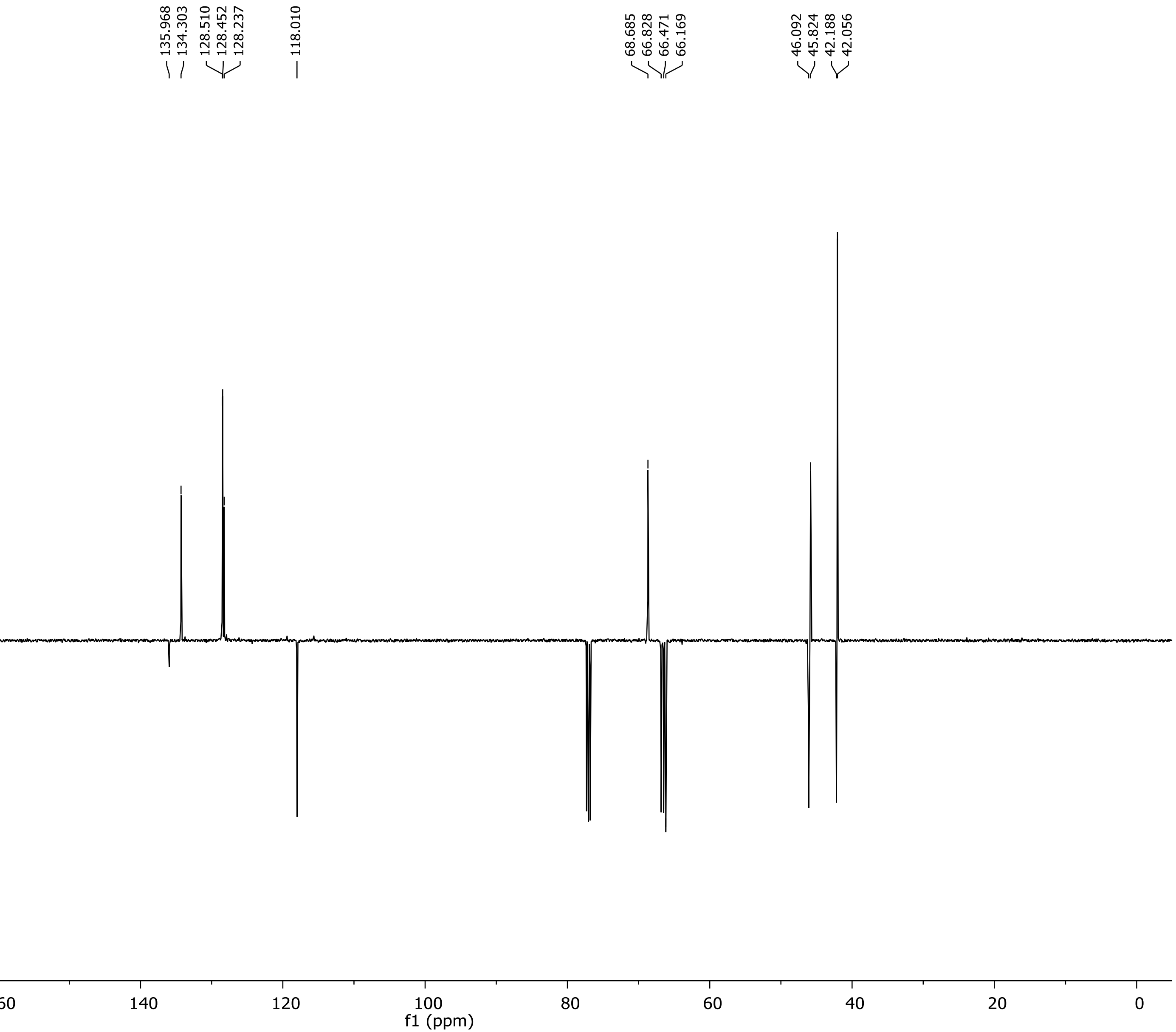

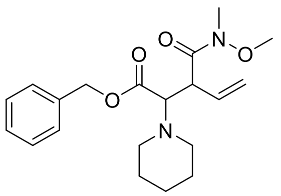

42

^{1}H, CDCl$_3$,500 MHz

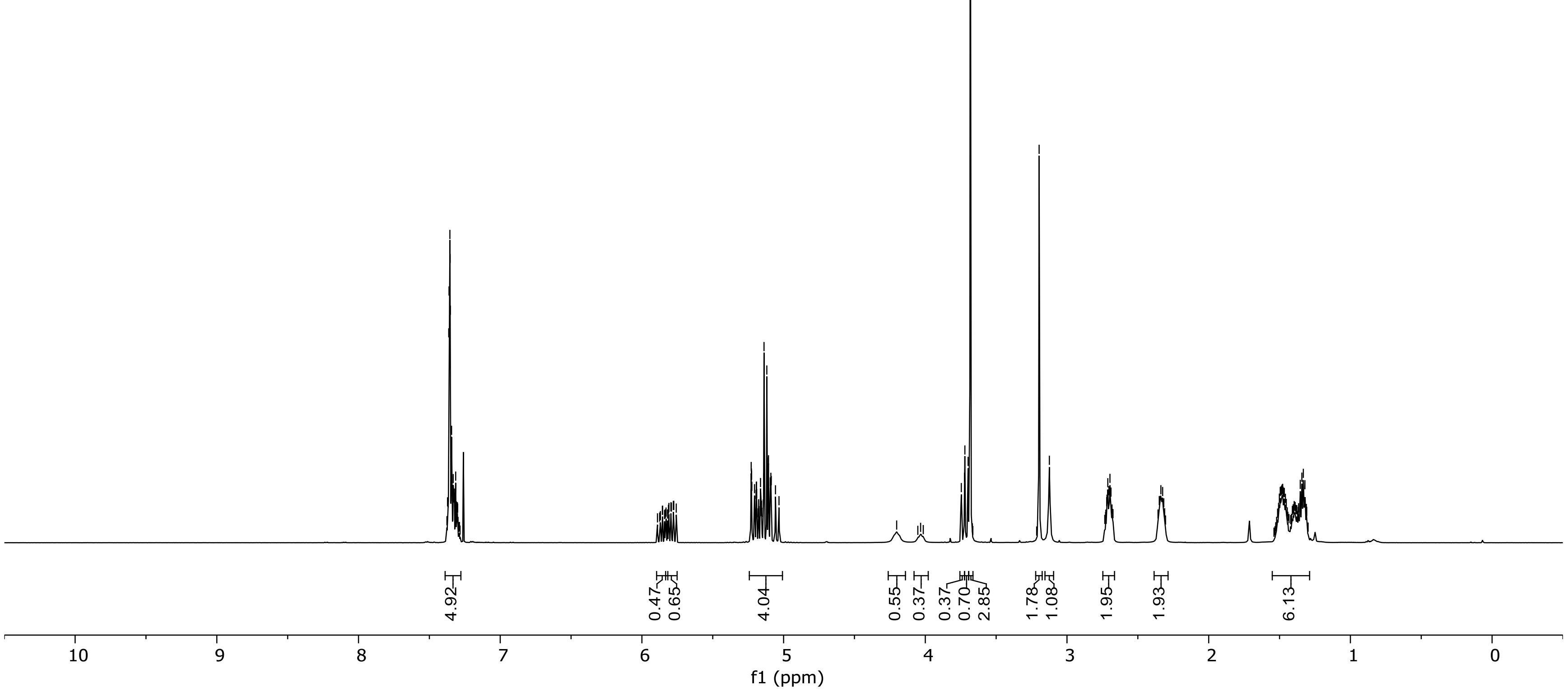

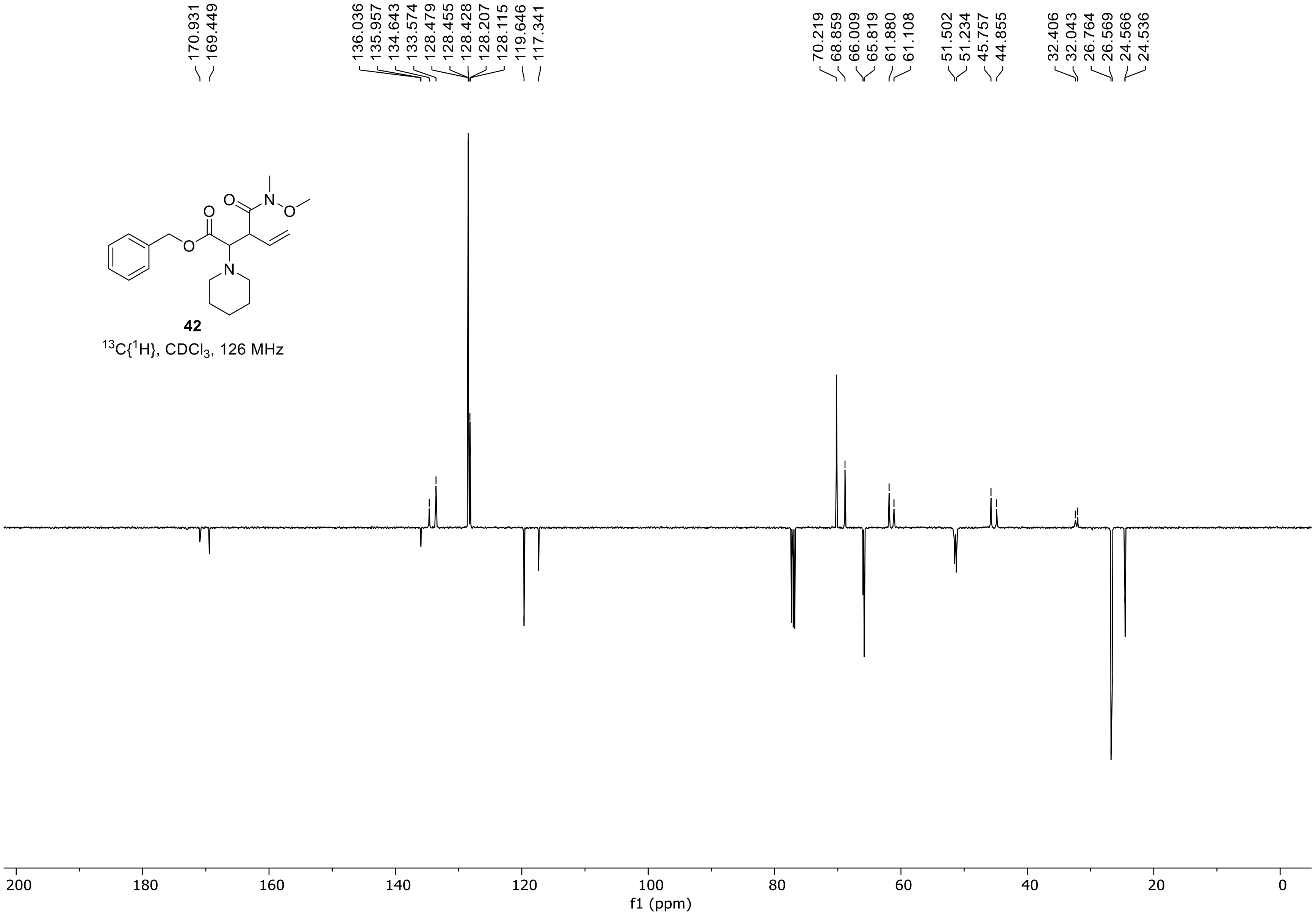
170.931
169.449
136.036
135.957
134.643
133.574
128.479
128.455
128.428
128.207
128.115
119.646
117.341
70.219
68.859
66.009
65.819
61.880
61.108
51.502
51.234
45.757
44.855
32.406
32.043
26.764
26.569
24.566
24.536
O
N
O
O
O
N
42
$^{13}C\{^{1}H\}$, $CDCl_3$, 126 MHz
200
180
160
140
120
100
80
60
40
20
0
f1 (ppm)

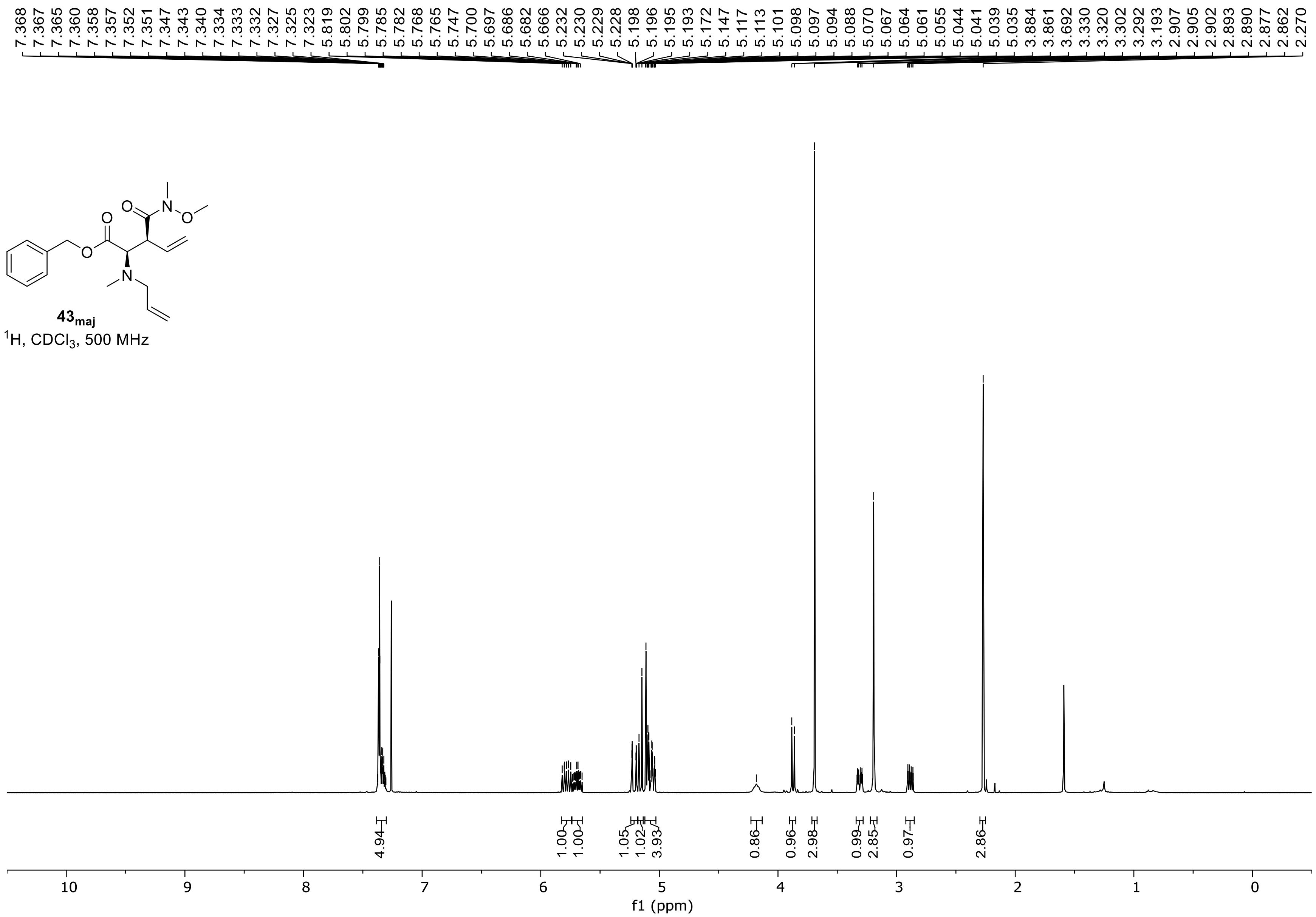

43_{maj}

^1H, $CDCl_3$, 500 MHz

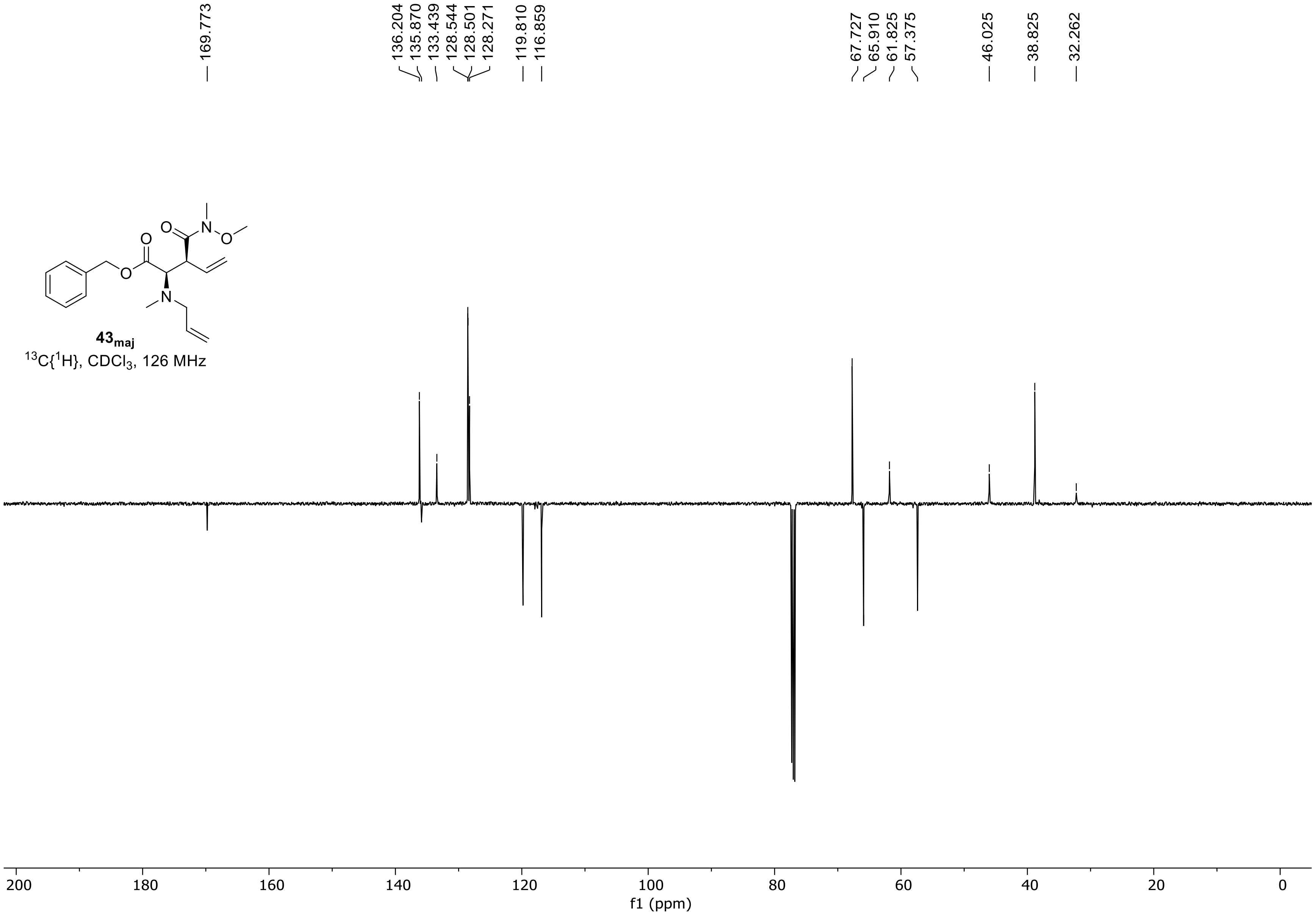
169.773
136.204
135.870
133.439
128.544
128.501
128.271
119.810
116.859
67.727
65.910
61.825
57.375
46.025
38.825
32.262
43maj
13C{1H}, CDCl3, 126 MHz
200 180 160 140 120 100 80 60 40 20 0
f1 (ppm)

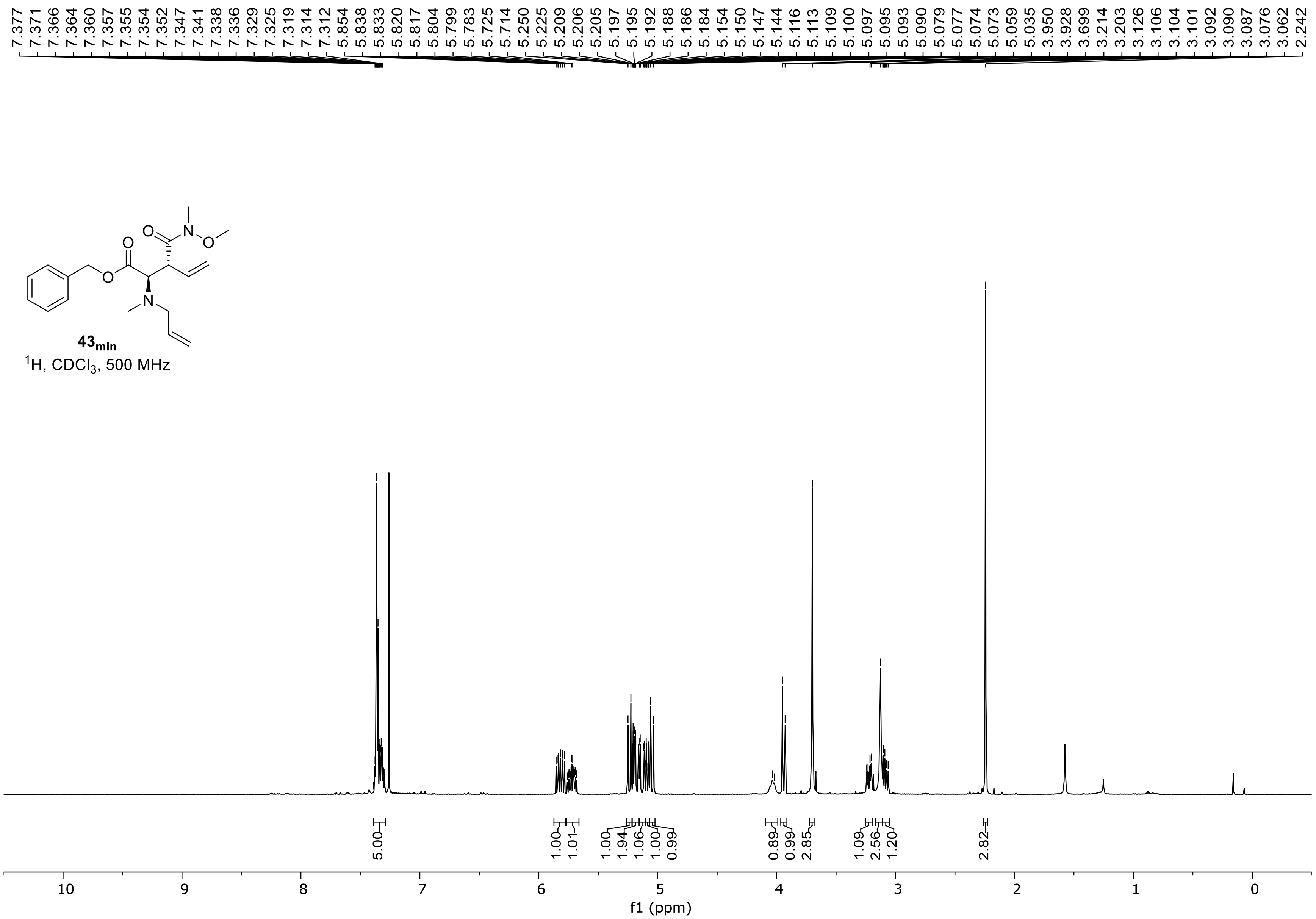

43$_{min}$

^{1}H, CDCl$_3$, 500 MHz

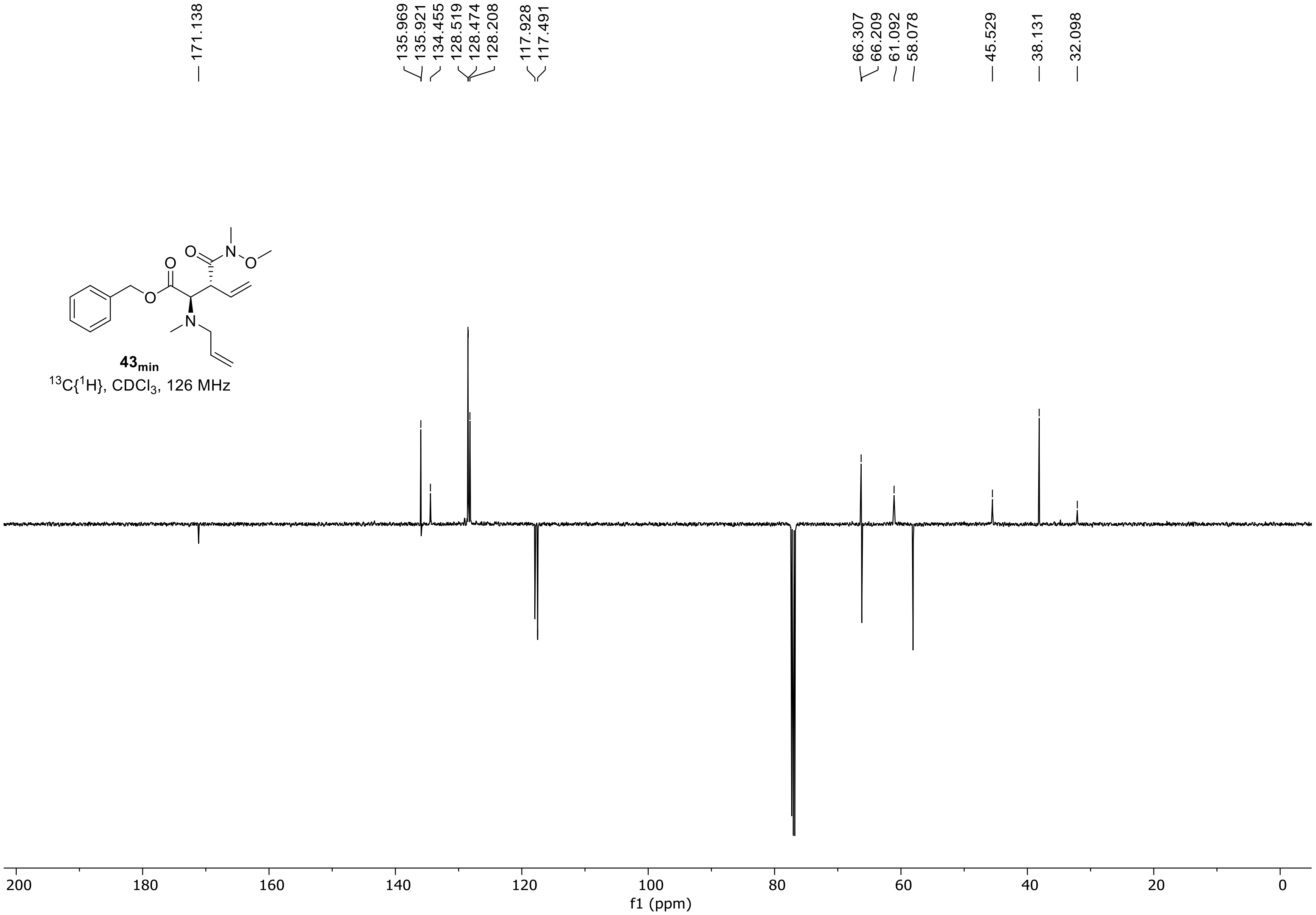
171.138
135.969
135.921
134.455
128.519
128.474
128.208
117.928
117.491
66.307
66.209
61.092
58.078
45.529
38.131
32.098
43min
13C{1H}, CDCl3, 126 MHz
200
180
160
140
120
100
80
60
40
20
0
f1 (ppm)

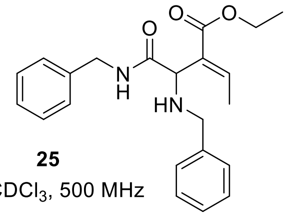

25

^1H, $CDCl_3$, 500 MHz

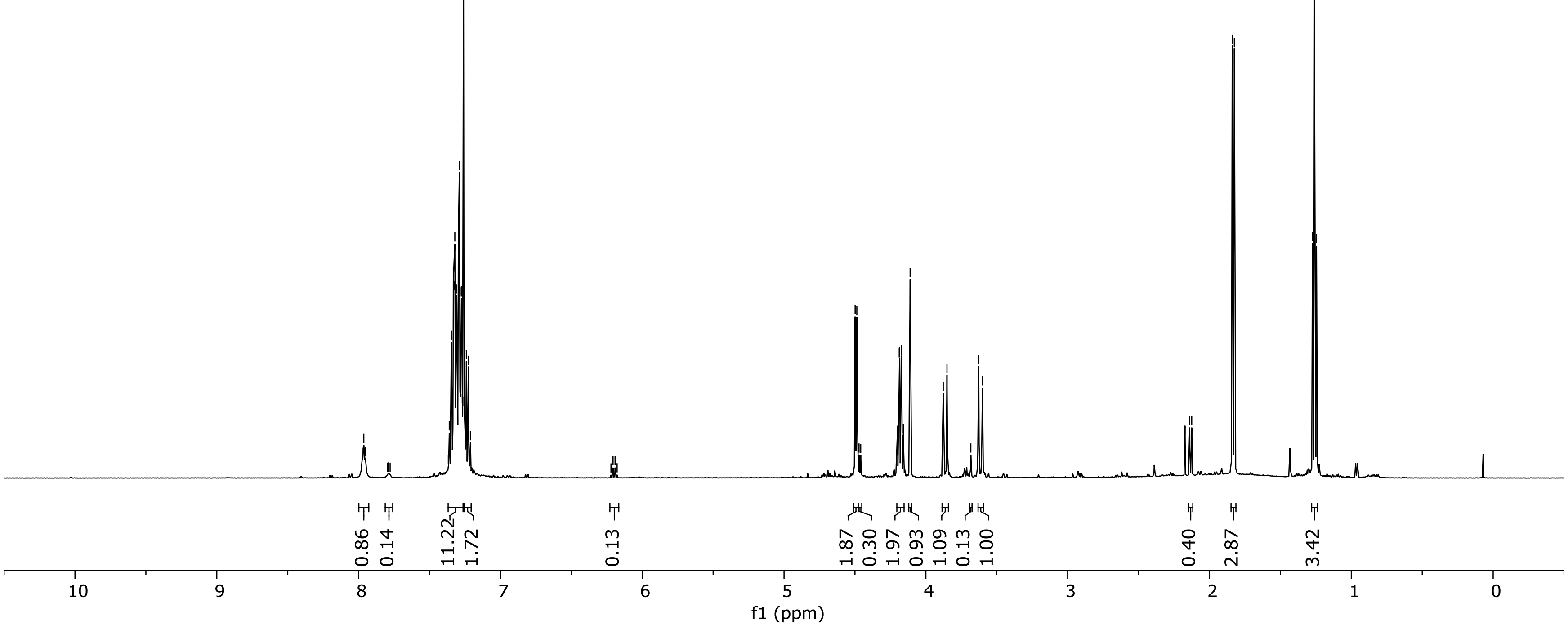

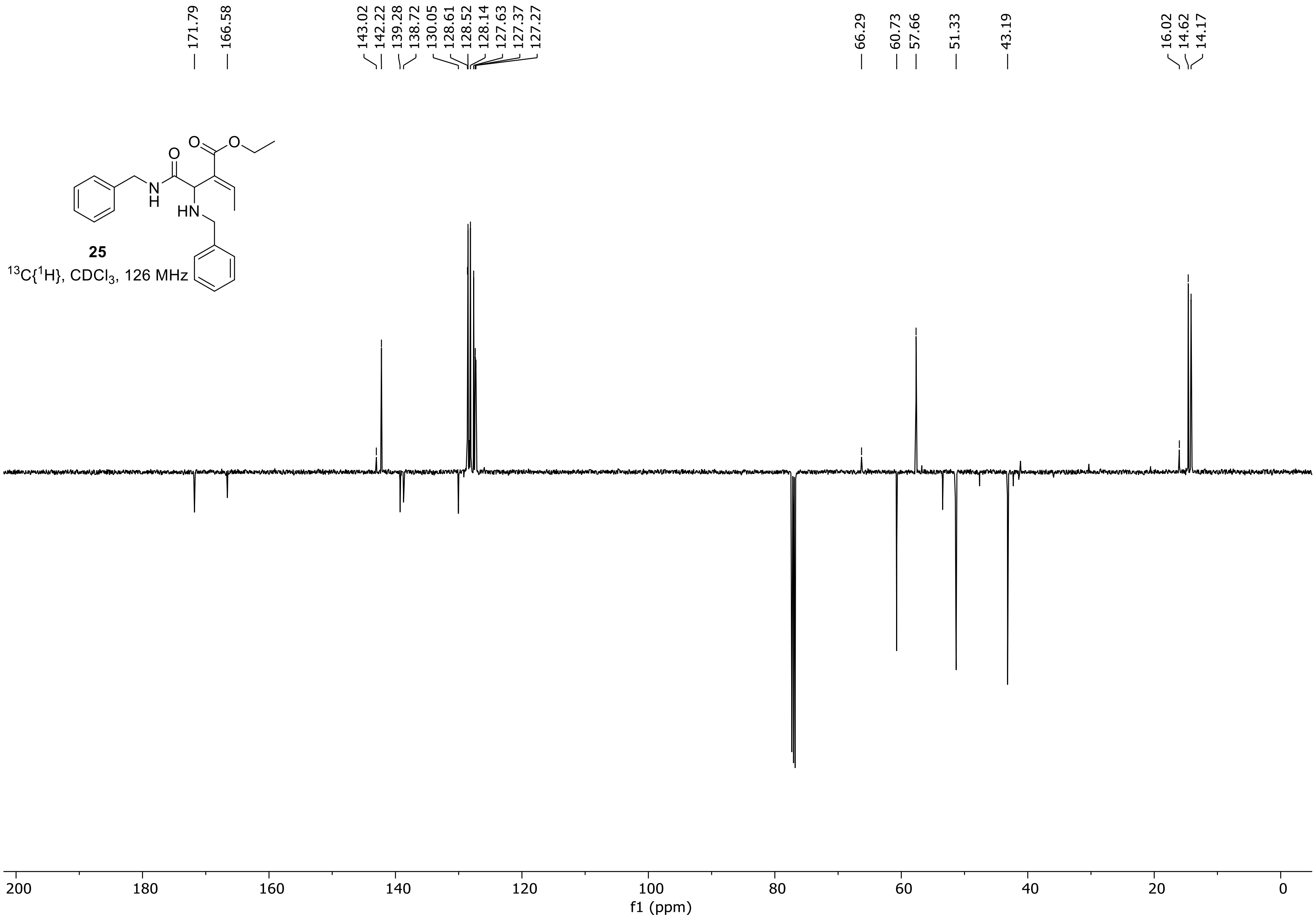

171.79
166.58
143.02
142.22
139.28
138.72
130.05
128.61
128.52
128.14
127.63
127.37
127.27
66.29
60.73
57.66
51.33
43.19
16.02
14.62
14.17
25
$^{13}C\{^1H\}$, $CDCl_3$, 126 MHz
200
180
160
140
120
100
80
60
40
20
0
f1 (ppm)

Appendix III. HPLC traces

HPLC Data for **18** (60:40 dr): Chiralcel OD-H, (*n*-hexane : *i*PrOH 99:1, flow rate 0.5 mLmin^{-1}, 254 nm, 30 °C) *major diastereoisomer*: t_R (2*S*,3*R*): 26.7 min, t_R (2*R*,3*S*): 29.5 min, 10:90 er; *minor diastereoisomer:* t_R (2*R*,3*R*): 25.0 min, t_R (2*S*,3*S*): 41.7 min, 70:30 er.

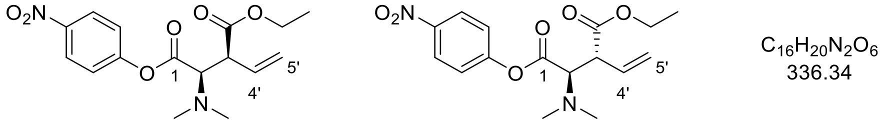

18$_{maj}$ **18**$_{min}$

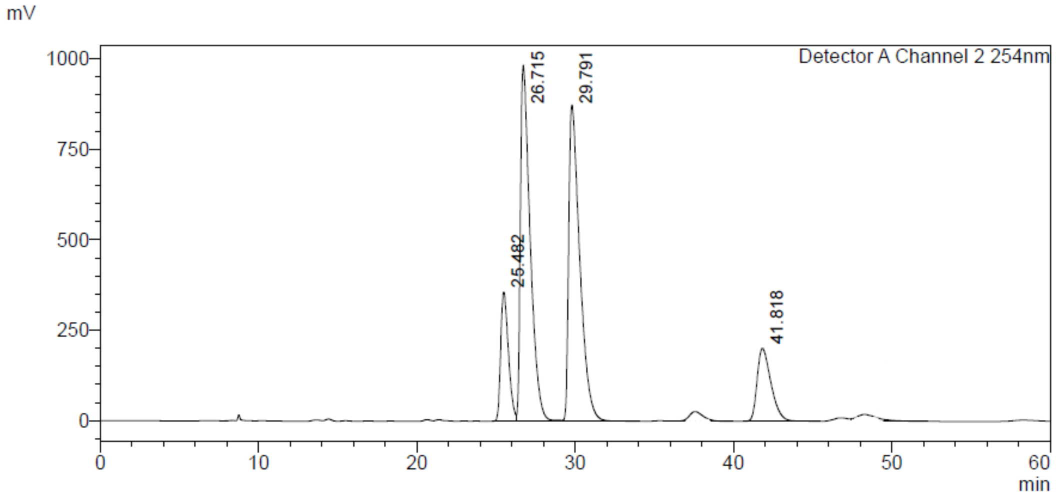

<Peak Table>

Detector A Channel 2 254 nm

Peak#	Ret. Time	Area%
1	25.482	11.098
2	26.715	38.744
3	29.791	38.926
4	41.818	11.232
Total		100

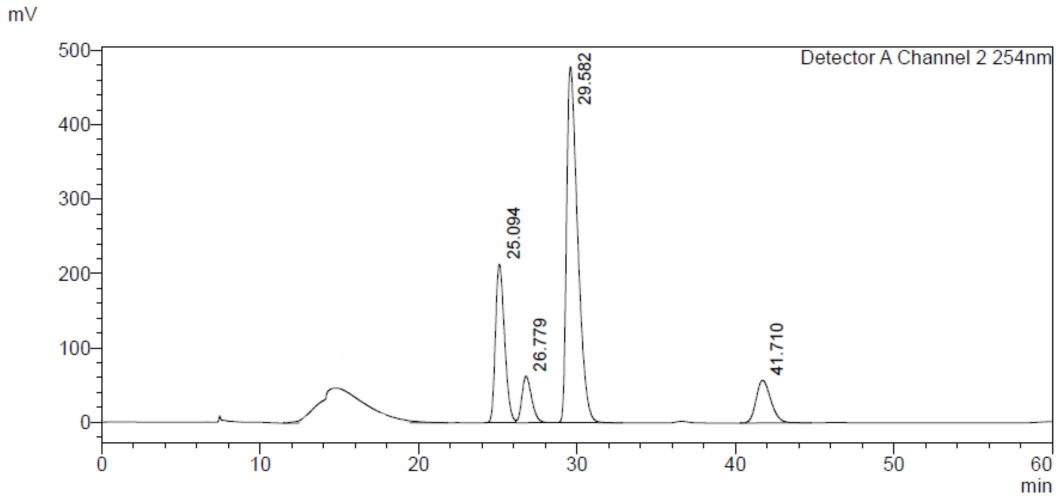

<Peak Table>

Detector A Channel 2 254 nm

Peak#	Ret. Time	Area%
1	25.094	22.269
2	26.779	6.989
3	29.582	61.022
4	41.710	9.720
Total		100

HPLC Data for **27**$_{maj}$: Chiralcel OD-H, (*n*-hexane : *i*PrOH 95:5, flow rate 1.0 mLmin^{-1}, 211 nm, 40 °C) t$_R$ (2*S*,3*R*) 14.7 min, t$_R$ (2*R*,3*S*) 19.5 min, 9:91 er.

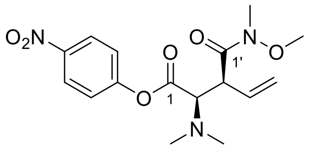

27$_{maj}$

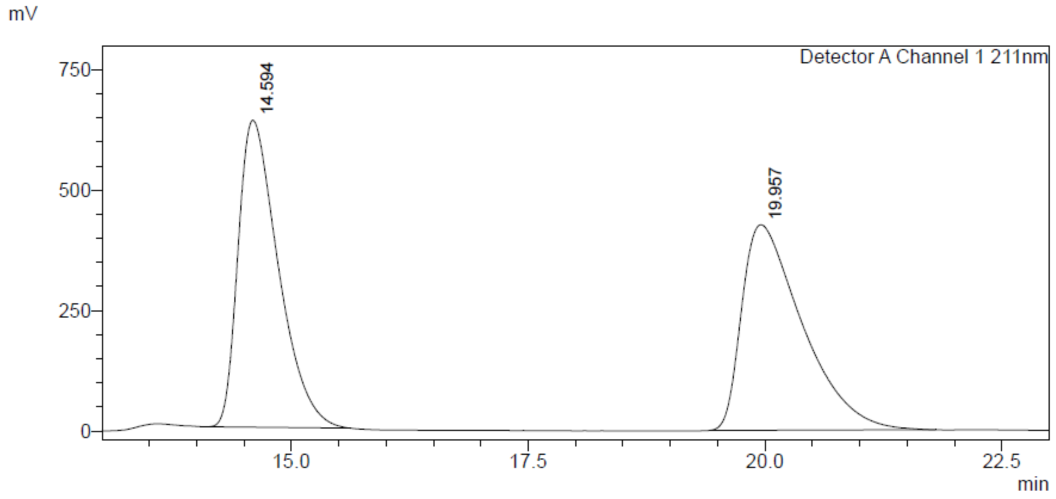

<Peak Table>

Detector A Channel 1 211nm

Peak#	Ret. Time	Area%
1	14.594	49.792
2	19.957	50.208
Total		100.000

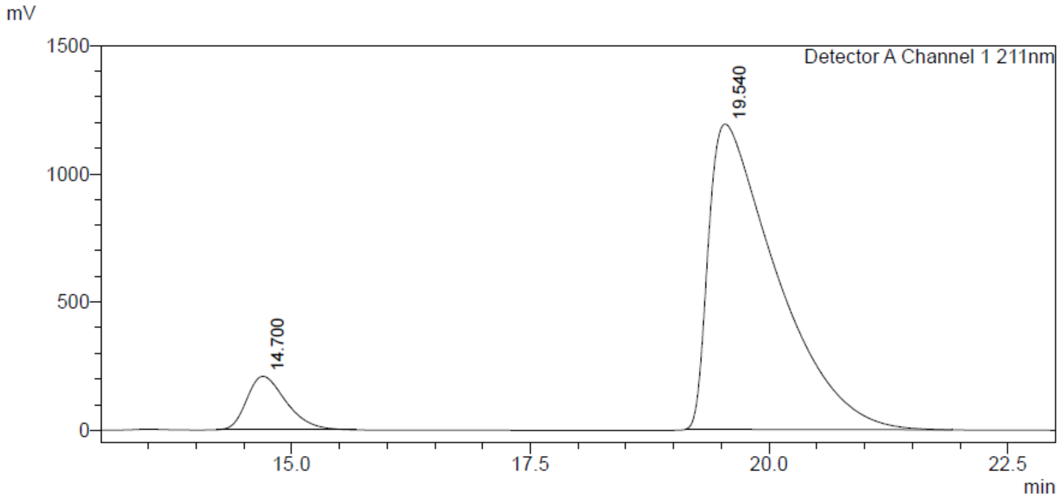

<Peak Table>

Detector A Channel 1 211nm

Peak#	Ret. Time	Area%
1	14.700	9.189
2	19.540	90.811
Total		100.000

HPLC Data for **27**$_{min}$: Chiralcel OD-H, (*n*-hexane : *i*PrOH 95:5, flow rate 1.0 mLmin^{-1}, 211 nm, 40 °C) t_R (2*R*,3*R*): 10.1 min, t_R (2*S*,3*S*): 13.3 min, 83:17 er.

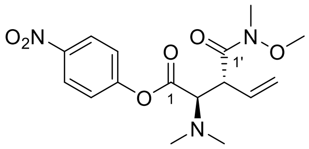

27$_{min}$

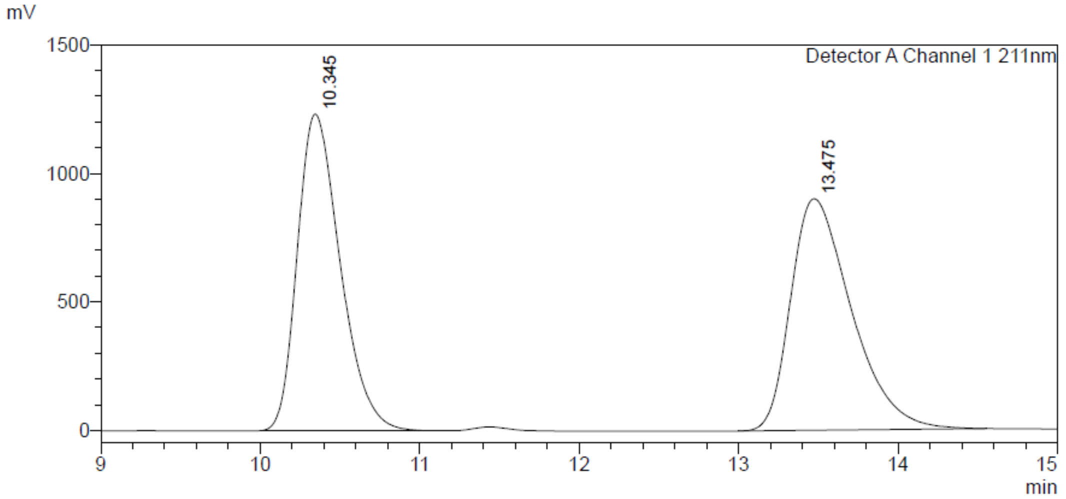

<Peak Table>

Detector A Channel 1 211nm

Peak#	Ret. Time	Area%
1	10.345	49.133
2	13.475	50.867
Total		100.000

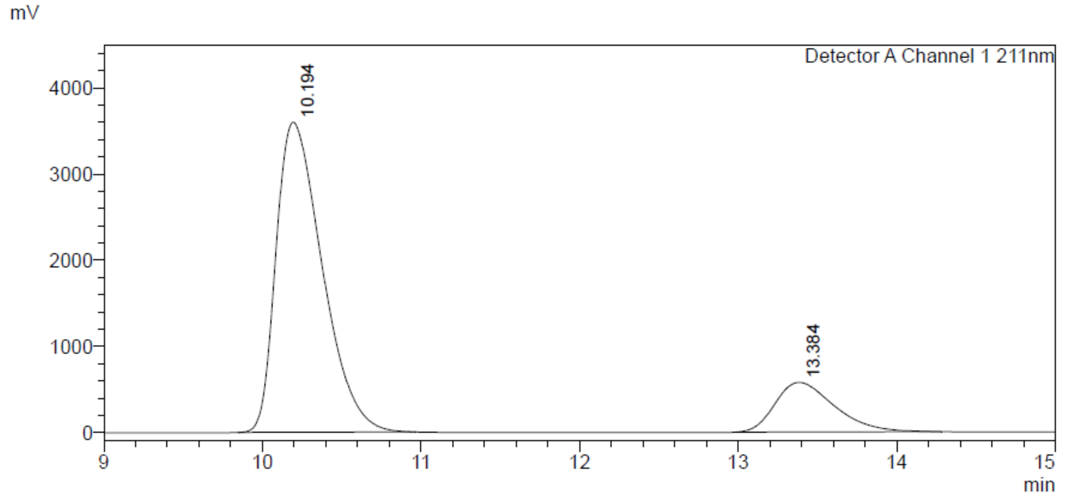

<Peak Table>

Detector A Channel 1 211nm

Peak#	Ret. Time	Area%
1	10.194	82.951
2	13.384	17.049
Total		100.000

HPLC Data for **28**$_{maj}$: Chiralpak ID, (*n*-hexane : *i*PrOH 88:12, flow rate 1.5 mLmin^{-1}, 211 nm, 40 °C) t$_R$ (2*S*,1′*R*): 20.0 min, t$_R$ (2*R*,1′*S*) 13.9 min, 90:10 er.

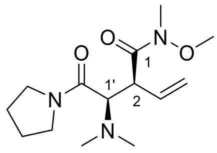

28$_{maj}$

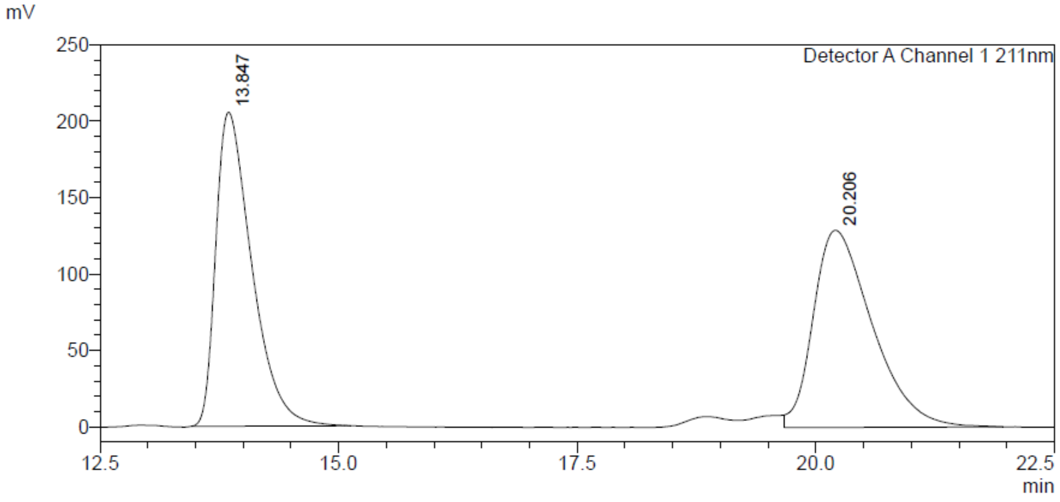

<Peak Table>

Detector A Channel 1 211nm

Peak#	Ret. Time	Area%
1	13.847	49.250
2	20.206	50.750
Total		100.000

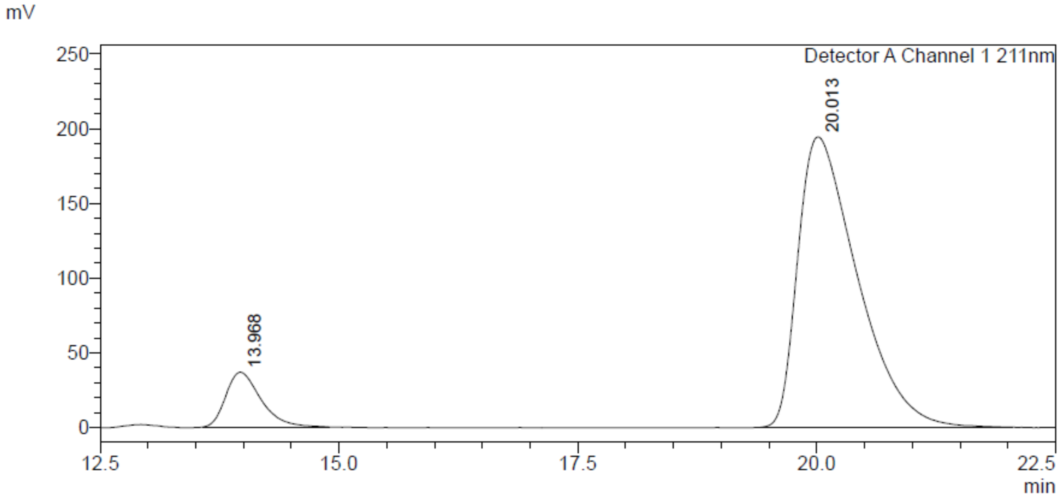

<Peak Table>

Detector A Channel 1 211nm

Peak#	Ret. Time	Area%
1	13.968	10.001
2	20.013	89.999
Total		100.000

HPLC Data for **28ₘᵢₙ**: Chiralpak ID, (*n*-hexane : *i*PrOH 88:12, flow rate 1.5 mLmin^{-1}, 211 nm, 40 °C) t_R (major) 12.5 min, t_R (minor) 6.2 min, 84:16 er.

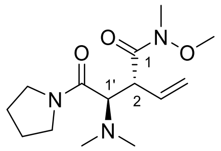

28$_{min}$

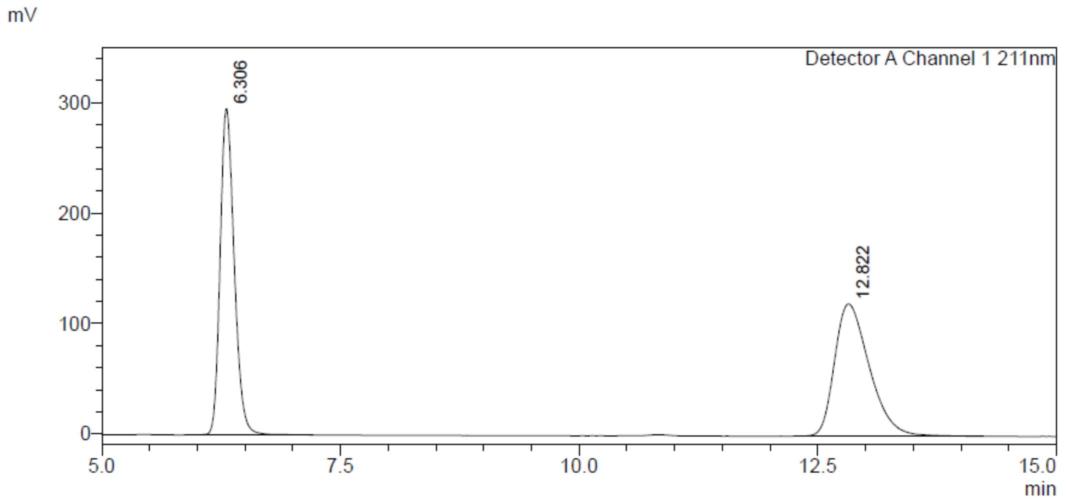

<Peak Table>

Detector A Channel 1 211nm

Peak#	Ret. Time	Area%
1	6.306	50.079
2	12.822	49.921
Total		100.000

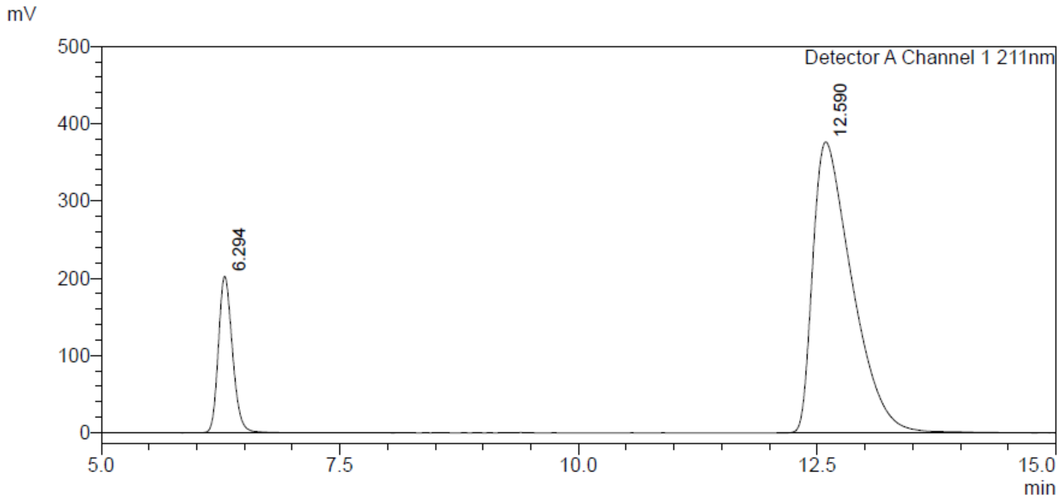

<Peak Table>

Detector A Channel 1 211nm

Peak#	Ret. Time	Area%
1	6.294	16.209
2	12.590	83.791
Total		100.000

HPLC Data for **29_{maj}:** Chiralcel OD-H (95:5 hexane : *i*PrOH, flow rate 1 mlmin^{-1}, 211 nm, 40 °C) t_R (2*R*,3*S*): 8.3 min, t_R (2*S*,3*R*): 9.8 min, 87:13 er.

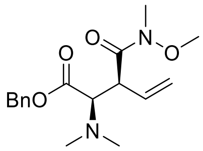

29_{maj}

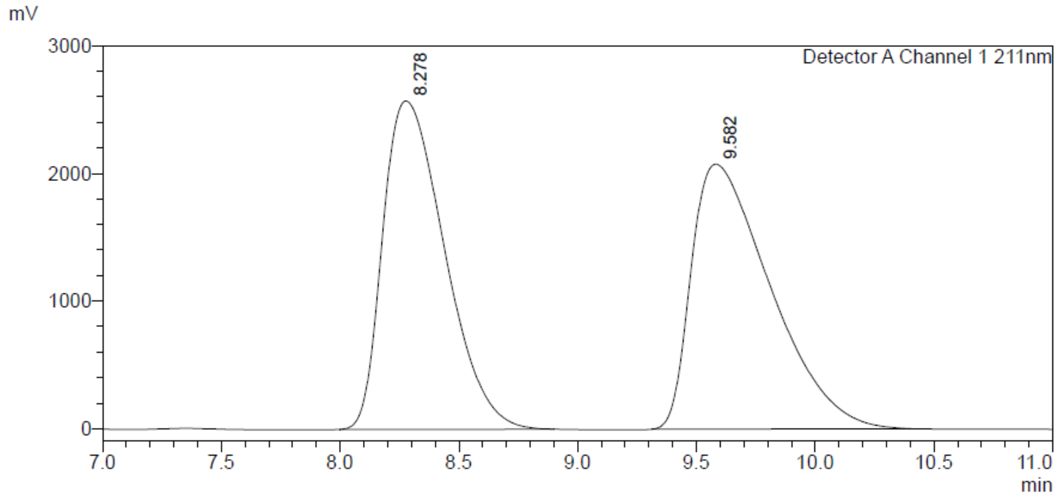

<Peak Table>

Detector A Channel 1 211nm

Peak#	Ret. Time	Area%
1	8.278	49.210
2	9.582	50.790
Total		100.000

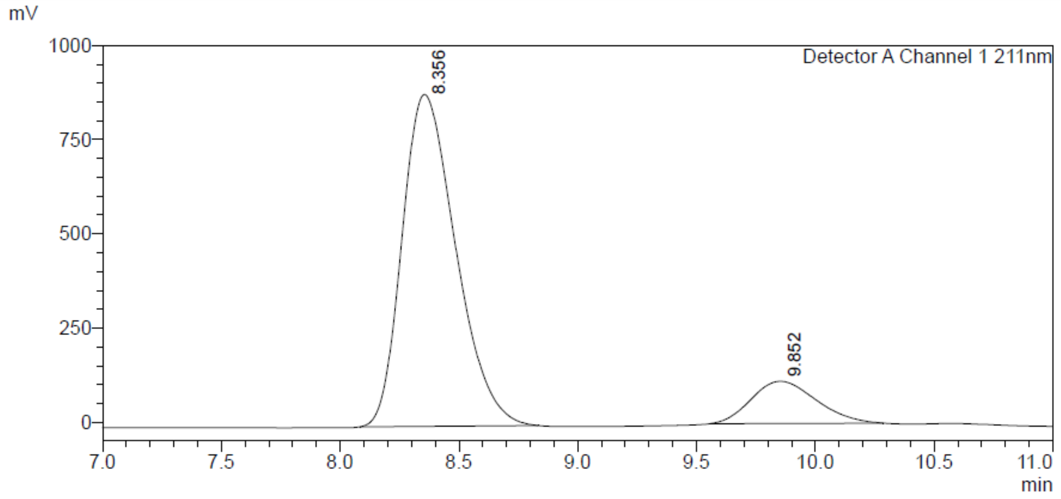

<Peak Table>

Detector A Channel 1 211nm

Peak#	Ret. Time	Area%
1	8.356	86.597
2	9.852	13.403
Total		100.000

HPLC Data for **29**$_{\mathbf{min}}$**:** Chiralcel OD-H (99:1 hexane : *i*PrOH, flow rate 1 mlmin^{-1}, 211 nm, 40 °C) t_R (2*R*,3*R*): 16.3 min, t_R (2*S*,3*S*): 19.0 min, 77:23 er.

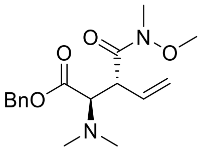

29$_{\mathbf{min}}$

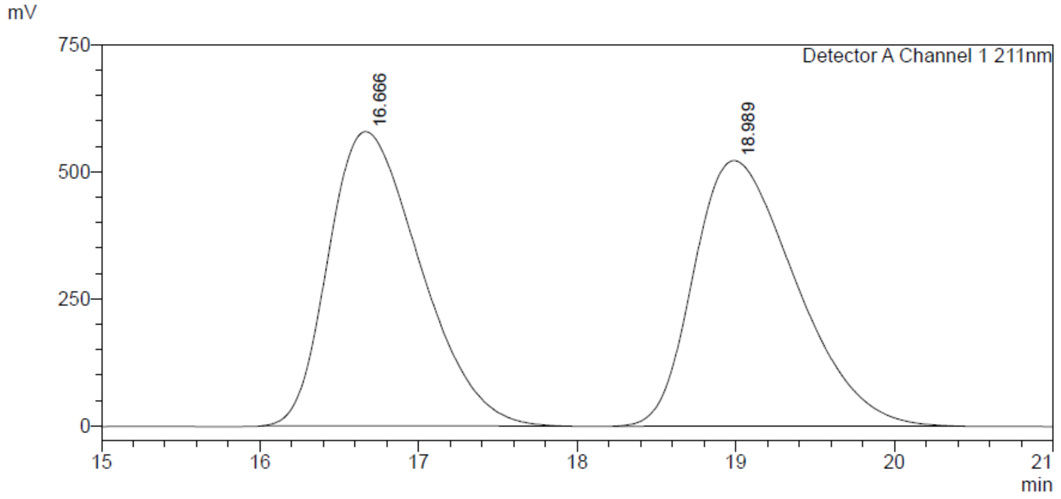

<Peak Table>

Detector A Channel 1 211nm

Peak#	Ret. Time	Area%
1	16.666	49.940
2	18.989	50.060
Total		100.000

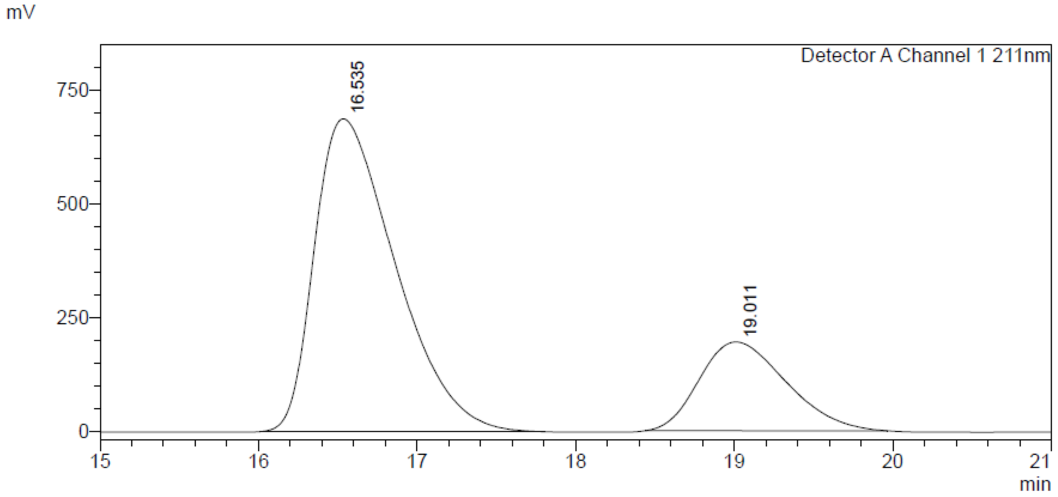

<Peak Table>

Detector A Channel 1 211nm

Peak#	Ret. Time	Area%
1	16.535	76.652
2	19.011	23.348
Total		100.000

HPLC Data for **38**$_{maj}$**:** Chiralpak AD-H (98.2:1.8 hexane : *i*PrOH, flow rate 1 mlmin^{-1}, 211 nm, 30 °C) t_R (2*R*,3*S*): 26.8 min, t_R (2*S*,3*R*): 31.4 min, 66:34 er.

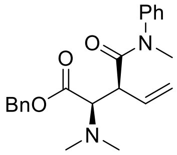

38$_{maj}$

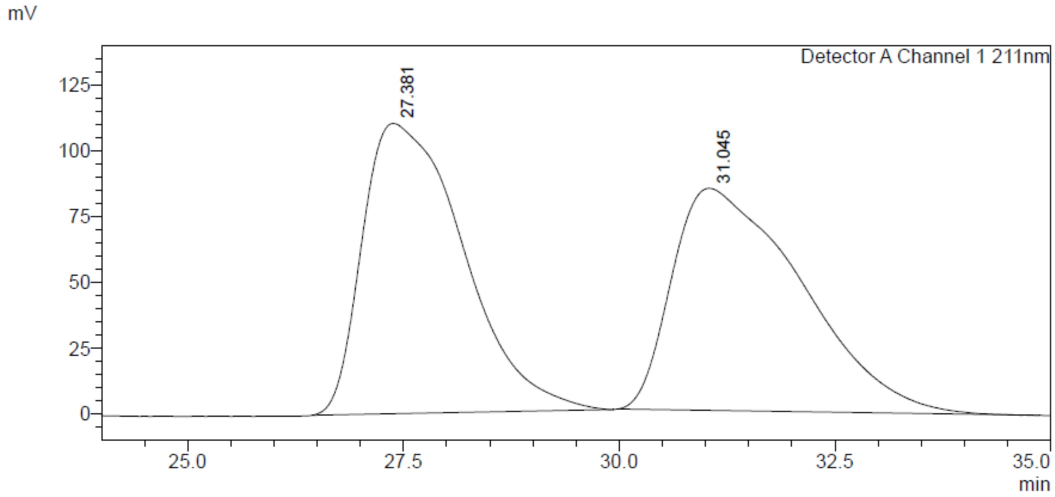

<Peak Table>

Detector A Channel 1 211nm

Peak#	Ret. Time	Area%
1	27.381	50.451
2	31.045	49.549
Total		100.000

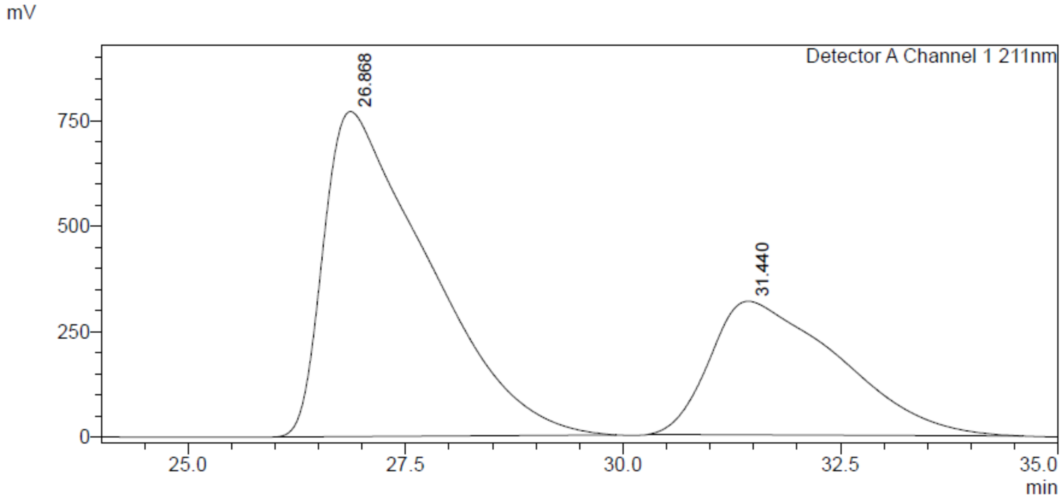

<Peak Table>

Detector A Channel 1 211nm

Peak#	Ret. Time	Area%
1	26.868	66.289
2	31.440	33.711
Total		100.000

HPLC Data for **38**min: Chiralcel OD-H (99.5:0.5 hexane : *i*PrOH, flow rate 1 mlmin^{-1}, 211 nm, 30 °C) t_R (2*R*,3*R*): 33.1 min, t_R (2*S*,3*S*): 38.4 min, 67:33 er.

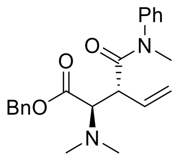

38min

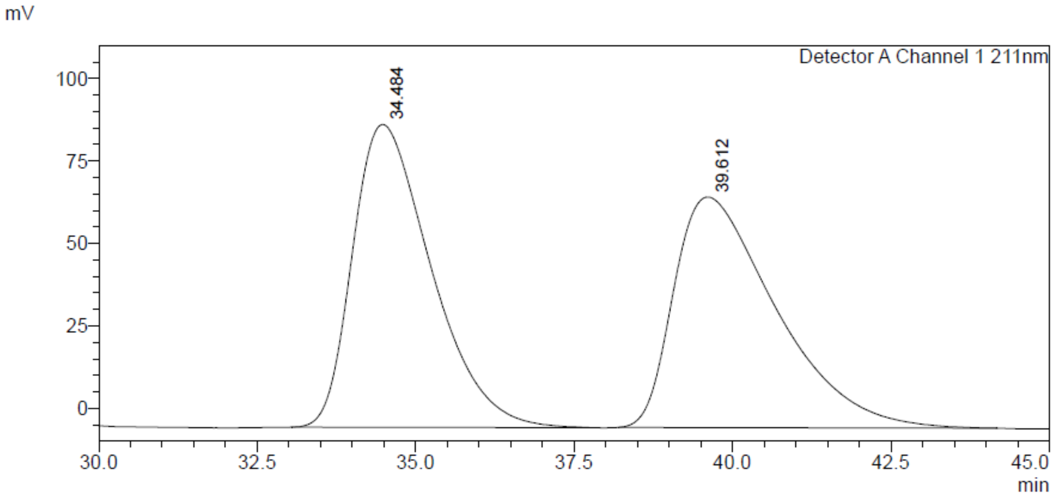

<Peak Table>

Detector A Channel 1 211nm

Peak#	Ret. Time	Area%
1	34.484	50.306
2	39.612	49.694
Total		100.000

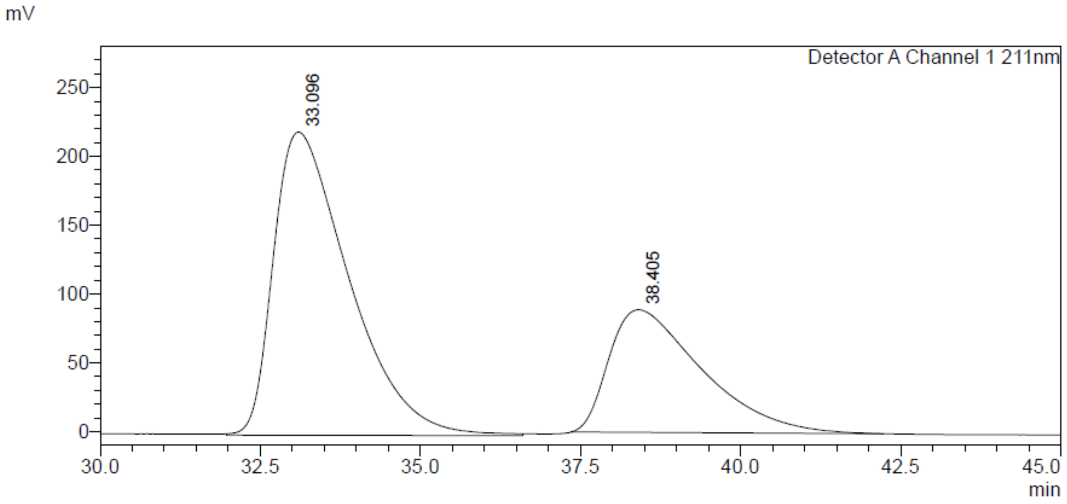

<Peak Table>

Detector A Channel 1 211nm

Peak#	Ret. Time	Area%
1	33.096	66.817
2	38.405	33.183
Total		100.000

HPLC Data for **39**$_{maj}$: Chiralcel OD-H (98:2 hexane : *i*PrOH, flow rate 1 mlmin^{-1}, 211 nm, 40 °C) t_R (2*R*,3*S*): 22.4 min, t_R (2*S*,3*R*): 29.9 min, 71:29 er.

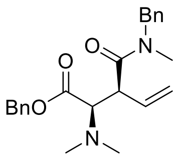

39$_{maj}$

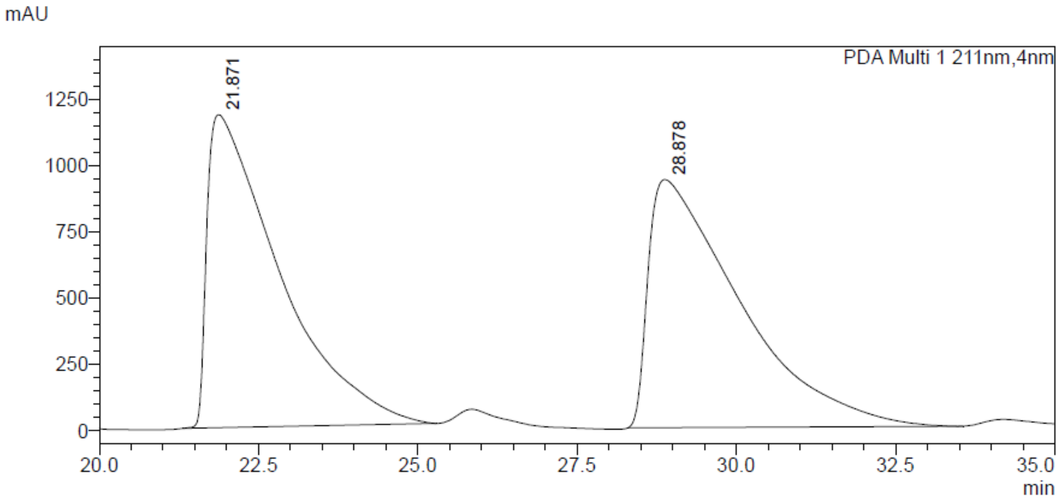

<Peak Table>

PDA Ch1 211nm

Peak#	Ret. Time	Area%
1	21.871	49.800
2	28.878	50.200
Total		100.000

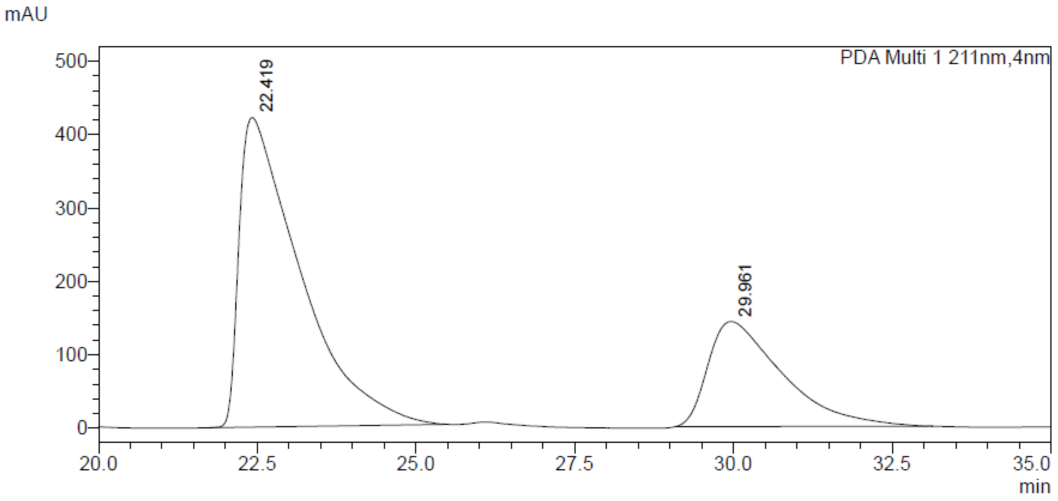

<Peak Table>

PDA Ch1 211nm

Peak#	Ret. Time	Area%
1	22.419	70.837
2	29.961	29.163
Total		100.000

HPLC Data for **39min**: Chiralcel OD-H (98:2 hexane : *i*PrOH, flow rate 1 mlmin^{-1}, 211 nm, 40 °C) t_R (2*R*,3*R*): 13.4 min, t_R (2*S*,3*S*): 20.8 min, 53:47 er.

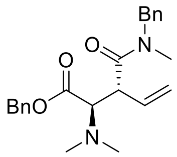

39$_{min}$

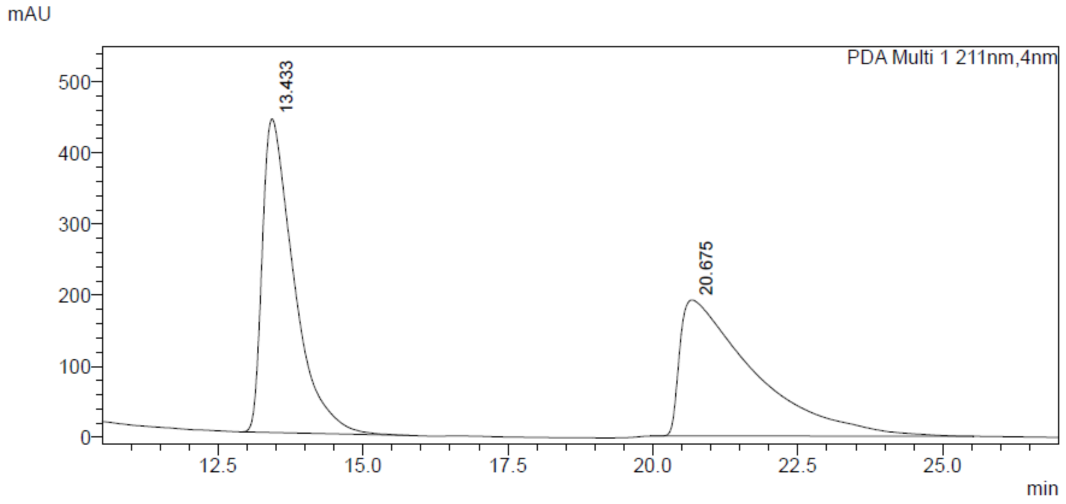

<Peak Table>

PDA Ch1 211nm

Peak#	Ret. Time	Area%
1	13.433	49.612
2	20.675	50.388
Total		100.000

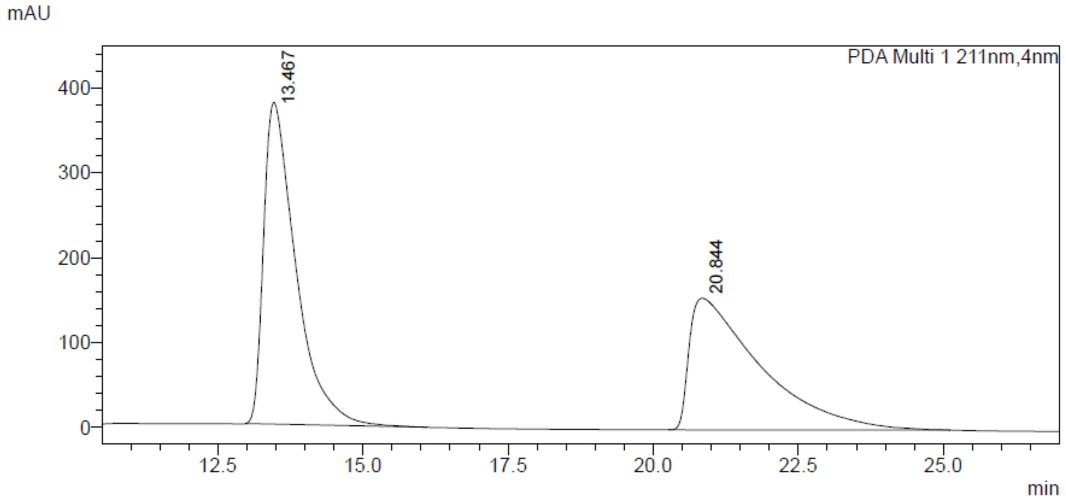

<Peak Table>

PDA Ch1 211nm

Peak#	Ret. Time	Area%
1	13.467	52.604
2	20.844	47.396
Total		100.000

HPLC Data for **40$_{maj}$**: Chiralcel OD-H (95:5 hexane : *i*PrOH, flow rate 1 mlmin^{-1}, 211 nm, 40 °C) t_R (2*R*,3*S*): 12.4 min, t_R (2*S*,3*R*): 19.0 min, 71:29 er.

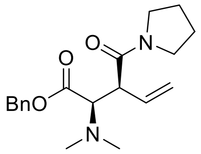

40$_{maj}$

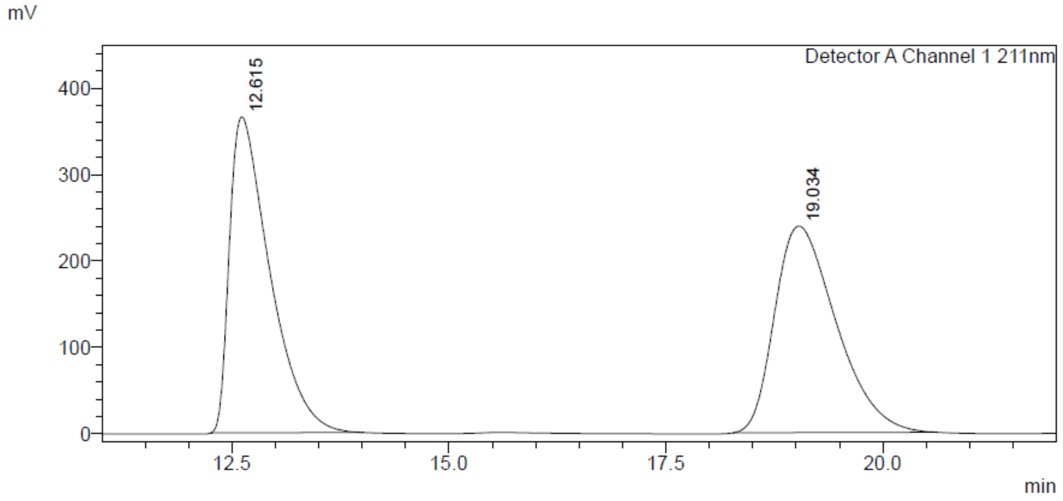

<Peak Table>

Detector A Channel 1 211nm

Peak#	Ret. Time	Area%
1	12.615	50.043
2	19.034	49.957
Total		100.000

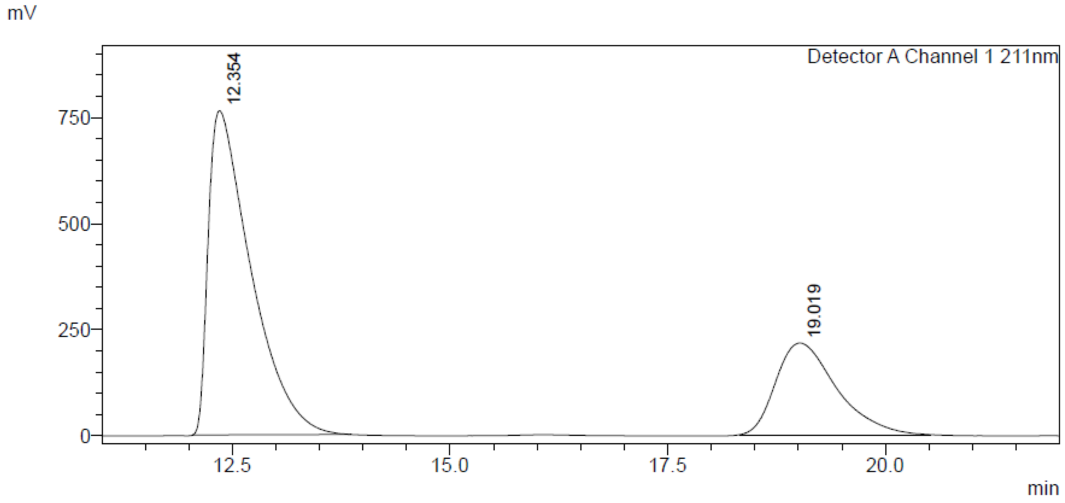

<Peak Table>

Detector A Channel 1 211nm

Peak#	Ret. Time	Area%
1	12.354	71.418
2	19.019	28.582
Total		100.000

HPLC Data for **40_{min}**: Chiralcel OD-H (95:5 hexane : *i*PrOH, flow rate 1 mlmin^{-1}, 211 nm, 40 °C) t_R (2*S*,3*S*): 7.9 min, t_R (2*R*,3*R*): 8.8 min, 66:34 er.

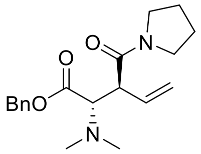

40_{min}

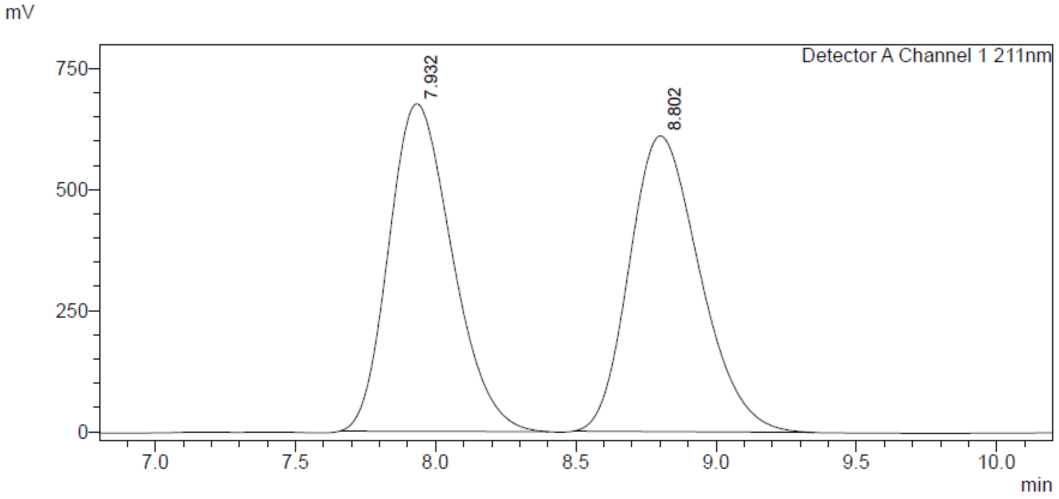

<Peak Table>

Detector A Channel 1 211nm

Peak#	Ret. Time	Area%
1	7.932	49.916
2	8.802	50.084
Total		100.000

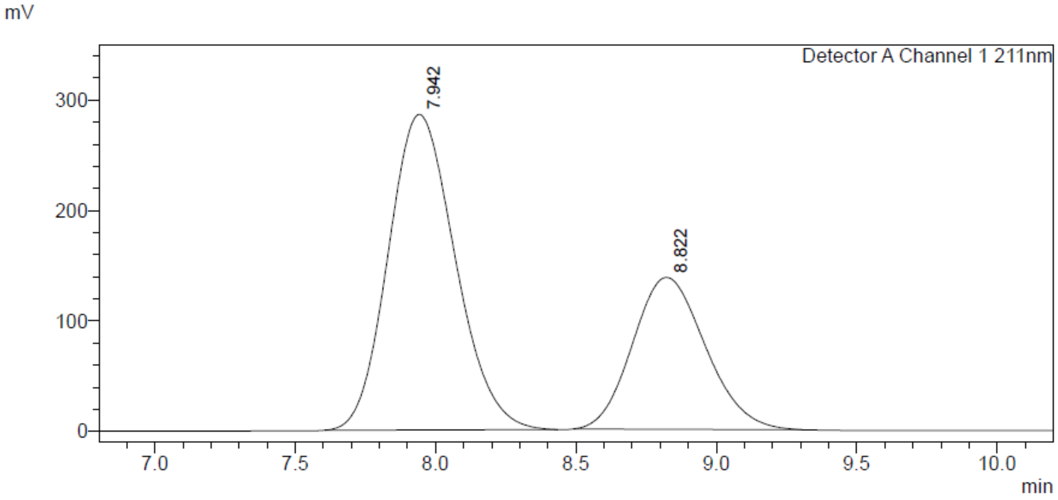

<Peak Table>

Detector A Channel 1 211nm

Peak#	Ret. Time	Area%
1	7.942	65.649
2	8.822	34.351
Total		100.000

HPLC Data for **41**$_{maj}$: Chiralcel OD-H (95:5 hexane : *i*PrOH, flow rate 1 mlmin^{-1}, 211 nm, 40 °C) t_R (2*R*,3*S*): 15.4 min, t_R (2*S*,3*R*): 18.7 min, 75:25 er.

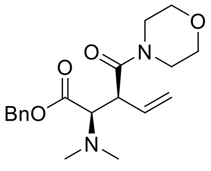

41$_{maj}$

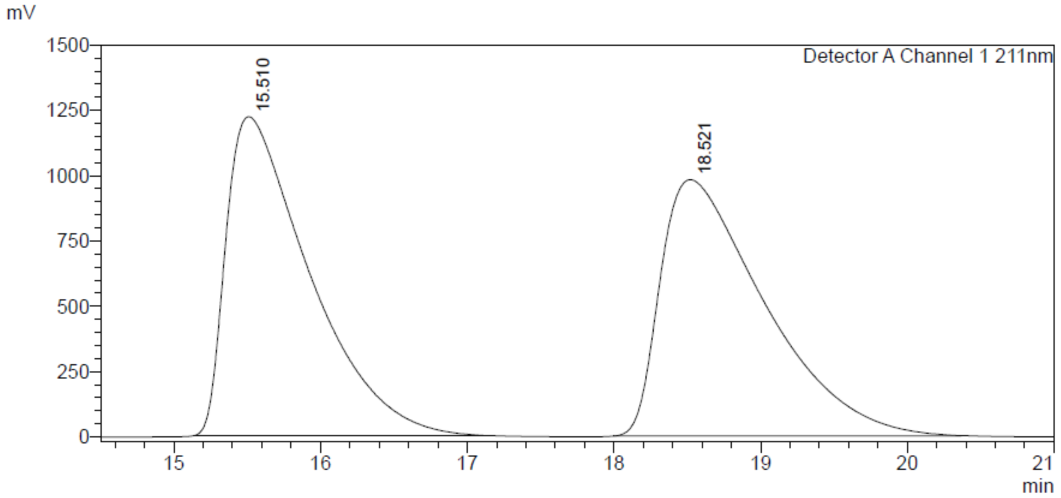

<Peak Table>

Detector A Channel 1 211nm

Peak#	Ret. Time	Area%
1	15.510	49.957
2	18.521	50.043
Total		100.000

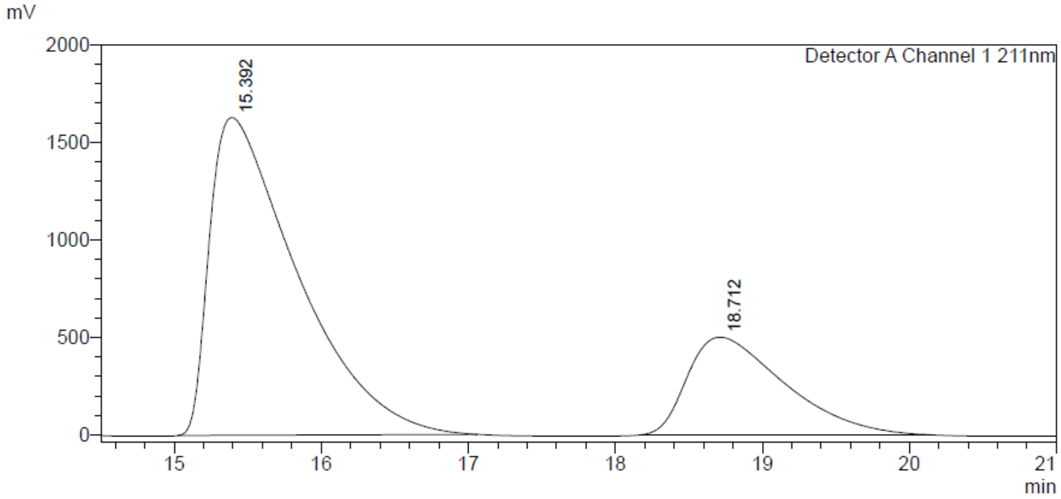

<Peak Table>

Detector A Channel 1 211nm

Peak#	Ret. Time	Area%
1	15.392	74.669
2	18.712	25.331
Total		100.000

HPLC Data for **41min**: Chiralcel OD-H (97:3 hexane : *i*PrOH, flow rate 1 mlmin^{-1}, 211 nm, 40 °C) t_R (2*S*,3*S*): 14.0 min, t_R (2*R*,3*R*): 15.9 min, 44:56 er.

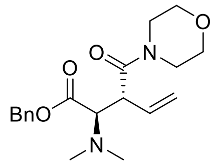

41min

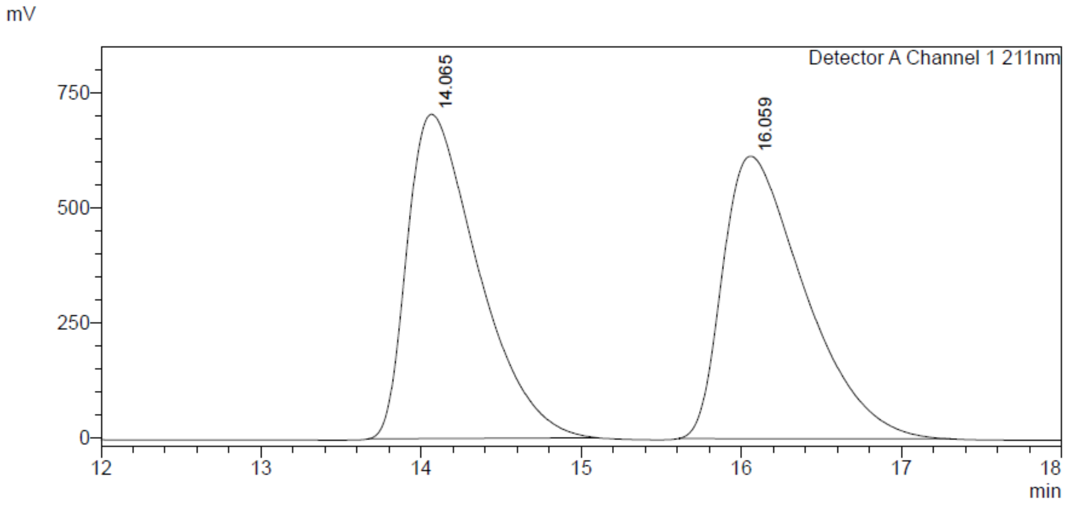

<Peak Table>

Detector A Channel 1 211nm

Peak#	Ret. Time	Area%
1	14.065	49.803
2	16.059	50.197
Total		100.000

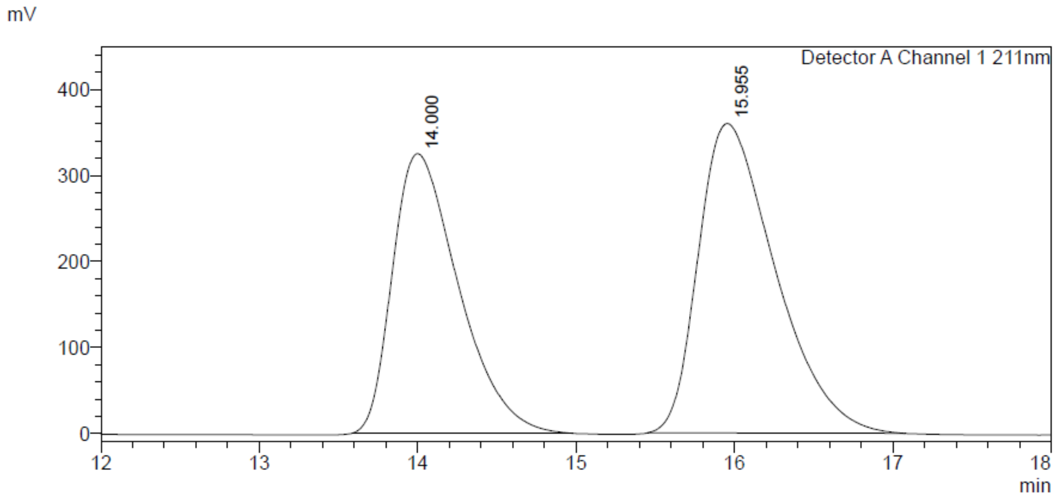

<Peak Table>

Detector A Channel 1 211nm

Peak#	Ret. Time	Area%
1	14.000	43.667
2	15.955	56.333
Total		100.000
